# Highly Strained Tricyclic Oxanorbornenes with Uncommon Reactivity Enable Rapid ROMP for Thermally High-Performing Polyenes

**DOI:** 10.1021/acs.macromol.4c02601

**Published:** 2025-04-04

**Authors:** Björn Grabbet, Abdullah Taiem, Răzvan C. Cioc, Pieter C. A. Bruijnincx, Arnaud Thevenon

**Affiliations:** Utrecht University, Organic Chemistry & Catalysis, Institute for Sustainable and Circular Chemistry, Faculty of Science, Utrecht 3584 CG, the Netherlands

## Abstract

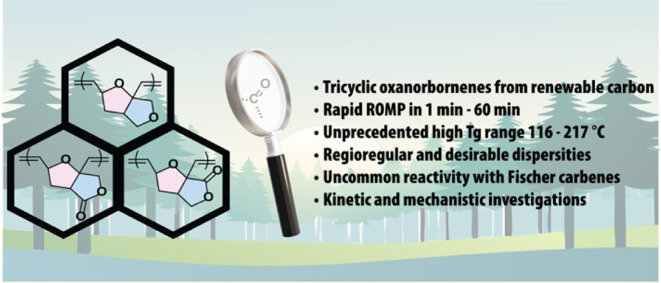

Bioderived monomers, readily available from biomass via atom- and redox-efficient processes, will need to play a major role in the development of sustainable polymeric materials. Here, we show that a family of tricyclic monomers, efficiently made from biobased furans via Diels–Alder chemistry, allows the production of polyenes with diverse thermo/physical properties through ring opening metathesis polymerization (ROMP). Via small structural variations, we offer insight into the intricacies of monomer design and its implications for polymerization. Notably, the thermostable polyenes all show very similar head-to-tail regioregularities, *trans*-linkage isomerism distributions, and narrow dispersities. Additionally, the monomers exhibit rare reactivity with ethyl vinyl ether, which can be used as a chain transfer agent, enabling the synthesis of monotelechelic polyenes. The monomers do differ substantially in polymerization rate, spanning two orders of magnitude, in the extent of molecular weight control and in the properties of the resulting amorphous polymers. With glass transition temperatures ranging from 116 to 217 °C and degradation temperatures exceeding 350 °C, these materials are among the highest performing biobased homopolymers reported. We elucidate these variations, demonstrating that the ROMP is profoundly influenced by subtle structural changes in the monomers.

## Introduction

In recent years, the concept of a circular, biobased economy has gained significant traction, which combines an emphasis on transitioning from linear to circular manufacturing processes and consumption habits with feedstock change. This transition is particularly imperative for the polymer industry, which primarily relies on petrochemical resources to produce over 250 million tons of synthetic materials yearly. Clearly, the targeted transitions require reducing emissions and mitigating environmental impact through changes in feedstock use and material design.^1,2^

This urgency is mirrored in the exploration of biobased building blocks, with furanics from renewable lignocellulosic biomass or other sugar sources, emerging as pivotal platform molecules for a more sustainable chemical industry.^3−9^ Diels–Alder cycloaddition of biobased furanics is a valorization route of particular interest, as it allows rapid, redox- and atom-efficient access to versatile and chemically rich oxanorbornene scaffolds.^10−16^ In the context of polymer chemistry, the oxanorbornene is a common motif used in ring-opening metathesis polymerization (ROMP) to prepare polyenes, offering a large chemical space to explore for the production of sustainable polymers.^17−19^ By further carefully choosing the dienophile for the Diels–Alder reaction, tricyclic structures, consisting of an oxanorbornene unit fused to a furanyl unit, can be generated which not only open possibilities for orthogonal polymerization^20^ but also offer new opportunities for modulation of the reactivity of the olefin functionality, e.g., through additional ring strain by electronic alterations.^21−23^

**Figure 1:**
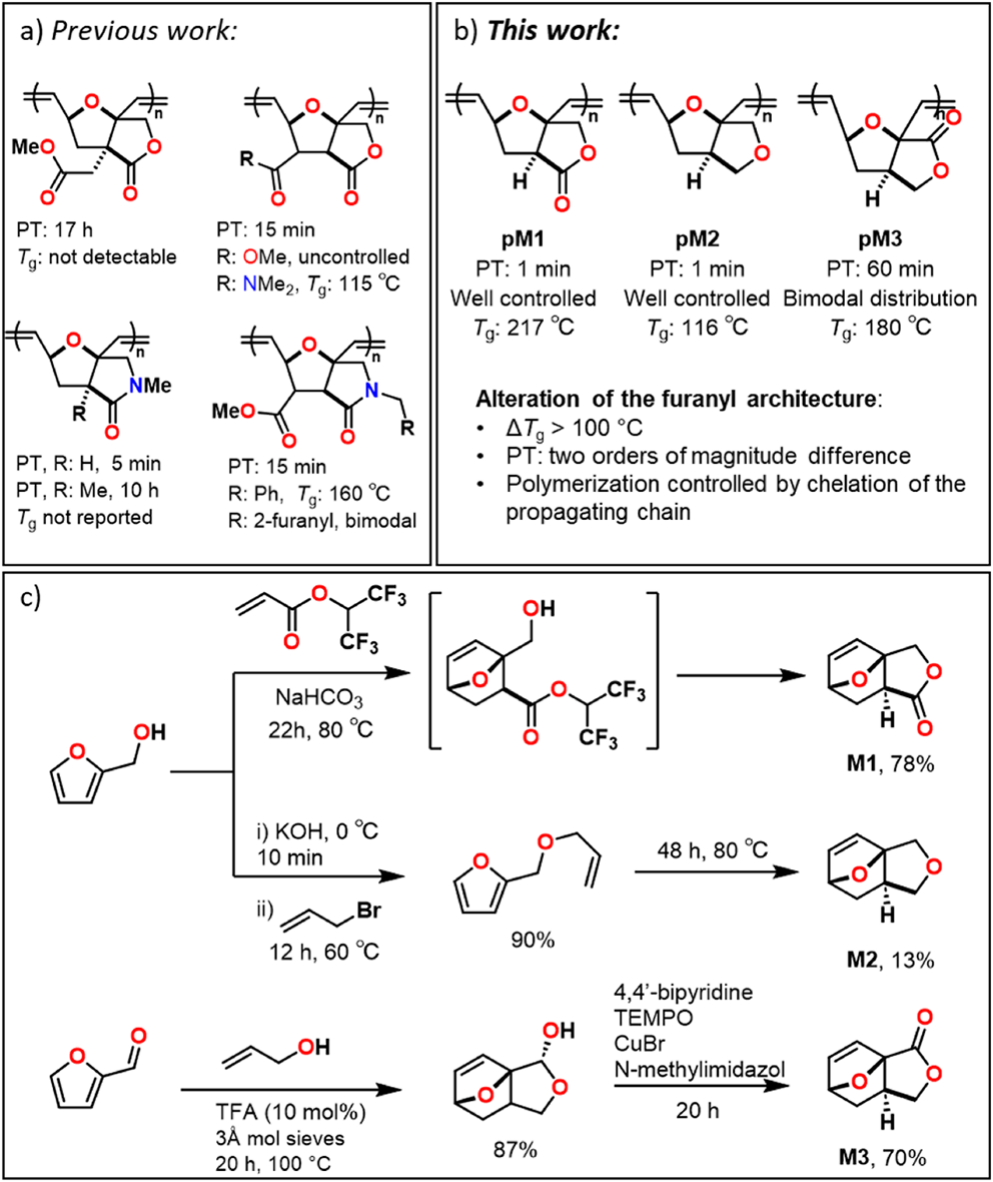
a) Reported tricyclic oxanorbornene fused furanyl monomers constructed from Diels–Alder cyclization;^21,22,24−26^ b) monomers investigated in this study; c) synthesis of the monomers.^27−30^ PT: polymerization time.

Previous reports from the groups of North et al.,^21,24,25^ Keddie et al.,^22^ and Kilbinger et al.^26^ have demonstrated that, biobased, fused tricyclic oxanorbornenes generally form polyenes via ROMP with controllable molecular weights and narrow dispersities. Interestingly, striking alterations in the rate and control of the polymerization are observed upon small structural alterations of the monomers (Figure 1a). For example, a recent study by Kilbinger et al.^26^ has shown that the introduction of an *endo*-positioned methyl group slowed down the polymerization rate by multiple orders of magnitude (Figure 1a). These structural changes also significantly affect the properties of the resulting polymer, as reflected in the large range of glass transition temperatures (*T*_g_) observed (Figure 1a). Despite these preliminary observations, a deep mechanistic understanding of how the structural properties of this oxygen-rich class of monomers affect polymerization behavior (rate + control) is still underexplored, precluding the rational design of novel materials with improved properties.

For more classical fossil-based, less oxygen-rich bicyclic norbornene substrates, mechanistic studies using third generation Grubbs catalyst from the groups of Guironnet et al.^31,32^ and Grubbs et al.^33,34^ provided detailed insight into the relationship between monomer structure, reactivity, and polymer properties. For instance, they found that with 2,3-functionalized norbornene monomers (e.g., ester, anhydride, succinimide substituted) the propagating polymer chain can chelate to the Ru center. Such chelate metallacycle formation is enforced by both the steric bulk of the NHC ligand and the geometry of the monomer. For tricyclic, oxygen-rich fused oxanorbornene monomers, the large changes observed in their polymerization behavior may also stem from the formation of chelates during polymerization. Moreover, the asymmetry of these monomers could influence monomer enchainment and ultimately the properties of the resulting polymer.

This study investigates the impact of the fused lactone or ether ring on both the mechanism of the ROMP and the resulting polymer properties. Three novel polyenes, **pM1**, **pM2**, and **pM3** (Figure 1b), are synthesized via ROMP of the tricyclic oxanorbornene fused-furanyl monomers **M1**, **M2**, and **M3** (Figure 1c), developed previously in our group.^27−30^ These monomers include two isomeric lactones and one without a carbonyl, allowing for regioisomeric chelation of the active catalyst site to be studied. We report that structural differences between the monomers significantly influence polymerization rates (up to a two-order magnitude variation), stereochemical control, and the physical properties of the polymers, such as glass transition temperatures. Notably, **pM1** exhibits one of the highest *T*_g_ values reported for biobased polymers.^35^ Furthermore, we show that ring strain can be harnessed to form monotelechelic polyenes using a chain transfer agent, although other structural factors, such as decoration on the [3,4b]furanyl motif, also critically affect ROMP kinetics. This work thus offers new insight into key design considerations for developing novel high-performance bioderived polymers.

## Results and Discussions

**Figure 2:**
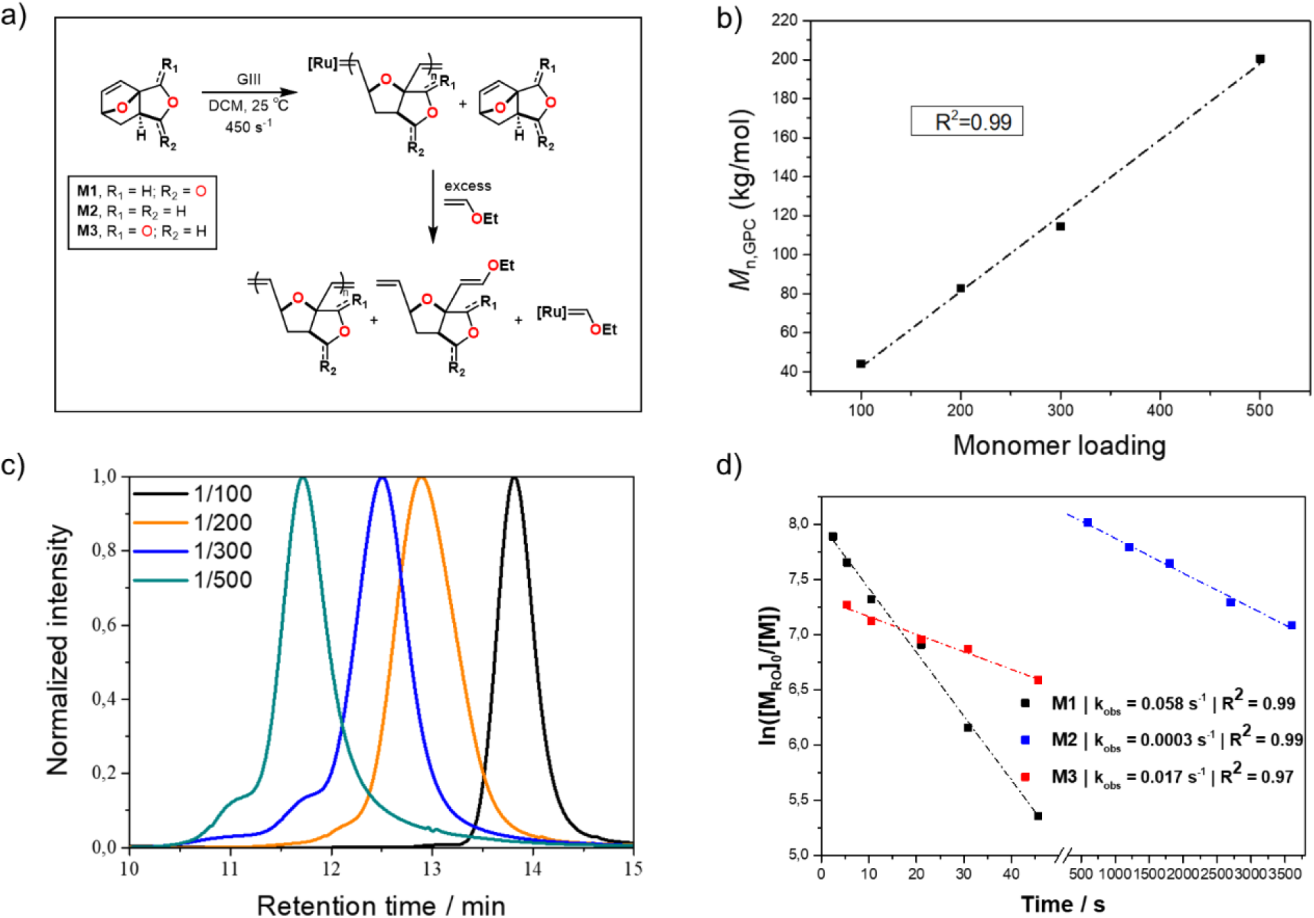
a) General scheme for polymerization reactions and nomenclature; b) linear growth of *M*_n_ with increased **M1**:catalyst (**GIII**) ratio; c) normalized GPC traces for different **M1**:catalyst (**GIII**) ratios; d) plot of propagation kinetics for determination of *k*_obs_ (Kinetic Studies section in the Supporting Information).

ROMP of **M1** in DCM, at 25 °C for 24 h, did not show any conversion with the first generation Grubbs’ (GI) catalyst, as expected,^36,37^ but second generation Grubbs (GII), second generation Hoveyda–Grubbs’ (HGII) catalysts, as well as GIII led to rapid, full monomer consumption within less than 3 min (Table 1, entries 1–4, Figure 2a). Independently of the catalyst used, the polymer from **M1** (**pM1**) precipitated from the reaction mixture during polymerization. Yet, this does not prevent the polymerization reactions to reach full conversion. **pM1** is isolated as white powder and is soluble in DMF, DMSO, and HFIP. It is processable using solvent casting, generating flexible and brittle transparent films (Figure S19). The mechanical properties of **pM1** are currently under investigation in our laboratories. When treating **pM1** with aqueous 2 M NaOH, the lactone can be hydrolyzed, yielding a water-soluble polymer (see Figures S20 and S21). Upon neutralization with HCl, the lactone reforms, causing the polymer to precipitate out from water as pristine **pM1**. The reversibility of this hydrolyzation opens up the possibility of basic aqueous recovery of the presented material from polymer blends.

**Table 1:** Catalyst Screening for the ROMP of **M1** and Catalyst Loading Variation with GIII

Entry	Catalyst	*t*/min	^[^a^]^Loading	Conv/%	^[^b^]^*M*_n(calc)_/ kg·mol^–1^	^[^c^]^*M*_n(GPC)_/ kg·mol^–1^	^[^c^]^Đ
1	GI	>1440	100	0	-	-	-
2	GII	3	100	>99	15.1	140	1.38
3	H-GII	2	100	>99	15.1	110	1.36
4	GIII	1	100	>99	15.1	44.1	1.02
5	GIII	0.75	200	85	25.9	82.9	1.06
6	GIII	0.75	300	86	39.3	114.5	1.02
7	GIII	2	500	82	62.4	200.5	1.05
8	GIII^[^d^]^	10	100	90	1.4	7.9	1.13

a Loading = [**M1**]/[catalyst].

b *M*_n_(calc) = (conversion/100) × loading × *M*_W_ (**M1**).

c Đ and *M*_n_(GPC) were determined by GPC versus polystyrene standards, with DMF as eluent, at 60 °C.

d GIII was reacted with 10 eq. of ethyl vinyl ether and stirred for 5 min before addition of **M1**.

Analysis of **pM1** made with GII and HGII shows that these polymerization reaction lacks control, as evidenced by a much higher than expected *M*_n_ and relatively large polydispersity (Table 1, entries 2 and 3), likely due to the inherently slow initiation rates typically observed with these two catalysts.^38−42^ Instead, **pM1** obtained from GIII exhibits an *M*_n_ closer to the expected value and low polydispersity (Table 1, entry 4). *M*_n_ linearly increases over the conversion and by increasing the monomer loading, demonstrating the living character of the polymerization (Table 1, entries 4–7, Figure 2b,c). To further scrutinize the living character of this system, we carried out a series of chain extension experiments by consecutive additions of monomer to an unquenched reaction mixture to demonstrate that active Ru stays attached to the propagating chains (see Supporting Information, section Chain Extension pM1). Indeed, the chains can be extended by further addition of monomer while maintaining a low polydispersity. When comparing the *M*_n(GPC)_ for 100 and 200 eq. **M1** obtained by the chain extension experiment to the *M*_n(GPC)_ as can be seen in Table 1 entries 4 and 5 it can be concluded that **M1** indeed undergoes living ROMP. It is worth noting that the chemical structure of polystyrene differs from that of the polymers studied, which may result in some discrepancies in the reported weight-averaged (*M*_w_) and number-averaged (*M*_n_) molecular weights.

Despite the low solubility of the polymer in DCM, reducing the catalyst loading enabled the generation of polymers with an *M*_n_ as high 200.5 kg·mol^–1^ (vs polystyrene standard) while maintaining low dispersity (Table 1, entry 7).

**M2** and **M3** were efficiently converted to **pM2** and **pM3**, respectively, under similar conditions as **M1** (Figure 2a, Table 2). Kinetic studies performed on the three monomers indicate a first-order rate dependence on monomer concentration. Interestingly, the rates for **M1** (*k*_obs_ = 5.8 × 10^–2^ s^–1^) and **M2** (*k*_obs_ = 1.7 × 10^–2^ s^–1^) are 2 orders of magnitude higher than the one for **M3** (*k*_obs_ = 3.0 × 10^–4^ s^–1^) and other similar bridgehead-substituted monomers, which are typically reported with *k*_obs_ values ranging from 2.0 × 10^–5^ s^–1^ to 4 × 10^–3^ s^–1^ at ambient temperature (Figure 2d).^24−26^

**Table 2:** Comparison of Formation Kinetics and Physicochemical Properties of **pM1**, **pM2,** and **pM3**

Polymer	Time/min	*k*_obs_/s^–1[^a^]^	*k*_*i*_/s^–1[^b^]^	*T*_g_/°C^[^c^]^	*T*_10%_/°C^[^d^]^	*M*_n(calc)_/kg·mol^–1^[e]	*M*_n (GPC)_/kg·mol^–1^[e]	Đ[e]
**pM1**	1	0.058	0.973	217	366	15.0	44.1	1.02
**pM2**	4	0.017	0.932	116	371	13.1	17.2	1.35
**pM3**	60	0.0003	0.004	180	374	15.0	18.2/80.4^f^	1.32/1.21^f^

a Supporting Information, Kinetic Studies section.

b determined from the slope of the linear plot of [Benzylidine]_i_/[Benzylidine]_t_ vs time, at 0 °C.

c determined through modulated differential scanning calorimetry (MDSC).

d determined by thermogravimetric analysis (TGA).

e determined by GPC with DMF as eluent, polystyrene standard.

f pM3 was found to be inherently bimodal under the conditions used for ROMP. All polymerisations were stopped at a monomer conversion >99%.

Similarly to **pM1**, **pM3** precipitates from DCM during polymerization, and the final polymer is only soluble in DMF, DMSO, and HFIP. GPC analysis shows a bimodal distribution of polymer chains, with a minor fraction having the expected *M*_n_ and a larger fraction with an above anticipated *M*_n_ (Figure S40). This distinct population of polymeric chains could potentially be caused by a complicated speciation of the propagating species (vide infra) and/or a premature termination mechanism, where the longer chains keep propagating over the course of the reaction (Figure S44). In contrast, **pM2** remains in solution during polymerization and is obtained initially as a brown viscous oil after phase separation from methanol, due to residual DCM acting as a plasticizer, but can be isolated as an off-white powder by adding its DCM solution dropwise into vigorously stirred petroleum ether (Figures S32 and S33). **pM2** dissolves easily in common organic solvents such as THF and DCM. GPC analyses confirm that **M2**’s polymerization is well-controlled, resulting in **pM2** with the expected molecular weight and a narrow molecular weight distribution.

**Figure 3:**
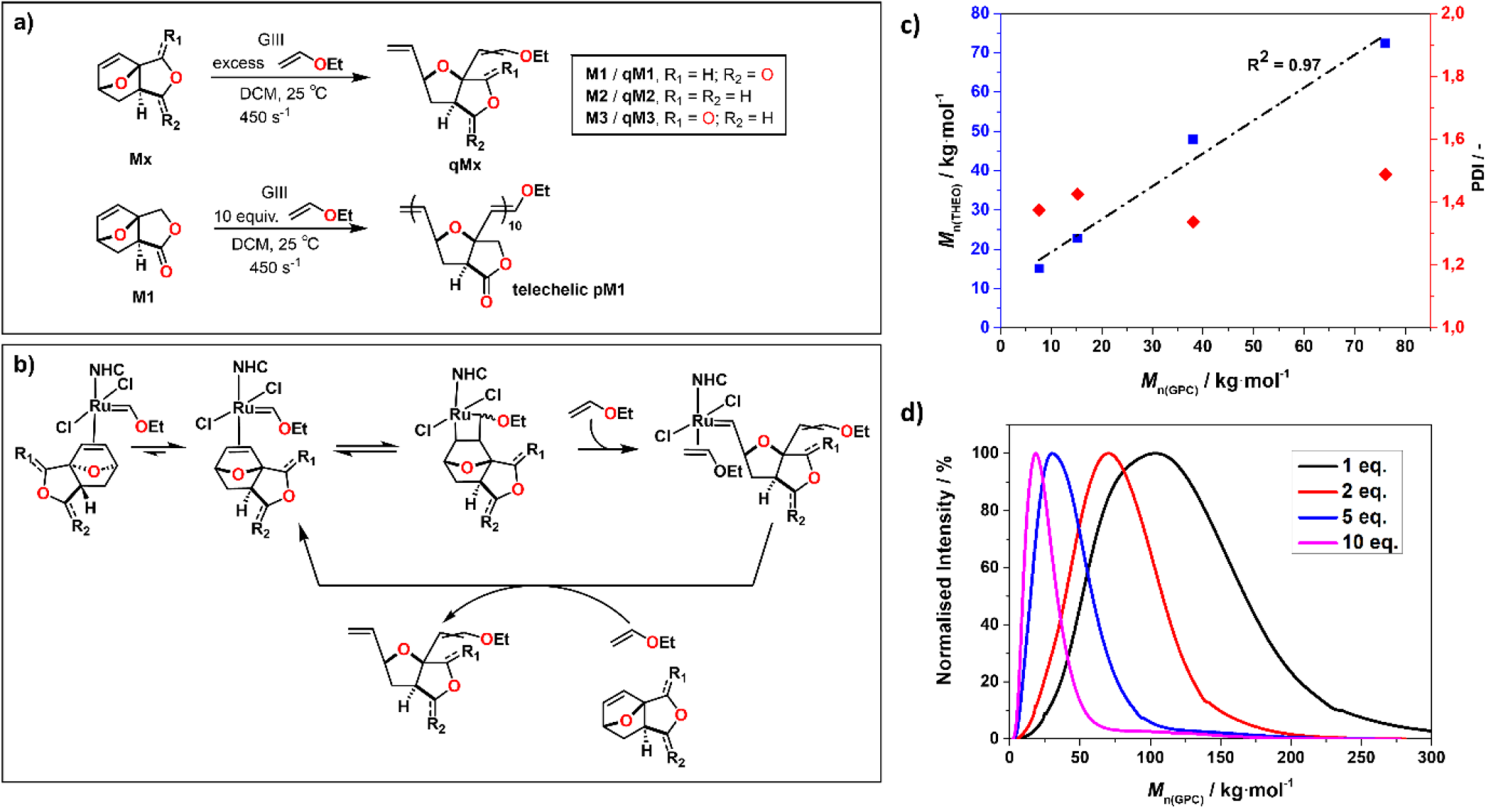
a) Reactivity of the monomers in the presence of GIII + ethyl vinyl ether; b) proposed mechanism to explain the observed regioselectivity; c) plot of *M*_n(Theo)_ (*M*_(M1)_ × monomer loading) against *M*_n(GPC)_ to highlight the linearity of chain transfer extension while maintaining narrow polydispersity; d) GPC traces of chain transfer experiment.

Interestingly, independently of the monomer used, we consistently observed that all unreacted monomers were cleanly converted into two distinct new compounds (one for **M3**) upon quenching polymerization reactions with ethyl vinyl ether (Figure 2a). These new species, quantitatively prepared by reacting a premixed solution of an excess of ethyl vinyl ether and GIII (1 mol %) with the corresponding monomers (Figure 3a, top), were unambiguously identified as *cis*-6a-(2-ethoxyvinyl)-2-vinyl-tetrahydrofuran-[3,4-*b*]furanyl (*cis*-**qM1**, *cis*-**qM2**) and *trans*-6a-(2-ethoxyvinyl)-2-vinyl-tetrahydrofuran-[3,4-*b*]furanyl (*trans*-**qM1**, *trans*-**qM2** and *trans*-**qM3**) by multinuclear and 2D NMR spectroscopy (Supprting Information, section Prequenched reactions). We presume that these ring-opened products are formed from ring opening metathesis with the Fischer carbene complex formed upon the addition of ethyl vinyl ether (Figure 3b). We also want to point out the perfect regioselectivity of the reaction as well as that only the *trans* product was formed from **M3**.

Inspired by previous works on chain transfer agents in ROMP,^43−47^ we aim to leverage this unusual reactivity to investigate whether ethyl vinyl ether could be used as a chain transfer agent during ROMP of **M1** (Figure 3a, bottom; Table 1, entry 8). Accordingly, a polymerization reaction was performed by adding a solution of **M1** to a premixed solution of GIII and 10 eq. of ethyl vinyl ether (immortal conditions) affording ethyl vinyl ether end-capped polymers with low *M*_n_ and narrow dispersity. This suggests that ethyl vinyl ether can be used as an efficient chain transfer agent in combination with this series of monomers (Supporting Information, section Immortal Conditions). We exploited this reactivity to investigate molecular weight control by chain transfer for **M1**. For that we added defined equivalents (1, 2, 5, and 10) of ethyl vinyl ether to GIII and reacted this with 500 eq. **M1** which afforded materials with the expected *M*_n_, albeit with a higher polydispersity (see the Supporting Information, section Chain transfer control). This opens a new possibility to control the end group of the polymer chains and to form monotelechelic polymers for the synthesis of (multi)block copolymers, tethering them to surfaces, or targeting higher architectures such as brush polymers or dendritic materials.^48−50^

**Figure 4:**
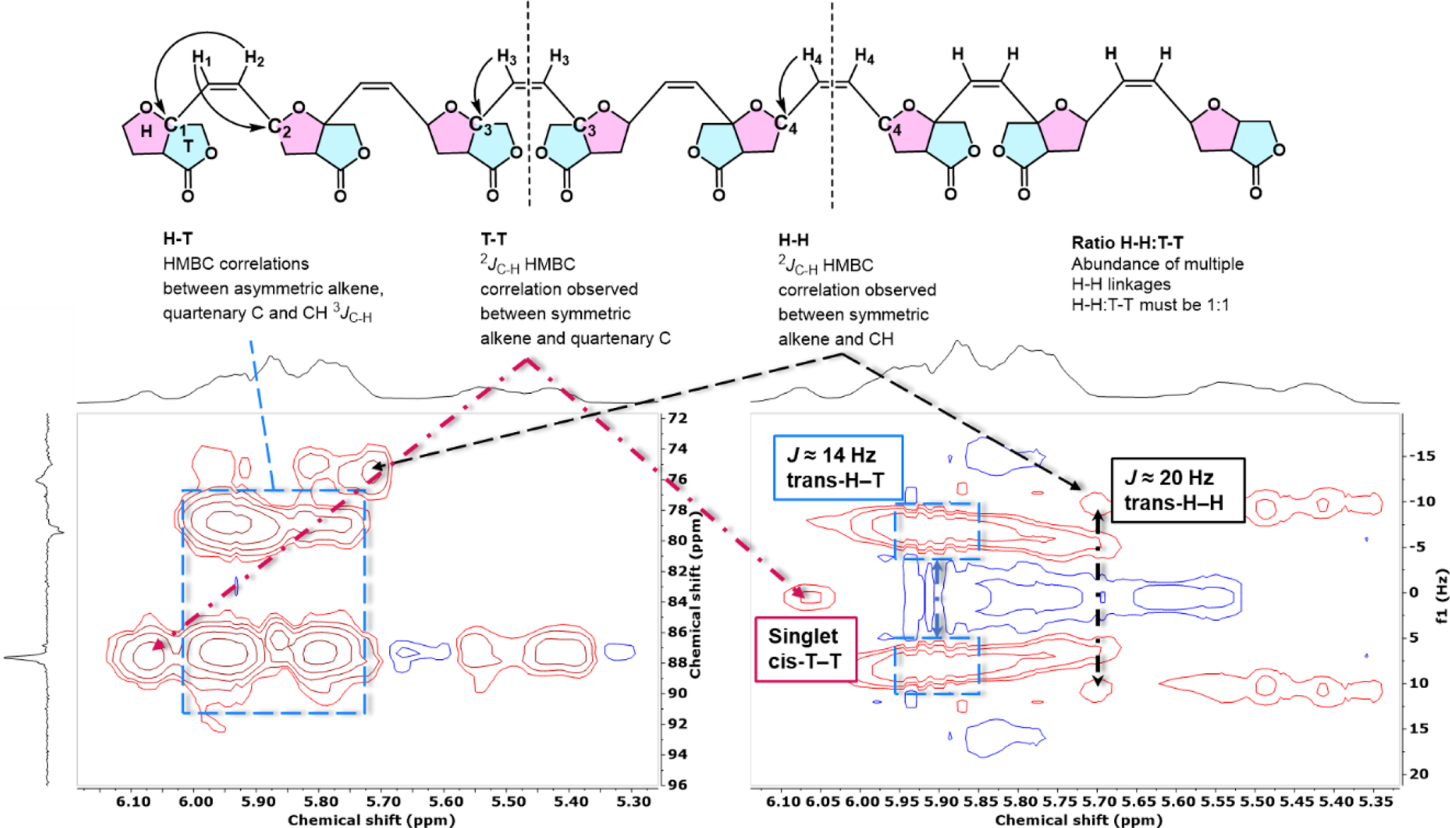
Rationale behind the assignment of linkages. HMBC (left) and JRES (right) spectra are both from **pM1**.

In addition to this unexpected reactivity, the perfect regioselectivity observed during the ring opening metathesis with excess ethyl vinyl ether suggests that there is a preference for the monomer to coordinate to Ru with the furanyl unit pointing toward the benzylidene carbene (Figure 3b). This prompted us to scrutinize the regioselectivity of monomer enchainment in **pM1–3** using NMR spectroscopy, designating the oxanorbornene ring as the head (H) and the [3,4-*b*]-furanyl subunit as the tail (T) of the monomer (Figure 4). The regioregularity of the polymers was determined from the polymers’ (^1^H–^13^C)-HMBC, (^1^H–^13^C)-HSQC, and *J*-resolved (JRES) NMR spectra. Since the ^1^H NMR spectra of the polymers showed significant signal broadening, JRES was chosen as a technique to better identify the underlying multiplicity and thus assign the geometrical isomerism of the linkages. The full description of the data interpretation is given in the Supporting Information. Briefly, we postulate that independently of geometrical isomerism, an H–T connection would lead to two inequivalent H–C_sp2_ resonances of the alkene (H1 and H2). These are detectable in HMBC by looking at ^2/3^*J*_C–H_ coupling between H1 and C1/C2 and ^2/ 3^*J*_C–H_ coupling between H2 and C1/C2. On the other hand, H–H or T–T linkages give equivalent H–C_sp2_ resonances of the alkene (H3 or H4). The resulting HMBC will therefore give only one associated ^2^*J*_C–H_ coupling between H3 and C3 (T–T) or H4 and C4 (H–H) atoms. We want to point out that statistically the ratio between H–H and T–T must be 1:1 since two consecutive H–H or T–T linkages are impossible. The contribution of each geometrical isomerism was established by identifying ^1^H resonances from *cis* (8 Hz) and *trans* (14/20 Hz) linkages using *J*-resolved NMR. The percentiles were then estimated by integration of the corresponding HSQC spectrum providing an approximation of each of the different connections.

For conciseness, only the assignment of **pM1** will be discussed here (Figure 4), and a discussion on **pM2** and **pM3** connectivity is included in the Supporting Information (Assignment Linkages section). The ^1^H–^13^C HMBC of **pM1** shows a cluster of four ^1^H resonances (5.96, 5.80 ppm) showing coupling to both quaternary (87.7 ppm) and tertiary carbon (79.1 ppm) resonances, indicating an H–T link. From the recorded JRES NMR spectrum, it can be derived that these H–T linkages are mostly the *trans* isomer given that *J* = 14 Hz. Three distinct cross peaks are observed that only show coupling to the quaternary carbon resonance at 87.7 ppm, indicating a T–T connection. JRES NMR shows the proton signal at 6.07 ppm to be a singlet, which would correlate to a T–T_cis_ connectivity, while the resonances at 5.55 and 5.41 ppm exhibit and underlying doublet multiplicity with a coupling of *J* = 20 Hz, suggesting a T–T_trans_ connection. With the resonances at 5.96, 5.80, and 5.72 ppm only displaying coupling to the tertiary carbon resonance at 79.1 ppm (overlap with H–T), they are assigned to be H–H connections. Normalized integration of the relevant peak areas in the HSQC spectrum gave 18% T–T and, by necessity, 18% H–H linkages, the latter consisting of at least 4% H–H_cis_ connectivity. Accurate direct quantification of H–T linkage abundance was not possible due to peaks overlapping, but based on the argument that the number of T–T linkages is equal to the number of H–H linkages, we deduct that H–T linkages are the principal linkages in the polymer accounting for at least 60% of all linkages. Furthermore, we also determined that most of the linkages are *trans.*

A similar approach was used for **pM2** and **pM3**. Similarly to **pM1**, we found that **pM2** and **pM3** principally consist of H–T (66% in **pM2**, 60% in **pM3**); the geometrical isomerism could not be estimated due to peaks overlapping. These results are in line with closely related tricyclic oxanorbornene monomers.^22^ This suggests that while the furanyl unit itself influences the monomer enchainment during polymerization, any functional decoration of the fused furanyl unit has only a little influence. However, the discrepancy between the perfect regioselectivity observed for the ring opening metathesis of a single monomer and the monomer enchainment in the polymers suggests that the growing polymer chain is somehow responsible for introducing regioirregularity within the polymer chain, an observation that warrants further follow-up investigation.

The structural variation of the furanyl unit in the monomers is reflected in the thermal properties of the polymers, as evidenced by TGA (Figures S17, S33 and S43) and modulated differential scanning calorimetry (MDSC, Figures S15, S32 and S42). The polymers are characterized by high thermal stability and an amorphous macrostructure. To our surprise, the recorded glass transition temperatures span a range of over 100 °C, despite the subtle structural differences of the furanyl units, comparable *M*_n_, polydispersity, and monomer enchainment between each polymer. Specifically, **pM1** showed one of the highest glass transition (*T*_*g*_ = 217 °C) thus far observed for a homopolymer made from tricyclic oxanorbornene derived monomers.^35^ It is worth mentioning that an endothermic event (Δ*H*_norm_ = +1.5 J/g) is concomitantly observed in the *T*_g_ region of **pM1**. This event has been attributed to enthalpic recovery since a powder X-ray diffraction (pXRD) measurement indicated that the polymer is completely amorphous (Figure S18).^51^**pM1** has a high decomposition temperature (*T*_10%_ = 367 °C) and showed no significant loss of mass even under prolonged heating (12 h, 200 °C, N_2_ atmosphere, Figure S16). Being a structural isomer of **pM1**, **pM3** shows a slightly lower *T*_g_ of 180 °C and a similar *T*_10%_ of 374 °C. It is worth noting that the slight decrease in *T*_g_ is likely due to the plasticization effect of the lower molecular weight fractions, as indicated by the GPC chromatogram (see Figure S40). In sharp contrast, the recorded glass transition temperature of **pM2** is approximately 100 °C lower in comparison to **pM1** (*T*_g_ = 116 °C). This demonstrates the large contribution of the more rigid furanyl ring in **pM1** and **pM3**, most likely by reducing the chain flexibility, and therefore significantly increasing the *T*_g_ of the resulting material. The structural differences between **pM1** and **pM3** suggest that a difference in dipole moment does not significantly contribute to the observed differences in thermal properties (Table S8). Weaker interactions were not quantified, so we cannot definitively determine whether carbonyl groups reduce chain mobility or affect intermolecular interactions.

**Figure 5:**
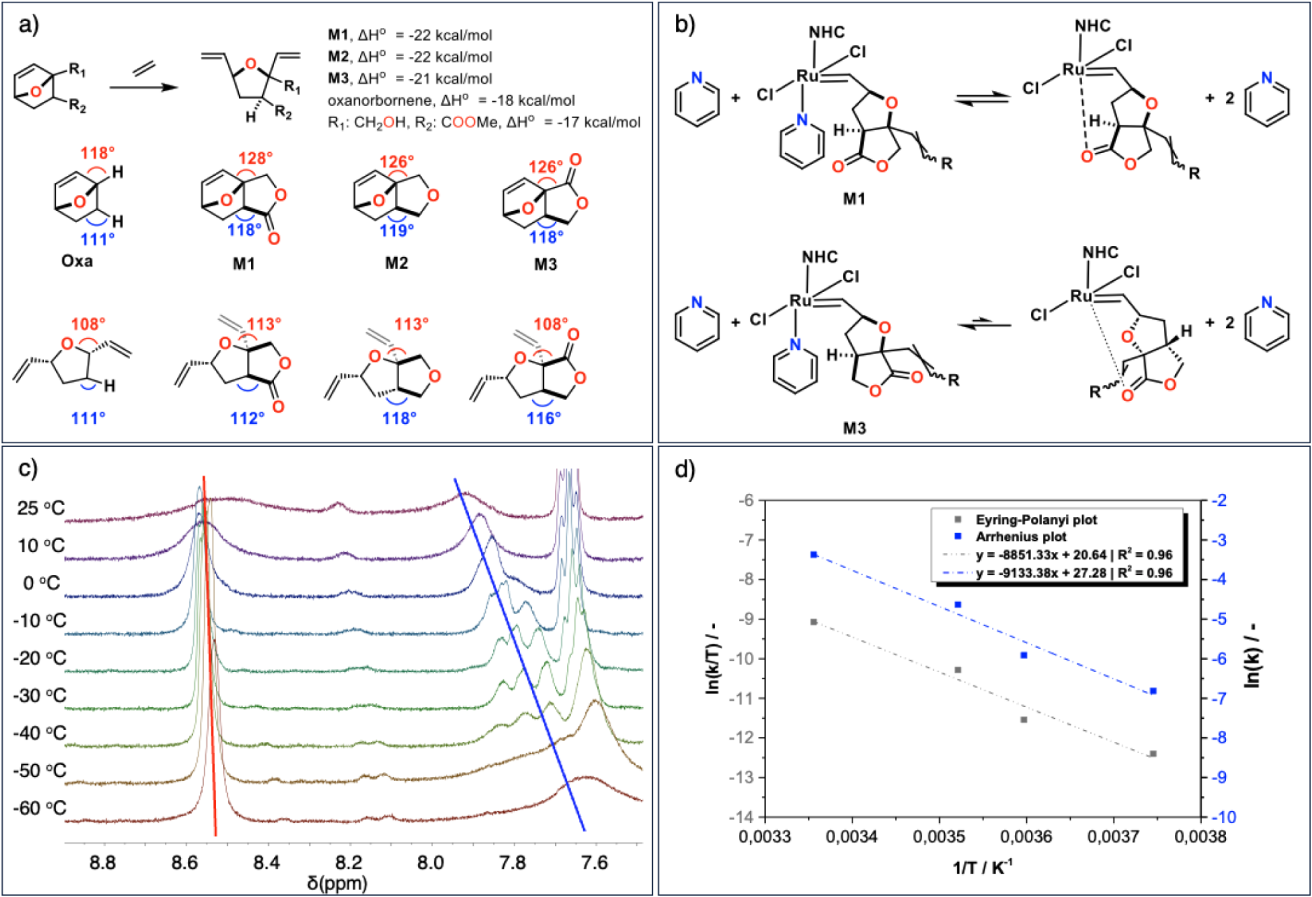
a) Comparison of *in silico*-determined ring strain energies for the series of oxanorbornene-derived monomers. Depicted on the right are the calculated angles at the fused bridgehead position before and after a simulated metathesis with ethylene (B3LYP-GD3BJ/6-311+G(d)); b) schematic representation of the potential chelation modes of the last inserted monomer to Ru, highlighting that in the case of **M3**, the carbonyl is not well positioned to form a chelate with Ru; c) overlay of ^1^H NMR (CD_2_Cl_2_) at various temperature ranging from −60 to 25 °C showing free pyridine at 8.55 ppm (highlighted with a red line) and coordinated pyridine between 7.6 and 8 ppm (highlighted with a blue line) for the oligo-**M1** bound to Ru; c) combined Eyring–Polanyi and Arrhenius analysis for **M1**.

The unusual reactivity of this series of monomers prompted us to perform more mechanistic studies (Figure 5). First, density functional theory (DFT) calculations were performed to investigate structural differences and the ring strain imposed by the fused furanyl unit (Figure 5a). It is worth noting that the *in-silico-*determined structural parameters of **M1** and **M3** are in good agreement with the data obtained from single crystal X-ray crystallography.^20,29^ Analysis of the bond angles of the two C atoms found at the junction of the oxanorbornene ring with the furanyl ring clearly shows anomalously large values (7–10° difference) compared to the parent unsubstituted oxanorbornene as well as the lactone ring-opened furanyl derivative (Supporting Information, section Computational Details).^29^ The ring strain imposed by the fused furanyl unit was further estimated following a method reported by Coates et al.^52,53^ Calculations show that **M1**, **M2,** and **M3** have similarly high ring strains of ∼22 kcal/mol, which is ∼4–5 kcal/mol higher than that of oxanorbornene and structurally related bridgehead substituted monomers. While ring strain does not explain the difference in reactivity between **M1** and **M3**, the significant difference with other oxanorbornene monomers could explain the propensity of **M1**, **M2,** and **M3** to react with (otherwise) quenched Grubbs’ catalysts.

We then went on to probe whether the incoming monomer (monomer control) and the speciation of GIII during the propagating step contribute to the difference in the polymerization rate between **M1** and **M3** (Supporting Information, section Kinetic Studies). As previously studied by Guironnet, the extent of the monomer control can be experimentally evaluated by studying the difference in the initiation rate of GIII with the two monomers.^32^ Monitoring the disappearance of the benzylidene peak in GIII upon the addition of 10 eq. of monomers by ^1^H NMR spectroscopy, at 0 °C showed that the initiation rate for the polymerization of **M1** (0.9 s^–1^) is around 2 orders of magnitude faster than **M3** (0.004 s^–1^) (Figure S112). The one-order-of-magnitude difference in propagation rates indicates that the position of the oxo-functionality on the furanyl unit plays a crucial role in the overall difference in rates observed, most likely through geometrically limited interactions between the growing polymer chain and the catalyst.

The speciation of the propagating chains was then investigated by looking at the alkylidene region once the initiation step was completed. Due to the asymmetry of the monomer, we are expecting to have two distinct alkylidene resonances depending if the last ring opened monomer presents the furanyl unit pointing toward or away from Ru.^34^ Indeed, two main resonances are observed between 18.3 and 18.9 ppm for **M1** and **M2** which are in good agreement to previously assigned to the alkylidene proton from the last ring opened monomer coordinated to Ru (Figures S107 and S109).^42^ For **M3**, significantly more resonances are observed suggesting a much more complex speciation of the catalyst during the propagation phase (Figure S111). Each of these propagation states is characterized by its own propagation rate, which ultimately influences the overall rate of the reaction, which could explain the broad multimodal distribution observed in **pM3**.

To further study the difference in the speciation of the propagating chain between **M1** and **M3**, we investigated whether the growing polymer chain would chelate to the metal center during the polymerization reaction (Figure 5b).^32,34^ To this extent, GIII was reacted with 10 eq. of **M1** or **M3** in CD_2_Cl_2_ to generate an oligomeric chain coordinated to Ru. The resulting reaction mixtures were analyzed by ^1^H NMR at different temperatures (Figure 5c). The chelation propensity of the growing polymer chain was studied by analyzing the resonances at 8.6 ppm and between 7.6 and 7.8 ppm, assigned by Guironnet et al. as the *ortho* proton of free and coordinated pyridine, respectively.^32,34^ With **M1**, the coalescence of these peaks at 25 °C indicates rapid and reversible chelation to Ru, consistent with previous findings by Grubbs et al. and Guironnet et al.^32,34^ This was further supported by determining the enthalpy (Δ*H*‡ = 17 kcal·mol^–1^) and entropy (Δ*S*‡ = −35 J·mol·K^–^^1^ of activation from an Eyring plot (Figure 5d). While the measured parameters can reflect contributions from several reversible steps, such large negative entropy has previously been observed by Grubbs and attributed to the chelation of the propagating chain.^34^

The situation with **M3** is more complex. It appears that there are two types of coordinated pyridine. However, at temperatures above −20 °C the data suggest that there is no chain chelation (no peak coalescence, and both pyridine peaks integrate to two protons, as expected). Below −20 °C, the data show that there is more uncoordinated than coordinated pyridine. Chelation of the last inserted monomer is unlikely with **M3** since the carbonyl is thought to be not ideally positioned apically to the central ruthenium (Figure 5b). This could reflect weak intermolecular interactions of the growing polymer chains with Ru. Additionally, not all monomers were consumed, indicating the possible unproductive coordination of monomers. Based on all these results, we attributed the difference observed between **M1** and **M3** to the propensity of the last inserted monomer of **M1** to chelate to Ru. This in turn maintains the well-defined speciation of Ru throughout the polymerization reaction, enabling controlled polymerization of **M1**. Even though chelating monomers have demonstrated lower polymerization rates than nonchelating monomers,^32,34,42^ we attributed the exceptionally fast reactivity of **M1** to the high ring strain of the oxanorbornene ring.

## Conclusion

To conclude, we report three novel polyenes synthesized by ROMP from readily available biobased monomers and demonstrate that subtle structural variations in the fused furanyl unit (structural isomerism in **M1** and **M3** or the absence of a carbonyl group in **M2**) significantly impact reactivity, polymer chemical structure, and resulting polymer properties in the following ways.

First, polymerization rates differ by two orders of magnitude between **M1/M2** and **M3**, with **M1/M2** producing polymers with controlled *M*_n_ and narrow dispersity, while **M3** yields polymers characterized by a bimodal distribution. The high ring strain in these monomers not only leads to exceptionally fast polymerization but also enables the use of ethyl vinyl ether as a chain-transfer agent, allowing for the preparation of monotelechelic polymers. Interestingly, it is not only the ring strain of the oxanorbornene motif that drives high ROMP activity and ensures controlled polymerization but also predominantly the furanyl ring itself, which strongly influences initiation and speciation during the propagation step. We found that judiciously positioning the carbonyl on the furanyl ring can promote chelation of the last inserted monomer to ruthenium, preventing unproductive monomer coordination or undesirable intermolecular interactions with other propagating chains.

Second, the glass transition temperature (*T*_g_) of the resulting polymers varies by over 100 °C, with **pM1** exhibiting the highest *T*_g_ reported for a biobased nonaromatic homopolymer at 217 °C. In depth characterization of the polymer microstructures ruled out the possibility of different monomer enchainment in the polymer chains since all polymers have predominantly *trans* H–T linkages. Instead, the more rigid furanyl ring in **pM1** and **pM3**, most likely reduces the chain flexibility which significantly increasing the *T*_g_ of the resulting material compared to the control polymer **pM2** (116 °C).

Overall, this study highlights how subtle structural variations in the monomers impact the ROMP behavior and polymer properties. Moreover, it demonstrates that traditional thermodynamic descriptors, such as ring strain, are insufficient for predicting reactivity in oxygen-rich monomers such as tricyclic oxanorbornenes. These findings provide valuable mechanistic insights into ROMP, offering implications for designing new biobased polymers with advanced properties.

## Supporting Information Available

Experimental details, characterizations, and additional optimization tables are available in the Supporting Information. Additional spectroscopic, thermoanalytic, chromatographic, and computational data files that support the findings of this study are openly available in the Yoda data repository at https://doi.org/10.24416/UU01-TTN3X8. The authors have cited additional references within the Supporting Information.^54-59^ (PDF)
