# Supplementary material for: Highly Strained Tricyclic Oxanorbornenes with Uncommon Reactivity Enable Rapid ROMP for Thermally High-Performing Polyenes
Source: Macromolecules. 2025 Apr 4;58(8):4215–24. doi: 10.1021/acs.macromol.4c02601 (PMC12020418; doi:10.1021/acs.macromol.4c02601)
Supplement: Supplementary file 1 — ma4c02601_si_001.pdf [file ma4c02601_si_001.pdf]

SUPPORTING INFORMATION

Highly Strained Tricyclic Oxanorbornenes with Uncommon Reactivity Enable Rapid ROMP for Thermally High-Performing Polyenes

Björn Grabbet,[a] Abdullah Taiem,[a] Răzvan C. Cioc,[a] Pieter C. A. Bruijnincx,*[a] Arnaud Thevenon*[a]

Contents

General considerations ... 2
Monomer synthesis[2,3] ... 3
Polymer synthesis ... 5
pM1 ... 5
pM2 ... 18
pM3 ... 25
Pre-quenched reactions ... 33
qM1 ... 33
qM2 ... 39
trans-qM3 ... 45
Immortal conditions ... 49
Chain transfer control ... 58
Chain extension pM1 ... 65
Assignment linkages ... 72
Assignment linkages pM1 ... 73
Assignment linkages pM2 ... 74
Assignment linkages pM3 ... 75
Kinetic studies ... 77
Chelation studies[4] ... 84
Eyring-Polanyi and Arrhenius analysis M1 ... 87
Computational details for mechanistic considerations ... 89
References ... 95

a B. Grabbet, A. Taiem, dr. R.C. Cioc, prof. dr. P.C.A. Bruijnincx, dr. A.A. Thevenon-Kozub
Utrecht University, Organic Chemistry & Catalysis, Institute for Sustainable and Circular Chemistry, Faculty of Science, Utrecht, The Netherlands
E-mail: a.a.thevenon-kozub@uu.nl; p.c.a.bruijnincx@uu.nl

General considerations

Unless stated otherwise, all solvents and commercially available reagents were used as purchased. Deuterated solvents were obtained from *Cambridge Isotope Laboratories*. Molecular sieves were activated thermally prior use at 250 °C for several days. Dichloromethane used for the polymerization reactions was collected from an M. Braun MB-SPS 800 solvent purification, degassed by bubbling nitrogen for 30 min through the solvent and stored over 4 Å molecular sieves. All reactions were carried out in an *M-Braun* nitrogen-filled Glovebox. Grubb's 3rd generation catalyst (**GIII**), **M1**, **M2** and **M3** were prepared following reported protocols.[1–3]

Nuclear **M**agnetic **R**esonance (NMR) spectra were recorded either on a Varian *VNMR-S-400* equipped with a *OneNMR* probe or a Jeol *JNM-ECZL G 400* equipped with a *ROYALPROBE HFX* probe. Resonances were referenced to residual solvent peaks (^{1}H: δ 7.26 ppm, ^{13}C{^{1}H}: δ 77.16 ppm for $CDCl_3$, ^{1}H: δ 2.50 ppm, ^{13}C{^{1}H}: δ 39.52 ppm for DMSO). Chemical shifts (δ) are given in ppm and coupling constants (*J*) are quoted in hertz (Hz). Resonances are described by their multiplicity in terms of s (singlet), d (doublet), t (triplet), q (quartet), br (broad singlet) and m (multiplet of higher order) or combinations thereof. ^{1}H, ^{13}C{^{1}H}, ^{13}C{^{1}H}-**A**ttached-**P**roton-**T**est (^{13}C{^{1}H}-APT), **D**iffusion **O**rdered **S**pectroscop**y** (DOSY), **H**eteronuclear **S**ingle **Q**uantum **C**oherence (HSQC), Homonuclear **Co**rrelation **S**pectroscop**y (**COSY), **G**radient-COSY (gCOSY) with an applied **D**ouble **Q**uantum **F**ilter (DQF-gCOSY), **H**eteronuclear **M**ultiple **B**ond **C**orrelation (HMBC) and **N**uclear **O**verhauser **E**ffect **S**pectroscopy (NOESY) spectra were processed through Whittaker-Henderson baseline and arbitrary phase correction, as well as visual apodization of the f1 and f2 dimension FIDs to give the best signal-to-noise ratio. **J-resolved 2D-^{1}H–^{1}H** spectra were processed through applying J-resolved symmetrization and non-local-means-denoise algorithms integrated in *MestReNova* Version 14.2.1. The coupling constants *J* are determined as shown in Equation 1 below.

$$J = \frac{\Delta f_1\,[ppm] \times 400\,MHz}{10^6} \qquad \text{(Eq.1)}$$

Attenuated **T**otal **R**eflection **I**nfra**r**ed spectroscopy (ATR-IR) spectra were recorded on a PerkinElmer UATR Two and processed using PerkinElmer Spectrum IR (version 10.6.2). Prior to each measurement a background spectrum consisting of 8 scans was collected and subsequently subtracted from the sample spectrum. Intensities are reported with w – weak (1 – 10 %Transmission), m – middle (10 – 25 %Transmission), s – strong (>25 %Transmission), b – broad and combinations thereof. Assignments are further differentiated by **v** – stretching and **δ** – bending vibration.

Modulated **D**ifferential **S**canning **C**alorimetry (MDSC) measurements were recorded on a TA Instruments Discovery DSC and analyzed within the TA instruments *Trios* software (version 5.1.1). Tzero low mass aluminium pans fitted with a Tzero aluminium lid were loaded with approximately 5 mg of sample. An isothermal annealing step (10 min at 60 °C) prior to data acquisition allowed the polymer chains to erase thermal history. The modulation was set to 1 °C/min and the temperature ramped up from 20 °C to 300 °C in 3 °C/min increments.

Thermo**G**ravimetric **A**nalysis (TGA) measurements were recorded on a TA Instruments TGA Q50 Thermographic Analyzer under nitrogen on a platinum pan and analyzed using TA instruments *Universal Analysis 2000*. Prior to DSC measurements, the samples were dried via an isothermal TGA method until weight was constant for at least 10 min at 120 °C. The thermal degradation was investigated with approximately 5 mg sample loaded onto the pan at a heating rate of 20 °C/min from 20 °C to 500 °C. The loss of mass at around 100 °C of about 1% is observed, due to the inherent hydrophilicity of the polymers.

Gel **P**ermeation **C**hromatography (GPC) measurements were carried out on a variable setup by Shimadzu (SCI-40 controller module, DGU-203 degassing unit, SIL-40 autosampler, CTO-40C column oven, RID-20A refractive index detector) equipped with an Agilent Technologies *PLgel 5µm Guard 50 x 7.5 mm* guard column followed by two Agilent Technologies *PLgel 5µm MIXED-D 300 x 7.7 mm* columns in series. *N*,*N*-dimethylformamide (peptide synthesis grade) was used as the eluent without any additions of lithium chloride. The measurements were run at a flow rate of 1 mL/min at 65 °C at pressures between 85-90 bar and analyzed with Shimadzu *LabSolutions GPC* software. For calibration, a combined calibration set of polystyrene was used consisting of peak molecular weight (M_p) defined standards of 4k, 7k, 13k, 20k, 50k, 100k, 200k and 300k g/mol, which was measured prior to each sample measurement. It is important to note, that the chemical structure of polystyrene differs from the investigated polymers, thus making the reported values for weight averaged molecular weight (M_w) and number averaged molecular weight (M_n) inherently erroneous. The samples were dissolved (2 mg/mL) in the eluent, taken up into a glass syringe and passed through a 0.45 µm syringe filter into the GPC vials.

Powder **X**-**R**ay **D**iffraction (pXRD) patterns were measured with a Bruker-AXS D2 Phaser X-ray diffractometer using Co-Kα1,2 radiation (λ = 1.790 Å). Diffraction patterns were collected between 0- 50° 2Θ with an increment of 0.03° 2Θ ·s^{-1}.

Monomer synthesis[2,3]

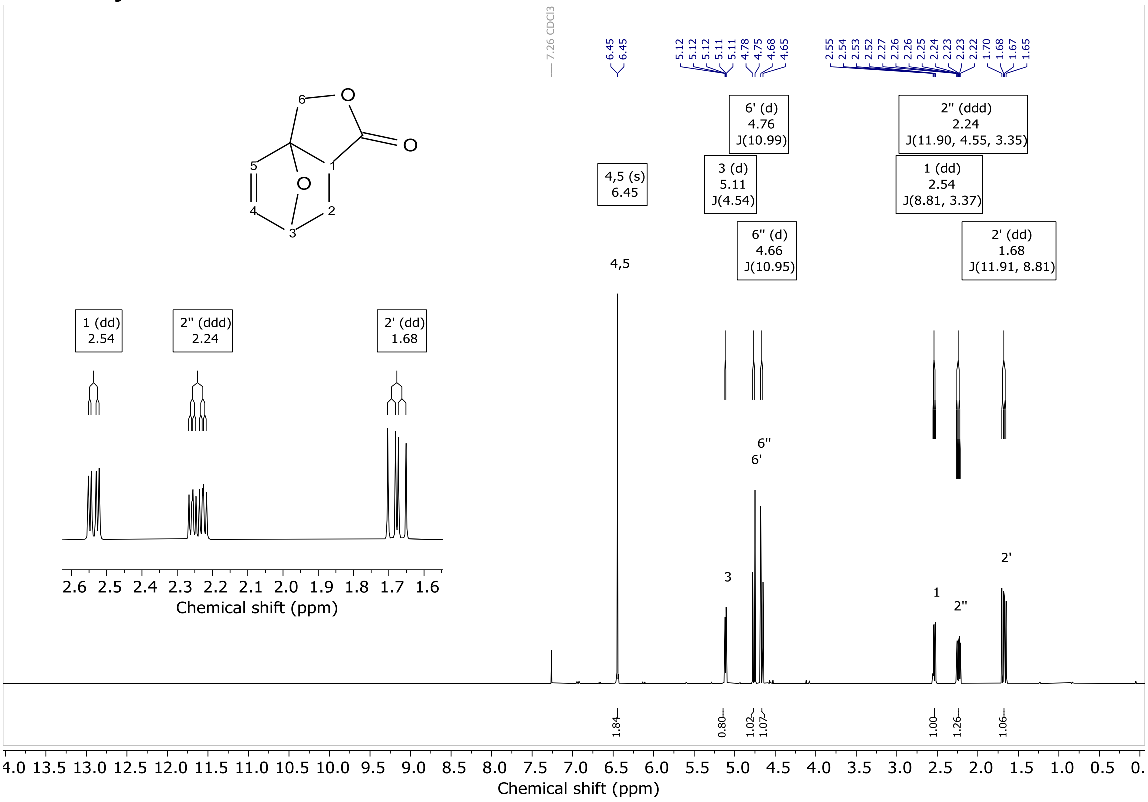

Figure S1: ^{1}H NMR spectrum of **M1** (400 MHz, $CDCl_3$).

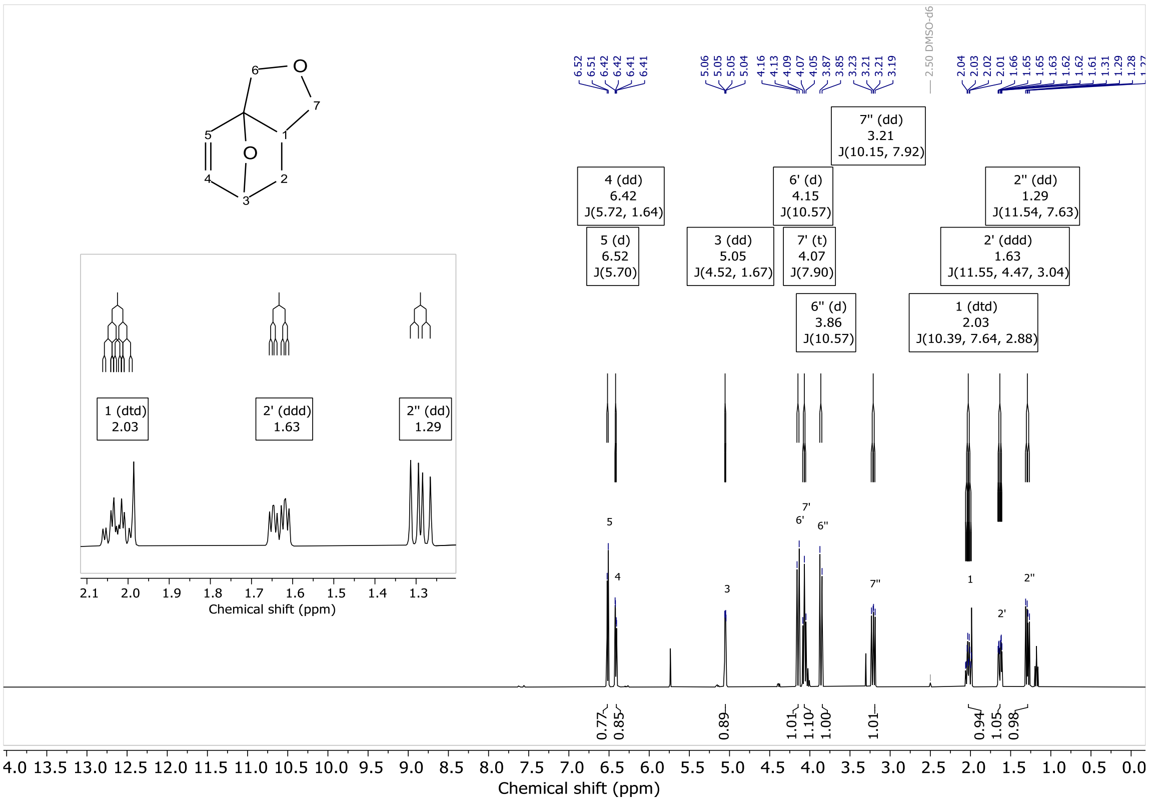

Figure S2: ^{1}H NMR spectrum of **M2** (400 MHz, DMSO-D6).

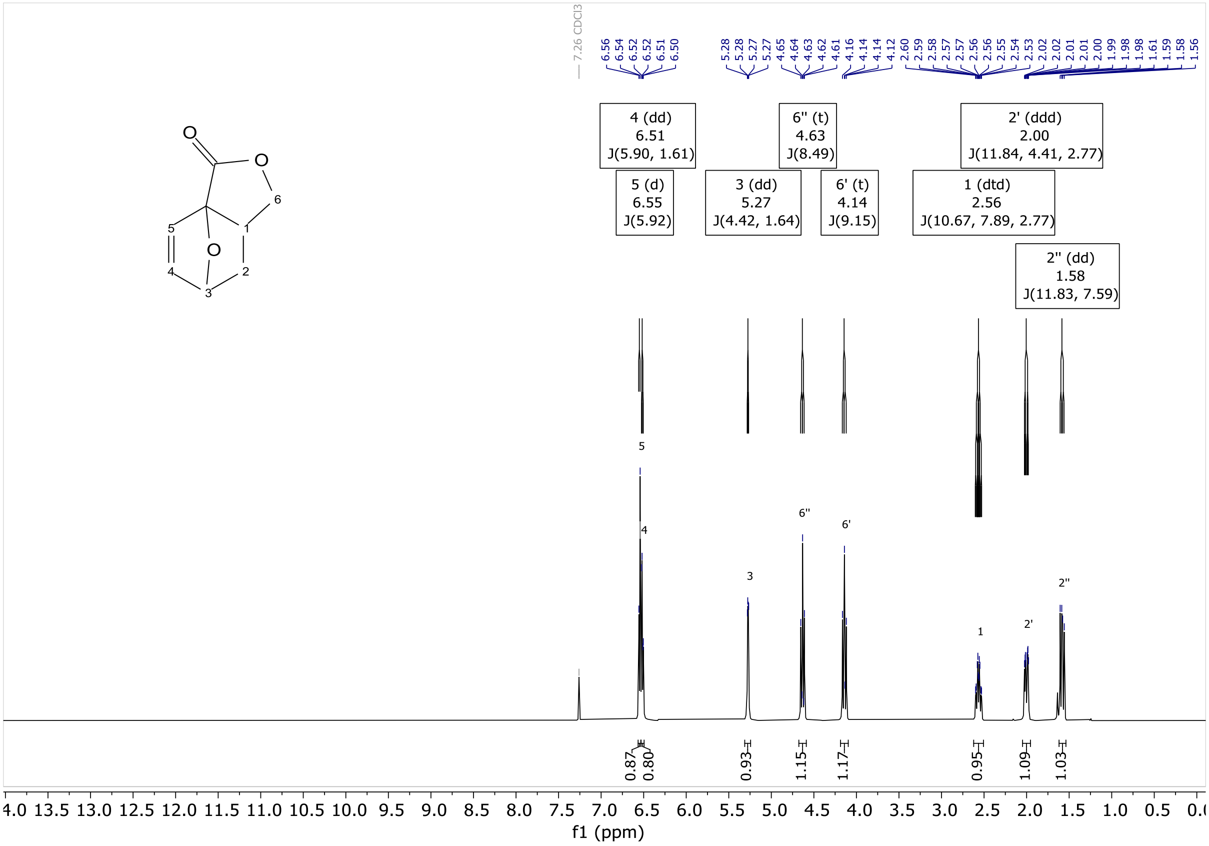

Figure S3: ^{1}H NMR spectrum of **M3** (400 MHz, CDCl3).

Polymer synthesis

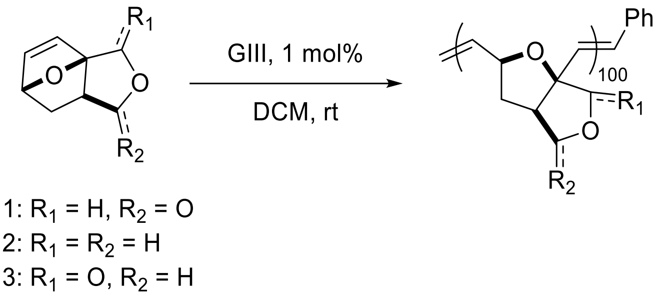

M1: R_1 = H, R_2 = O
M2: R_1 = R_2 = H
M3: R_1 = O, R_2 = H

In a glovebox, monomer (**M1** and **M3** 164.3 mg, **M2** 152.2 mg, final concentration 0.068 M) was dissolved in 8 mL DCM. **GIII** (9.6 mg) was dissolved in 8 mL DCM and the priorly prepared monomer solution was added (stirring rate 600 s^{-1}). The reaction was quenched with vinyl ethyl ether (2 mL) after definite time increments, 1 min for **pM1**, 4 min for **pM2** and 60 min for **pM3** respectively. Subsequently, the reaction vessel was taken outside the glovebox, and all volatiles were removed under reduced pressure. To the crude material, 2 mL of 1,1,1,3,3,3-hexafluoroisopropanol (for **pM1** and **pM3**) or DCM (for **pM2**) was added. The polymer was dissolved with the aid of a vortex mixer set to 2000 rpm, and then precipitated by slowly passing the solution through a syringe filter into 20 mL of MeOH (for **pM1** and **pM3**) or petroleum ether (for **pM2**). After filtration the polymers were obtained as off-white powders with near quantitative yields (**pM1** 96%, **pM2** 92%, **pM3** 95%).

It must be noted that in the collected spectral datasets of **pM1** and **pM3**, 1,1,1,3,3,3-hexafluoroisopropanol was present as an impurity (δ 8.03 (d, *J* = 7.5 Hz, 1H), 5.14 (hept, *J* = 6.9 Hz, 1H) ppm), which despite our best efforts could not be removed quantitatively from the prepared samples. This is denoted with (*) in the corresponding spectra.

pM1

^{1}H NMR (400 MHz, DMSO-D6) δ 6.34 – 5.34 (m), 5.21 – 4.83 (m), 4.78 – 4.58 (m), 4.49 – 4.14 (m), 3.34 (m), 2.42 – 2.11 (m), 2.08 – 1.66 (m) ppm.

^{13}C{^{1}H} NMR (101 MHz, DMSO-D6) δ 137.5 – 128.0 (m), 90.0 – 85.1 (m), 80.3 – 78.2 (m), 77.0 – 74.5 (m), 52.9 – 49.1 (m), 36.9 – 33.7 ppm.

1/λ (cm^{-1}): 2959 (wb, ν(**H–C**=C)), 1770 (s, ν(C=O)), 1461 (w, ν(CH_2)), 1372 (w, δ(C-H)), 1288 (w, ν(C–O)), 1262 (w, ν(O=**C–O**)), 1218 (w, ν(C=C–**C–O**)), 1171 (m, ν(C–O)), 1145 (w, ν(C–O)), 1102 (w, ν(C–O)), 1074 (w, δ(C–O)), 1020 (s, δ(O=**C–O**R)), 985 (m, δ(C=C)), 895 (m, δ(C=C)), 860 (w, δ(C–C)), 814 (w, δ(C–C)), 735 (w, δ(C=C)), 685 (w, δ(C=C)).

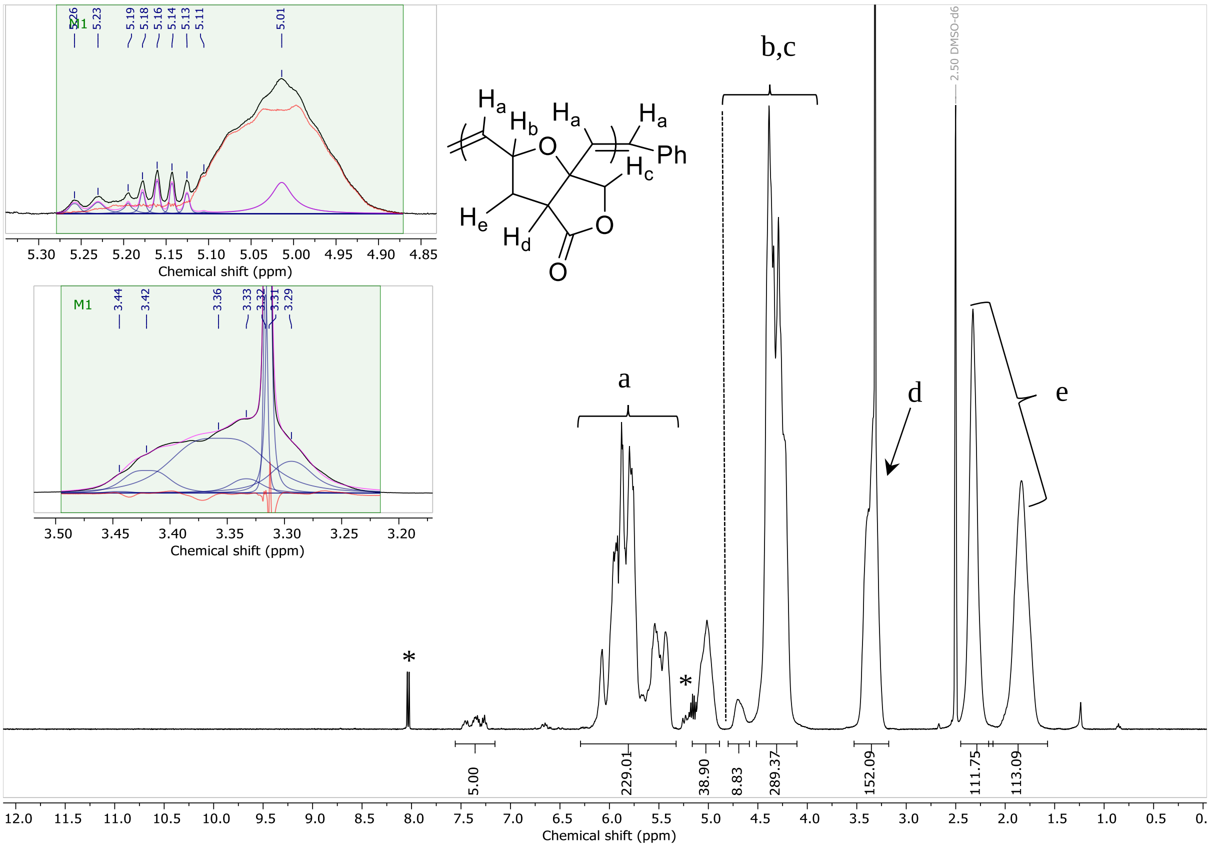

Figure S4: ^{1}H NMR spectrum with enhanced regions highlighting the signal deconvolution of **pM1** (400 MHz, DMSO-D6). Resonance signals at 7.5 ppm corresponds to the phenyl end-group of the polymer. The enhanced region depicts the applied Global Spectral Deconvolution (GSD), where the red line indicates residual resonances, corresponding to the overlapped signals.

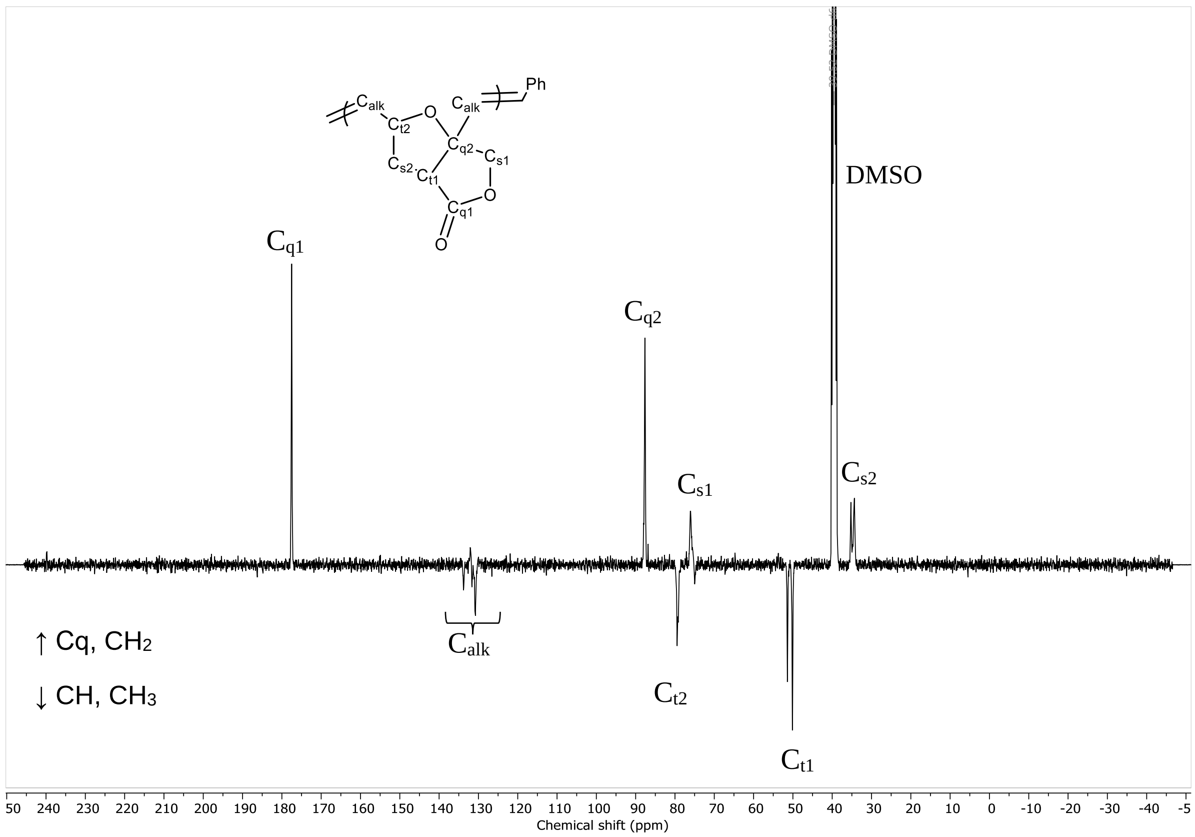

Figure S5: APT-^{13}C{^{1}H} NMR spectrum of **pM1** (101 MHz, DMSO-D6). C_s = secondary carbon, C_t = tertiary carbon, C_q = quartenary carbon, C_{alk} = alkene carbon.

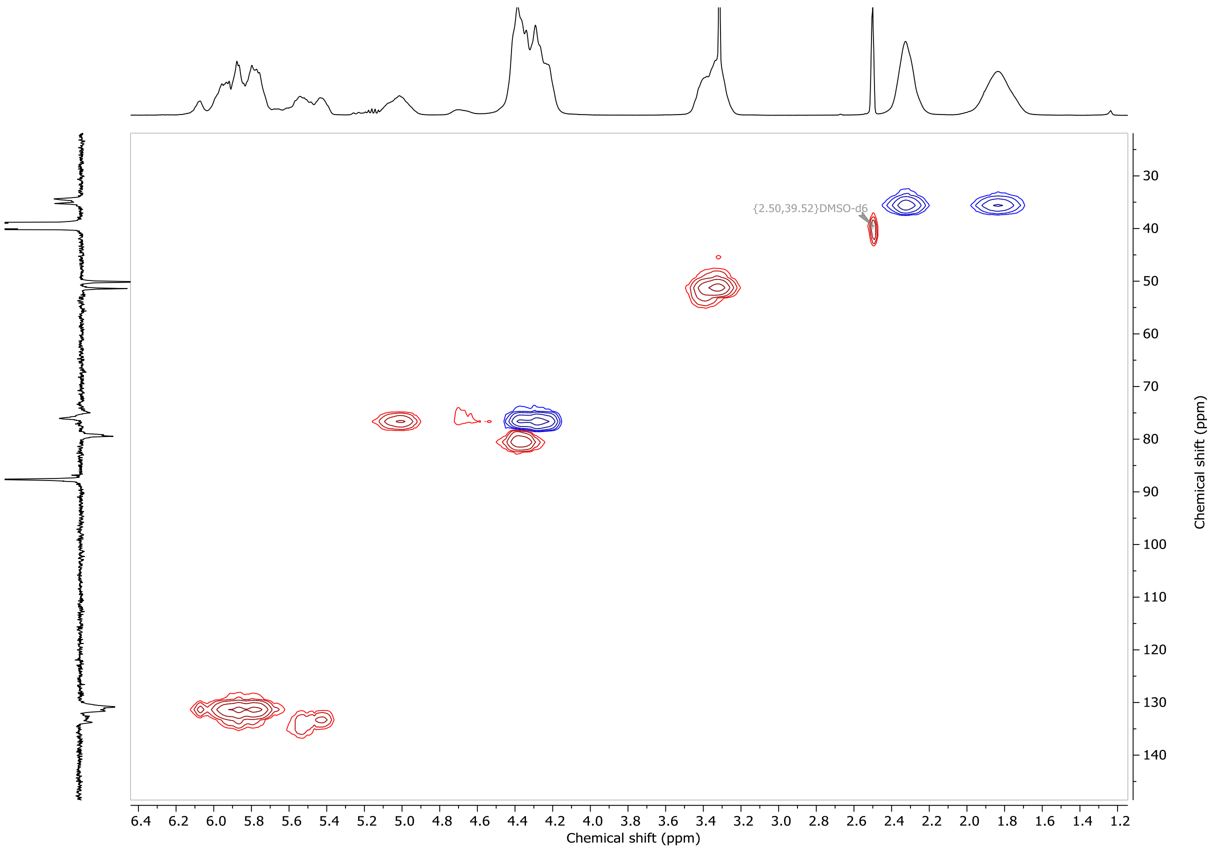

Figure S6: HSQC NMR spectrum of **pM1** (DMSO-D6).

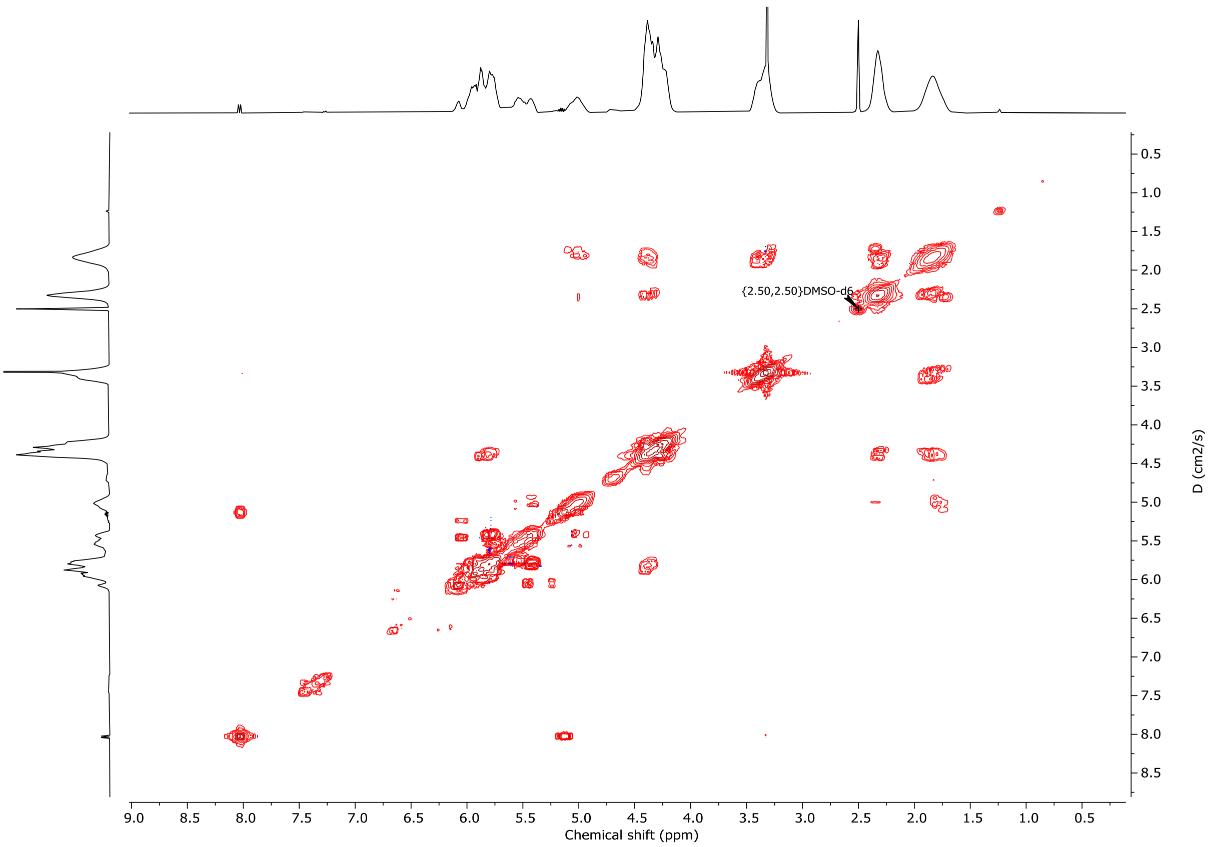

Figure S7: gCOSY NMR spectrum of **pM1** (DMSO-D6).

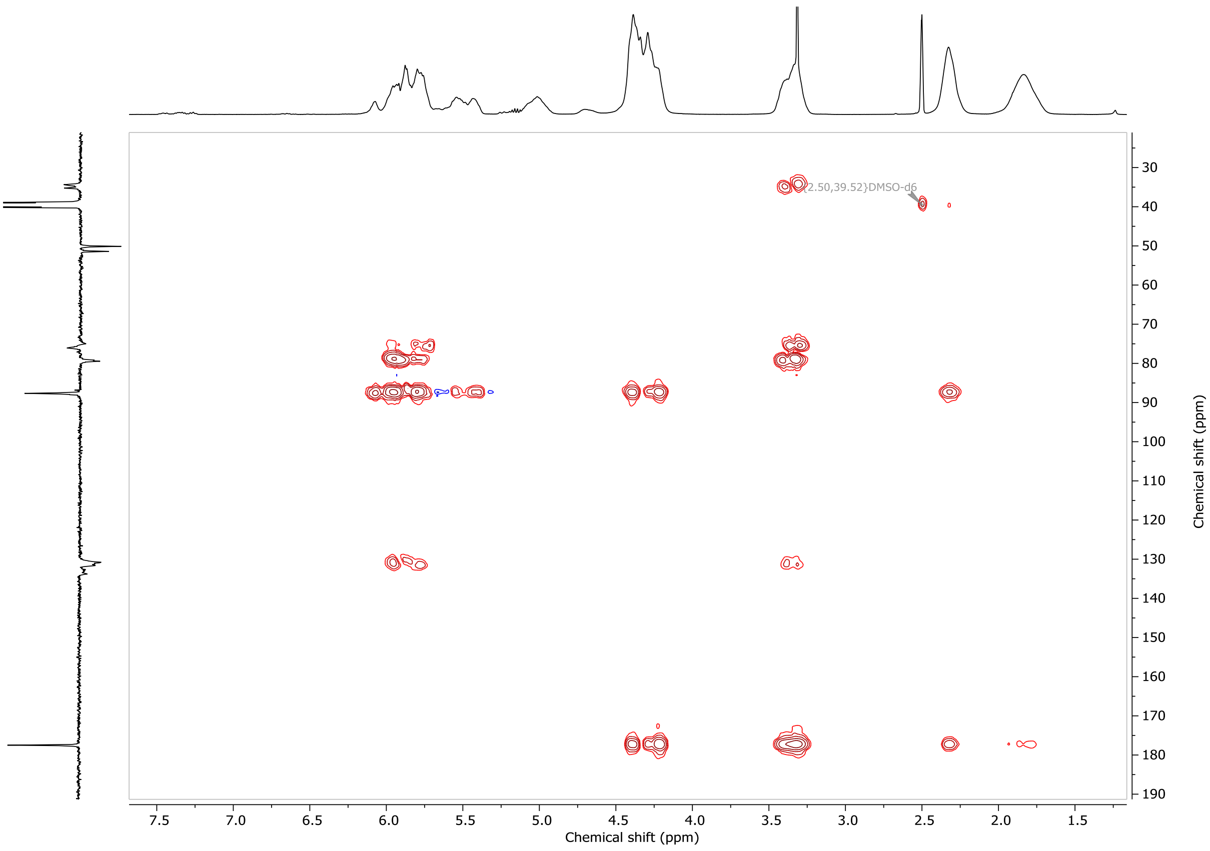

Figure S8: HMBC NMR spectrum spectrum of **pM1** (DMSO-D6).

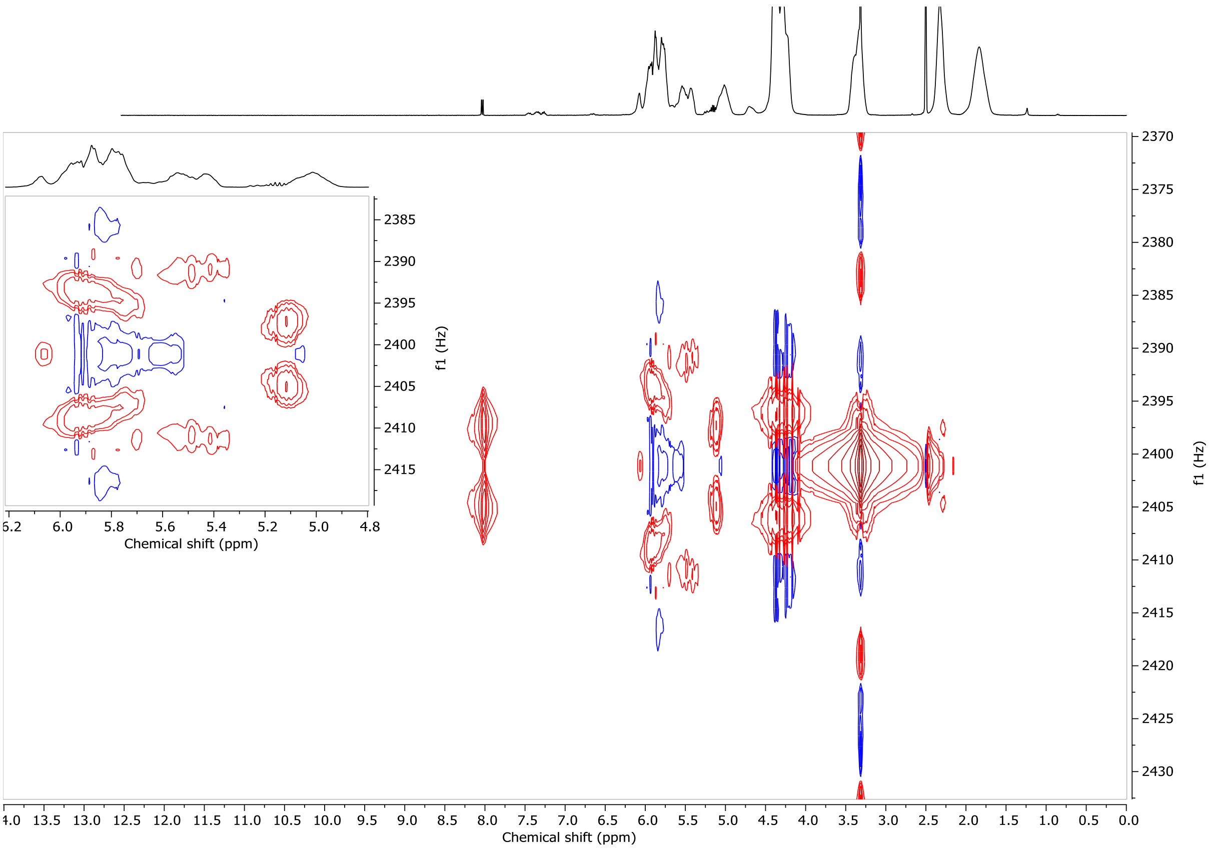

Figure S9: J-resolved NMR spectrum of **pM1** with enhanced region of interest (DMSO-D6).

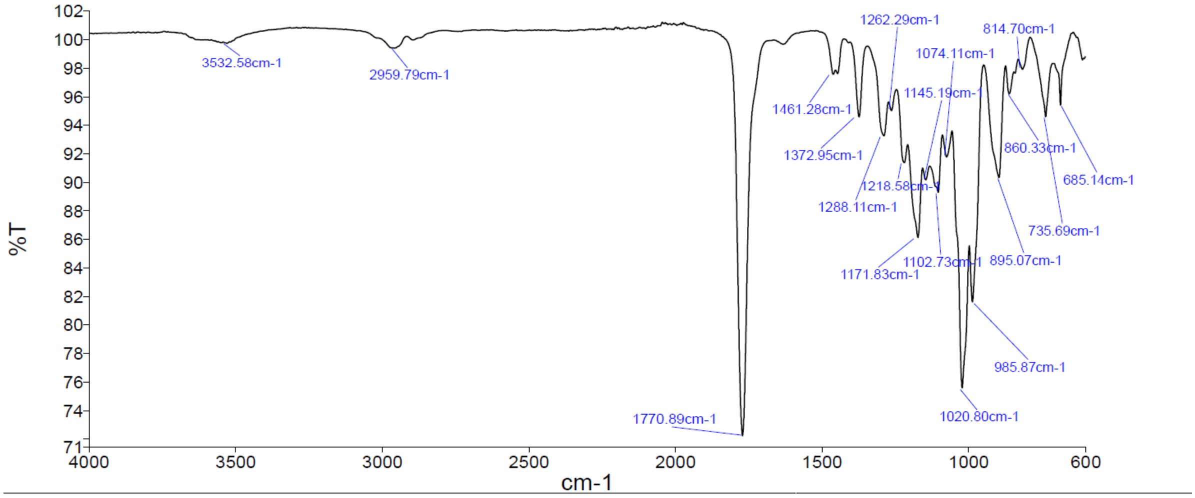

Figure S10: ATR-IR spectrum of **pM1**.

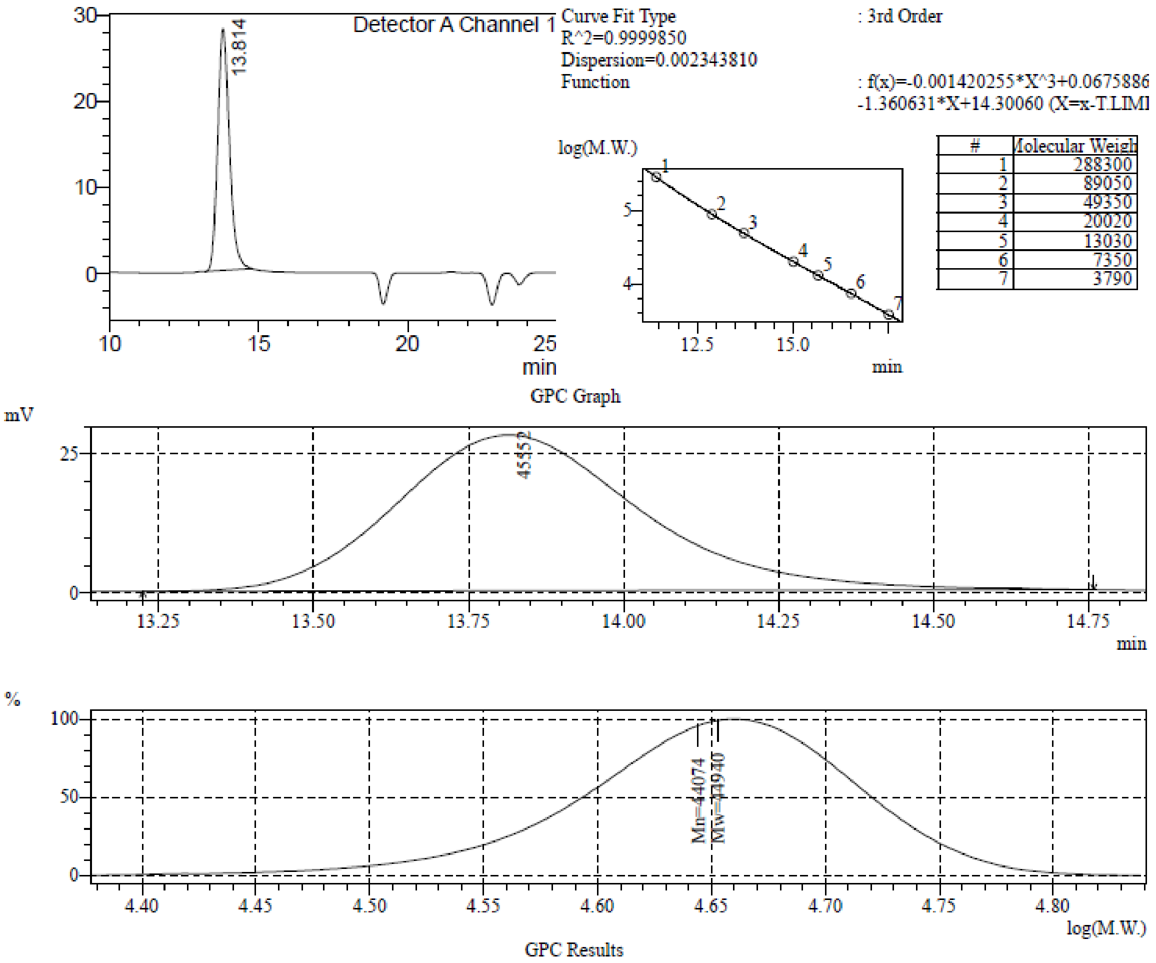

Peak#:1 (Detector A Channel 1)

[Peak Information]

Title	Time(min)	Molecular Weig	Height
Start	13.225	69567	279
Top	13.814	45552	28032
End	14.758	23764	624

Area : 768082

[Average Molecular Weight]

Number Average Molecular Weight(Mn)	44074
Weight Average Molecular Weight(Mw)	44940
Mw/Mn	1.01963

Figure S11: GPC chromatogram of **pM1** (catalyst to monomer ratio: 1:100) with utilized calibration.

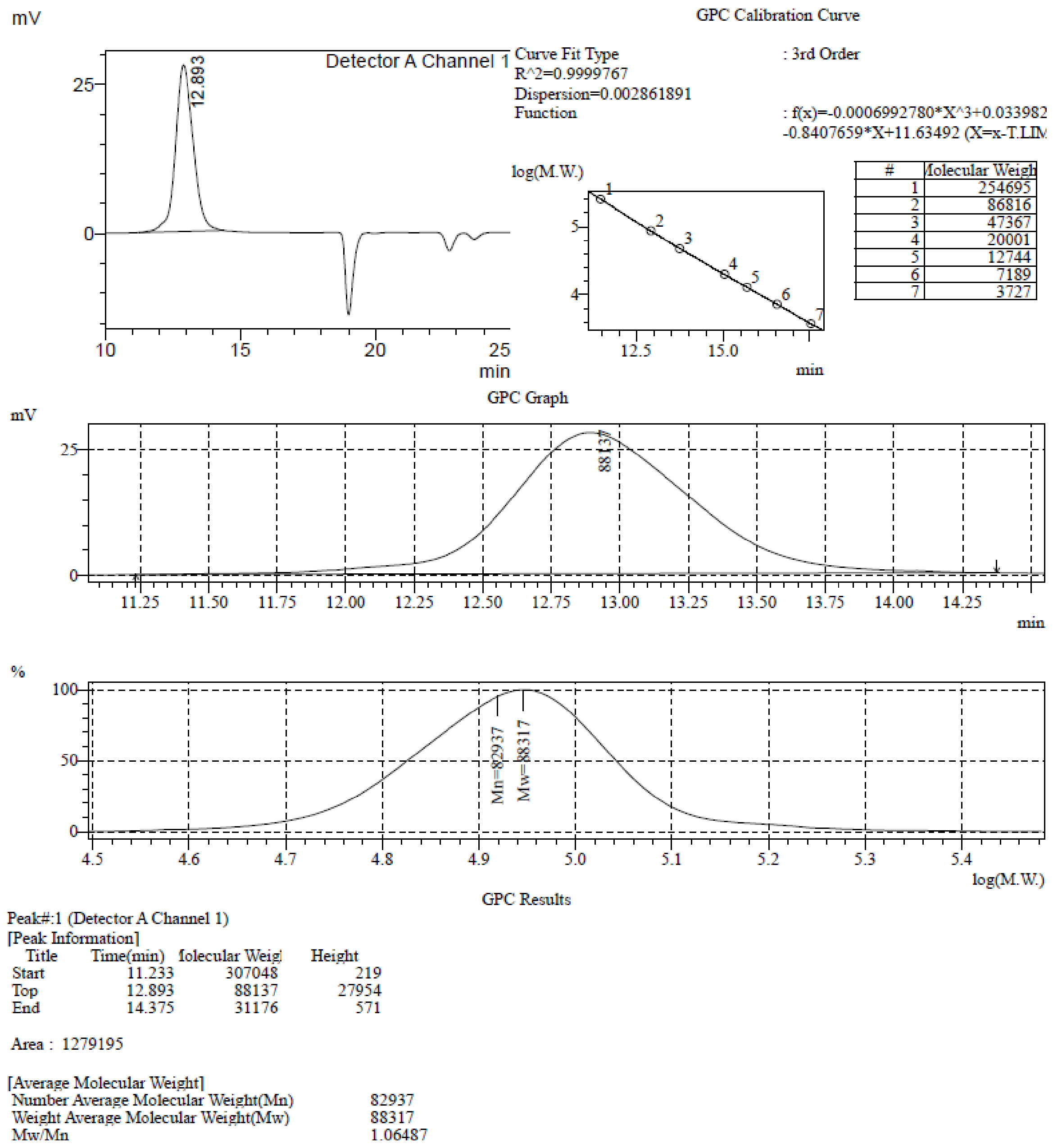

Figure S12: GPC chromatogram of **pM1** (catalyst to monomer ratio: 1:200) with utilized calibration.

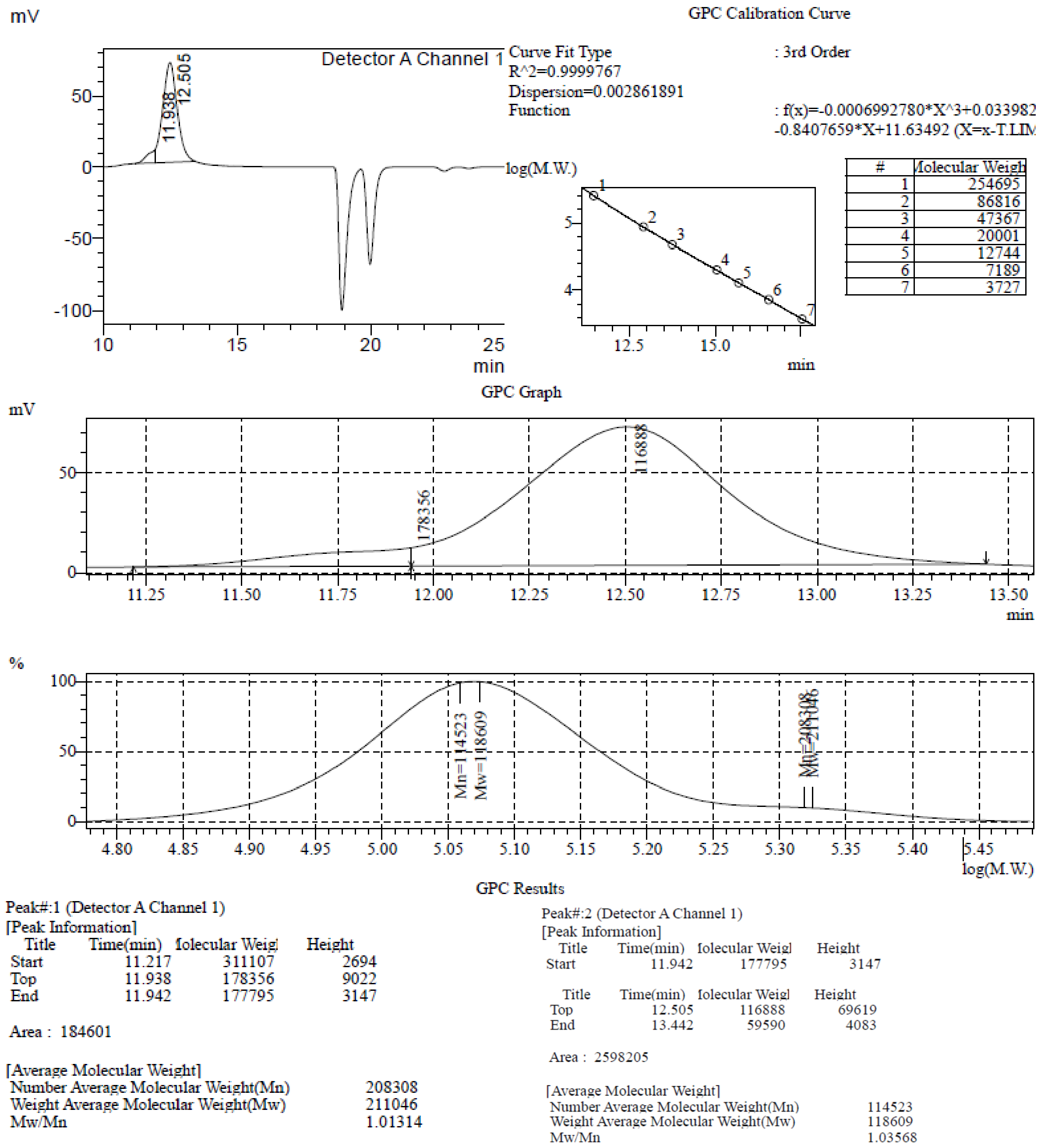

Figure S13: GPC chromatogram of **pM1** (catalyst to monomer ratio: 1:300) with utilized calibration.

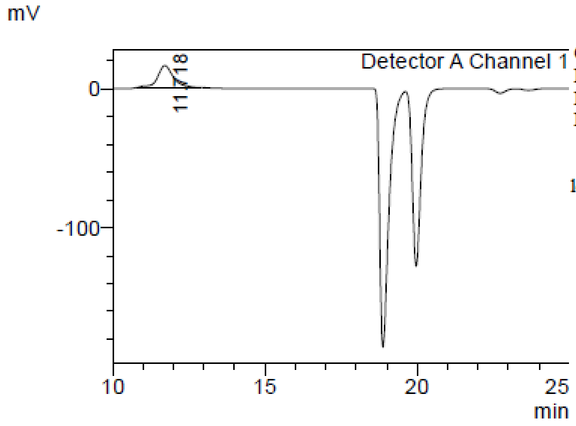

GPC Calibration Curve

Curve Fit Type : 3rd Order
R^2=0.9999767
Dispersion=0.002861891
Function : f(x)=-0.0006992780*X^3+0.033982
-0.8407659*X+11.63492 (X=x-T.LIM

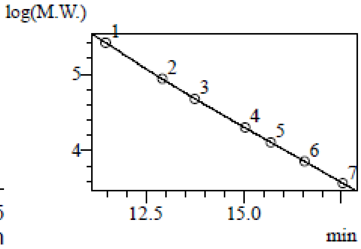

#	Iolecular Weigh
1	254695
2	86816
3	47367
4	20001
5	12744
6	7189
7	3727

GPC Graph

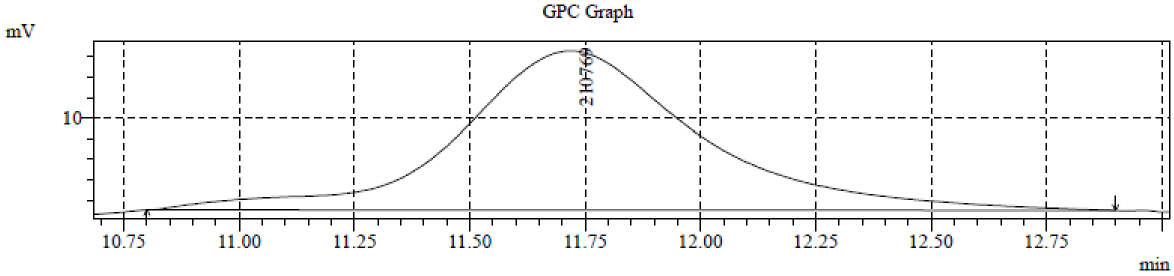

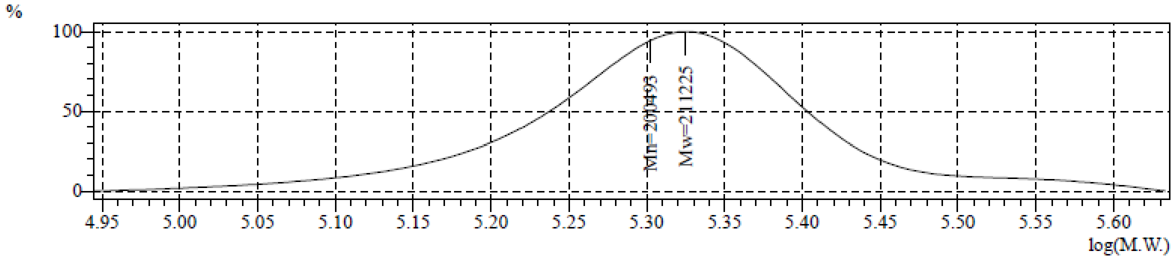

GPC Results

Peak#:1 (Detector A Channel 1)
[Peak Information]

Title	Time(min)	Iolecular Weig	Height
Start	10.800	433939	1052
Top	11.718	210769	15533
End	12.900	87673	995

Area : 559283

[Average Molecular Weight]

Number Average Molecular Weight(Mn)	200493
Weight Average Molecular Weight(Mw)	211225
Mw/Mn	1.05352

Figure S14: GPC chromatogram of **pM1** (catalyst to monomer ratio: 1:500) with utilized calibration.

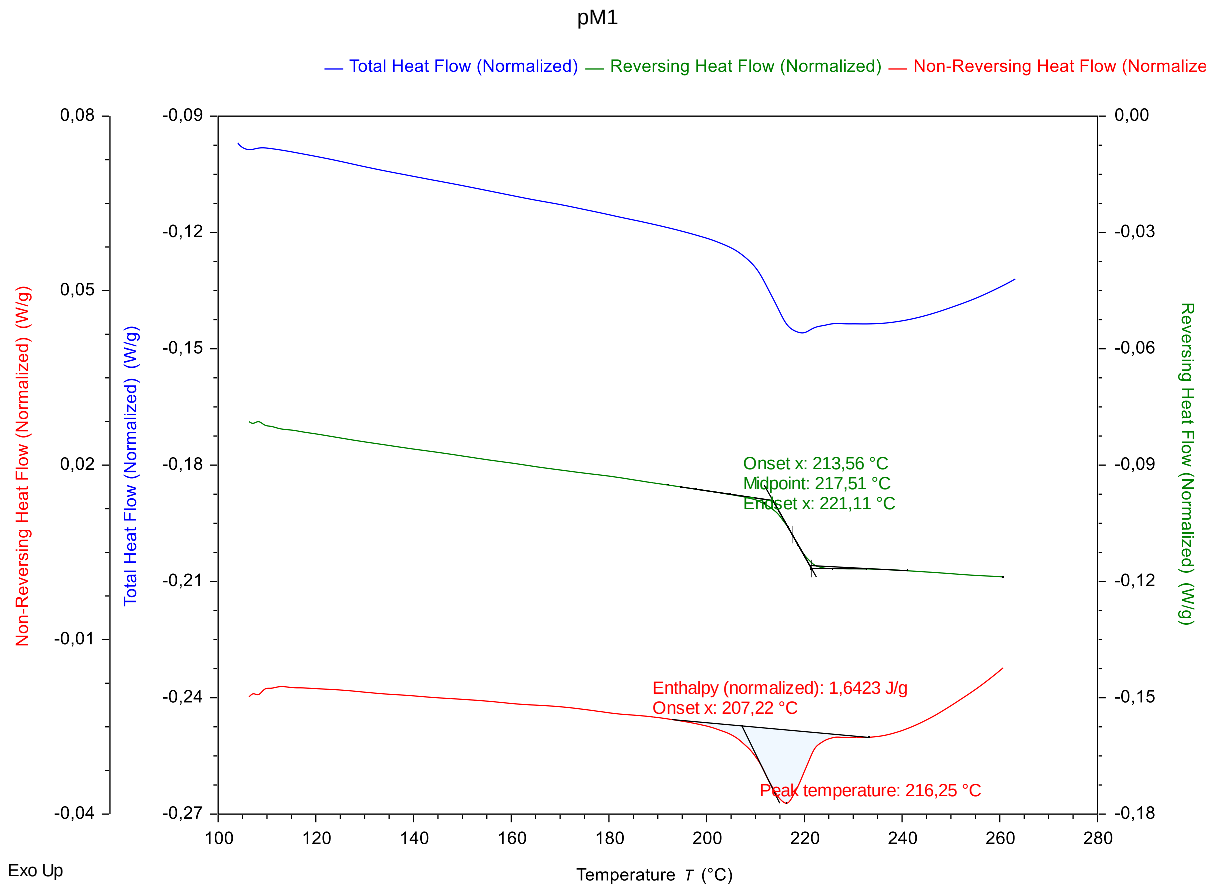

Figure S15: MDSC thermogram of **pM1**.

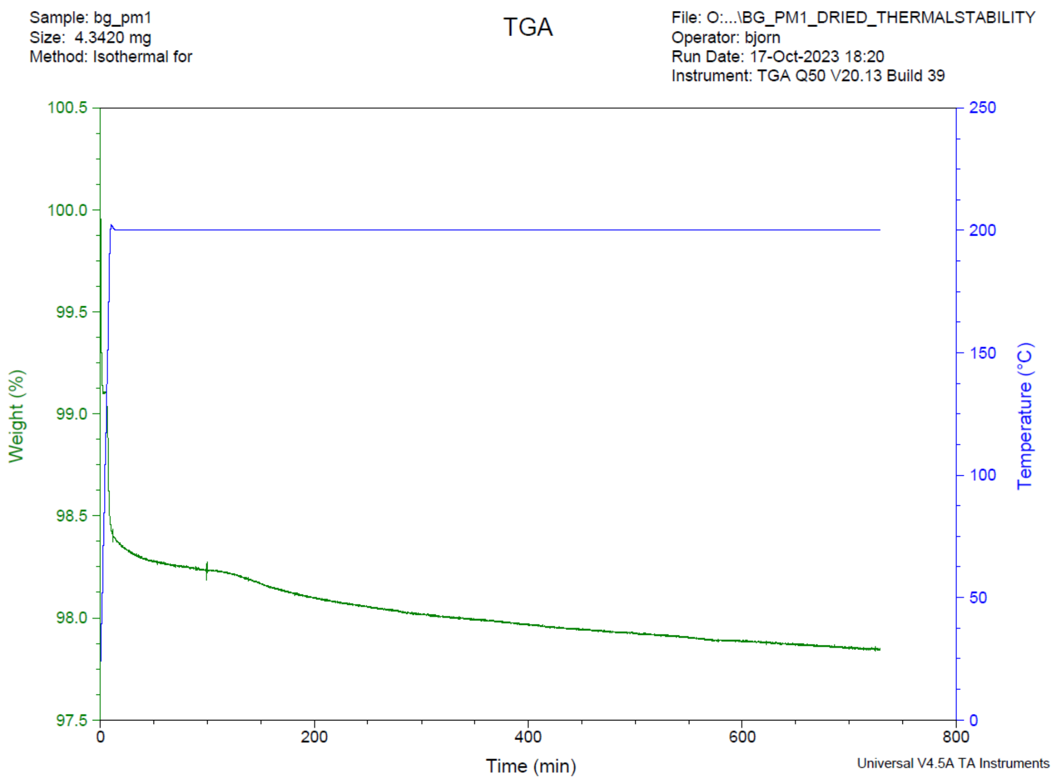

Figure S16: TGA thermogram of **pM1** for evaluating thermal stability (200°C for 12 h).

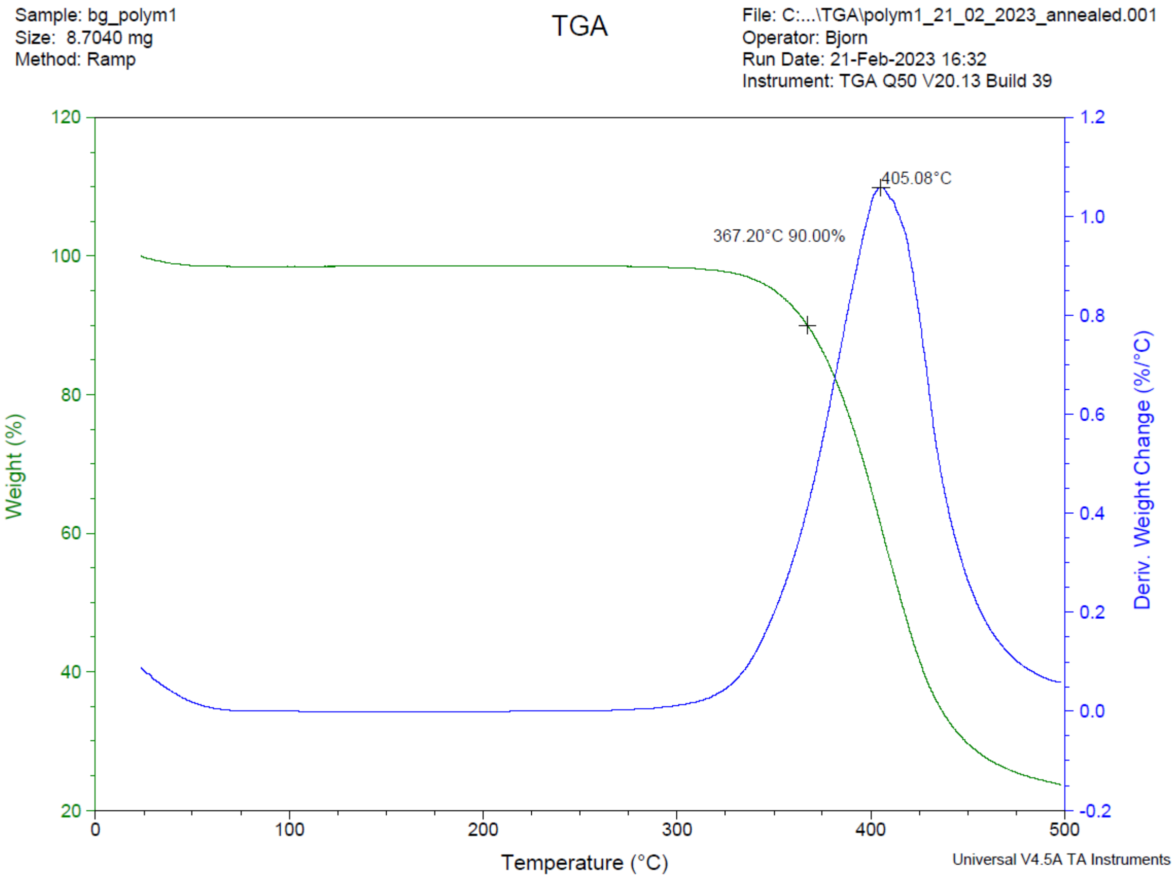

Figure S17: TGA thermogram for **pM1**

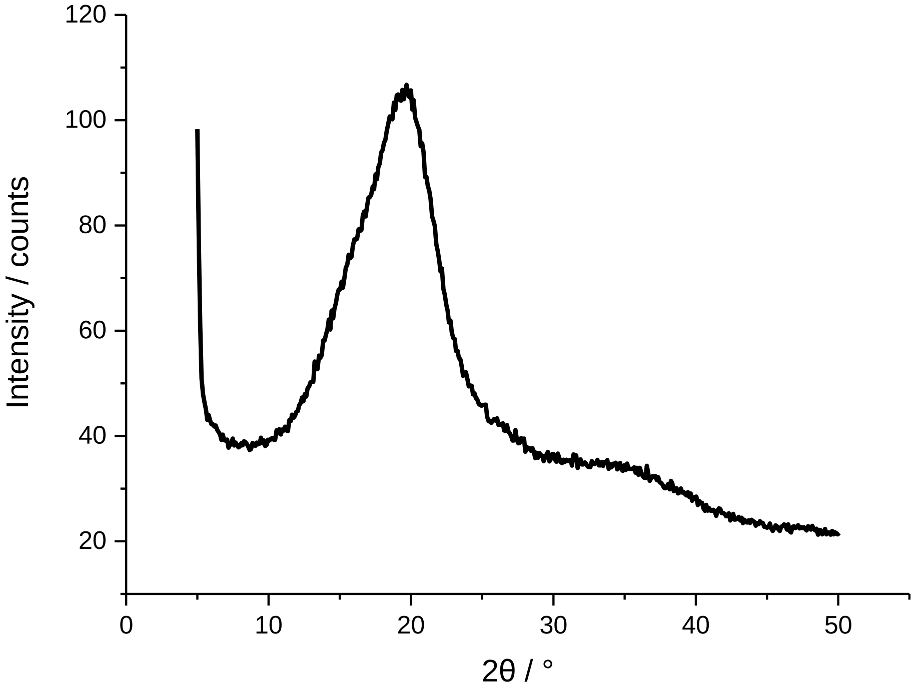

Figure S18: pXRD data for **pM1**.

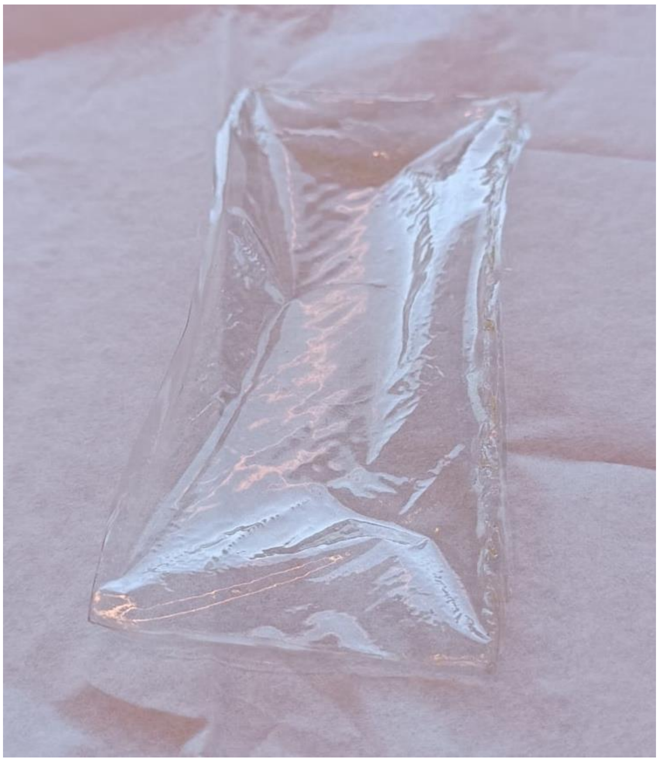

Figure S19: Transparent film of **pM1** made by solventcasting (HFIP).

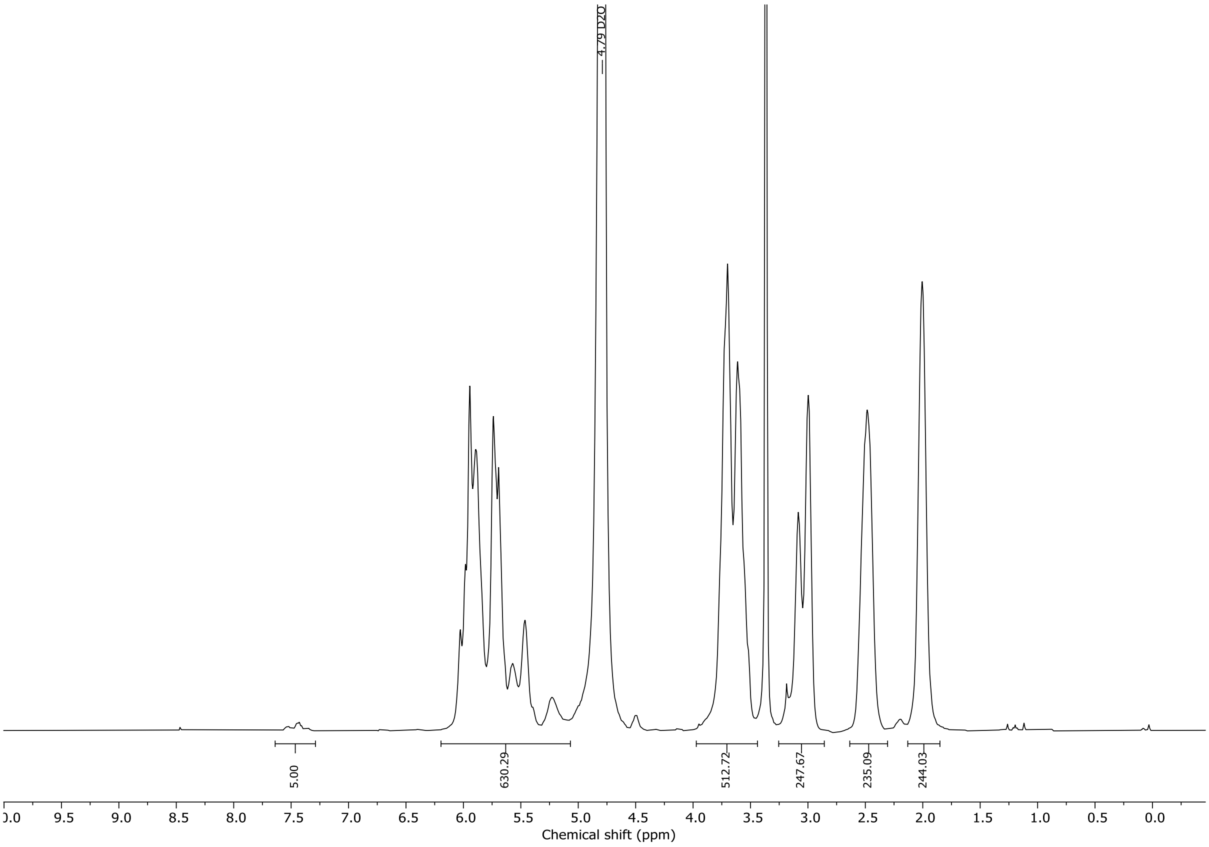

Figure S20: **pM1** (250 eq. **M1**) after being treated with 2 M NaOH, making the polymer soluble in D_2O.

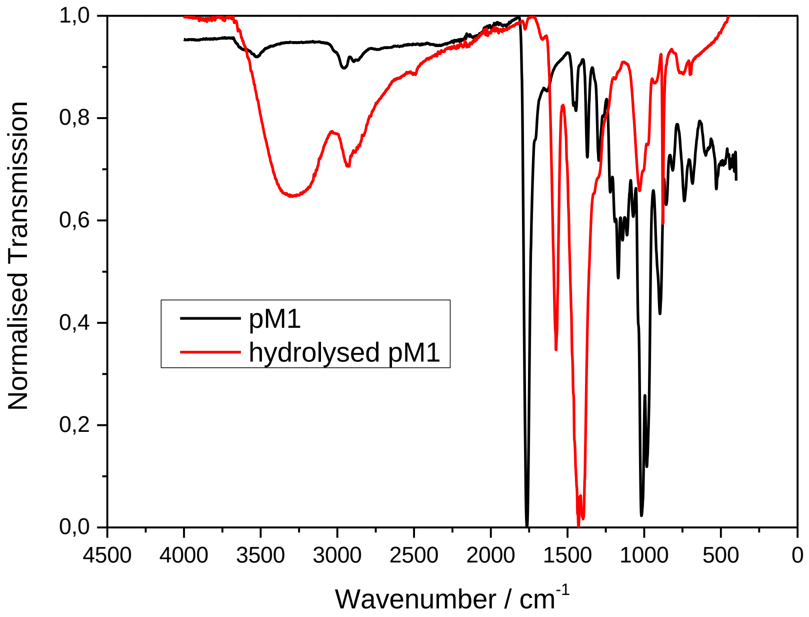

Figure S21: Overlap of IR spectra of **pM1** before and after hydrolysis.

pM2

^{1}H NMR (400 MHz, DMSO-D6) δ 6.10 – 5.23 (m), 5.23 – 4.38 (m), 4.19 – 3.40 (m), 3.03 – 2.53 (m), 2.22 – 1.79 (m), 1.78 – 1.51 (m) ppm.

^{13}C{^{1}H} NMR (101 MHz, DMSO-D6) δ 136.8 – 125.9, 96.2 – 89.5, 80.6 – 79.4, 79.3 – 77.7, 76.6 – 75.0, 74.7 – 73.6, 51.8 – 48.9 ppm.

1/λ (cm^{-1}): 2962 (w, ν(**H–C**=C)), 2851 (w, ν(C–**C–H**)), 1633 (w, ν(C=C_{cis}–C)), 1446 (w, ν(CH_2)), 1363 (w, δ(C-H)), 1331 (w, δ(C-H)), 1261 (w, ν(C=C–**C–O**)), 1190 (w, ν(C–O)), 1096 (m, ν(C–O)), 1046 (m, ν(C–O)), 1006 (s), 971 (m, δ(C=C)), 929 (s), 867 (w, δ(C–C)), 823 (w, δ(C–C)), 711 (w, δ(C=C)).

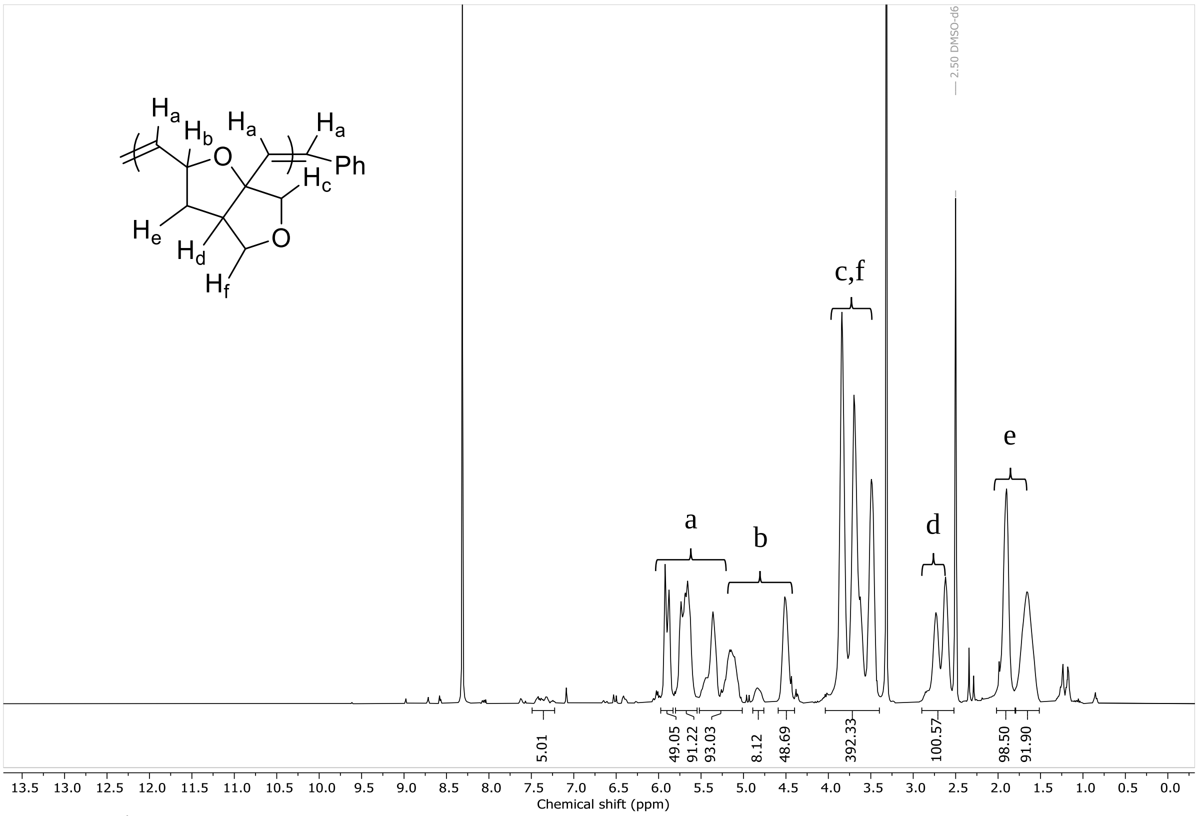

Figure S22: ^{1}H NMR spectrum of **pM2** (400 MHz, DMSO-D6) Resonance signals at 7.5 ppm corresponds to the phenyl end-group of the polymer.

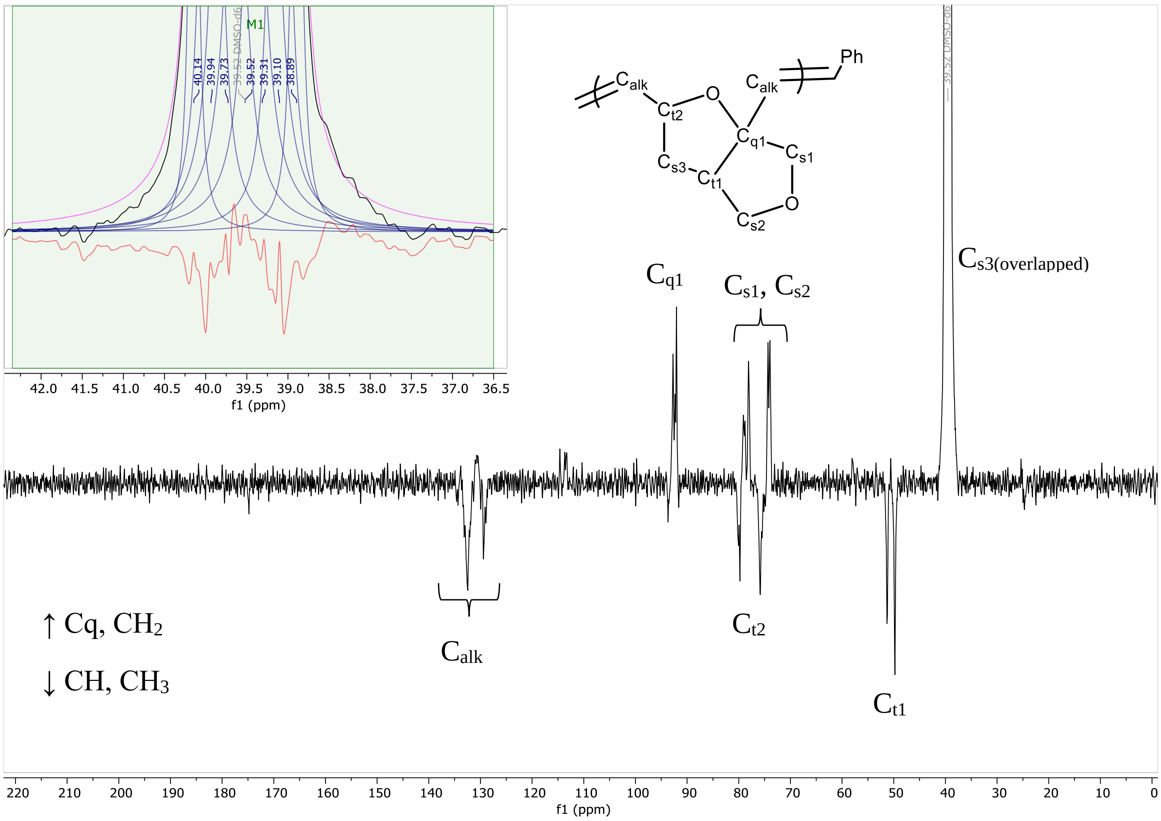

Figure S23: APT-$^{13}C\{^1H\}$ NMR spectrum of **pM2** (101 MHz, DMSO-D6). C_s = secondary carbon, C_t = tertiary carbon, C_q = quartenary carbon, C_{alk} = alkene carbon. The enhanced region depicts the applied Global Spectral Deconvolution (GSD), where the red line indicates residual resonances, corresponding to the overlapped signals.

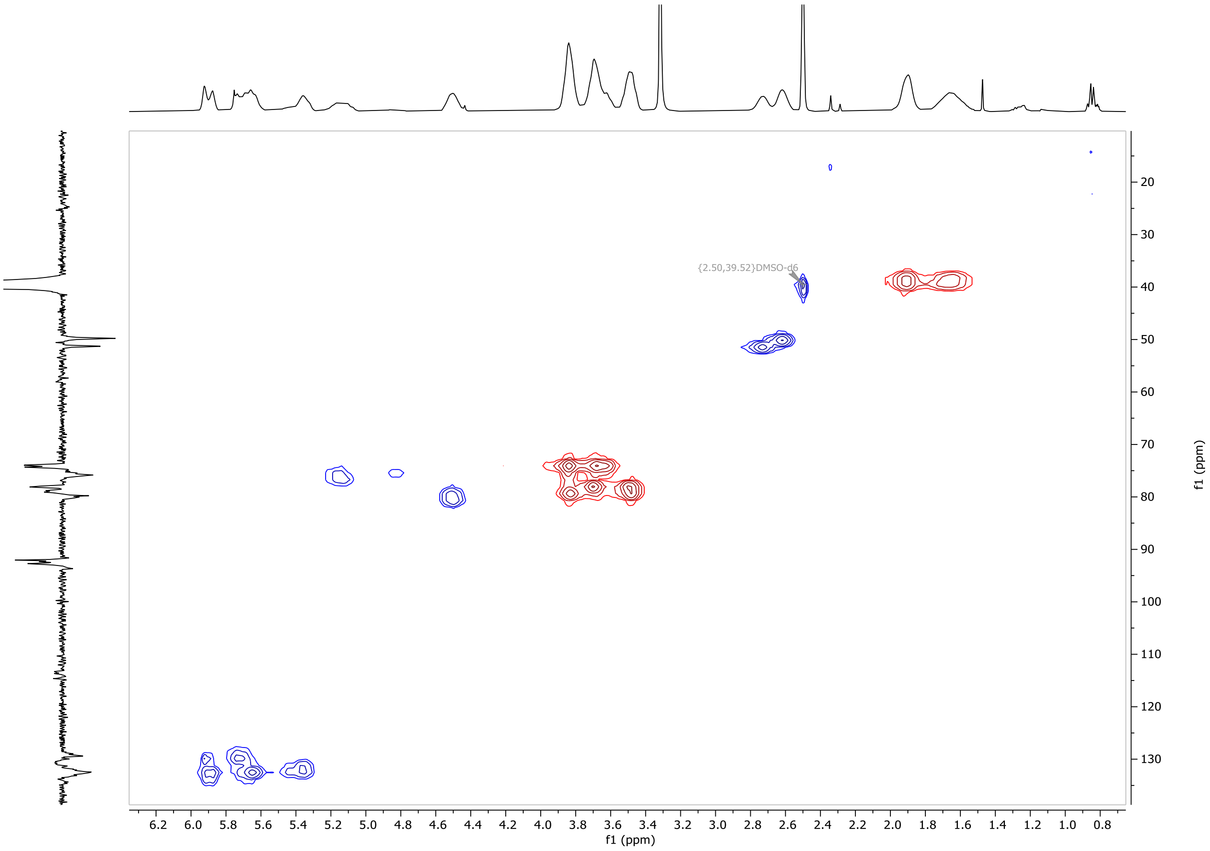

Figure S24: HSQC NMR spectrum of **pM2** (DMSO-D6).

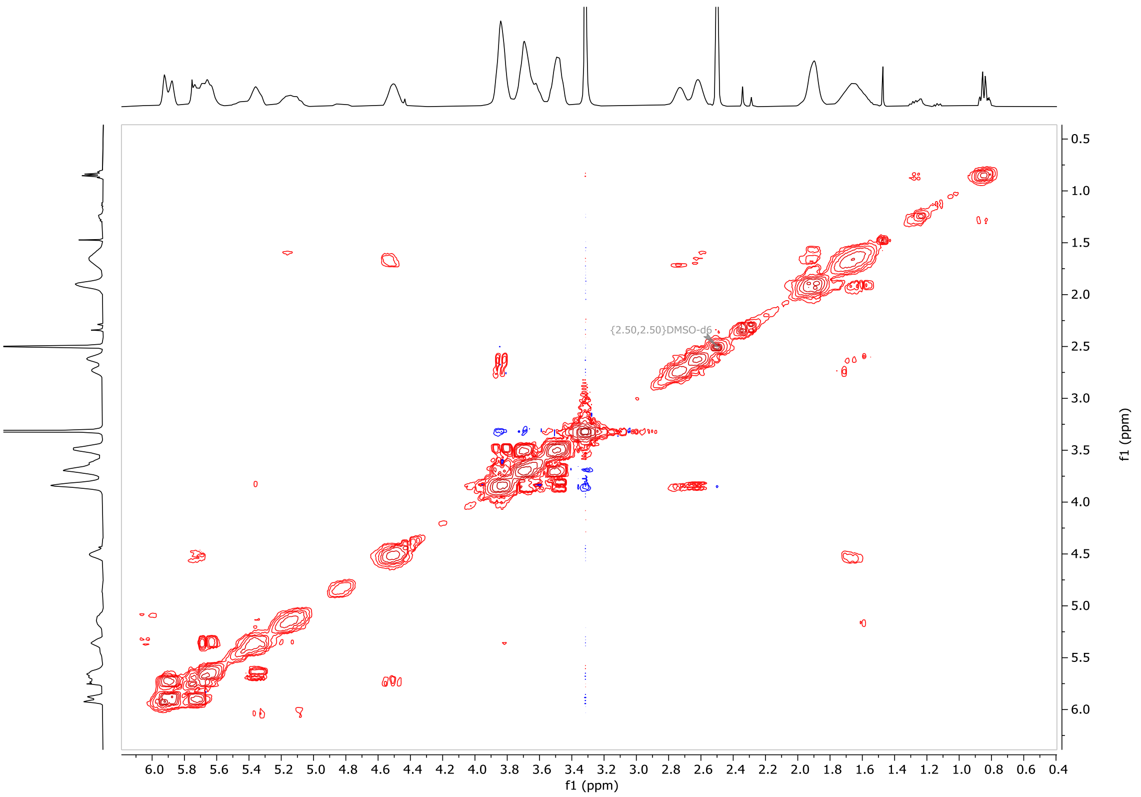

Figure S25: COSY NMR spectrum of **pM2** (DMSO-D6).

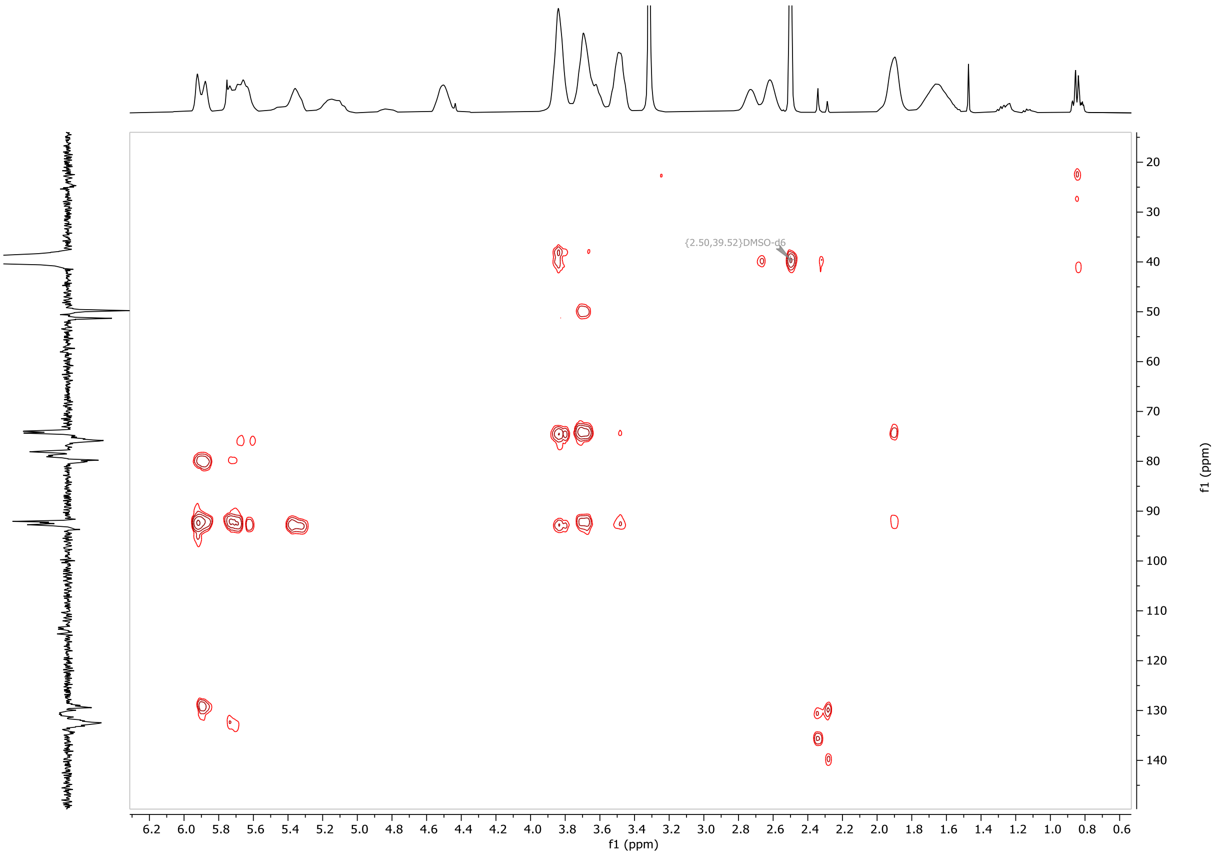

Figure S26: HMBC NMR spectrum of **pM2** (DMSO-D6).

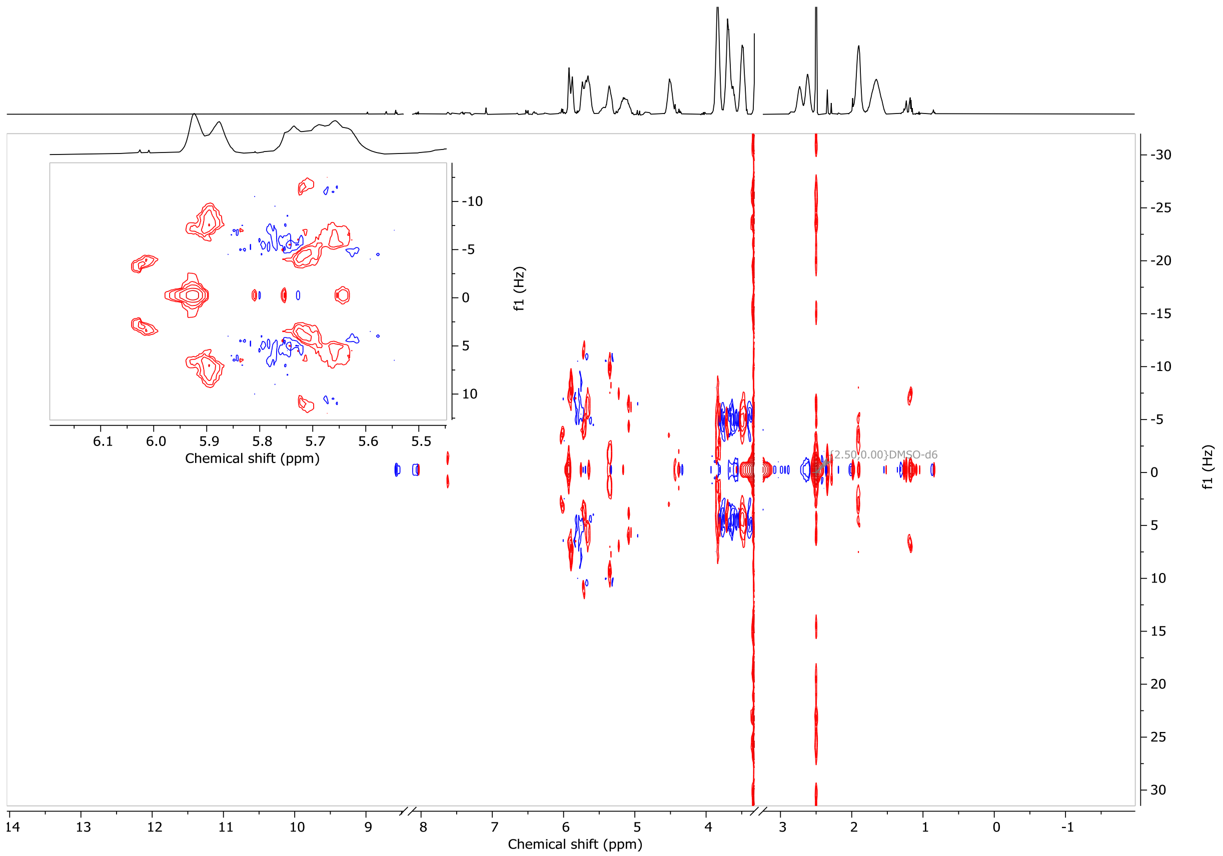

Figure S27: *J*-resolved NMR spectrum of **pM2** with enhanced region of interest (DMSO-D6). Two signals of water and HFIP were removed from the spectrum for clarity. The unedited ^{1}H spectrum can be seen in Figure S20.

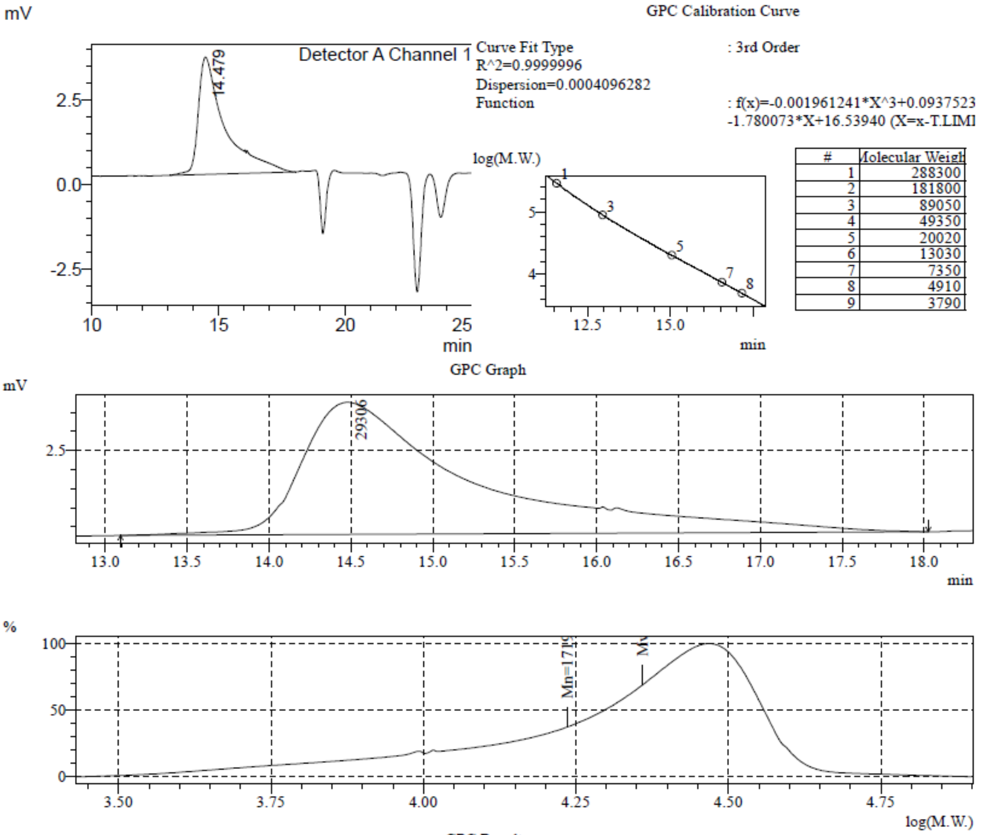

GPC Results

Peak#:1 (Detector A Channel 1)

[Peak Information]

Title	Time(min)	Molecular Weigl	Height
Start	13.092	79984	275
Top	14.479	29306	3460
End	18.025	2680	372

Area : 251710

[Average Molecular Weight]

Number Average Molecular Weight(Mn)	17195
Weight Average Molecular Weight(Mw)	22836
Mw/Mn	1.32807

Figure S28: GPC chromatogram of **pM2** with utilized calibration.

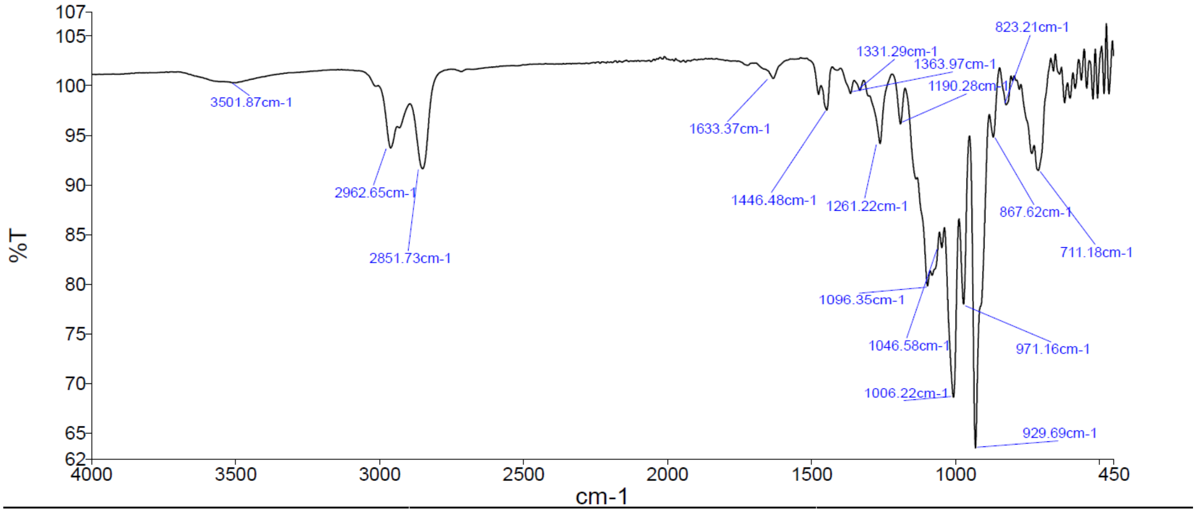

Figure S29: ATR-IR spectrum of **pM2**.

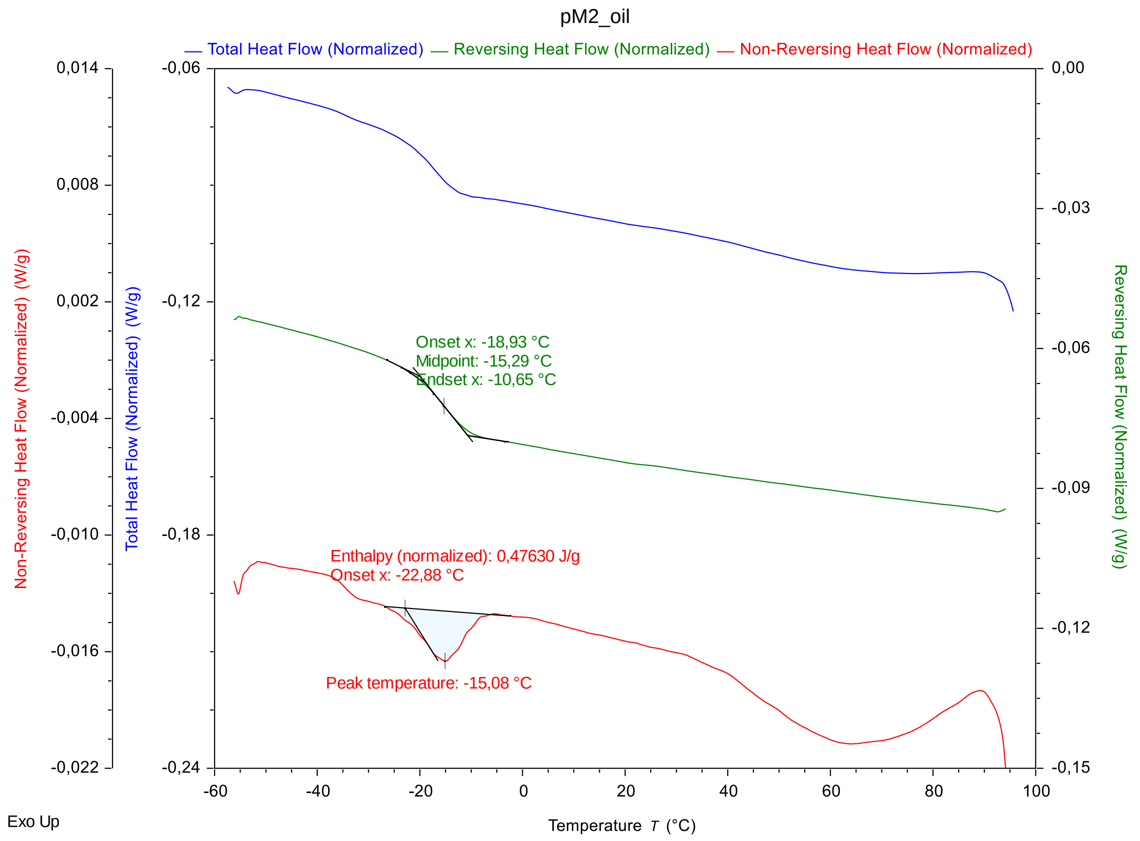

Figure S30: MDSC of **pM2** oil.

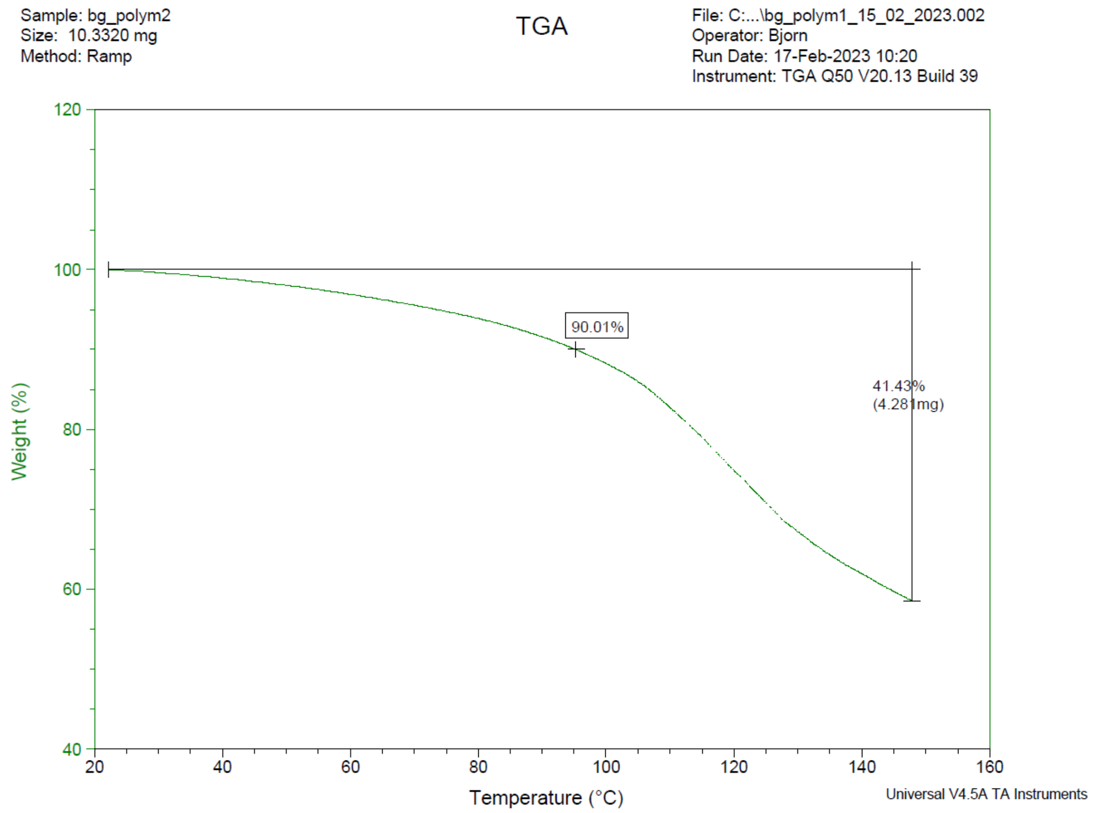

Figure S31: TGA thermogram of **pM2** oil.

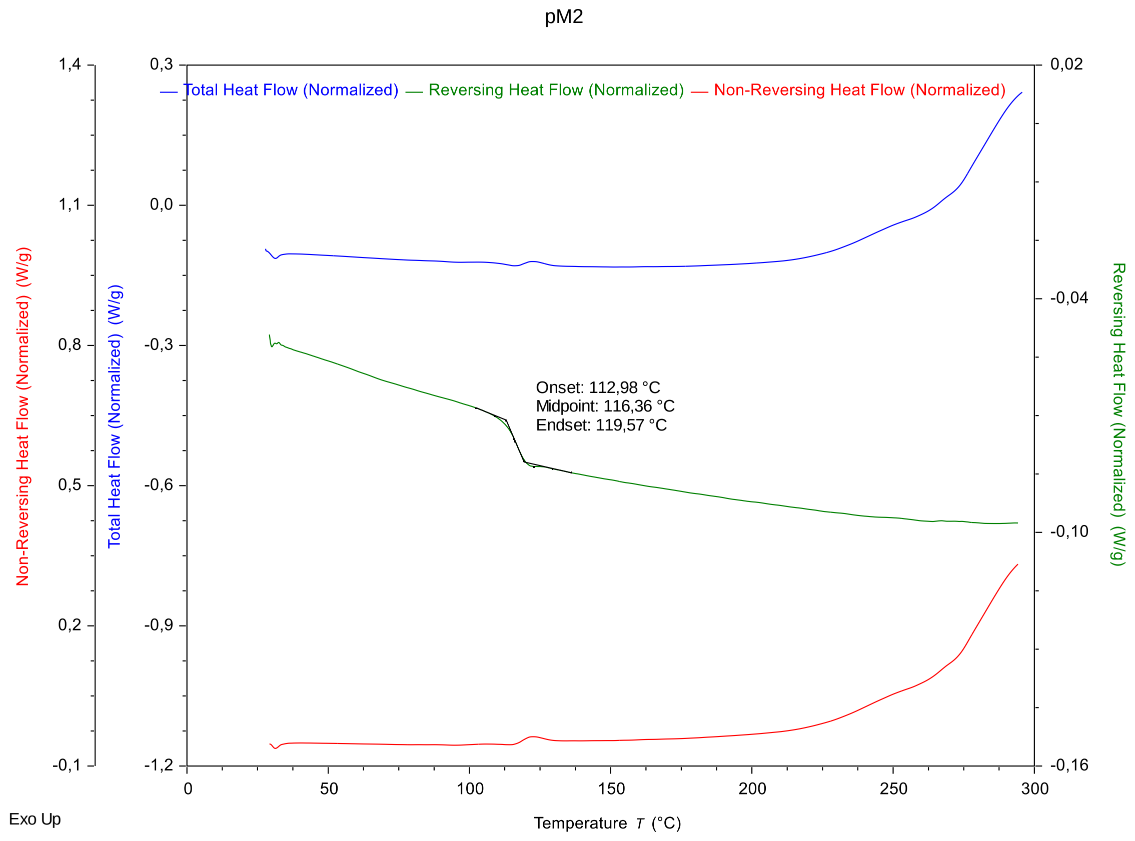

Figure S32: MDSC thermogram of precipitated **pM2**.

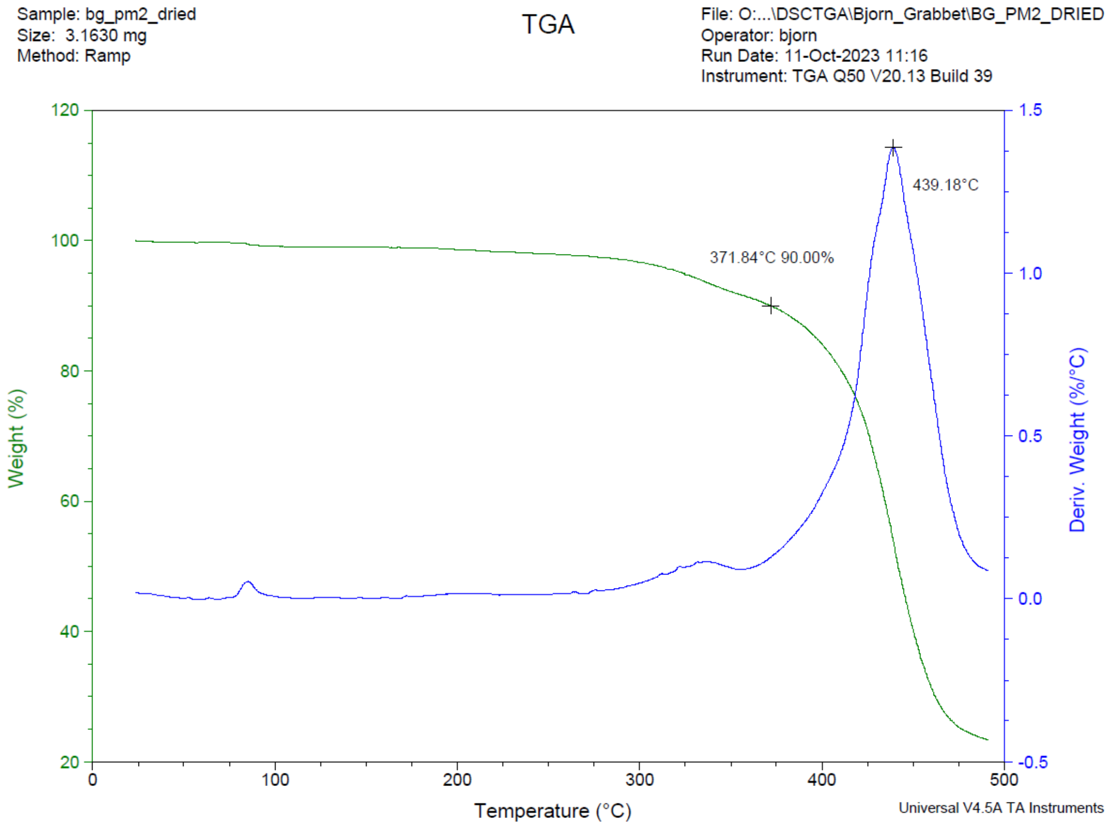

Figure S33: TGA thermogram of precipitated **pM2**.

pM3

^{1}H NMR (400 MHz, DMSO-D6) 6.26 – 5.50 (m), 5.04 – 4.76 (m), 4.66 – 4.39 (m), 4.34 – 3.97 (m), 3.22 – 3.01 (m), 2.20 – 2.02 (m), 2.01 – 1.81 (m) ppm.

^{13}C{^{1}H} NMR (100 MHz, DMSO-D6) δ 179.2 – 171.5, 137.3 – 125.0, 90.0 – 83.5, 80.1 – 74.9, 71.5 – 69.7, 46.8 – 42.3, 38.6 – 36.6 ppm.

1/λ (cm^{-1}): 2916 (wb, ν(**H–C**=C)), 1768 (s, ν(C=O)), 1483 (w, ν(CH_2)), 1450 (w, ν(CH_2)), 1379 (m, δ(C-H)), 1286 (w, ν(C–O)), 1259 (w, ν(O=**C–O**)), 1181 (s, ν(C–O)), 1100 (m, ν(C–O)), 1058 (m, δ(C–O)), 1023 (m δ(O=**C–O**R)), 988 (s, δ(C=C)), 920 (w, δ(C=C)), 892 (w, δ(C=C)), 874 (w, δ(C–C)), 838 (w, δ(C–C)), 781 (w, δ(C=C)), 735 (w, δ(C=C)), 685 (w, δ(C=C)).

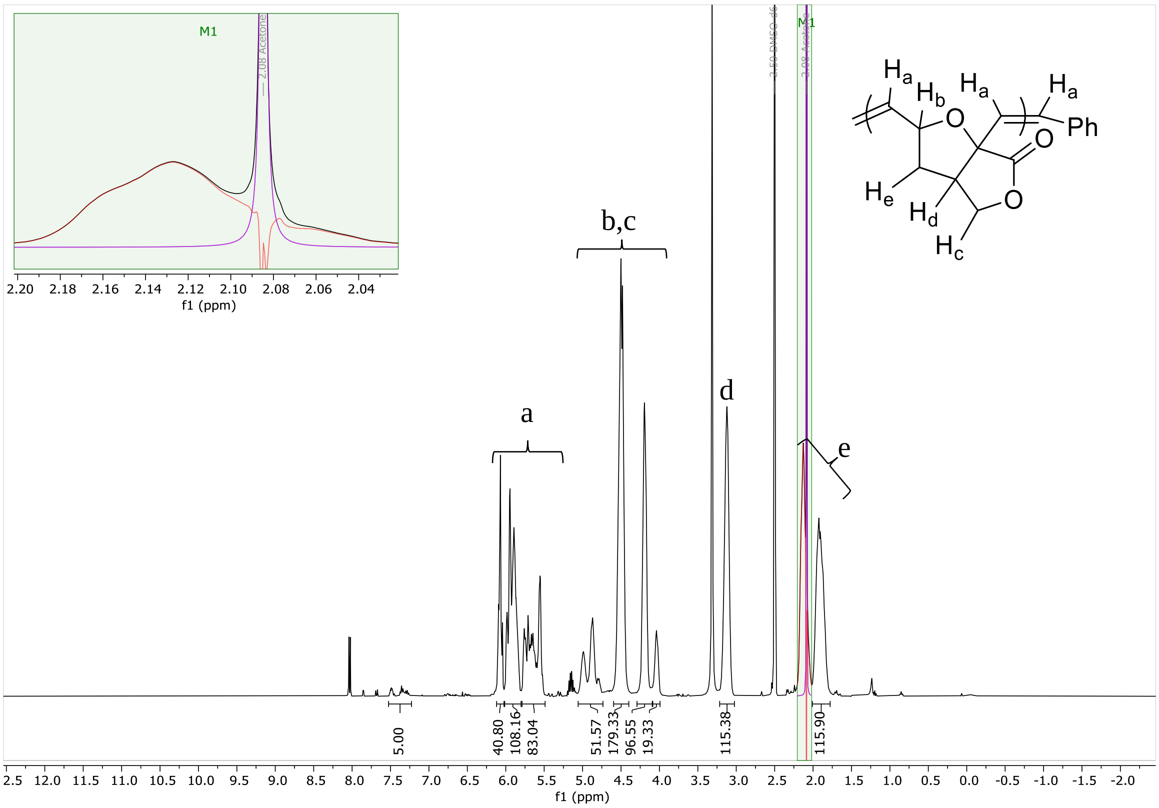

Figure S34: ^{1}H NMR spectrum of **pM3** (400 MHz, DMSO-D6). Resonance signals at 7.5 ppm corresponds to the phenyl end-group of the polymer.

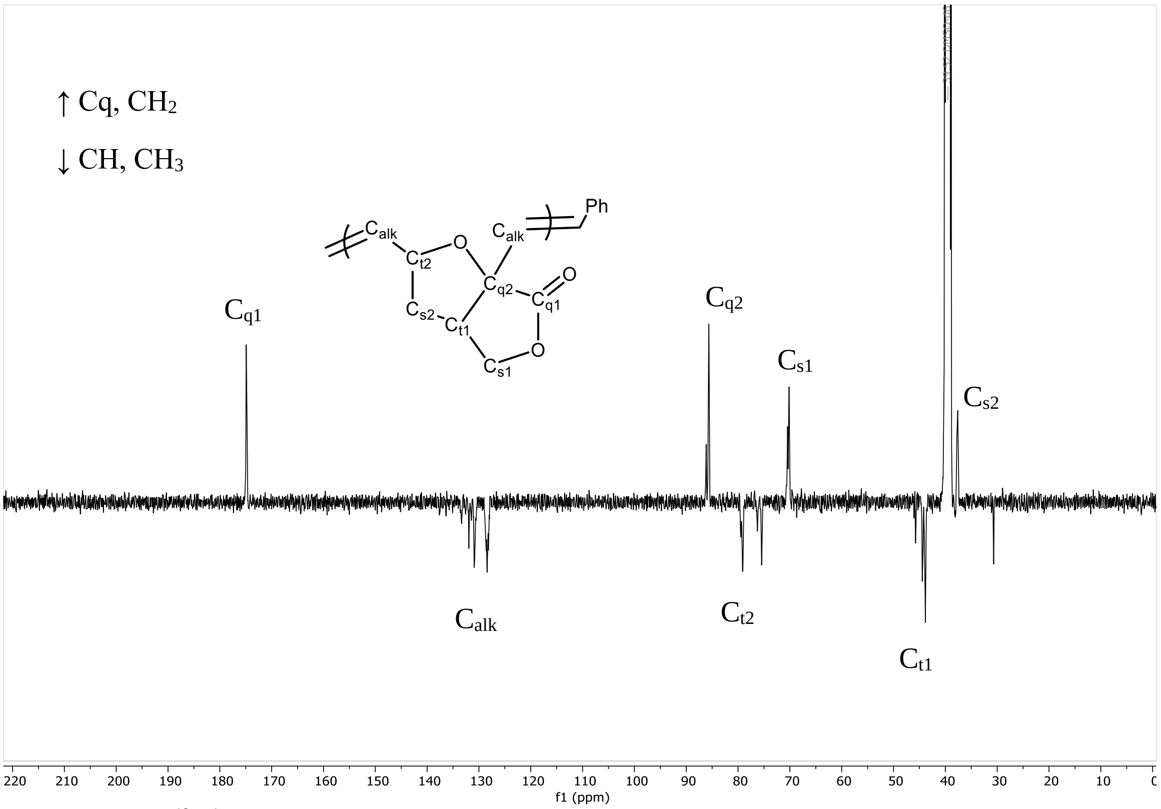

Figure S35: APT-$^{13}C\{^1H\}$ NMR spectrum of **pM3** (101 MHz, DMSO-D6). C_s = secondary carbon, C_t = tertiary carbon, C_q = quartenary carbon, C_{alk} = alkene carbon.

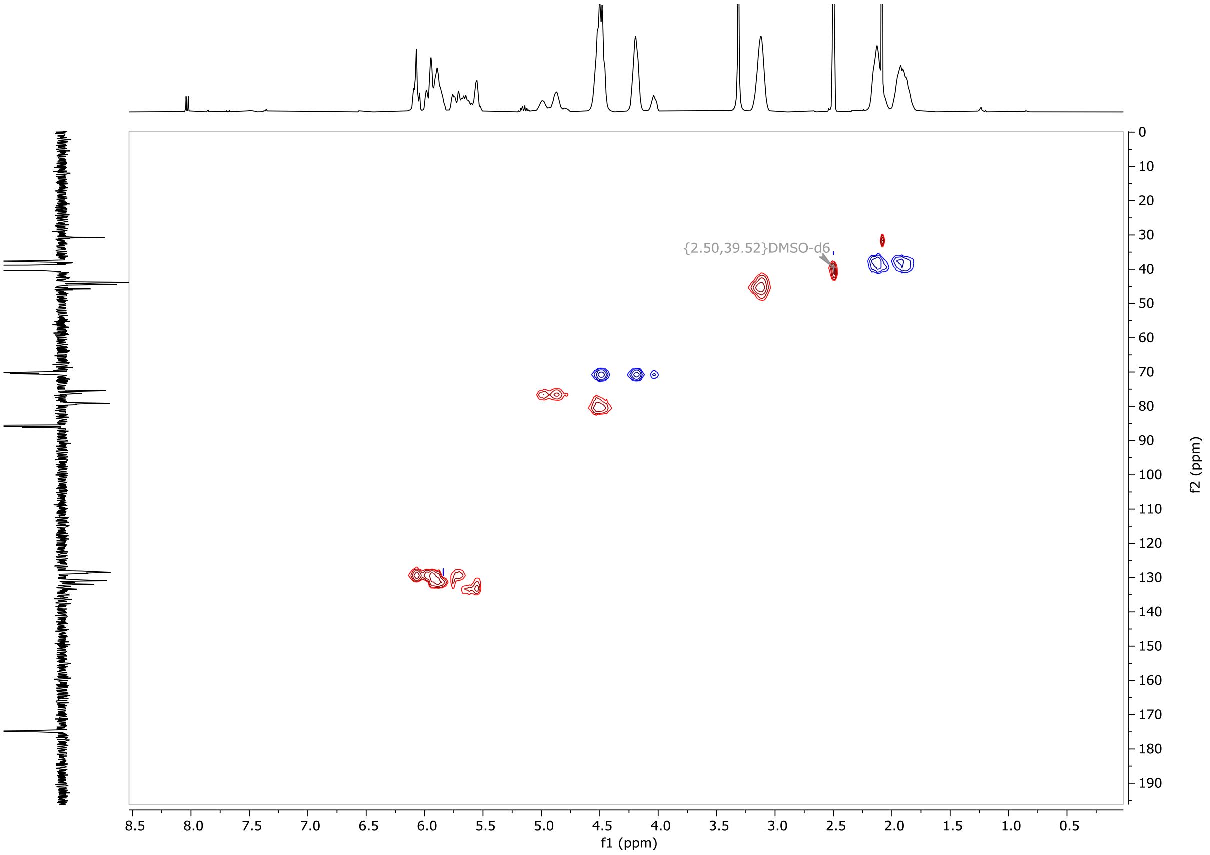

Figure S36: HSQC NMR spectrum of **pM3** (DMSO-D6).

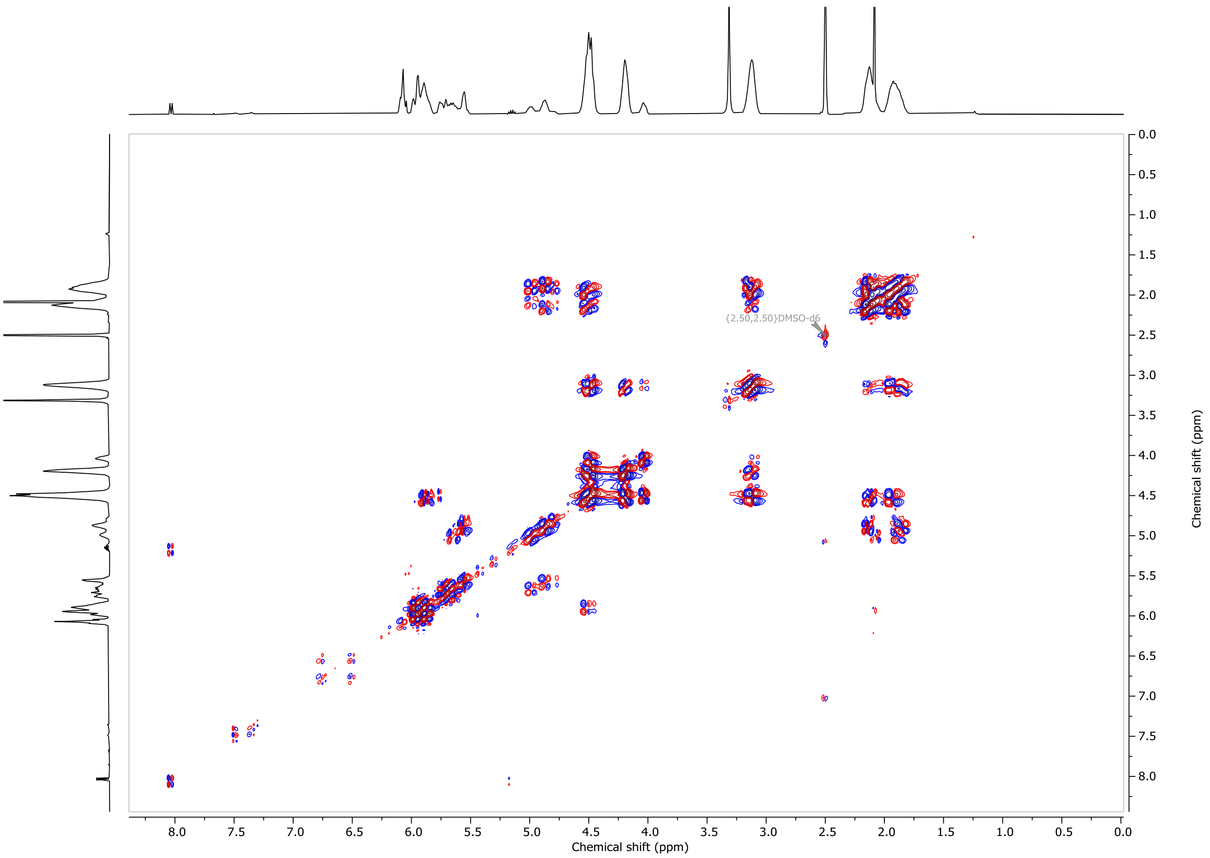

Figure S37: DQF-gCOSY of **pM3** (DMSO-D6).

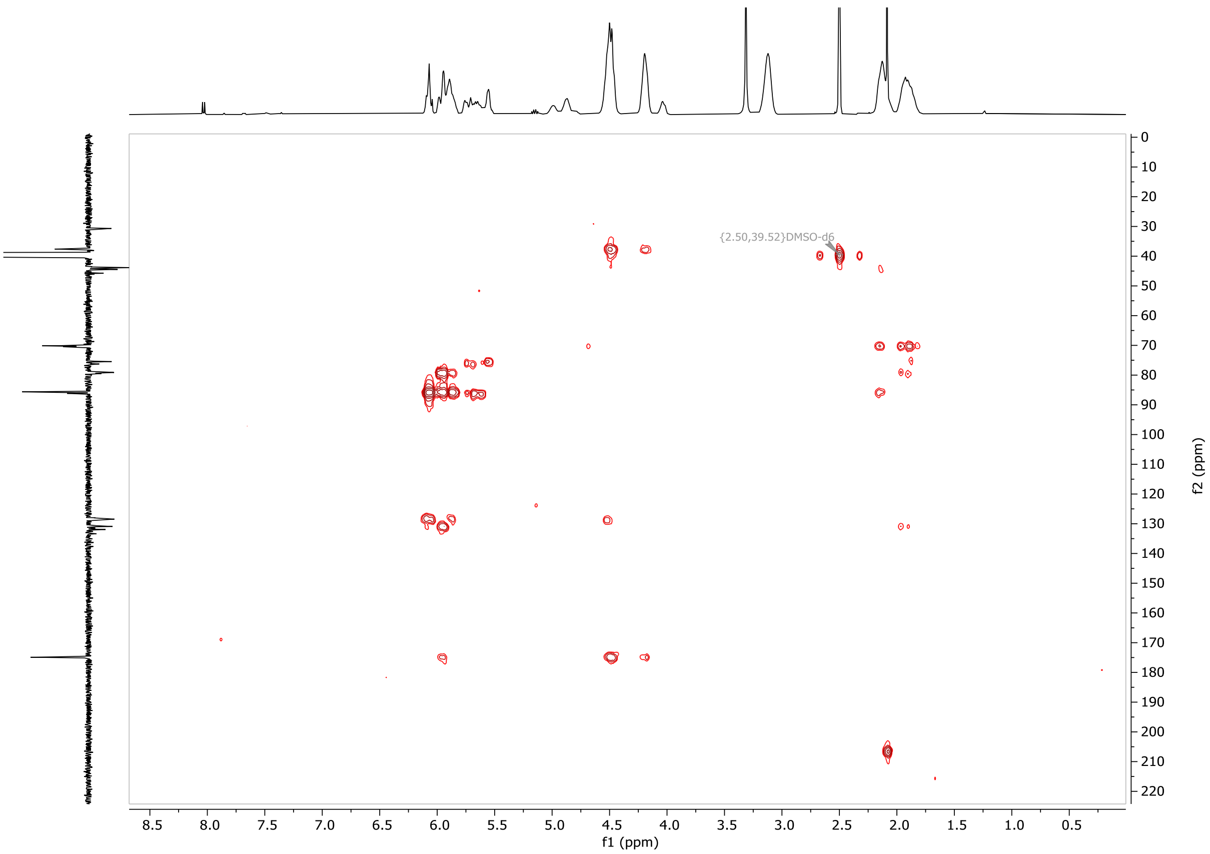

Figure S38: HMBC NMR spectrum of **pM3** (DMSO-D6).

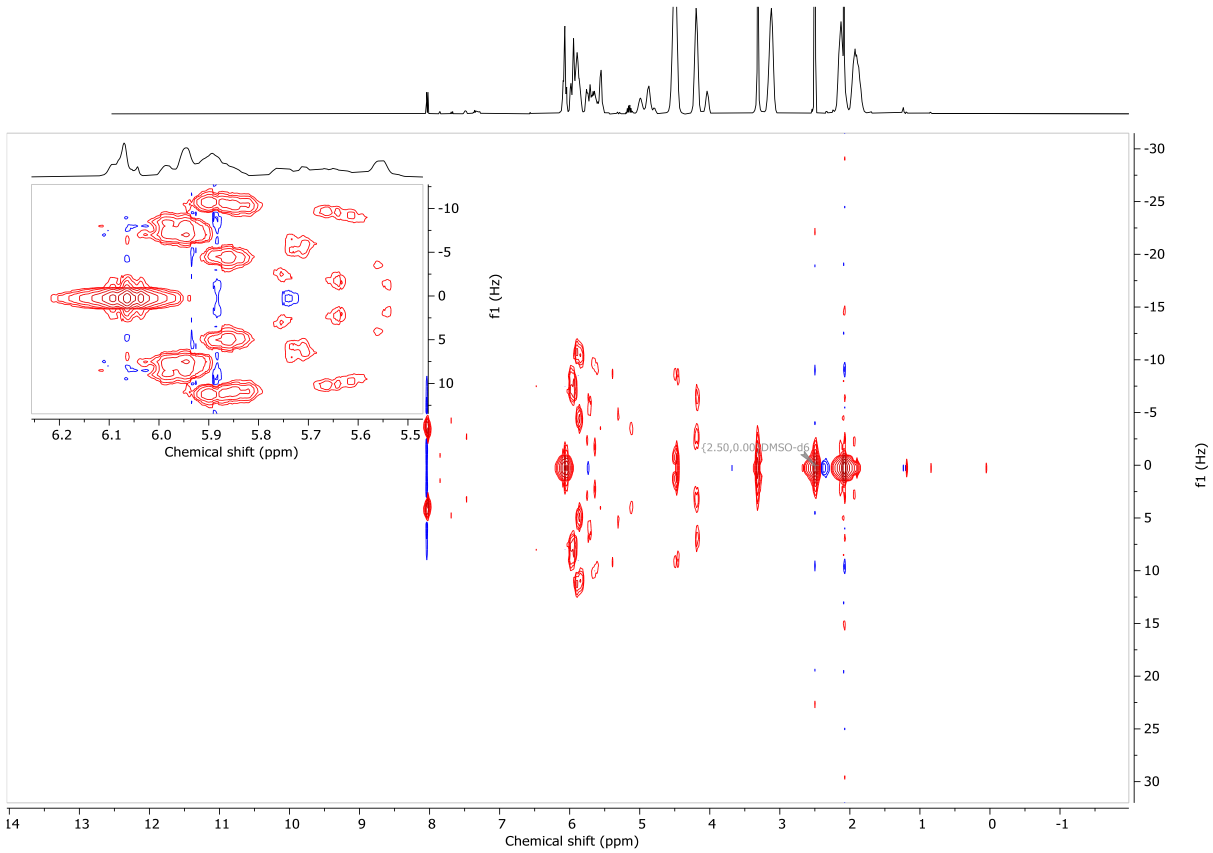

Figure S39: *J*-resolved NMR spectrum of **pM3** with enhanced region of interest (DMSO-D6).

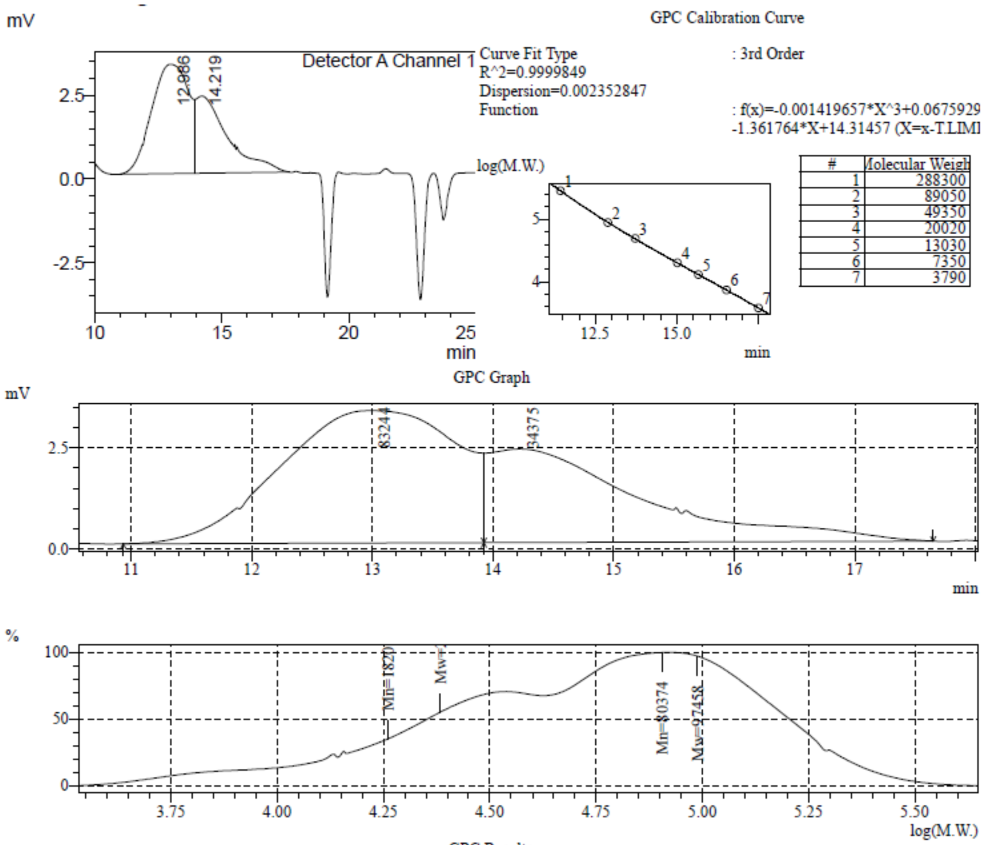

#	Molecular Weigh
1	288300
2	89050
3	49350
4	20020
5	13030
6	7350
7	3790

GPC Results

Peak#:1 (Detector A Channel 1)

[Peak Information]

Title	Time(min)	Molecular Weigl	Height
Start	10.933	447138	134
Top	12.986	83244	3262
End	13.925	42207	165

Area : 328680

[Average Molecular Weight]

Number Average Molecular Weight(Mn)	80374
Weight Average Molecular Weight(Mw)	97458
Mw/Mn	1.21255

Title	Time(min)	Molecular Weigl	Height
Top	14.219	34375	2303
End	17.650	3391	203

Area : 203969

[Average Molecular Weight]

Number Average Molecular Weight(Mn)	18203
Weight Average Molecular Weight(Mw)	24186
Mw/Mn	1.32872

Figure S40: GPC chromatogram of **pM3** with used calibration.

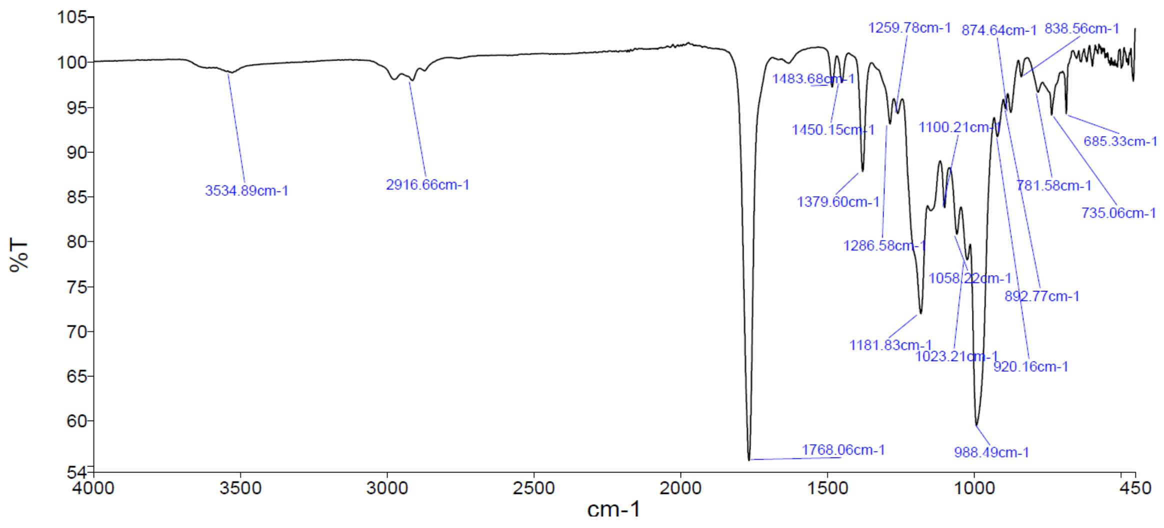

Figure S41: ATR-IR spectrum of **pM3**.

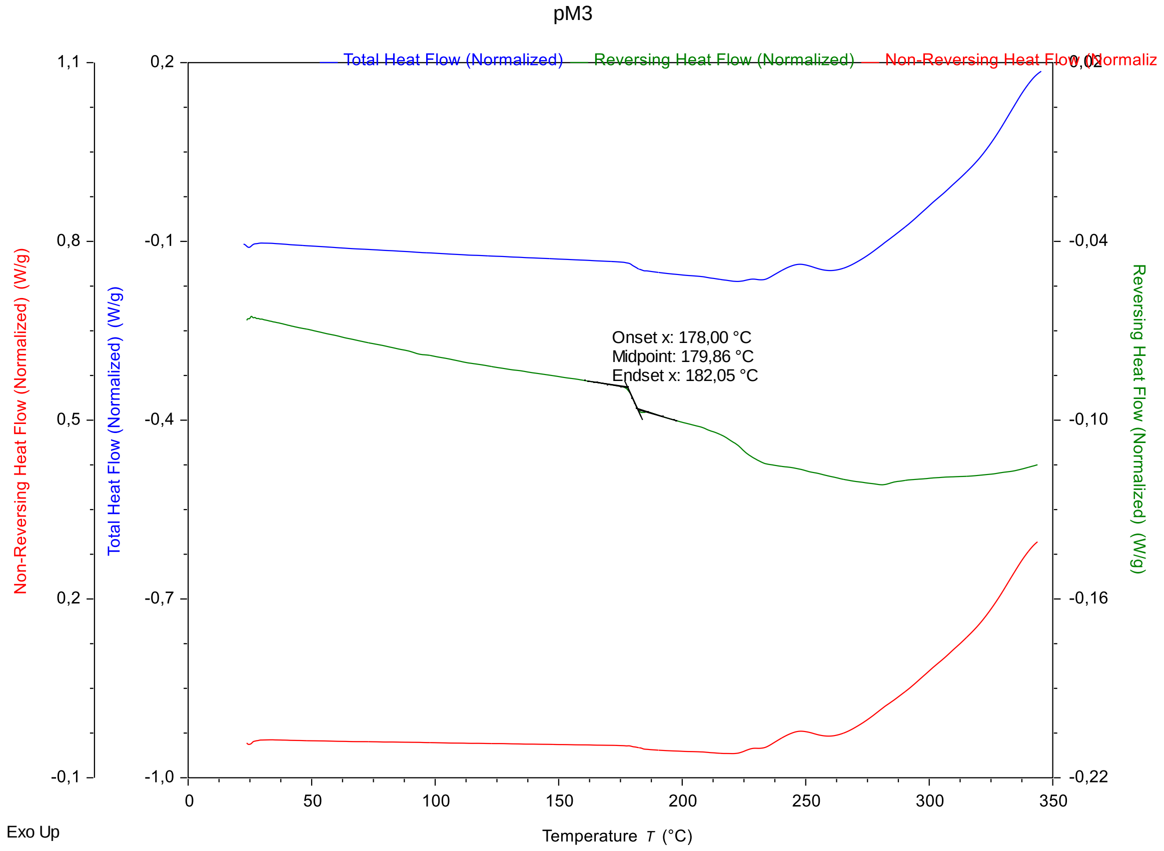

Figure S42: MDSC thermogram of **pM3**.

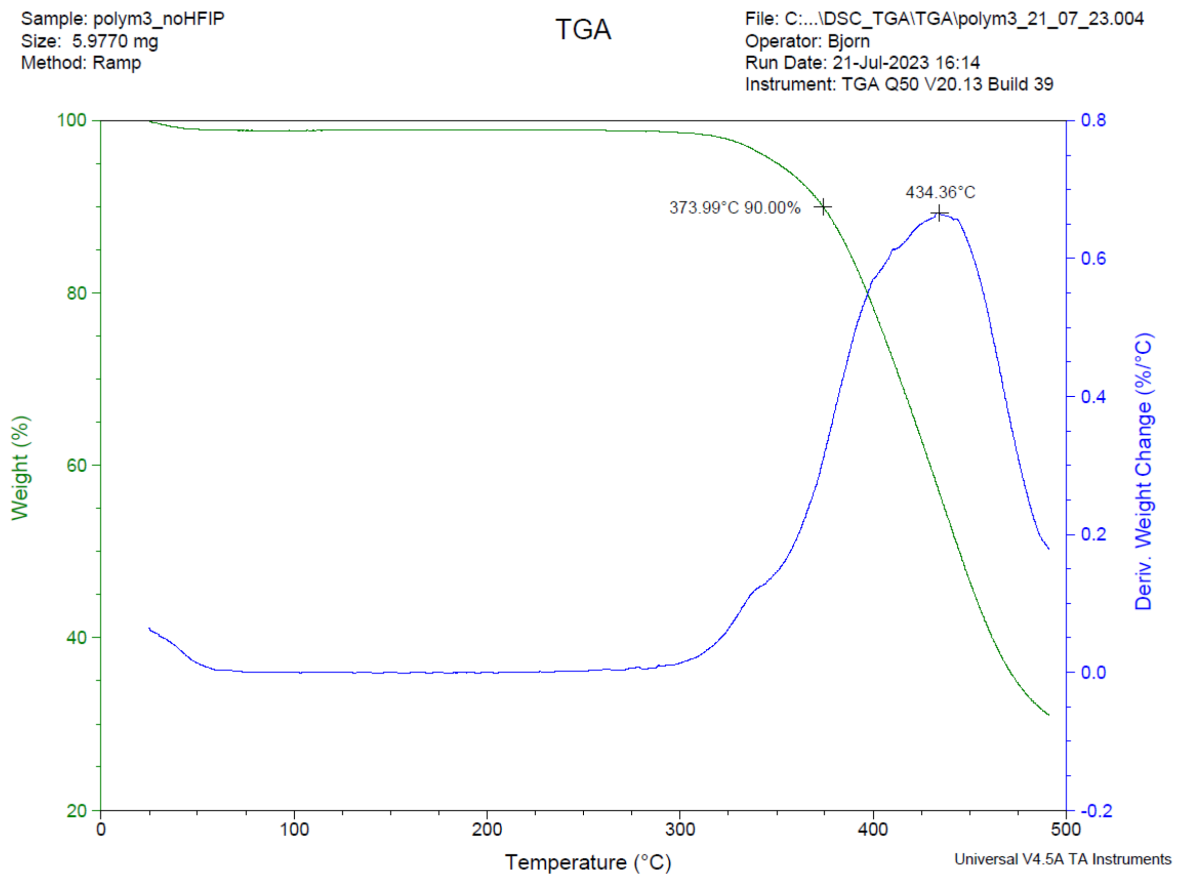

Figure S43: TGA thermogram of **pM3**.

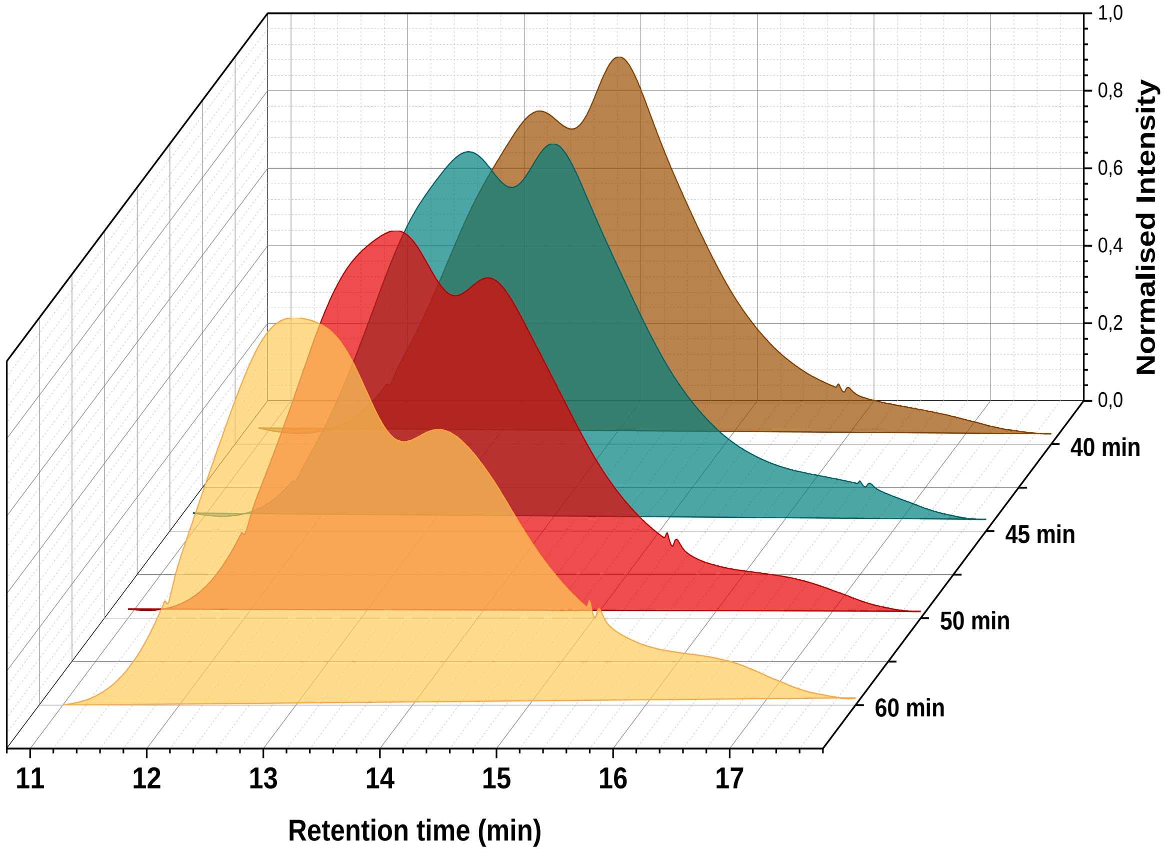

Figure S44: Stacked GPC chromatograms of **pM3** showcasing the bimodality and increase of higher molecular weight polymer over time.

Pre-quenched reactions

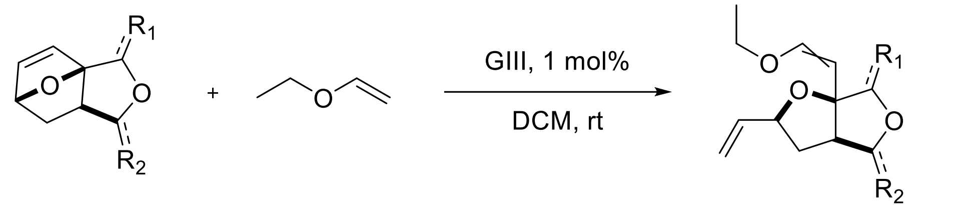

M1: R_1 = H, R_2 = O
M2: R_1 = R_2 = H
M3: R_1 = O, R_2 = H

qM1: R_1 = H, R_2 = O
qM2: R_1 = R_2 = H
qM3: R_1 = O, R_2 = H

To a solution of monomer (**M1** and **M3** 164.3 mg, **M2** 152.2 mg, final concentration in reaction 0.068 M) in DCM (3 mL) an excess of vinyl ethyl ether was added (2 mL) and stirred for 1 min. **GIII** (9.6 mg, 1 mol%) was dissolved in DCM (13 mL) in a separate vial and the two solutions were added together and stirred for 1 min (**M1**)/ 4 min (**M2**)/ 1 h (**M3**) at a stirring rate of 450 min^{-1}. The solvent was removed, HFIP was added (5 mL), the resulting suspension filtered through a 0.45 µm PTFE syringe filter to remove any residual degraded catalyst and the solvent removed in vacuo. The compounds **qM1**, **qM2** and **qM3** were isolated as brown oils in quantitative yields. The *cis*/*trans* ratio of the ether linkage was determined through integration of the corresponding ^{1}H NMR signals.

qM1

cis:*trans* ratio: 1:0.5

cis-**qM1**:

^{1}H NMR (400 MHz, DMSO-D6) δ 6.16 (dd, *J* = 6.7, 1.2 Hz, 1H), 5.83 (tdd, *J* = 17.0, 10.3, 6.7 Hz, 1H), 5.26 (ddd, *J* = 17.1, 7.5, 1.4 Hz, 1H), 5.17 – 5.04 (m, 1H), 4.55 (dd, *J* = 6.6, 1.2 Hz, 1H), 4.43 – 4.18 (m, 5H), 3.85 (q, *J* = 7.1 Hz, 2H), 3.37 (d, *J* = 8.2 Hz, 1H), 2.31 (dt, *J* = 12.7, 6.3 Hz, 2H), 1.99 (dddd, *J* = 12.0, 9.7, 8.4, 1.3 Hz, 1H), 1.24 – 1.11 (m, 6H) ppm

^{13}C{^{1}H} NMR (101 MHz, DMSO-D6) δ 177.8, 146.4, 138.4, 116.2, 105.2, 85.6, 80.1, 76.7, 68.1, 49.8, 35.4, 15.1 ppm

trans-**qM1**:

^{1}H NMR (400 MHz, DMSO-D6) δ 6.6 (dd, J = 12.7, 1.2 Hz, 1H), 5.8 (tdd, J = 17.0, 10.3, 6.7 Hz, 2H), 5.3 (ddd, J = 17.1, 7.5, 1.4 Hz, 3H), 5.2 – 5.0 (m, 2H), 5.0 (dd, J = 12.6, 1.2 Hz, 1H), 4.4 – 4.2 (m, 8H), 3.7 (q, J = 6.9 Hz, 2H), 3.2 (d, J = 7.5 Hz, 1H), 2.3 (dt, J = 12.7, 6.3 Hz, 3H), 1.9 (ddd, J = 10.6, 4.5, 1.9 Hz, 1H), 1.2 – 1.1 (m, 11H) ppm.

^{13}C{^{1}H} NMR (101 MHz, DMSO-D6) δ 177.8, 148.4, 138.1, 116.7, 103.0, 86.7, 79.3, 76.7, 64.6, 50.4, 34.0, 14.5 ppm.

1/λ (cm^{-1}): 2979 (m, ν(**H–C**=$C_{terminal}$)), 2939 (w, ν(**H–C**=C)), 2894 (w, ν(C–**C–H**)), 1770 (s, ν(C=O)), 1654 (m, ν(**H-C**=C)), 1445 (w, ν(CH_2)), 1382 (w, δ(C–**C–H**)), 1370 (w, δ(C–**C–H**)), 1295 (w, ν(C–O)), 1226 (w, ν(C–O)), 1196 (m, ν(C–O)), 1168 (w, ν(C–O)), 1098 (m, δ(C–**C–H**)), 1075 (m, ν(C–O)), 1020 (m, δ(C–O), 987 (m, δ(**H-C**=C)), 928 (w, δ(**H-C**=C)), 888 (w, δ(C–**C–H**)), 849 (w, δ(C–**C–H**)), 812 (w, δ(**H-C**=C)), 719 (w, δ(**H-C**=C)).

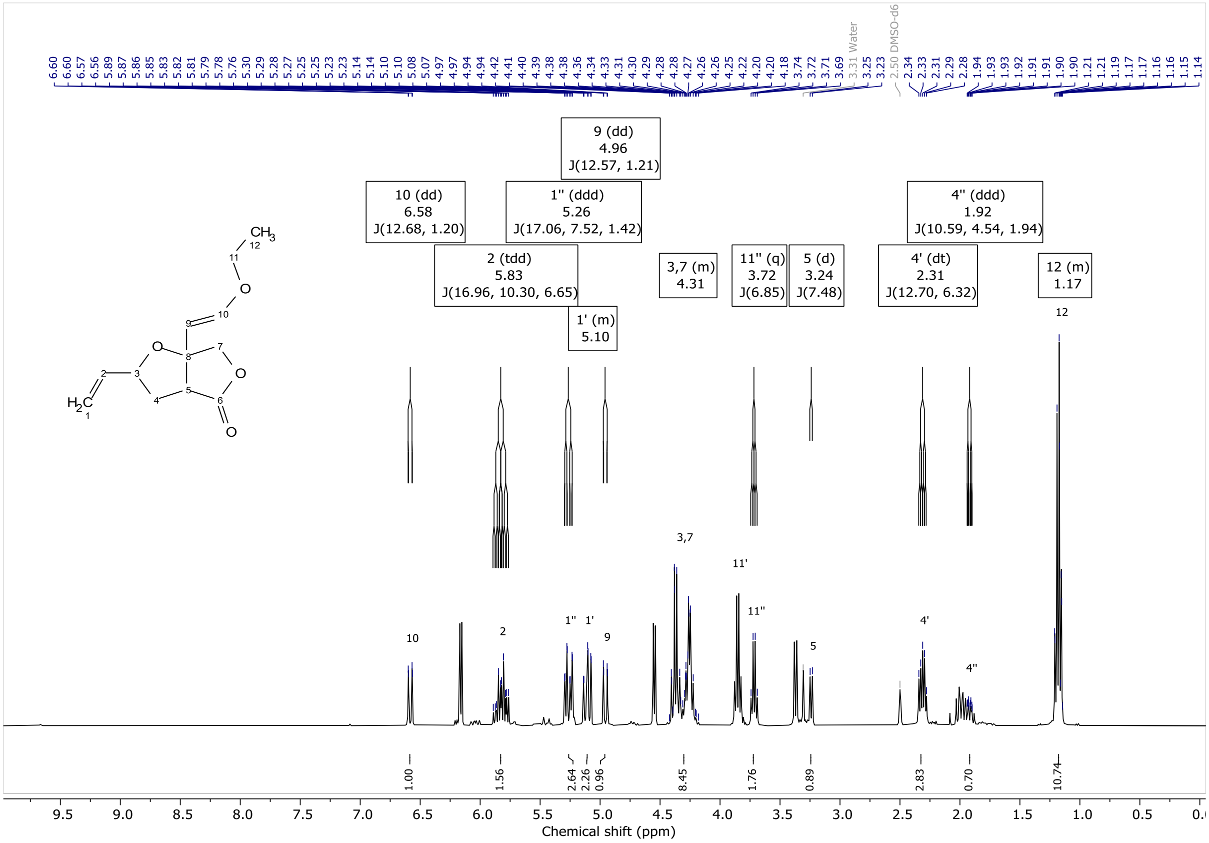

Figure S45: ^{1}H NMR spectrum of **qM1** (400 MHz, DMSO-D6). The labelled peaks correspond to *trans*-**qM1**, all the others are the *cis*-**qM1**.

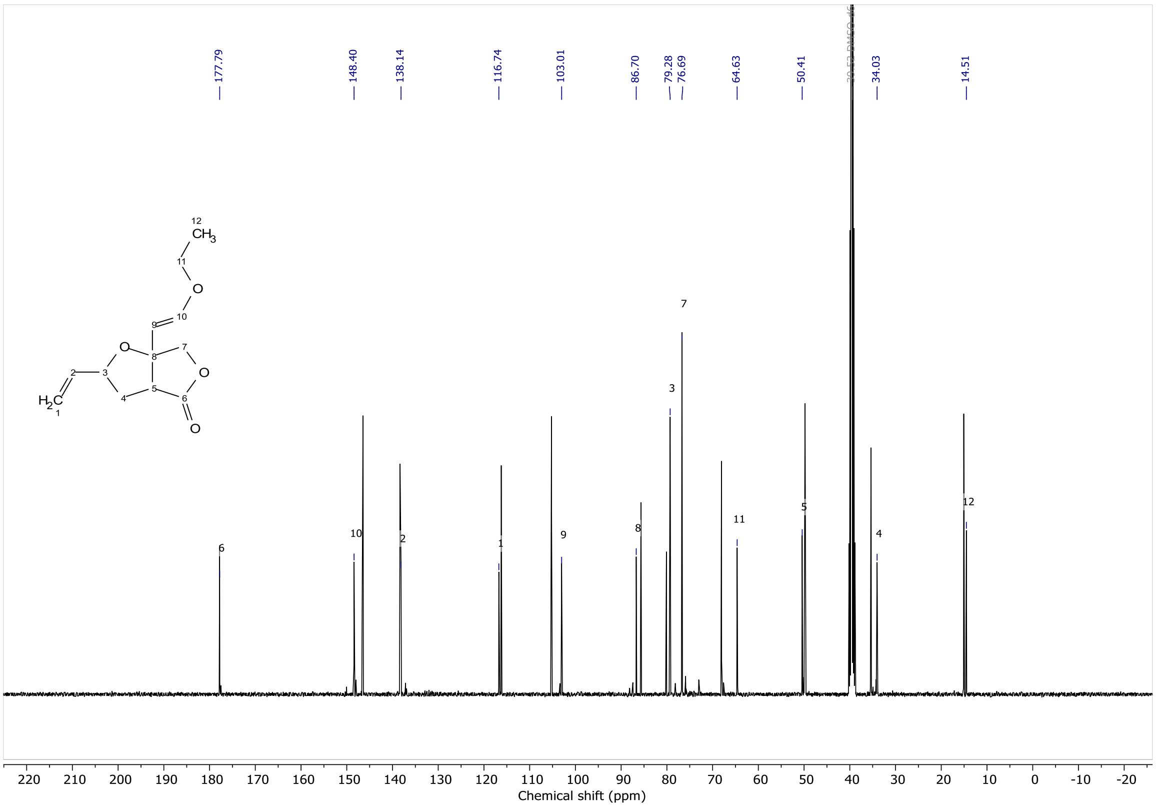

Figure S46: $^{13}C\{^{1}H\}$ NMR spectrum of **qM1** (101 MHz, DMSO-D6). The labelled peaks correspond to *trans*-**qM1**, all the others are the *cis*-**qM1**.

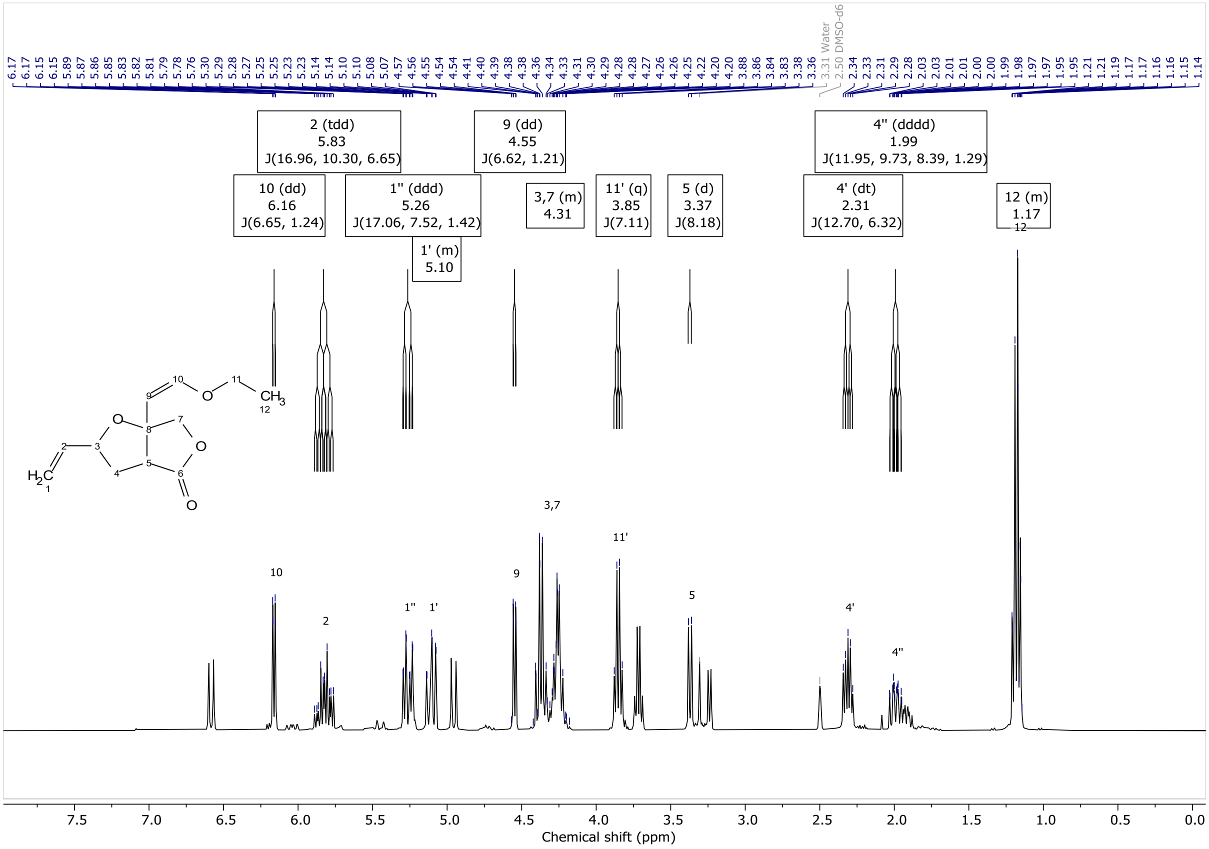

Figure S47: ^{1}H NMR spectrum of **qM1** (400 MHz, DMSO-D6). The labelled peaks correspond to *cis*-**qM1**, all the others are the *trans*-**qM1**.

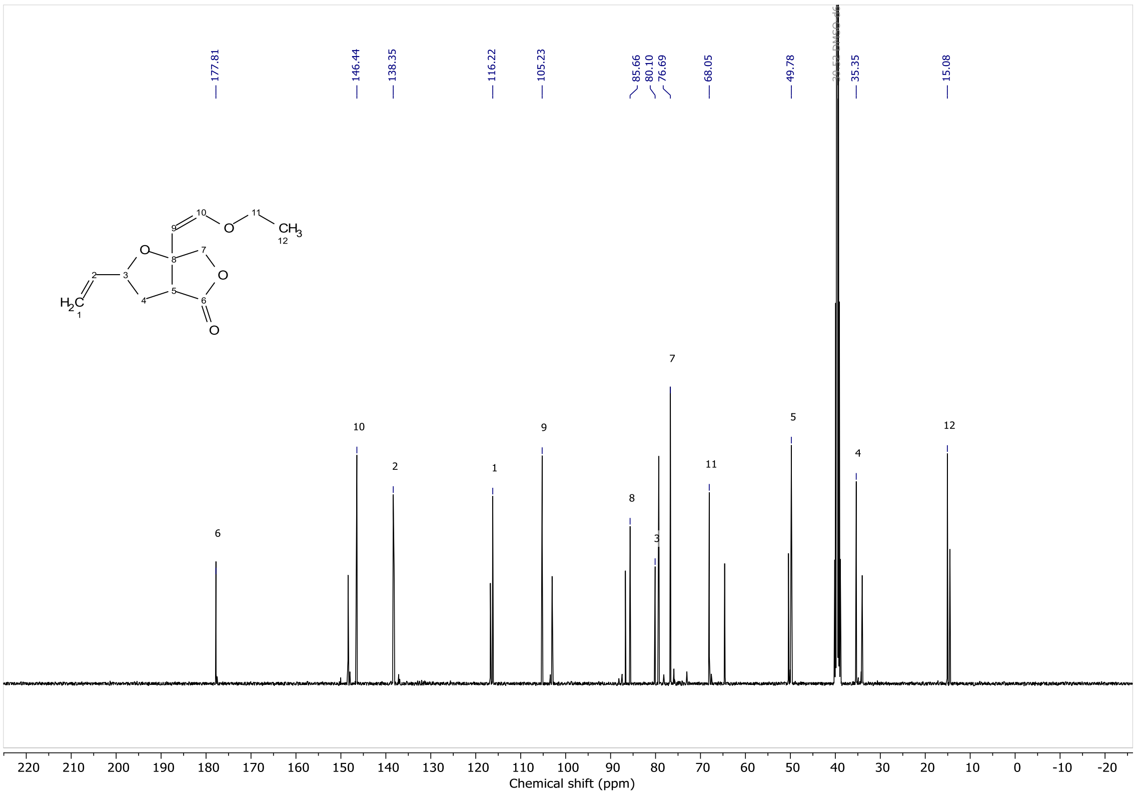

Figure S48: $^{13}C\{^{1}H\}$ NMR spectrum of **qM1** (101 MHz, DMSO-D6). The labelled peaks correspond to *cis*-**qM1**, all the others are the *trans*-**qM1**.

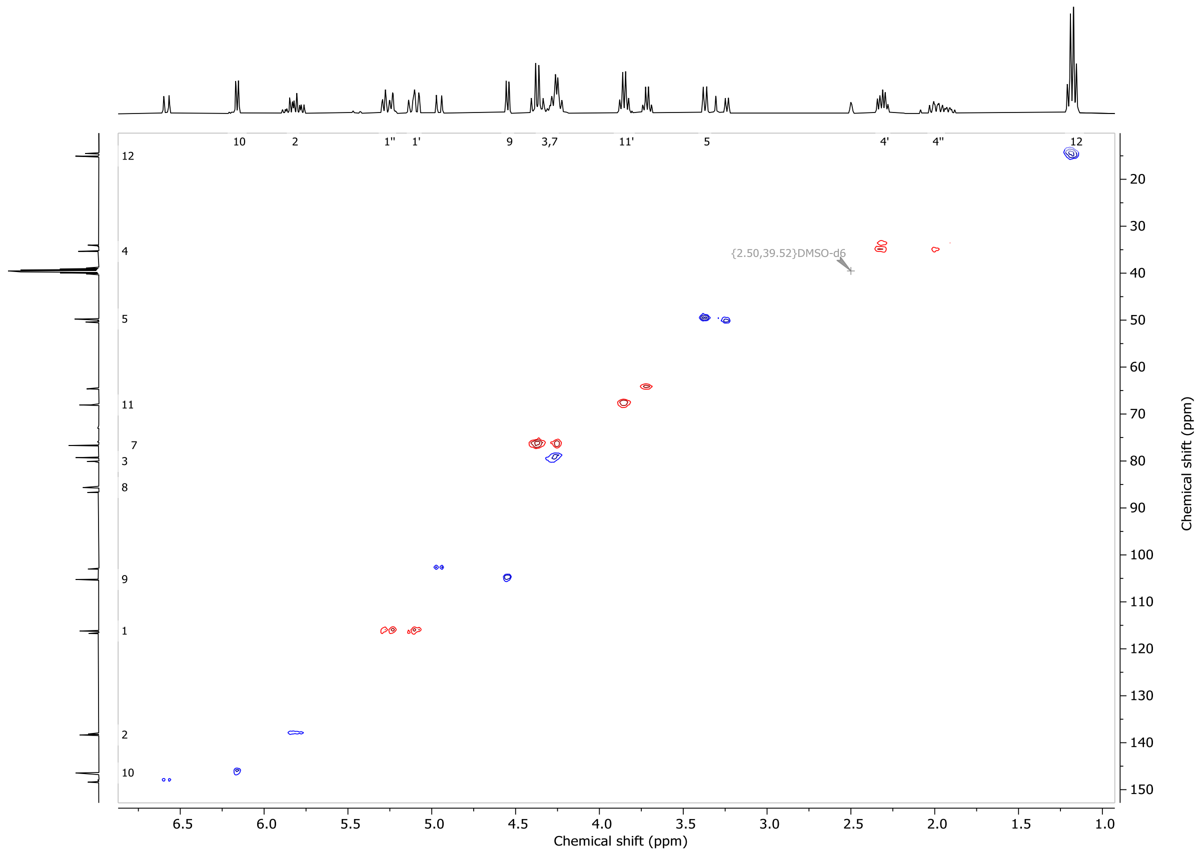

Figure S49: HSQC NMR spectrum of **qM1** (DMSO-D6).

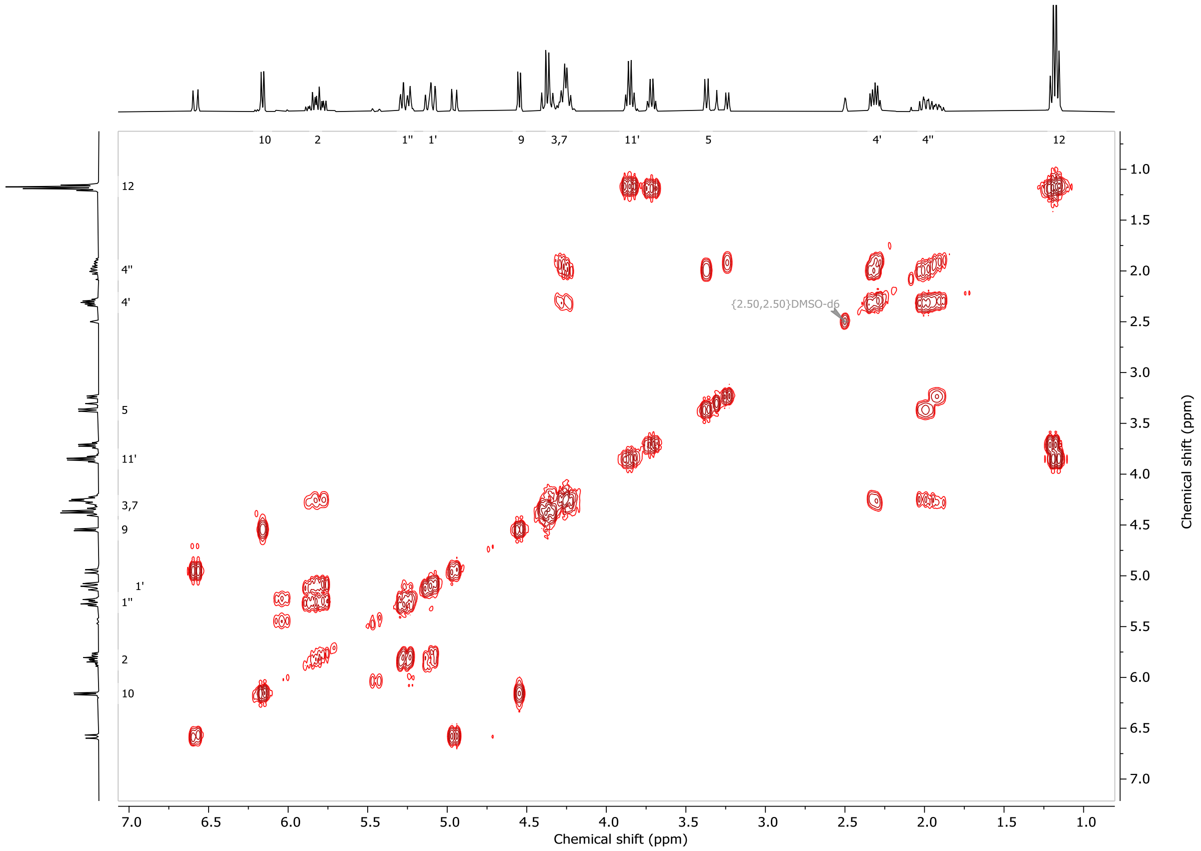

Figure S50: COSY NMR spectrum of **qM1** (DMSO-D6).

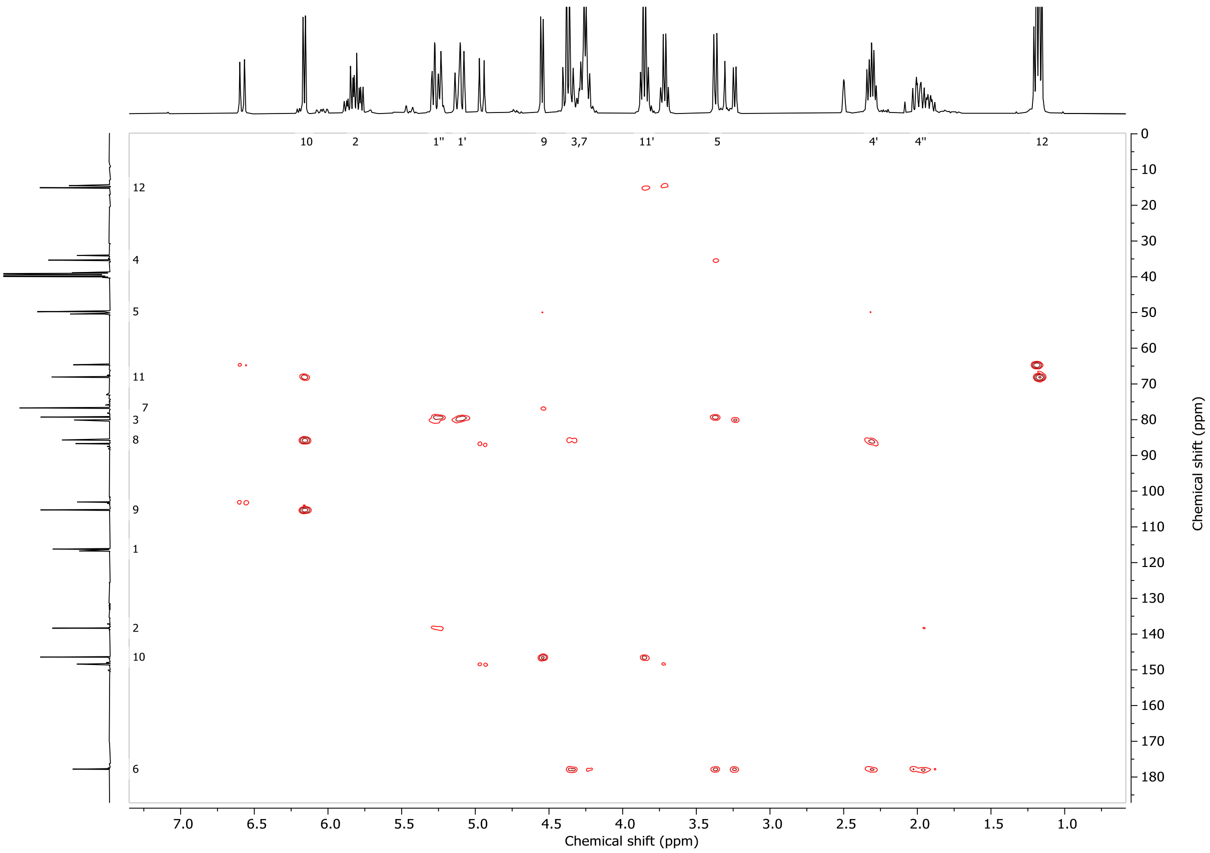

Figure S51: HMBC NMR spectrum of **qM1** (DMSO-D6).

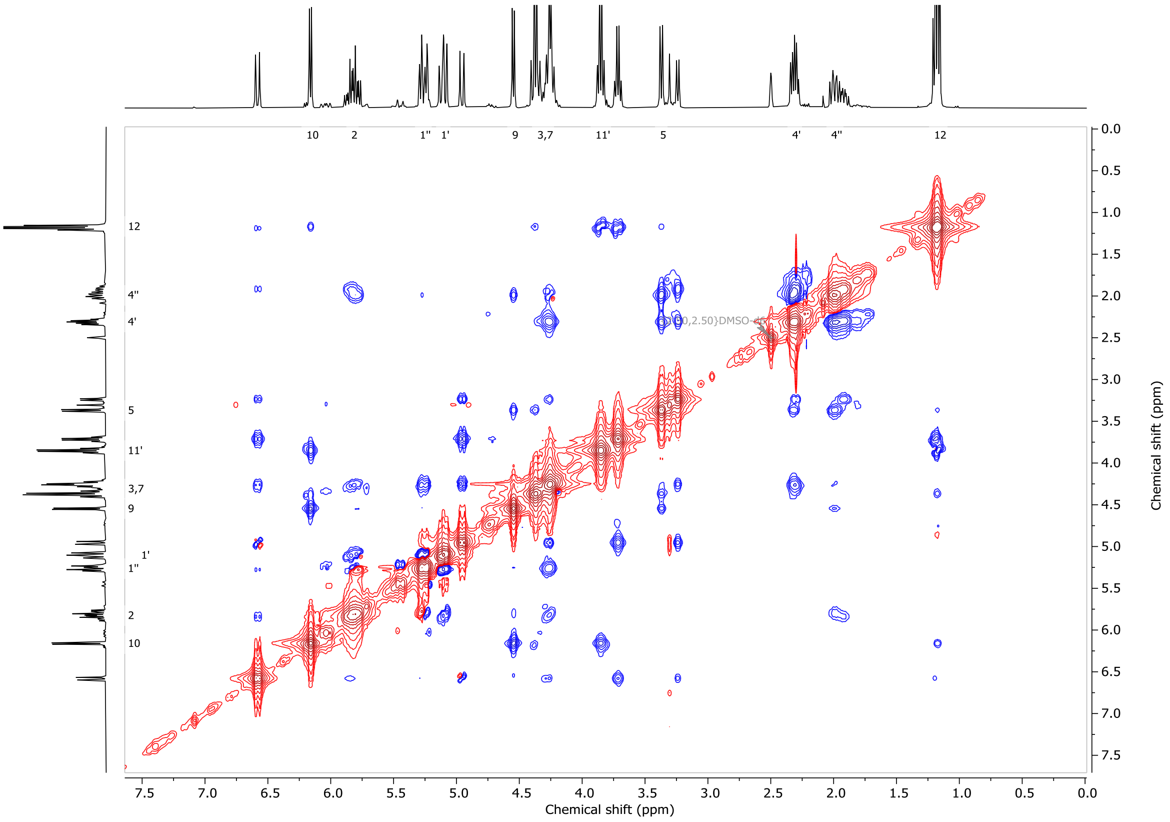

Figure S52: NOESY NMR spectrum of **qM1** (DMSO-D6). Positive phased signals (red) correspond to EXSY signals.

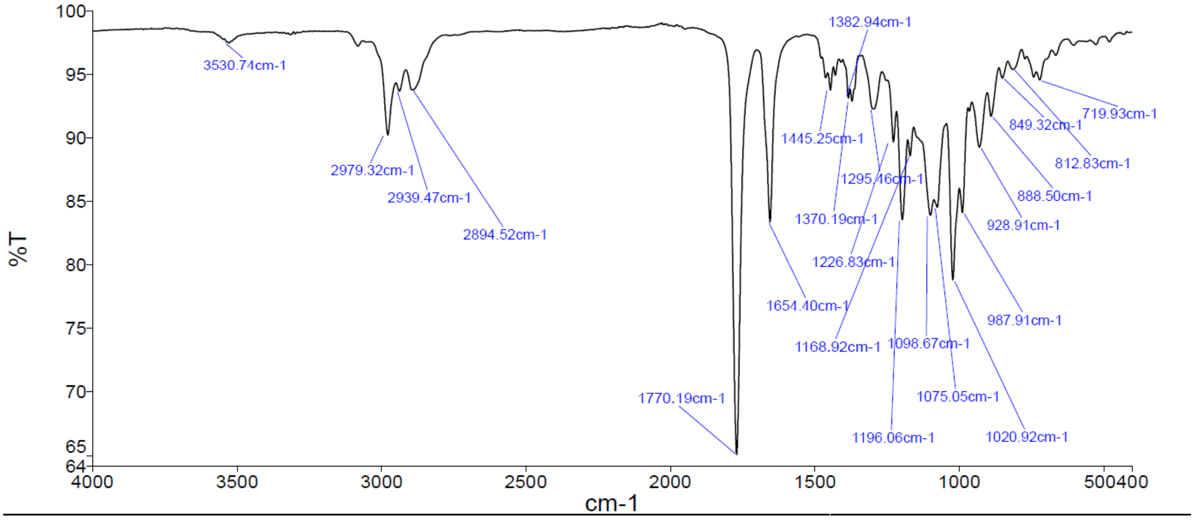

Figure S53: ATR-IR spectrum of **qM1**.

qM2
cis:trans ratio: 0.7:1

cis-**qM2***:*

^{1}H NMR (400 MHz, DMSO-D6) δ 6.0 (dd, J = 6.6, 1.2 Hz, 1H), 5.9 – 5.7 (m, 2H), 5.2 (ddd, J = 17.4, 12.6, 1.7 Hz, 2H), 5.1 (ddd, J = 12.6, 10.2, 1.7 Hz, 2H), 4.5 (dd, J = 6.8, 1.2 Hz, 1H), 4.4 (dt, J = 11.2, 6.0 Hz, 1H), 4.0 – 3.6 (m, 9H), 3.6 (d, J = 9.0 Hz, 0H), 2.9 (dt, J = 8.1, 4.1 Hz, 1H), 1.9 (dd, J = 12.1, 5.3 Hz, 2H), 1.8 (dtd, J = 22.9, 11.7, 8.4 Hz, 2H), 1.3 – 1.1 (m, 5H)..ppm

^{13}C{^{1}H} NMR (101 MHz, DMSO-D6) δ 145.1, 139.1, 115.5, 107.1, 90.9, [80.2, 78.6, 78.5, 74.3, 73.9, 67.6, 64.3], 49.6, 39.6, 39.5, 15.1 ppm / square brackets denote carbon 6,7 and 11 which are not distinguishable due to overlapping (see HSQC and HMBC).

trans-**qM2***:*

^{1}H NMR (400 MHz, DMSO-D6) δ 6.52 (dd, *J* = 12.6, 1.1 Hz, 1H), 5.89 – 5.69 (m, 2H), 5.22 (ddd, *J* = 17.4, 12.6, 1.7 Hz, 2H), 5.05 (ddd, *J* = 12.6, 10.2, 1.7 Hz, 2H), 4.95 (dd, *J* = 12.5, 1.2 Hz, 1H), 4.44 (dt, *J* = 11.3, 5.8 Hz, 1H), 3.97 – 3.62 (m, 9H), 3.44 (d, *J* = 9.0 Hz, 1H), 2.59 (td, *J* = 7.6, 3.4 Hz, 1H), 1.92 (dd, *J* = 12.1, 5.3 Hz, 2H), 1.75 (dtd, *J* = 22.9, 11.7, 8.4 Hz, 2H), 1.27 – 1.12 (m, 5H).ppm

^{13}C{^{1}H} NMR (101 MHz, DMSO-D6) δ 146.8, 138.9, 115.6, 104.7, 91.2, [80.4, 78.6, 78.5, 74.3, 73.9, 67.6, 64.3], 49.9, 38.2, 14.6. ppm / square brackets denote carbon 6,7 and 11 which are not distinguishable due to overlapping (see HSQC and HMBC).

1/λ (cm^{-1}): 2974 (m, ν(**H–C**=$C_{terminal}$)), 2931 (w, ν(**H–C**=C)), 2857 (m, ν(**H–C**–C)), 1652 (m, ν(**H–C**=C)), 1477 (w, ν(CH_2)), 1444 (w, ν(CH_2)), 1426 (w, ν(CH_2)), 1382 (w, δ(C–**C–H**)), 1304 (w, ν(C–O)), 1259 (w, δ(C–O)), 1184 (m, ν(C–O)), 1097 (m, δ(C–**C–H**)), 1015 (m, δ(**H–C**=C)), 992 (m, δ(**H–C**=C)), 928 (m, δ(C–**C–H**)) , 856 (w, δ(C–**C–H**)), 795 (w, δ(**H–C**=C)), 709 (w, δ(**H–C**=C)).

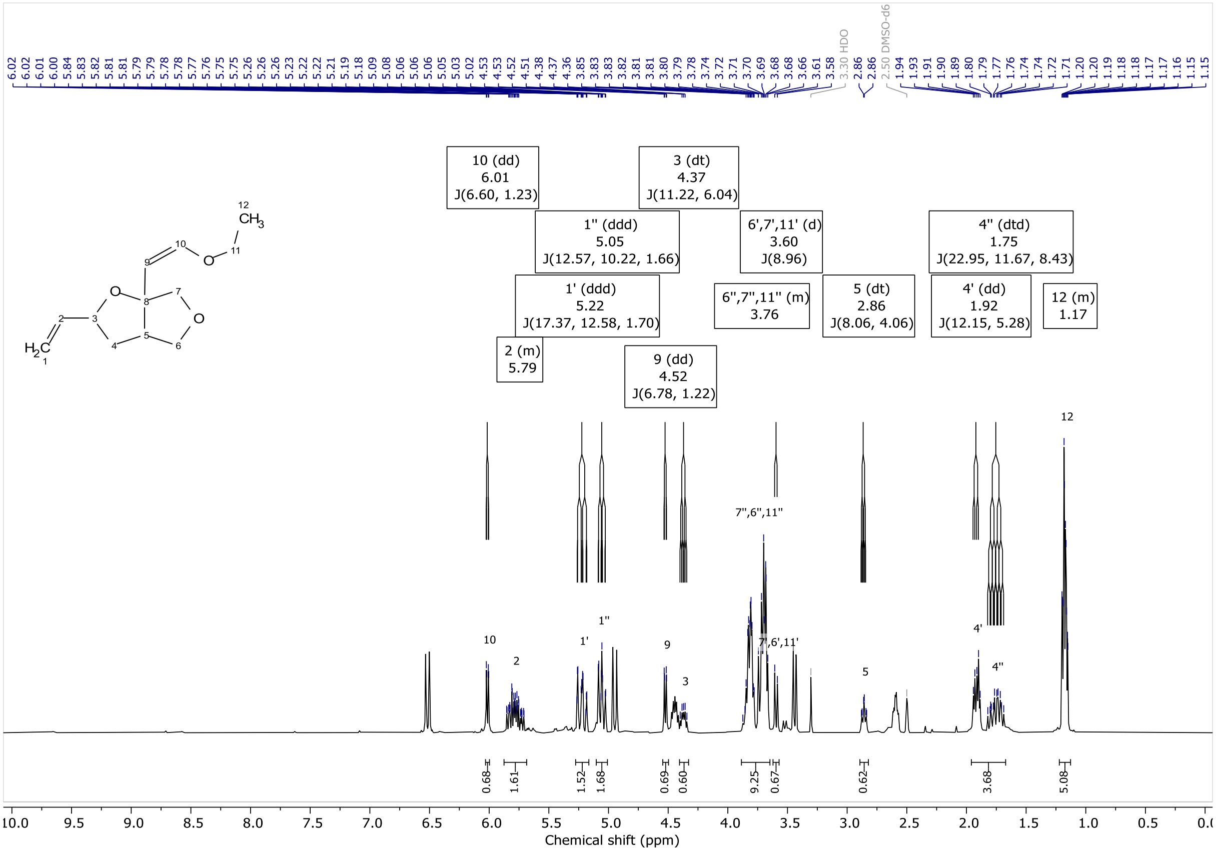

Figure S54: ^{1}H NMR spectrum of **qM2** (400 MHz, DMSO-D6). The labelled peaks correspond to *cis*-**qM2**, all the others are the *trans*-**qM2**

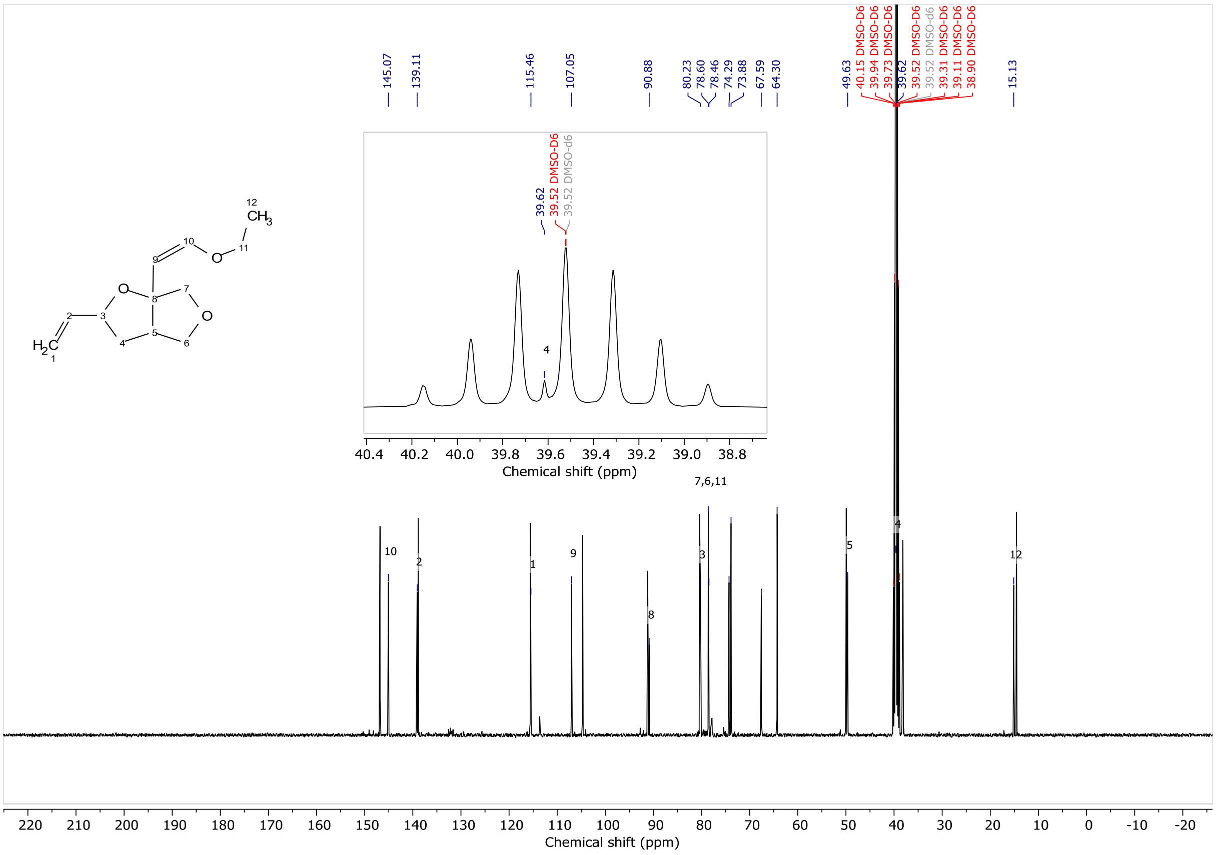

Figure S55: $^{13}C\{^{1}H\}$ NMR spectrum of **qM2** (101 MHz, DMSO-D6). The labelled peaks correspond to *cis*-**qM2**, all the others are the *trans*-**qM2**

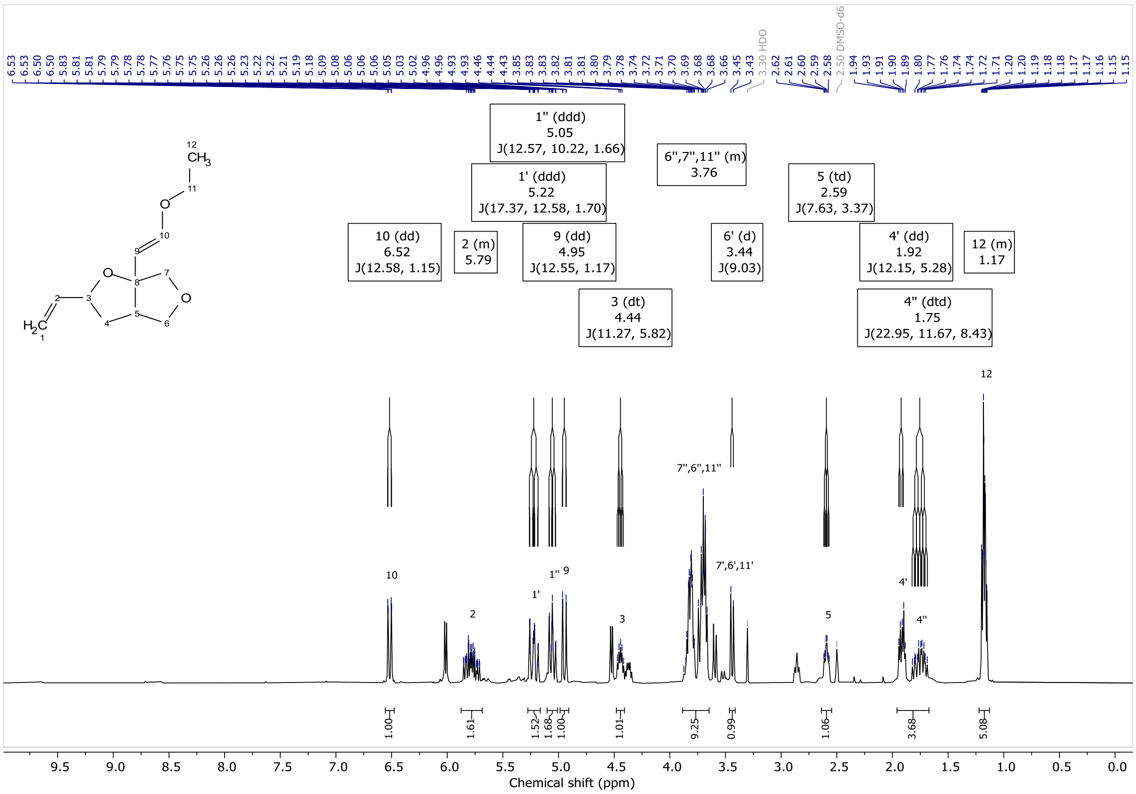

Figure S56: ^{1}H NMR spectrum of **qM2** (400 MHz, DMSO-D6). The labelled peaks correspond to *trans*-**qM2**, all the others are the *cis*-**qM2**.

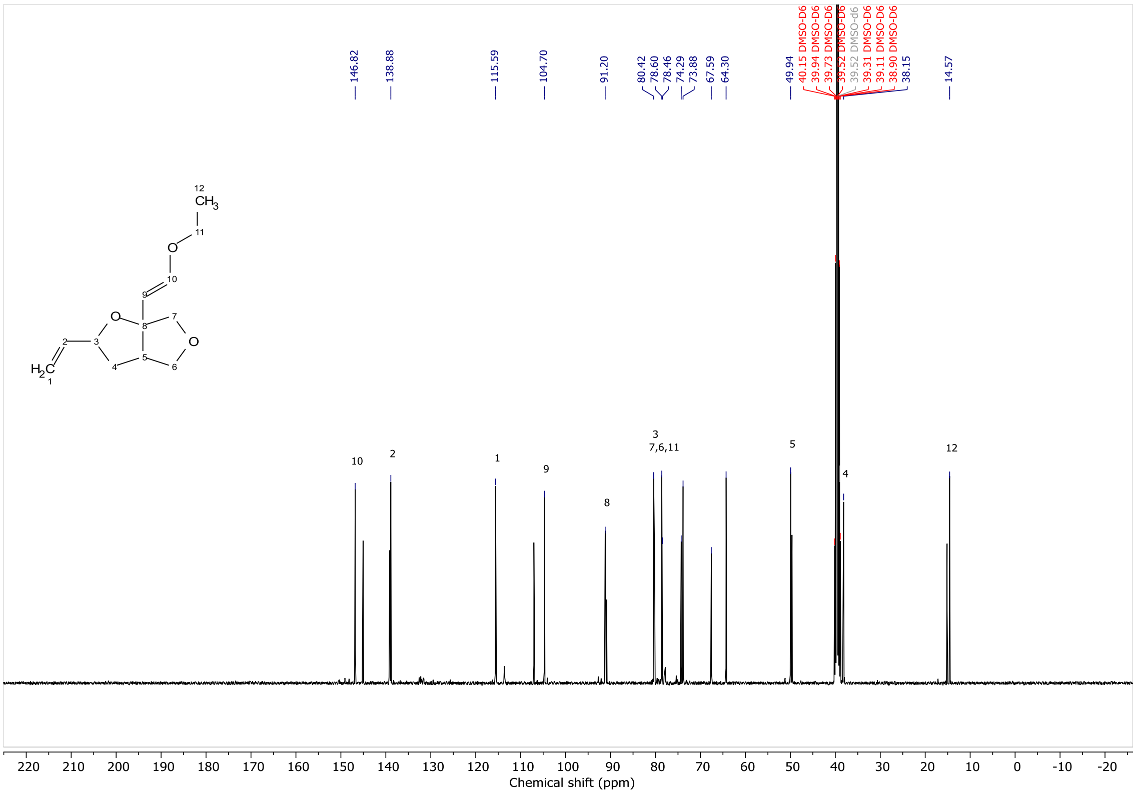

Figure S57: $^{13}C\{^{1}H\}$ NMR spectrum of **qM2** (101 MHz, DMSO-D6). The labelled peaks correspond to *trans*-**qM2**, all the others are the *cis*-**qM2**.

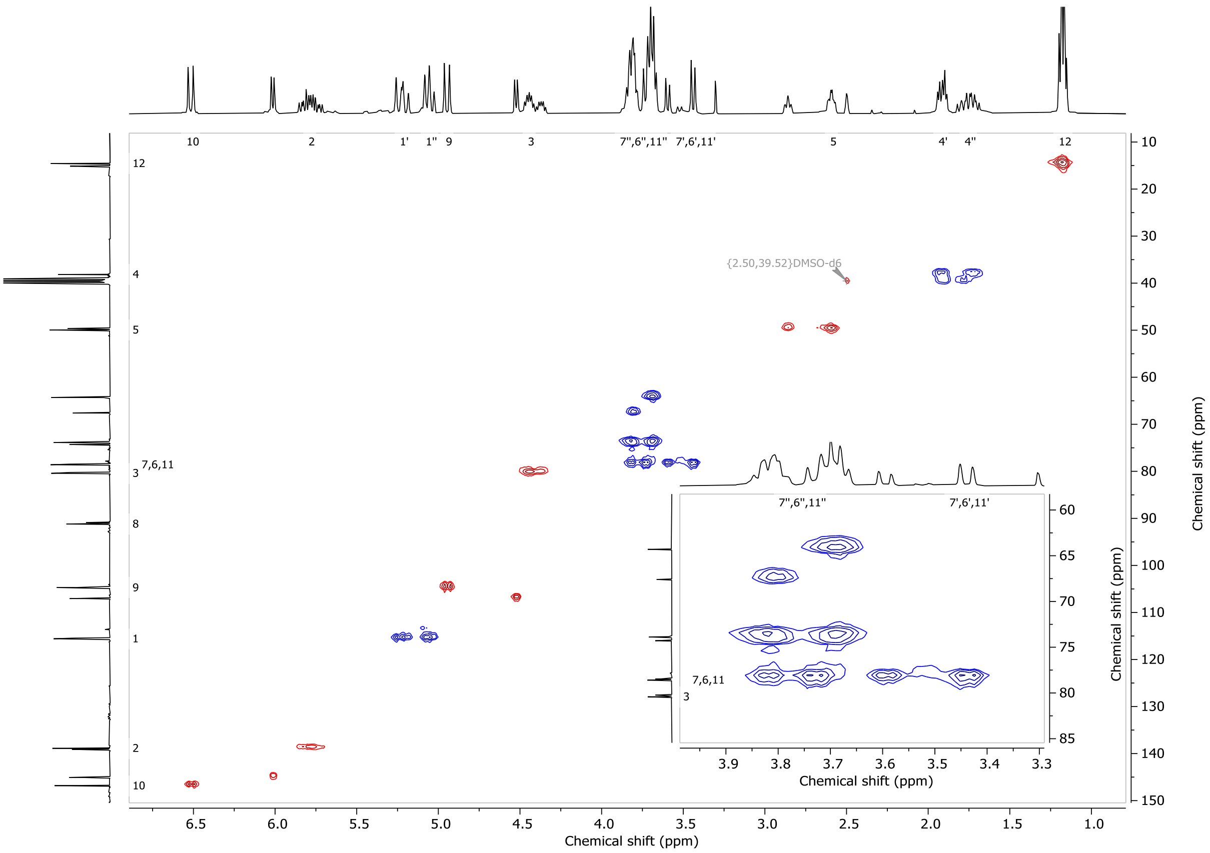

Figure S58: HSQC NMR spectrum of **qM2** (DMSO-D6).

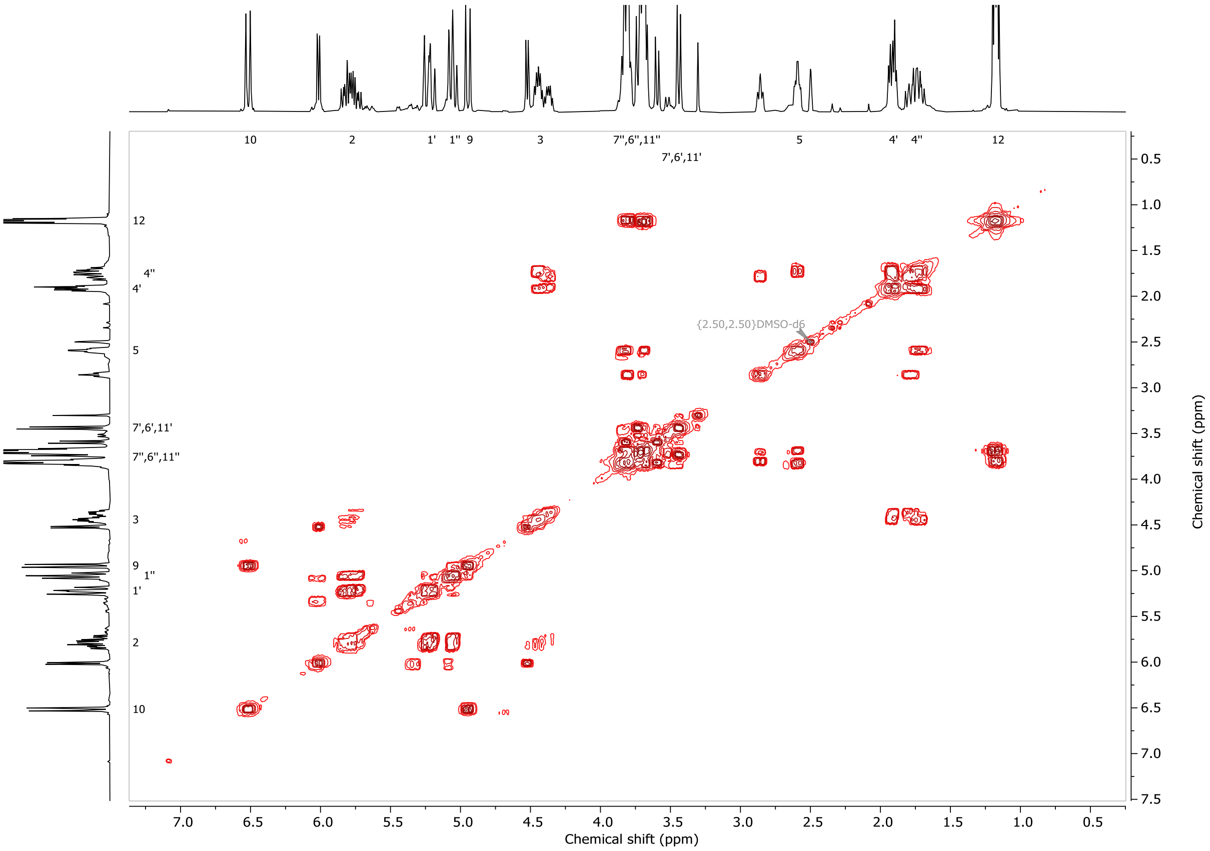

Figure S59: COSY NMR spectrum of **qM2** (DMSO-D6).

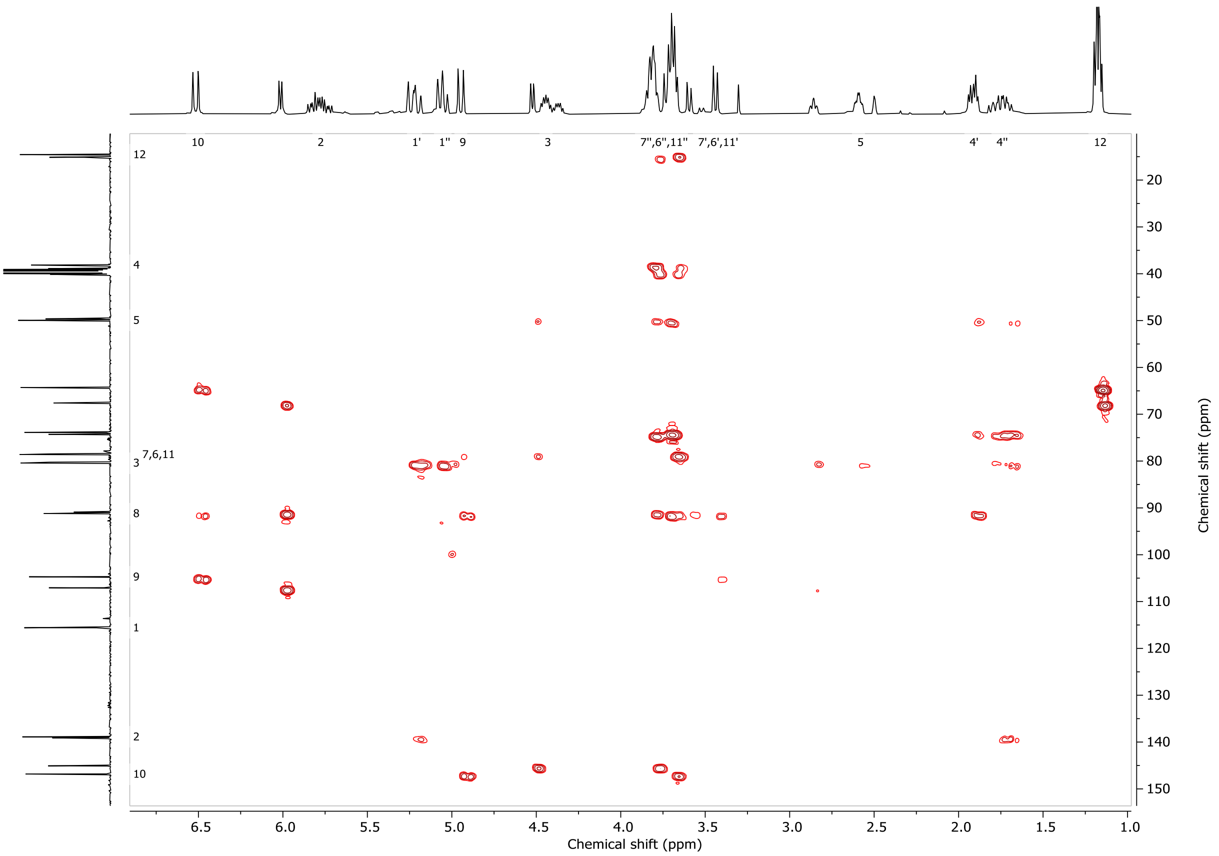

Figure S60:HMBC NMR spectrum of **qM2** (DMSO-D6).

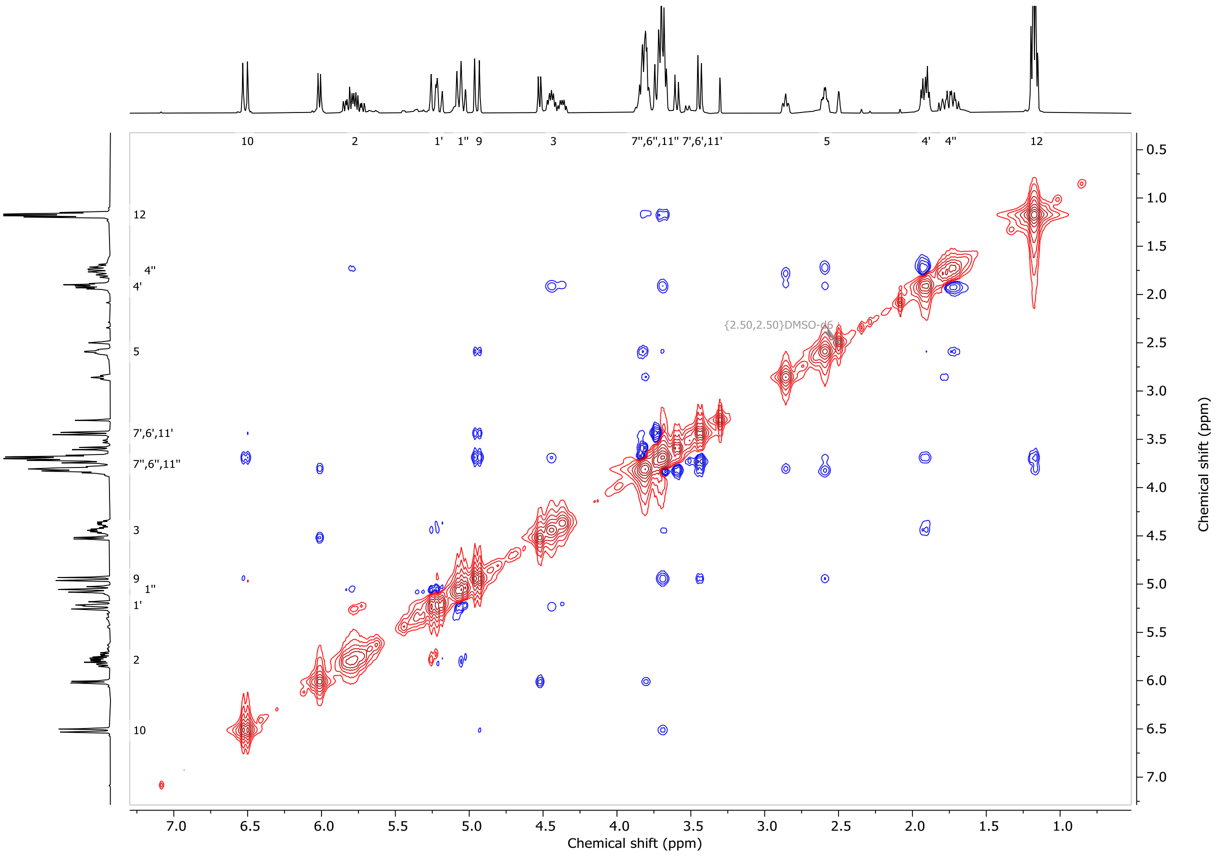

Figure S61: NOESY NMR spectrum of **qM2** (DMSO-D6). Positive phased signals (red) correspond to EXSY signals.

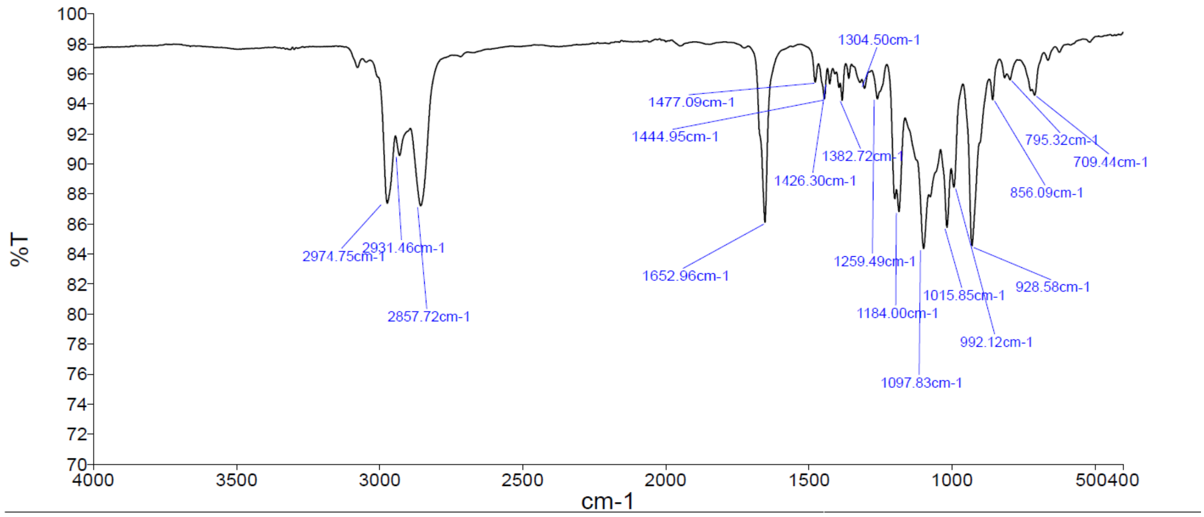

Figure S62: ATR-IR spectrum of **qM2**.

trans-**qM3**

^{1}H NMR (400 MHz, DMSO-D6) δ 6.68 (dd, *J* = 12.8, 1.2 Hz, 1H), 5.83 (ddd, *J* = 16.9, 10.4, 6.4 Hz, 1H), 5.27 (dd, *J* = 17.2, 1.6 Hz, 1H), 5.13 (dd, *J* = 10.5, 1.5 Hz, 1H), 4.96 (dd, *J* = 13.0, 1.3 Hz, 1H), 4.42 (t, *J* = 7.9 Hz, 1H), 4.36 (q, *J* = 6.9 Hz, 1H), 4.17 (dd, *J* = 9.7, 2.8 Hz, 1H), 3.76 (q, *J* = 6.9 Hz, 1H), 3.07 (tt, *J* = 7.7, 3.2 Hz, 1H), 2.15 – 2.04 (m, 1H), 2.03 – 1.91 (m, 1H), 1.20 (td, *J* = 7.0, 1.3 Hz, 3H) ppm

^{13}C{^{1}H} NMR (100 MHz, DMSO-D6) δ 175.77, 149.55, 137.56, 116.56, 100.49, 84.41, 79.84, 69.89, 64.80, 43.80, 40.15, 39.94, 39.73, 39.52, 39.31, 39.10, 38.90, 37.66, 14.48 ppm

1/λ (cm^{-1}): 3081 (w, v(**H–C**=$C_{terminal}$)), 2980 (m, v(**H–C**=$C_{terminal}$)), 2913 (w, v(C=**C–H**)), 2878 (w, v(C–**C–H**)), 1767 (s, v(C=O)), 1668 (m, v(C=**C–H**)), 1650 (m, v(C=**C–H**)), 1479 (w, v(CH_2)), 1447 (w, v(CH_2)), 1429 (w, δ(C–**C–H**)), 1379 (w, δ(C–**C–H**)), 1347 (w, δ(C–**C–H**)), 1300 (w, δ(**H-C**=C)), 1259 (w, δ(C–**C–H**)), 1202 (m, δ(C–**C–H**)), 1131 (m, δ(C–**C–H**)), 1112 (m, δ(C–**C–H**)), 984 (m, δ(C=**C–H**)), 930 (m, δ(C=**C–H**)), 852 (w, δ(C=**C–H**)), 796 (w, δ(C=O)), 689 (w, δ(C=**C–H**))

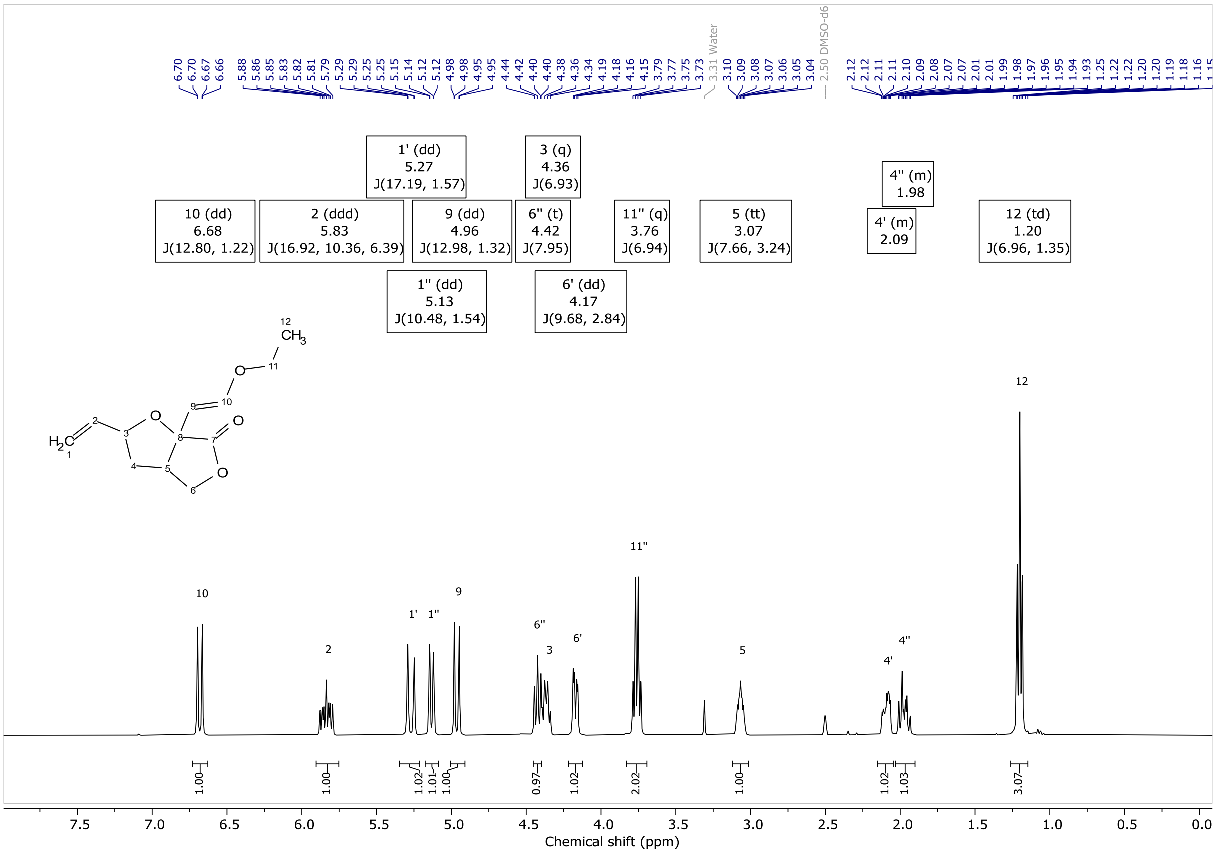

Figure S63: ^{1}H NMR spectrum of **qM3** (400 MHz, DMSO-D6).

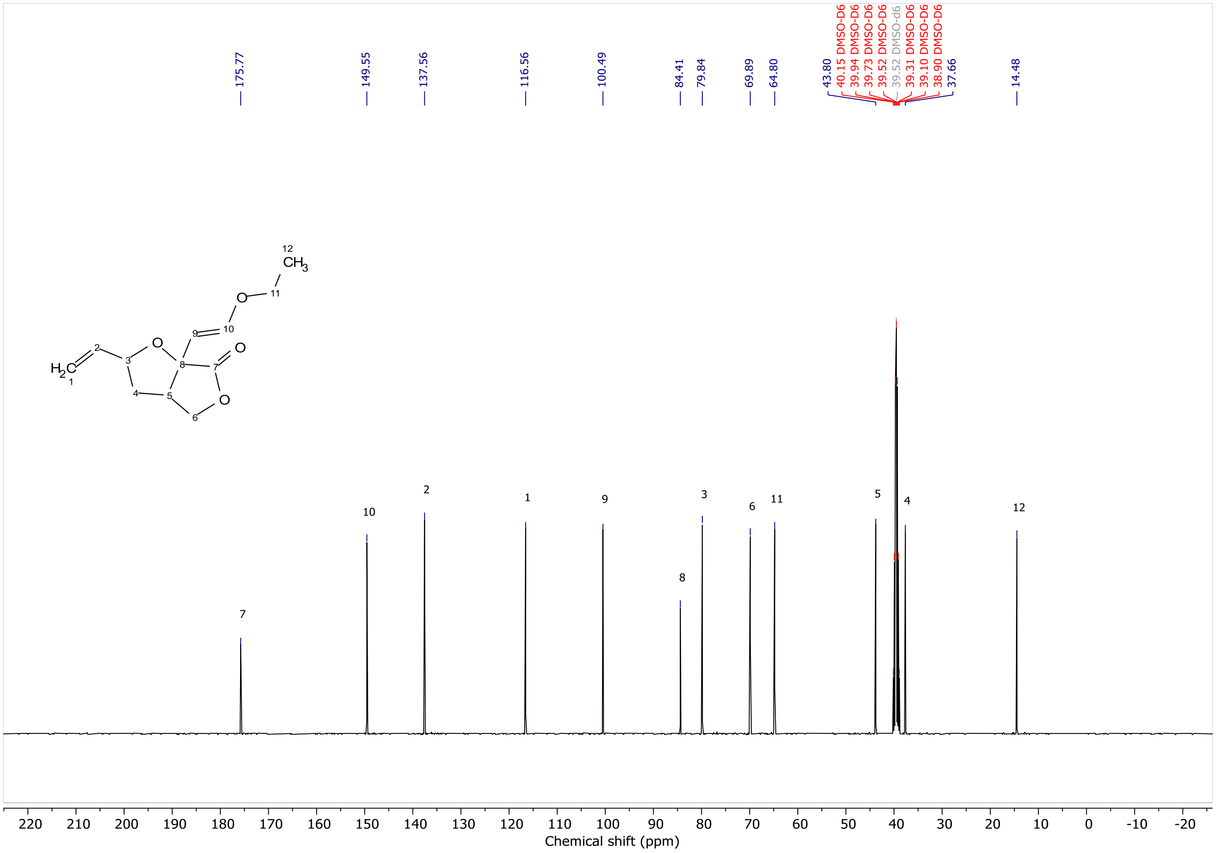

Figure S64: $^{13}C\{^{1}H\}$ NMR spectrum of **qM3** (100 MHz, DMSO-D6).

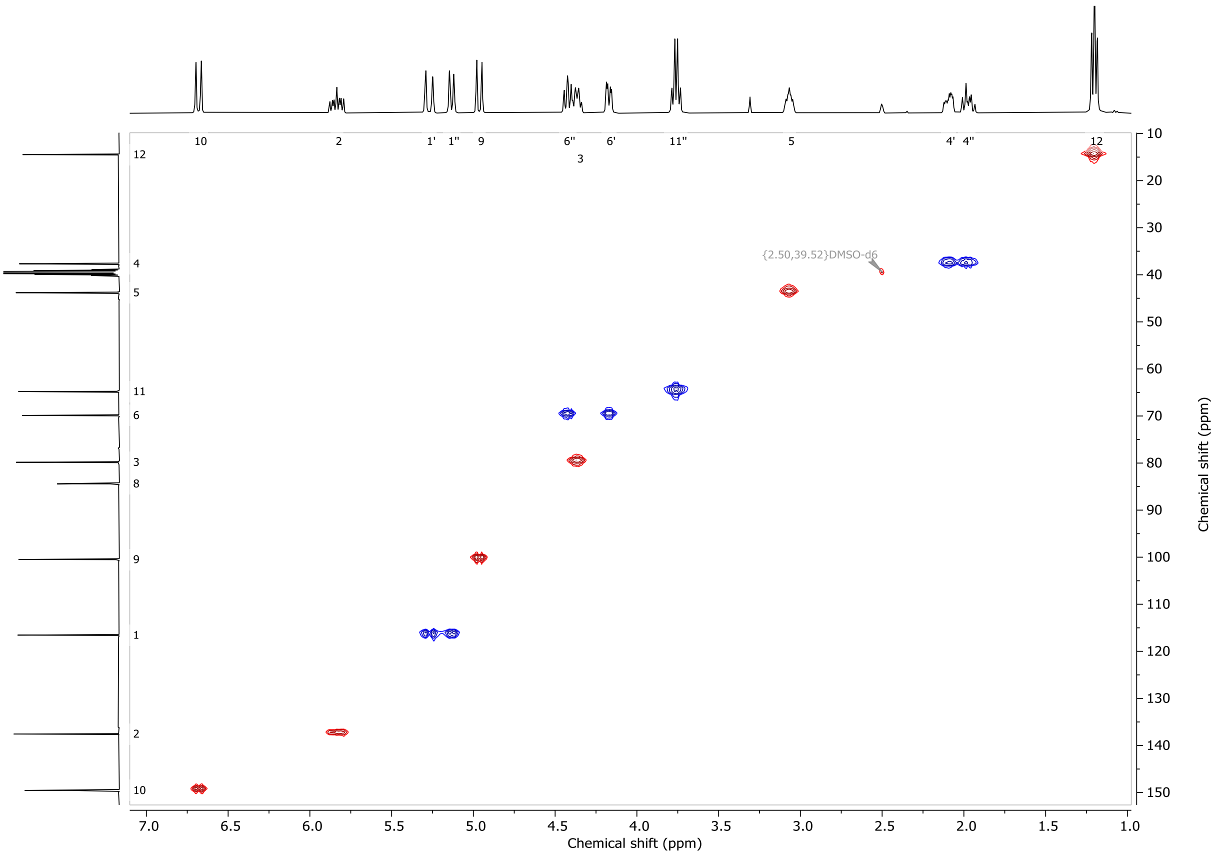

Figure S65: HSQC NMR spectrum of **qM3** (DMSO-D6).

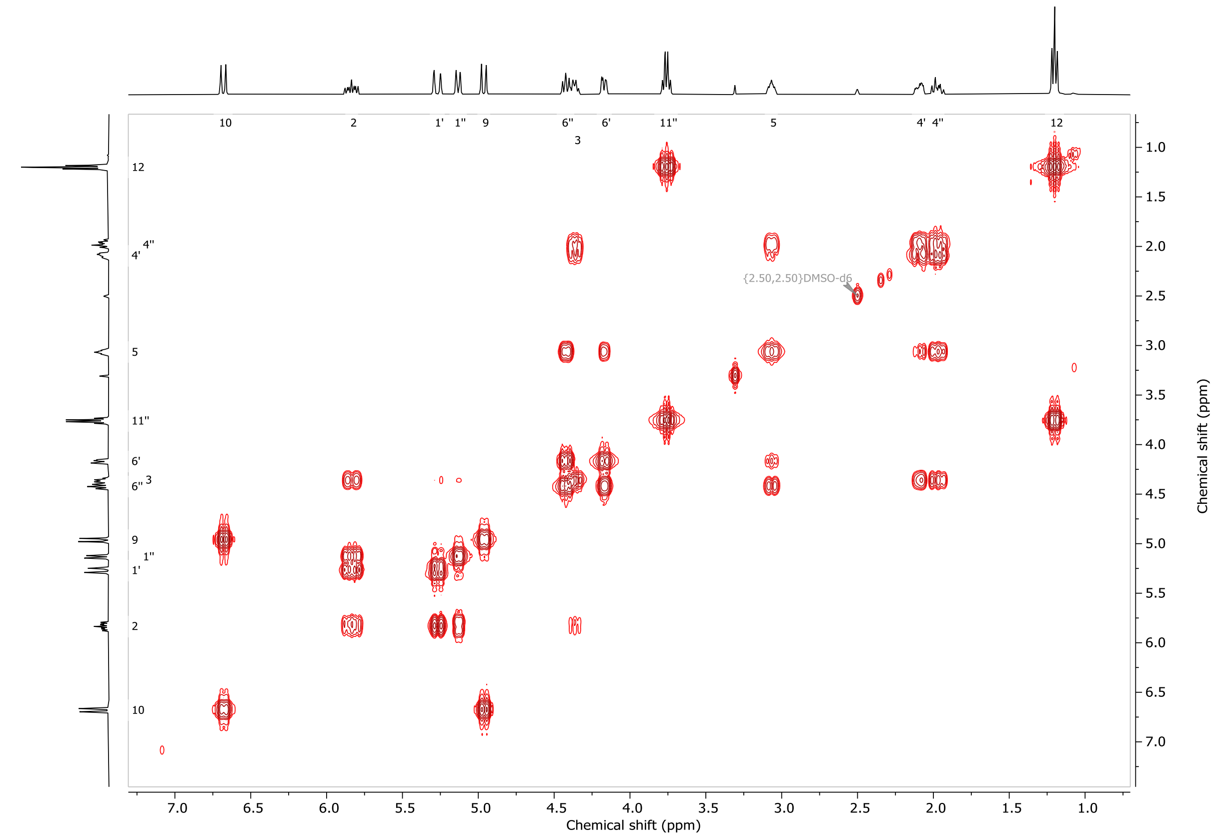

Figure S66: COSY NMR spectrum of **qM3** (DMSO-D6).

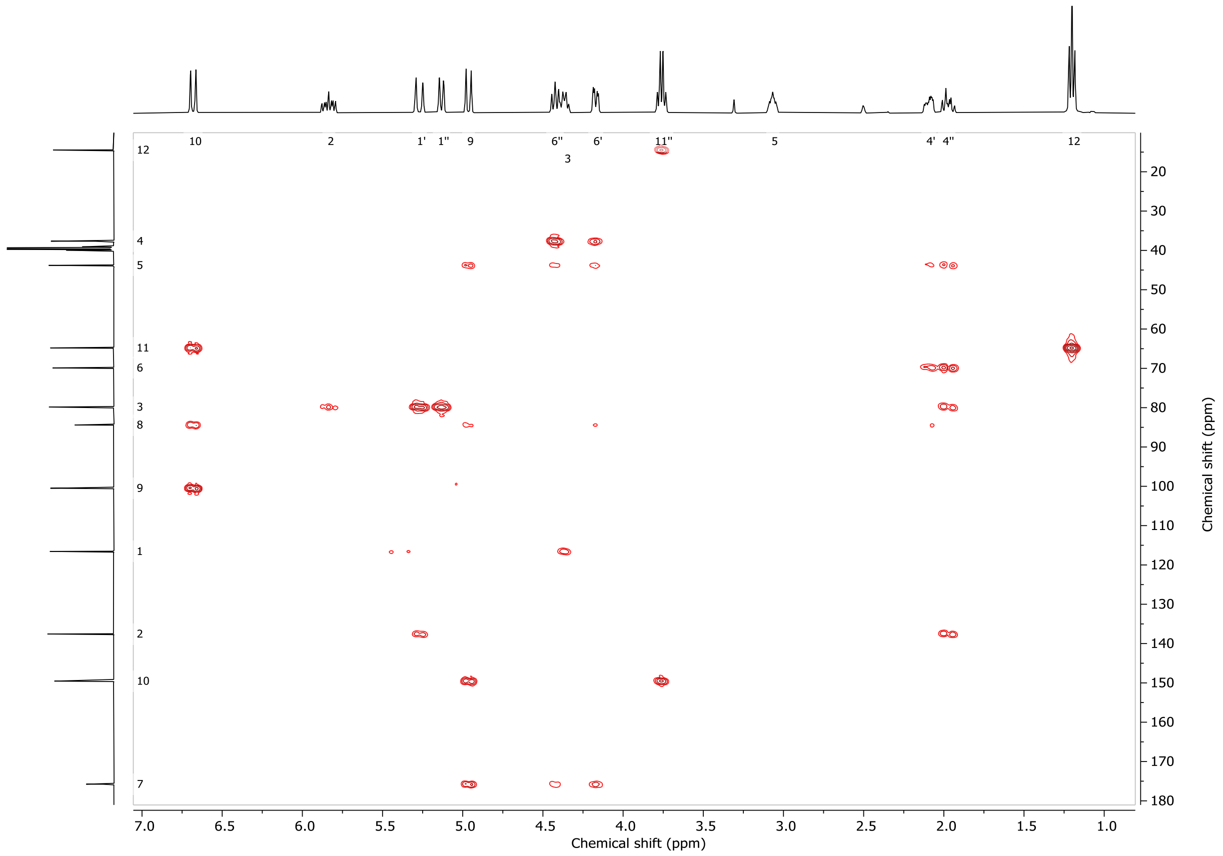

Figure S67: HMBC NMR spectrum of **qM3** (DMSO-D6).

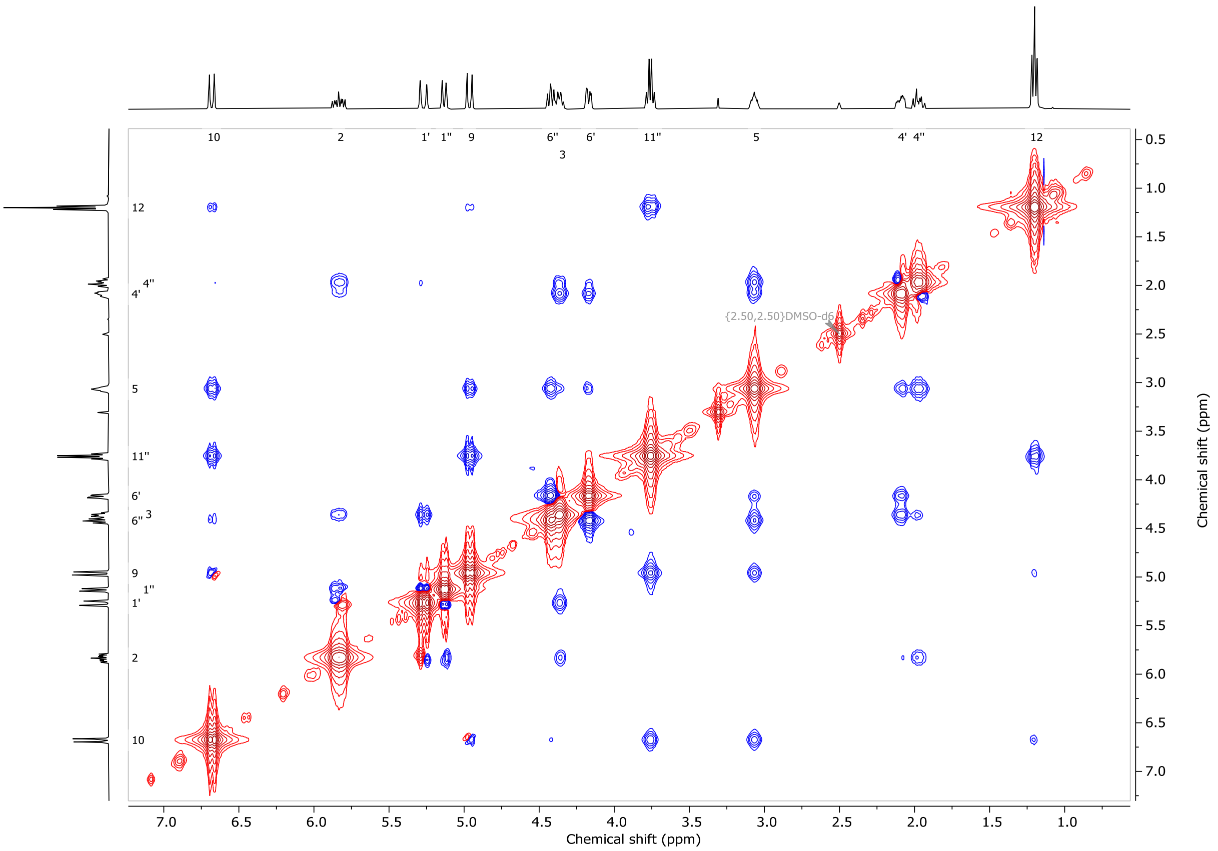

Figure S68: NOESY NMR spectrum of **qM3** (DMSO-D6).

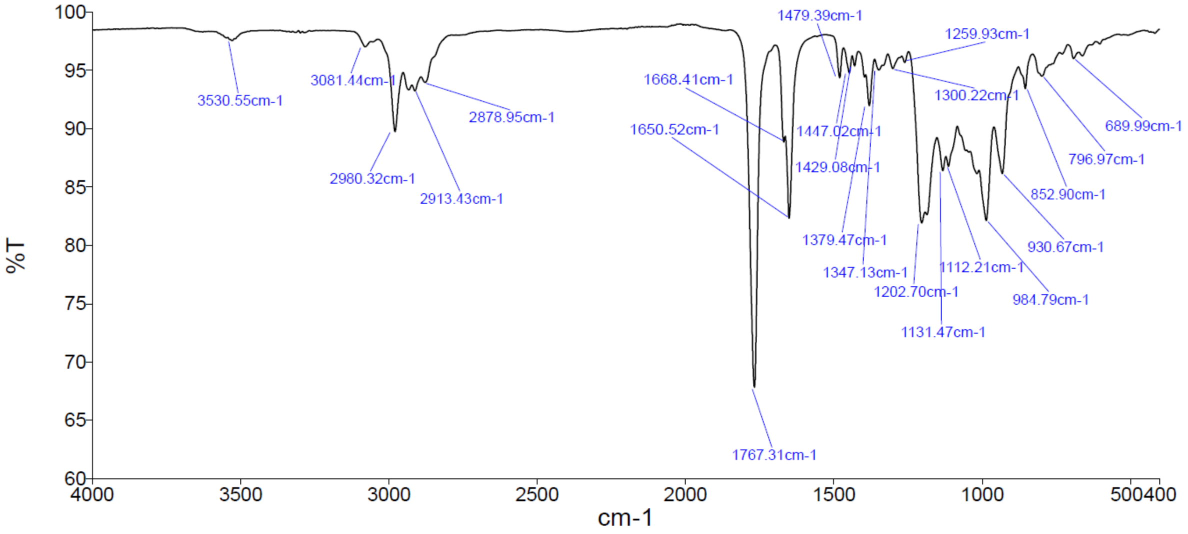

Figure S69: ATR-IR spectrum of **qM3**.

Immortal conditions

GIII (9.6 mg, 1 mol%) was dissolved in 13 mL DCM alongside 1 mL of a stock solution of ethyl vinyl ether in DCM (100 µL EVE in 10 mL DCM, 0.11 M, 10 eq.) and stirred for 5 min. **M1** (164.3 mg, 100 eq) was dissolved in 2 mL DCM, added to the other solution and stirred for 10 min. The reaction mixture was brought outside the glovebox and the volatiles removed under reduced pressure. The crude material was dissolved in 1,1,1,3,3,3-hexafluoroisopropanol (2 mL) and passed dropwise through a syringe filter into rigorously stirred MeOH (20 mL). The precipitated material was filtered off and dried in a vacuum oven set to 50 °C to ensure quantitative evaporation of EVE. The resulting polymer was isolated as an off-white powder.

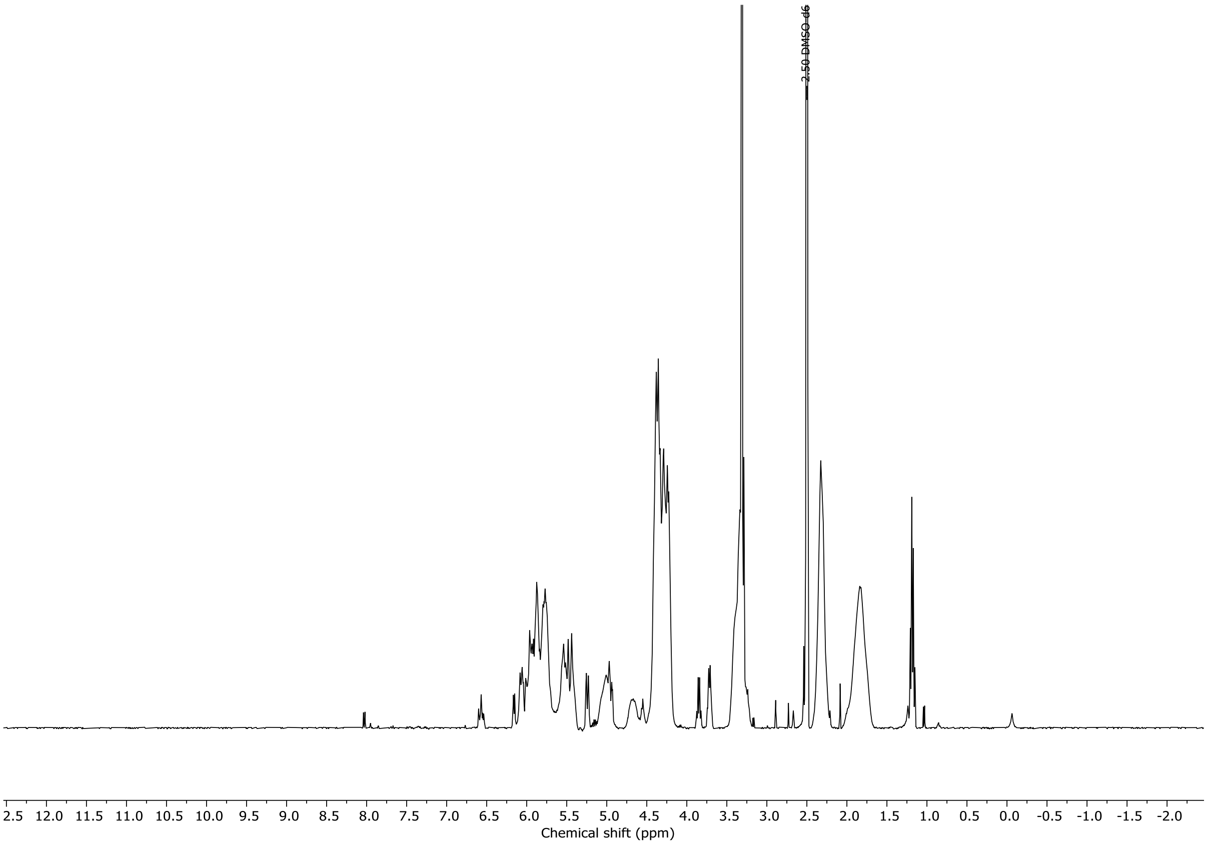

Figure S70: ^{1}H NMR spectrum of **pM1** prepared under immortal conditions (400 MHz, DMSO-D6).

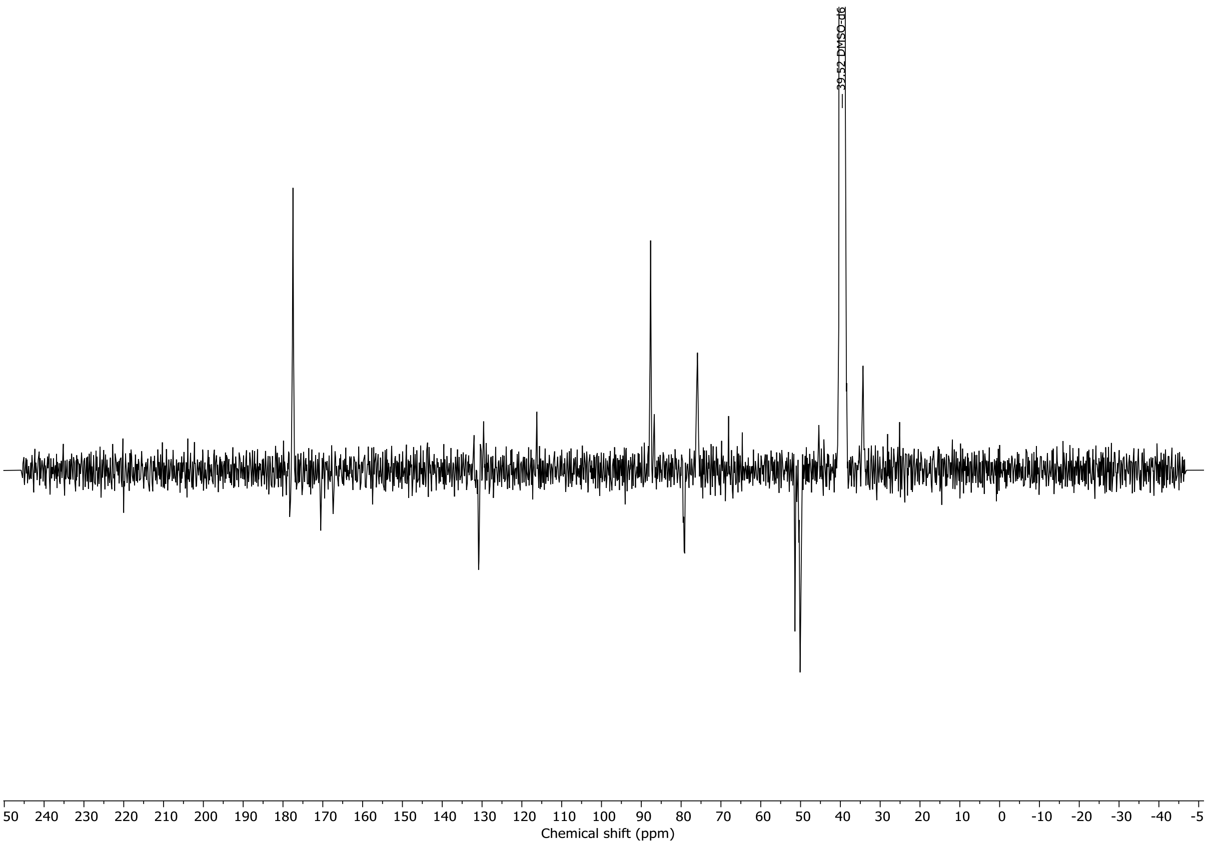

Figure S71: APT-$^{13}C\{^{1}H\}$ NMR spectrum of **pM1** prepared under immortal conditions (101 MHz, DMSO-D6).

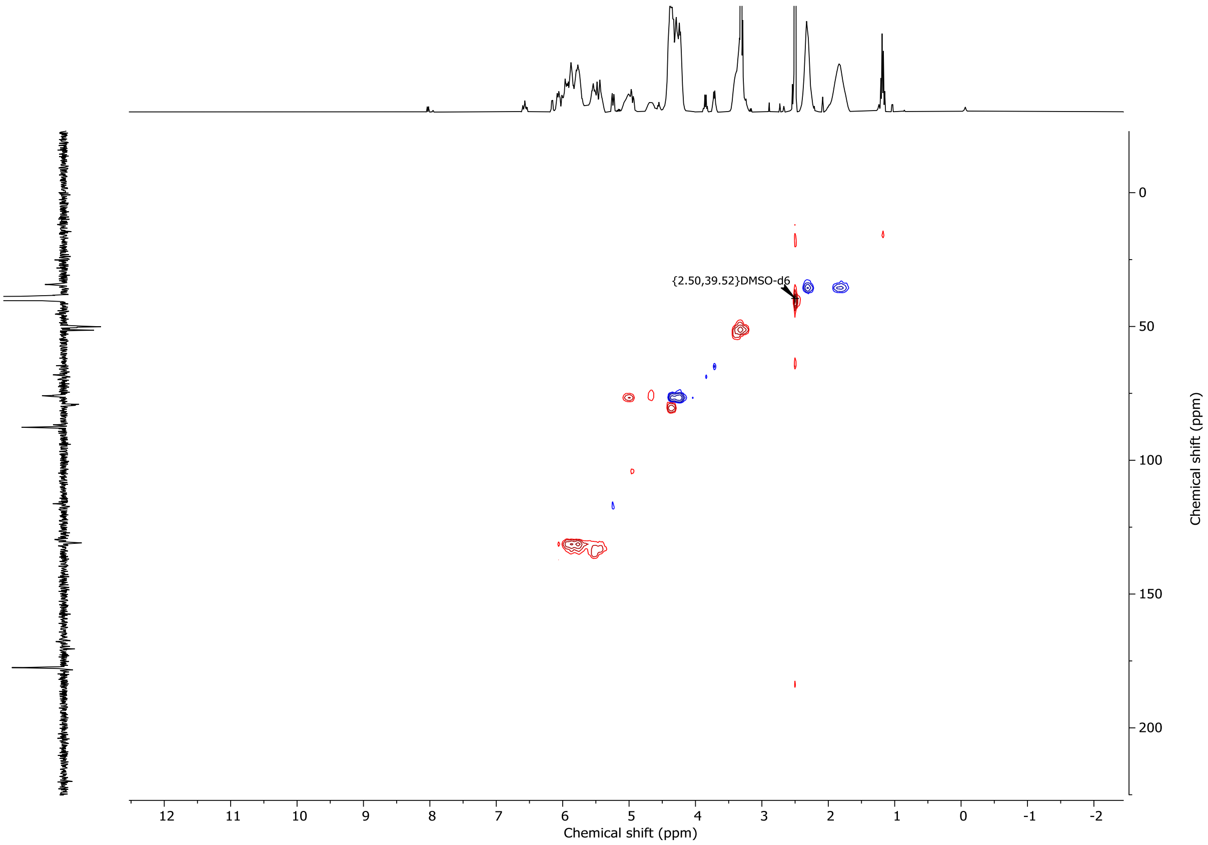

Figure S72: HSQC NMR spectrum of **pM1** prepared under immortal conditions (DMSO-D6).

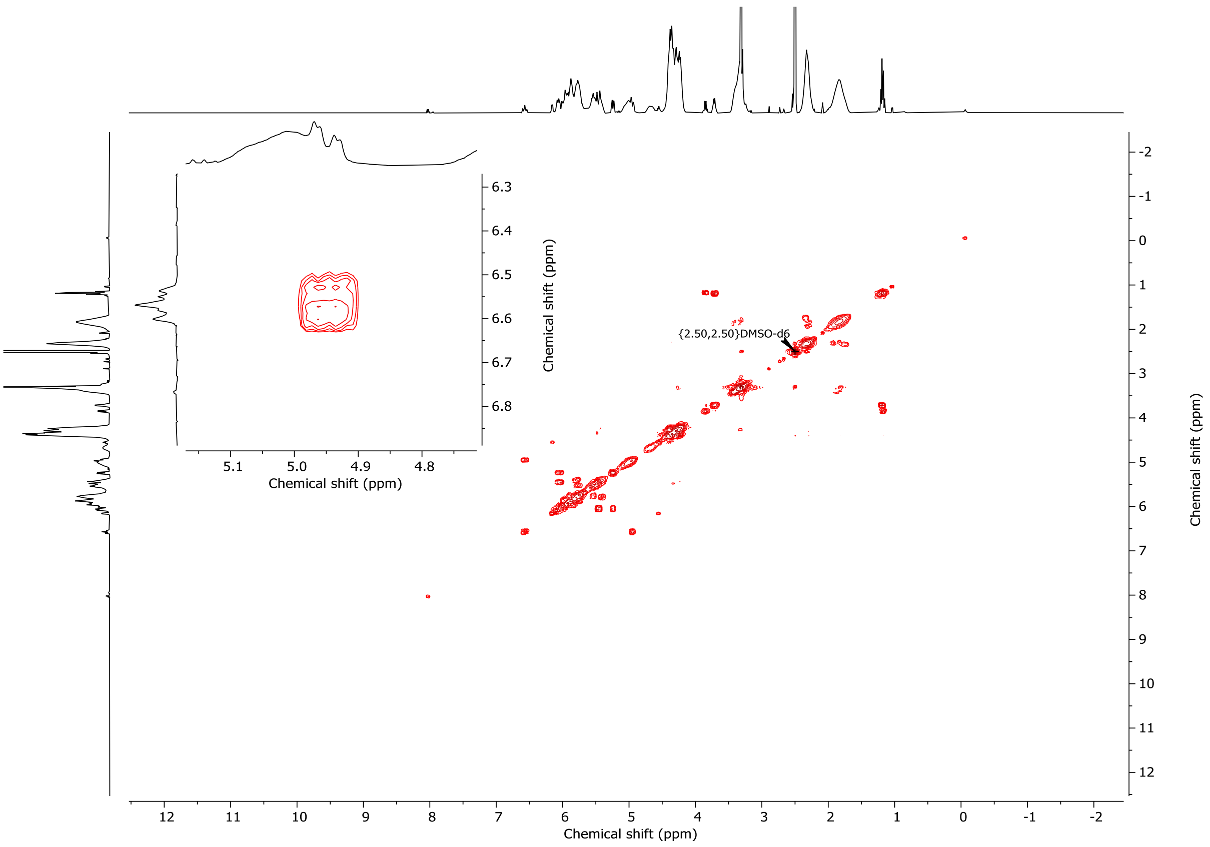

Figure S73: gCOSY NMR spectrum of **pM1** prepared under immortal conditions with highlighted region showing the crosspeak between EVE and alkene (DMSO-D6).

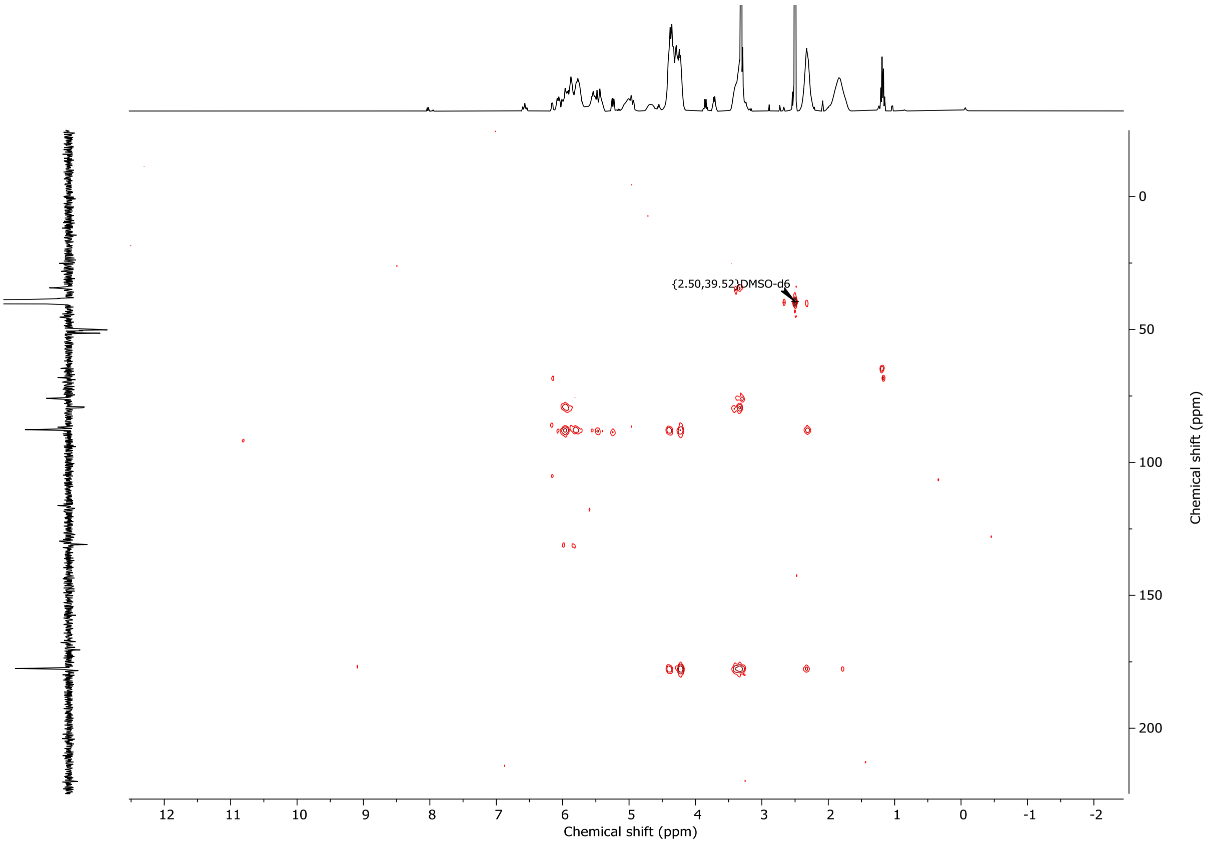

Figure S74: HMBC NMR spectrum spectrum of **pM1** prepared under immortal conditions (DMSO-D6).

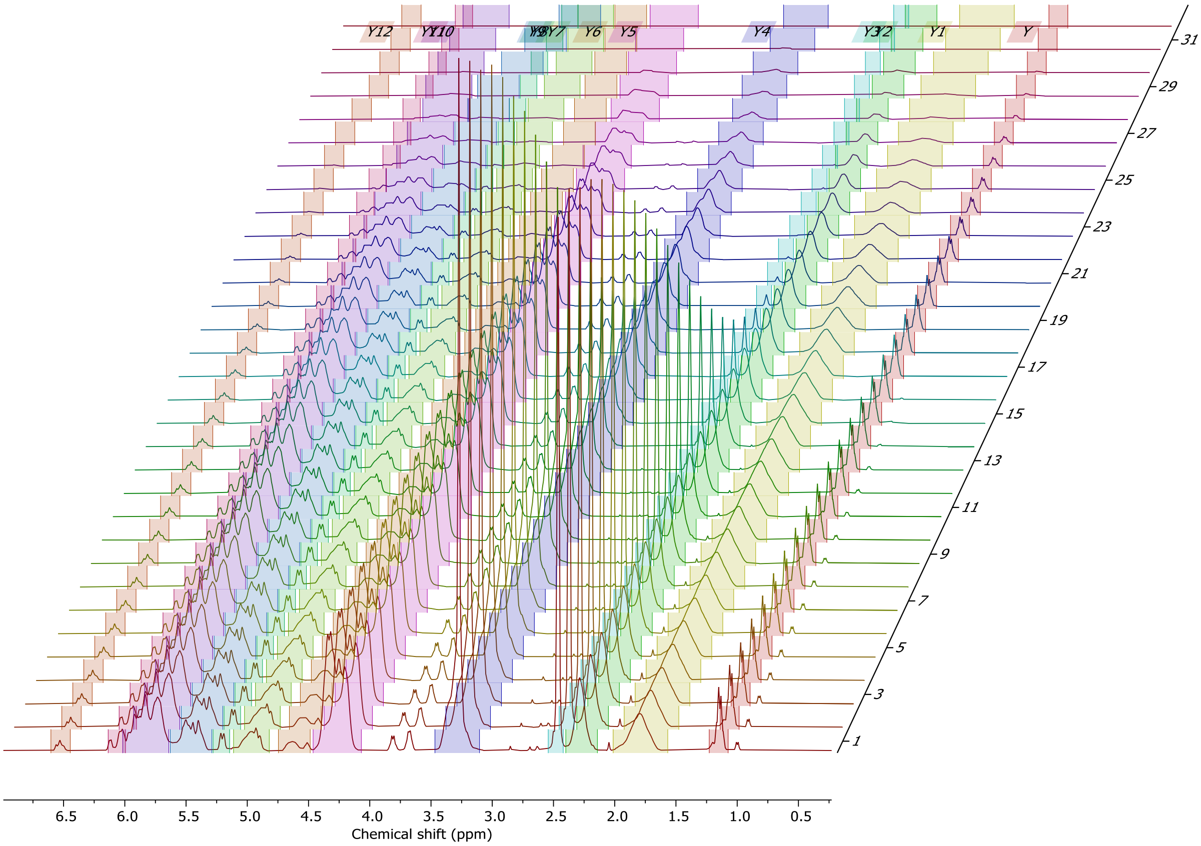

Figure S75: DOSY for **pM1** prepared under immortal conditions (32 spectra, δ 3 ms, Δ 150 ms, DMSO-d6, 400 MHz).

Table S1: Table of DOSY traces for **pM1** prepared under immortal conditions with applied regression.

#	X(I)	Y(X)	Y'(X)	Y1(X)	Y1'(X)	Y2(X)	Y2'(X)	Y3(X)	Y3'(X)	Y4(X)	Y4'(X)	Y5(X)	Y5'(X)
	ARR_DATA(I)	Integral(1.231,1.073)	B*exp(-x*F)	Integral(2.013,1.513)	B*exp(-x*F)	Integral(2.443,2.140)	B*exp(-x*F)	Integral(2.550,2.37	B*exp(-x*F)	Integral(3.479,3.033)	B*exp(-x*F)	Integral(4.517,4.053)	B*exp(-x*F)
Model			B= 0.523484		B= 1.11264		B= 1.22729		B= 2.49638		B= 3.51623		B= 3.72996
			F= 1.0266e-10		F= 8.65837e-11		F= 9.00483e-11		F= 5.69903e-10		F= 2.13635e-10		F= 8.72539e-11
1	239931788.7	0.523	0.511	1.097	1.09	1.2	1.201	2.129	2.177	3.675	3.341	3.632	3.653
2	286928061.5	0.51	0.508	1.096	1.085	1.212	1.196	2.154	2.12	3.577	3.307	3.68	3.638
3	343129959.7	0.506	0.505	1.084	1.08	1.197	1.19	2.057	2.053	3.467	3.268	3.615	3.62
4	410340034.7	0.506	0.502	1.074	1.074	1.187	1.183	1.986	1.976	3.403	3.221	3.647	3.599
5	490713372.6	0.501	0.498	1.067	1.066	1.188	1.174	1.901	1.887	3.281	3.166	3.57	3.574
6	586831646.7	0.496	0.493	1.062	1.058	1.176	1.164	1.794	1.787	3.166	3.102	3.534	3.544
7	701776569	0.488	0.487	1.047	1.047	1.159	1.152	1.677	1.673	3.012	3.027	3.54	3.508
8	839234643.3	0.485	0.48	1.032	1.035	1.15	1.138	1.547	1.547	2.865	2.939	3.473	3.467
9	1003616641	0.474	0.472	1.025	1.02	1.127	1.121	1.403	1.409	2.694	2.838	3.445	3.417
10	1200208410	0.464	0.463	1.013	1.003	1.103	1.102	1.253	1.26	2.524	2.721	3.368	3.359
11	1435280471	0.447	0.452	0.977	0.983	1.075	1.078	1.09	1.102	2.35	2.588	3.315	3.291
12	1716425497	0.437	0.439	0.958	0.959	1.047	1.052	0.931	0.939	2.16	2.437	3.183	3.211
13	2052626052	0.42	0.424	0.923	0.931	1.013	1.02	0.767	0.775	1.973	2.268	3.101	3.118
14	2454690802	0.402	0.407	0.891	0.9	0.966	0.984	0.608	0.616	1.804	2.081	2.986	3.011
15	2935470623	0.38	0.387	0.861	0.863	0.924	0.942	0.467	0.469	1.638	1.878	2.863	2.887
16	3510462155	0.361	0.365	0.811	0.821	0.88	0.895	0.345	0.338	1.493	1.661	2.72	2.746
17	4198083398	0.334	0.34	0.771	0.774	0.825	0.841	0.239	0.228	1.331	1.434	2.55	2.586
18	5020385649	0.307	0.313	0.719	0.72	0.766	0.781	0.156	0.143	1.218	1.203	2.382	2.407
19	6003718401	0.276	0.283	0.659	0.662	0.7	0.715	0.098	0.082	1.096	0.975	2.185	2.209
20	7179710034	0.24	0.25	0.583	0.598	0.631	0.643	0.06	0.042	0.982	0.758	1.981	1.994
21	8586026509	0.213	0.217	0.513	0.529	0.558	0.566	0.034	0.019	0.86	0.562	1.748	1.763
22	10267790657	0.186	0.182	0.455	0.457	0.488	0.487	0.02	0.007	0.748	0.392	1.523	1.523
23	12278954224	0.151	0.148	0.388	0.384	0.409	0.406	0.014	0.002	0.632	0.255	1.274	1.278
24	14684039872	0.124	0.116	0.322	0.312	0.342	0.327	0.012	0.001	0.525	0.153	1.066	1.036
25	17560297957	0.097	0.086	0.255	0.243	0.268	0.252	0.01	0	0.41	0.083	0.826	0.806
26	20999851677	0.07	0.061	0.192	0.181	0.204	0.185	0.01	0	0.319	0.04	0.634	0.597
27	25113190281	0.049	0.04	0.146	0.126	0.144	0.128	0.009	0	0.229	0.016	0.45	0.417
28	30032154961	0.033	0.024	0.088	0.083	0.105	0.082	0.01	0	0.161	0.006	0.31	0.271
29	35914646933	0.021	0.013	0.065	0.05	0.071	0.048	0.009	0	0.109	0.002	0.217	0.162
30	42949407033	0.016	0.006	0.001	0.027	0.042	0.026	0.002	0	0.068	0	0.052	0.088
31	51362119413	0.006	0.003	0.001	0.013	0.003	0.012	0.004	0	0.03	0	0.002	0.042
32	61422537900	0	0.001	-0.002	0.005	-0.001	0.005	0.002	0	0.002	0	0.001	0.018

#	Y6(X)	Y6'(X)	Y7(X)	Y7'(X)	Y8(X)	Y8'(X)	Y9(X)	Y9'(X)	Y10(X)	Y10'(X)	Y11(X)	Y11'(X)	Y12(X)	Y12'(X)
	Integral(4.775,4.486)	B*exp(-x*F)	Integral(5.144,4.777)	B*exp(-x*F)	Integral(5.280,5.074)	B*exp(-x*F)	Integral(5.634,4.891)	B*exp(-x*F)	Integral(6.050,5.608)	B*exp(-x*F)	Integral(6.166,5.936)	B*exp(-x*F)	Integral(6.608,6.354)	B*exp(-x*F)
Model		B= 0.247507		B= 0.543657		B= 0.158397		B= 0.974028		B= 2.20279		B= 0.281328		B= 0.0946536
		F= 1.12413e-10		F= 9.21192e-11		F= 9.64046e-11		F= 8.7988e-11		F= 8.58176e-11		F= 9.5447e-11		F= 9.12688e-11
1	0.218	0.241	0.511	0.532	0.147	0.155	0.932	0.954	2.176	2.158	0.284	0.275	0.091	0.093
2	0.264	0.24	0.557	0.529	0.165	0.154	0.97	0.95	2.176	2.149	0.279	0.274	0.09	0.092
3	0.255	0.238	0.55	0.527	0.162	0.153	0.954	0.945	2.141	2.139	0.273	0.272	0.095	0.092
4	0.262	0.236	0.549	0.523	0.162	0.152	0.959	0.939	2.142	2.127	0.27	0.271	0.093	0.091
5	0.209	0.234	0.501	0.52	0.148	0.151	0.929	0.933	2.122	2.112	0.27	0.268	0.09	0.091
6	0.207	0.232	0.5	0.515	0.146	0.15	0.922	0.925	2.096	2.095	0.266	0.266	0.088	0.09
7	0.24	0.229	0.46	0.51	0.106	0.148	0.838	0.916	2.033	2.074	0.254	0.263	0.091	0.089
8	0.237	0.225	0.528	0.503	0.157	0.146	0.928	0.905	2.063	2.05	0.259	0.26	0.087	0.088
9	0.237	0.221	0.519	0.496	0.154	0.144	0.909	0.892	2.026	2.021	0.252	0.256	0.084	0.086
10	0.229	0.216	0.506	0.487	0.15	0.141	0.897	0.876	1.994	1.987	0.247	0.251	0.083	0.085
11	0.227	0.211	0.498	0.476	0.148	0.138	0.882	0.858	1.958	1.948	0.246	0.245	0.085	0.083
12	0.181	0.204	0.451	0.464	0.131	0.134	0.83	0.837	1.892	1.901	0.236	0.239	0.081	0.081
13	0.18	0.197	0.434	0.45	0.125	0.13	0.808	0.813	1.836	1.847	0.229	0.231	0.081	0.078
14	0.173	0.188	0.424	0.434	0.123	0.125	0.787	0.785	1.775	1.784	0.222	0.223	0.075	0.076
15	0.168	0.178	0.404	0.415	0.119	0.119	0.752	0.752	1.709	1.712	0.212	0.213	0.074	0.072
16	0.158	0.167	0.383	0.393	0.111	0.113	0.709	0.715	1.613	1.63	0.202	0.201	0.069	0.069
17	0.144	0.154	0.357	0.369	0.104	0.106	0.667	0.673	1.522	1.536	0.189	0.188	0.065	0.065
18	0.138	0.141	0.338	0.342	0.097	0.098	0.623	0.626	1.42	1.432	0.177	0.174	0.057	0.06
19	0.127	0.126	0.308	0.313	0.087	0.089	0.572	0.574	1.309	1.316	0.161	0.159	0.055	0.055
20	0.112	0.11	0.275	0.281	0.078	0.079	0.501	0.518	1.166	1.19	0.138	0.142	0.047	0.049
21	0.099	0.094	0.245	0.247	0.07	0.069	0.453	0.458	1.041	1.054	0.125	0.124	0.042	0.043
22	0.09	0.078	0.216	0.211	0.06	0.059	0.402	0.395	0.911	0.913	0.111	0.106	0.035	0.037
23	0.066	0.062	0.178	0.175	0.049	0.048	0.33	0.331	0.772	0.768	0.093	0.087	0.03	0.031
24	0.06	0.048	0.148	0.141	0.04	0.038	0.272	0.268	0.635	0.625	0.055	0.069	0.026	0.025
25	0.04	0.034	0.112	0.108	0.029	0.029	0.213	0.208	0.501	0.488	0.048	0.053	0.023	0.019
26	0.025	0.023	0.09	0.079	0.02	0.021	0.165	0.154	0.387	0.363	0.044	0.038	0.017	0.014
27	0.014	0.015	0.074	0.054	0.02	0.014	0.129	0.107	0.289	0.255	0.032	0.026	0.011	0.01
28	-0.005	0.008	0.045	0.034	0.008	0.009	0.075	0.069	0.193	0.167	0.02	0.016	0.006	0.006
29	0.001	0.004	0	0.02	0.004	0.005	0.012	0.041	0.114	0.101	0.009	0.009	0	0.004
30	-0.001	0.002	0.003	0.01	0	0.003	0.006	0.022	0.031	0.055	-0.001	0.005	0	0.002
31	0	0.001	0.002	0.005	0	0.001	-0.001	0.011	0.002	0.027	-0.001	0.002	0	0.001
32	0	0	0	0.002	0	0	0.001	0.004	0	0.011	-0.001	0.001	0	0

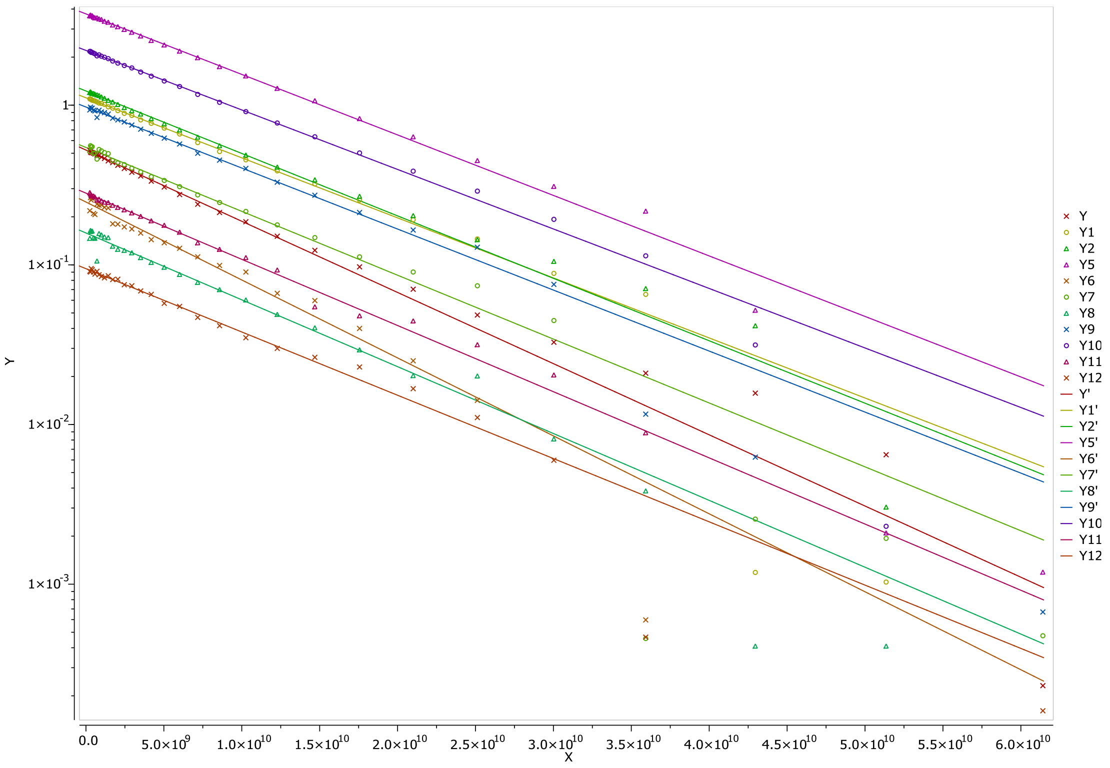

Figure S76:Logarithmic plot of integrals from DOSY data with monoexponential fits.

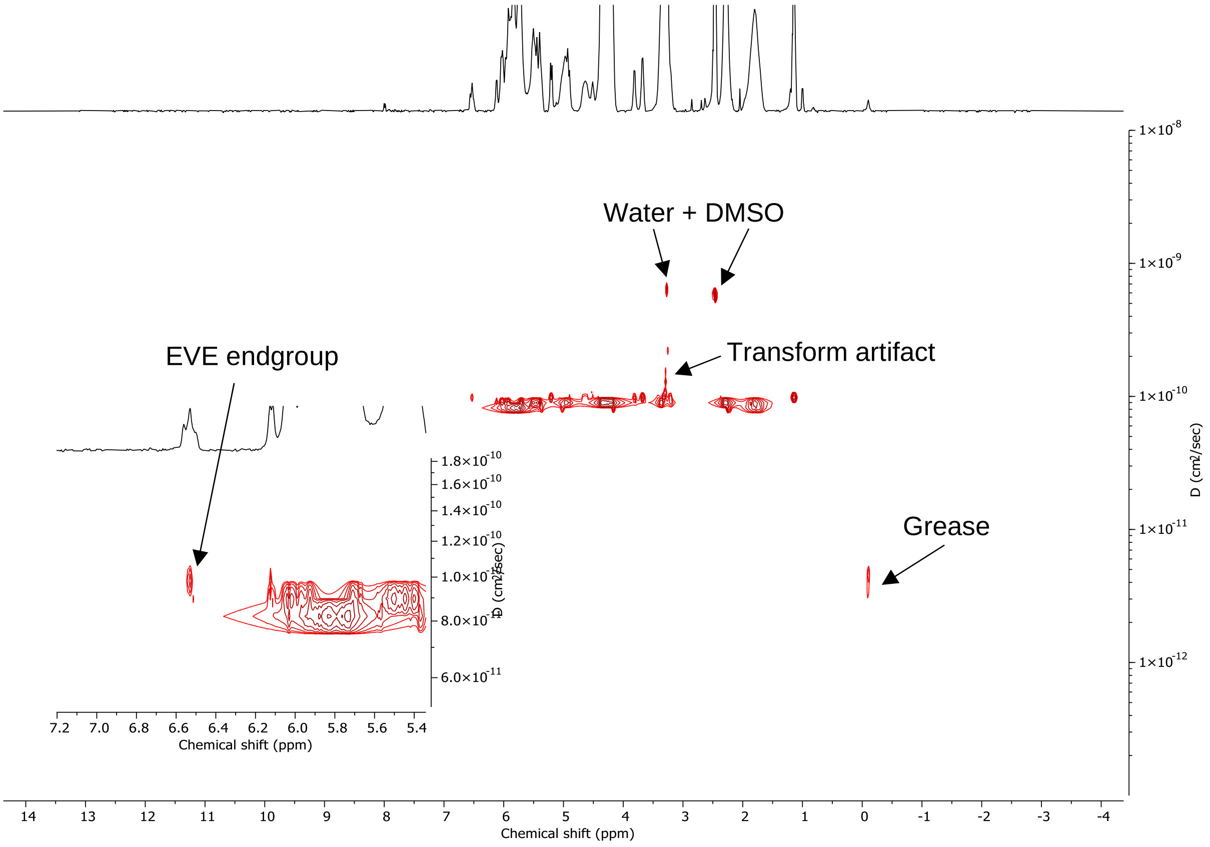

Figure S77: DOSY transform for **pM1** prepared under immortal conditions (Peak fit method, single decay component, 400 MHz DMSO-d6).

To further evaluate the connectivity of the EVE endgroup to the synthesized polymer, a DOSY NMR was taken of a sample that contained a mixture of telechelic **pM1** and an excess of **qM1** (see Figure S76).

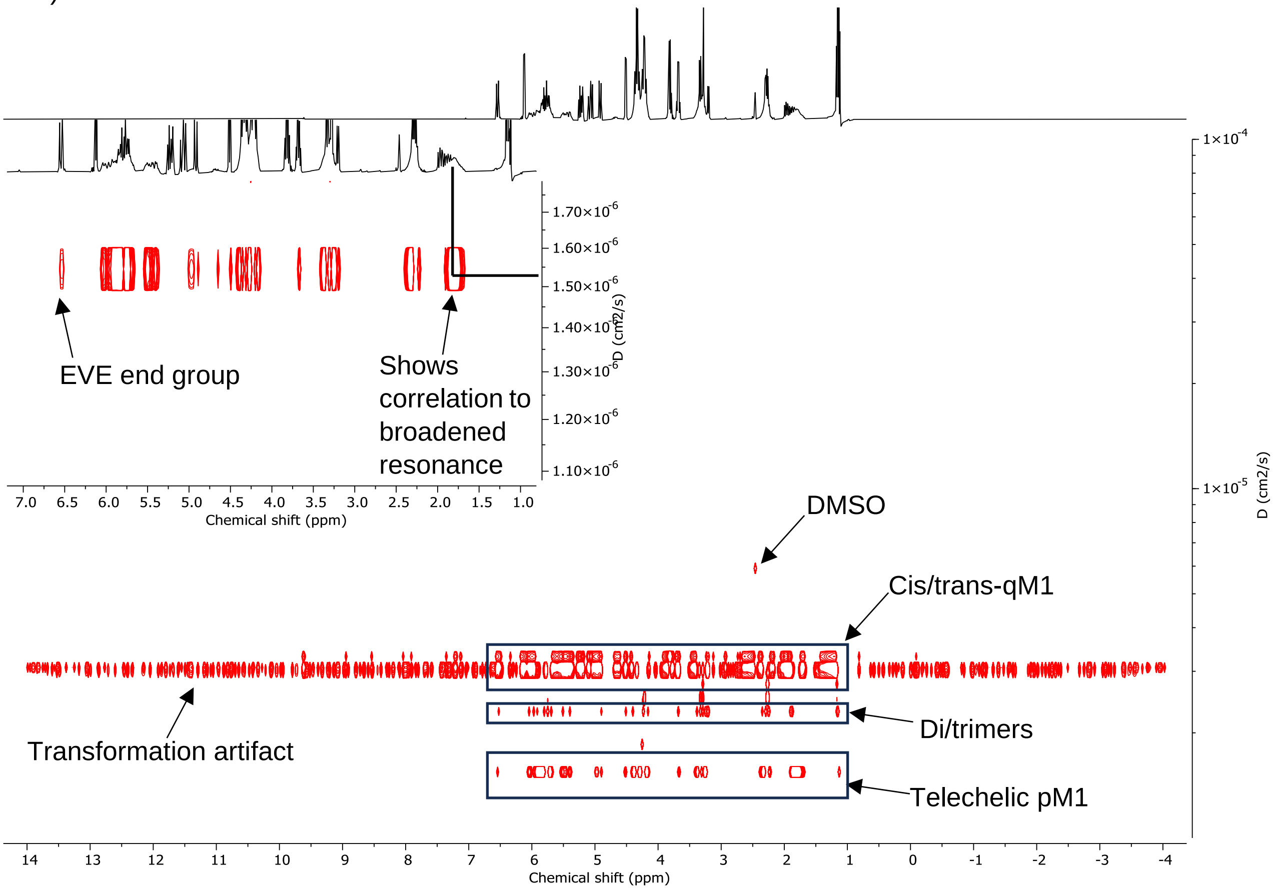

Figure S78: DOSY NMR spectrum of telechelic **pM1** with an excess of **qM1** (Bayesian transform, Resolution 10, Repetitions 5, 32 spectra, δ 2.5 ms, Δ 150 ms, DMSO-d6, 400 MHz).

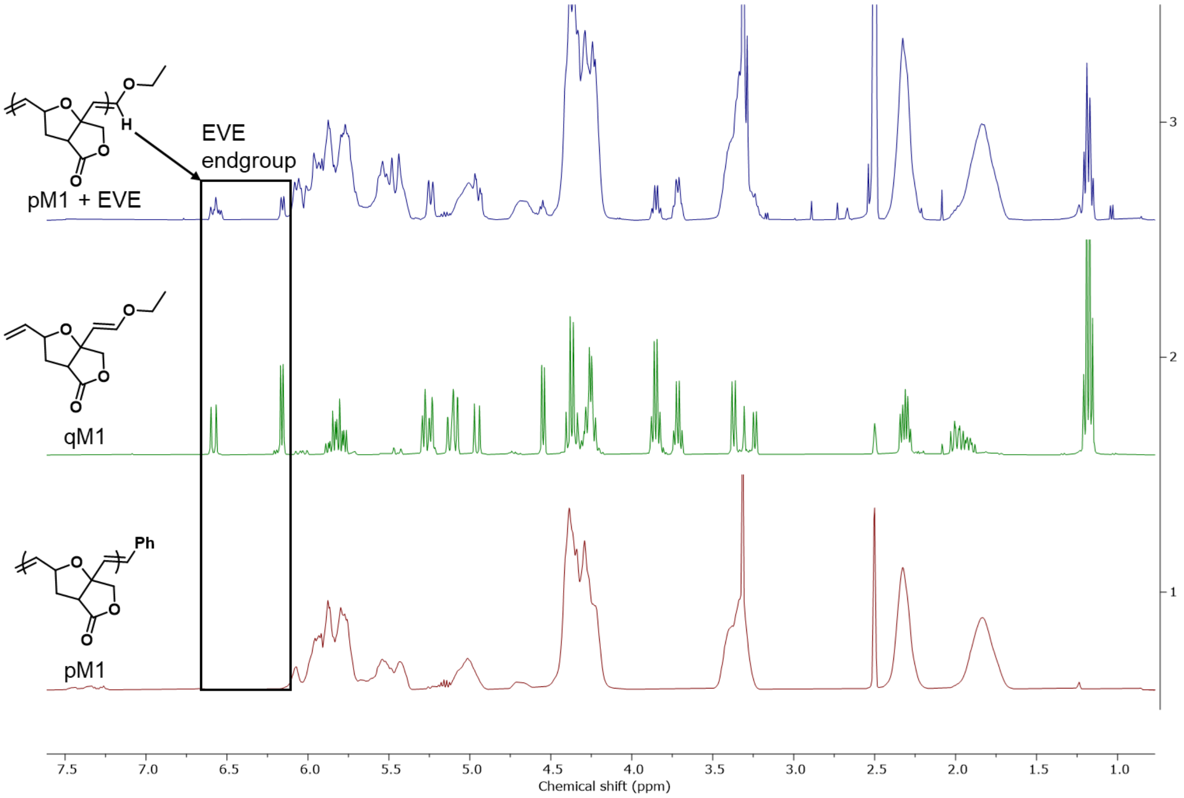

Figure S79: Comparison of ^{1}H NMR spectra of **pM1 + 10 eq EVE**, **qM1** and **pM1** to highlight the endgroup connectivity and change in peak shape.

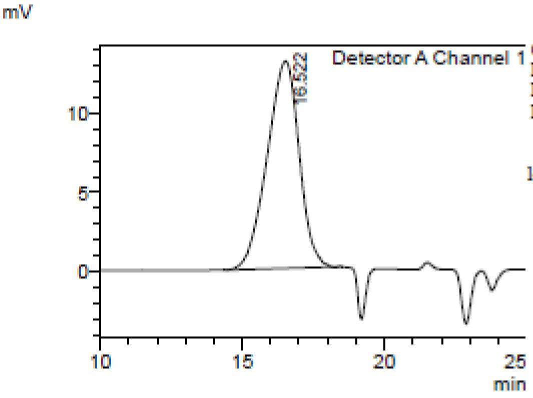

GPC Calibration Curve

Curve Fit Type : 3rd Order
R^2=0.9999814
Dispersion=0.002605724
Function : f(x)=-0.001314468*X^3+0.0634613
-1.309925*X+14.14415 (X=x-T.LIMI

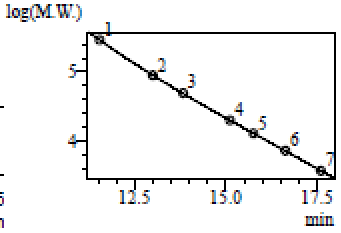

#	Molecular Weigh
1	288300
2	89050
3	49350
4	20020
5	13030
6	7350
7	3790

GPC Graph

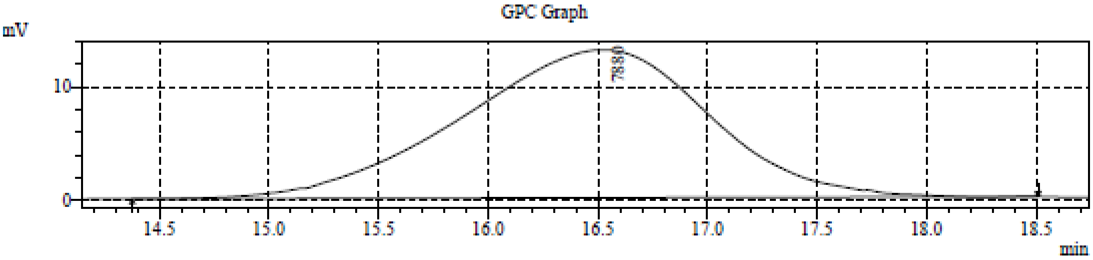

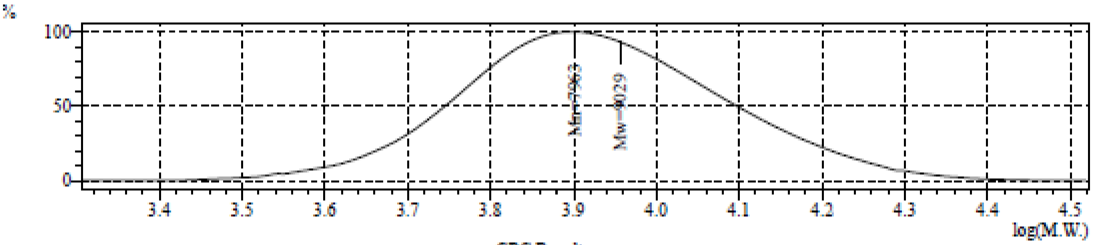

GPC Results

Peak#:1 (Detector A Channel 1)
Peak Information]

Title	Time(min)	Molecular Weig	Height
Start	14.375	33351	84
Top	16.522	7880	13111
End	18.508	2018	298

Area : 1029589

Average Molecular Weight]

Number Average Molecular Weight(Mn)	7963
Weight Average Molecular Weight(Mw)	9029
Mw/Mn	1.13398

Figure S80: GPC chromatogram of **pM1** prepared under immortal conditions with used calibration.

Chain transfer control
GIII (1.9 mg, 0.2 mol%) was dissolved in 13 mL DCM alongside a specific amount of a stock solution of ethyl vinyl ether in DCM and stirred for 5 min.

Stock solution 1 was prepared by adding 0.2 mL of EVE in 10 mL DCM. 1 mL of Stock solution 1 was then further diluted in 9 mL DCM and used as is seen in the table below.

Table S2: Specifics about stock solution used in the chain transfer control experiments.

Volume added of EVE stock solution	Equivalents in regards to **GIII**	$N_{monomer,theo}$ resulting from CT
0	0	500
0.1 mL	1	250
0.2 mL	2	125
0.5 mL	5	100
1.0 mL	10	50

M1 (164.3 mg, 500 eq) was dissolved in 2 mL DCM, added to the catalyst solution and stirred for 5 min. The reaction mixture was brought outside the glovebox and the volatiles removed under reduced pressure. The crude material was dissolved in 1,1,1,3,3,3-hexafluoroisopropanol (2 mL) and passed dropwise through a syringe filter into rigorously stirred MeOH (20 mL). The precipitated material was filtered off and dried in a vacuum oven set to 50 °C. The resulting polymer was isolated as an off-white powder.

Note: Due to the high volatility of DCM and EVE it is recommended to prepare these solutions fresh before use.

Chain end-analysis by ^{1}H NMR proved to be inconclusive since part of the endgroup gets exchanged by a vinyl ether linkage (see immortal conditions section). In the expansion in the ^{1}H NMR spectra below both endgroups are highlighted with the conventional Ph endgroup showing resonances between 7.4 ppm – 7.0 ppm and the EVE endgroup at 6.5 ppm.

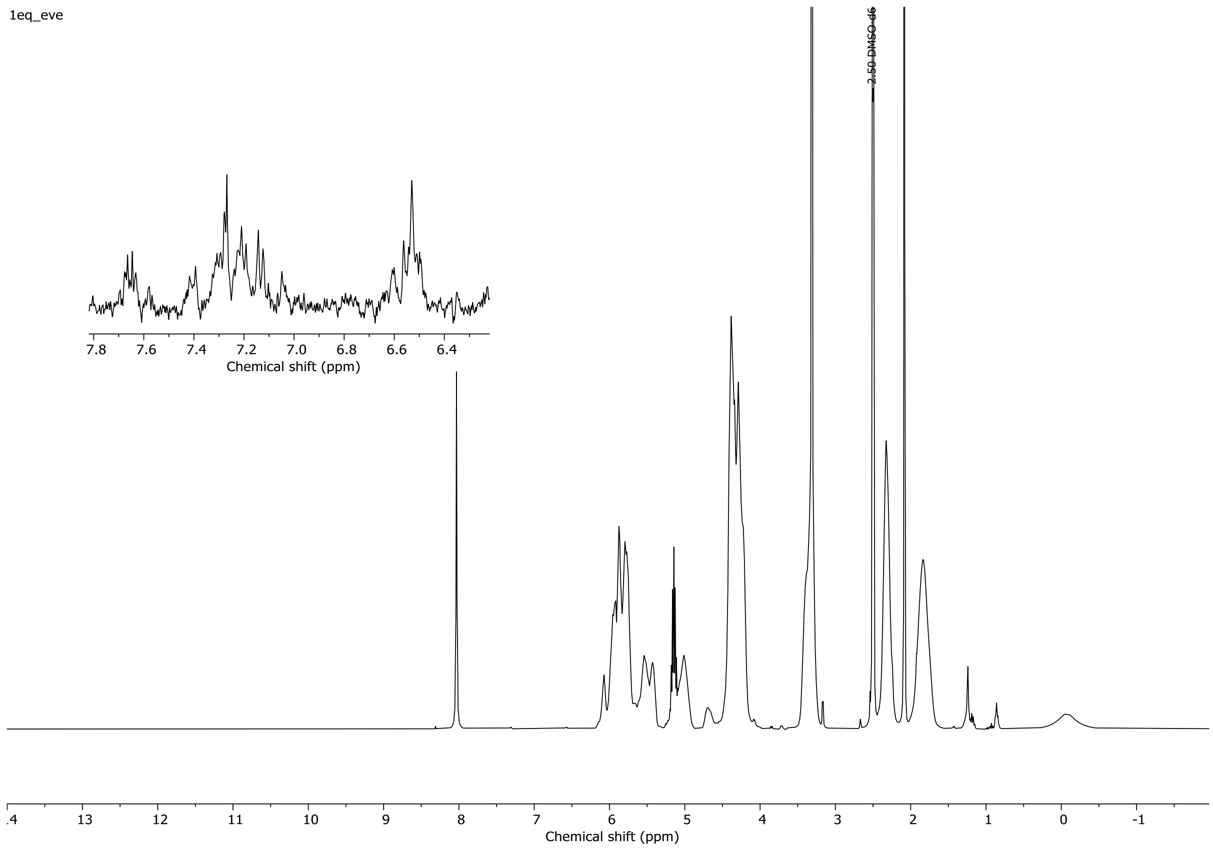

Figure S81: ^{1}H NMR spectrum of **pM1** made by 500 eq monomer in the presence of 1 eq EVE.

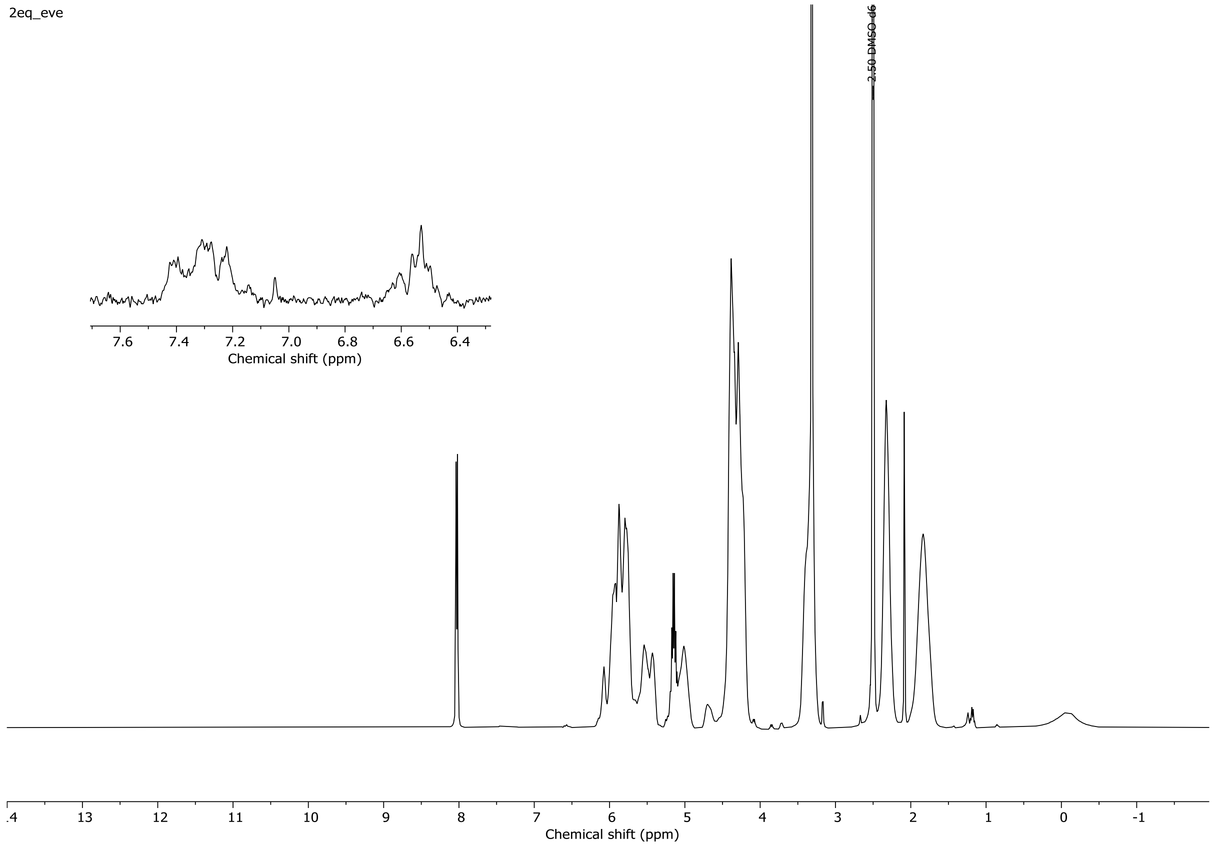

Figure S82: ^{1}H NMR spectrum of **pM1** made by 500 eq monomer in the presence of 2 eq EVE.

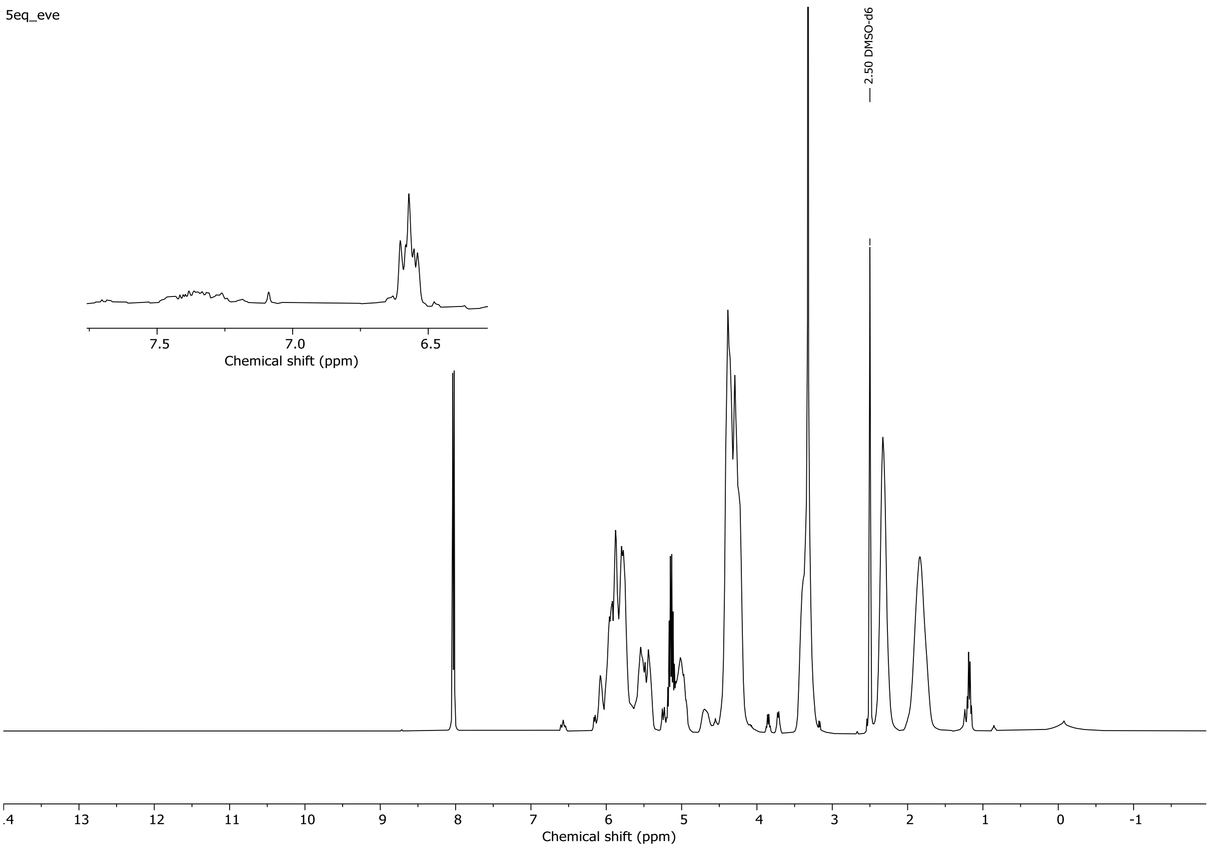

Figure S83: ^{1}H NMR spectrum of **pM1** made by 500 eq monomer in the presence of 5 eq EVE.

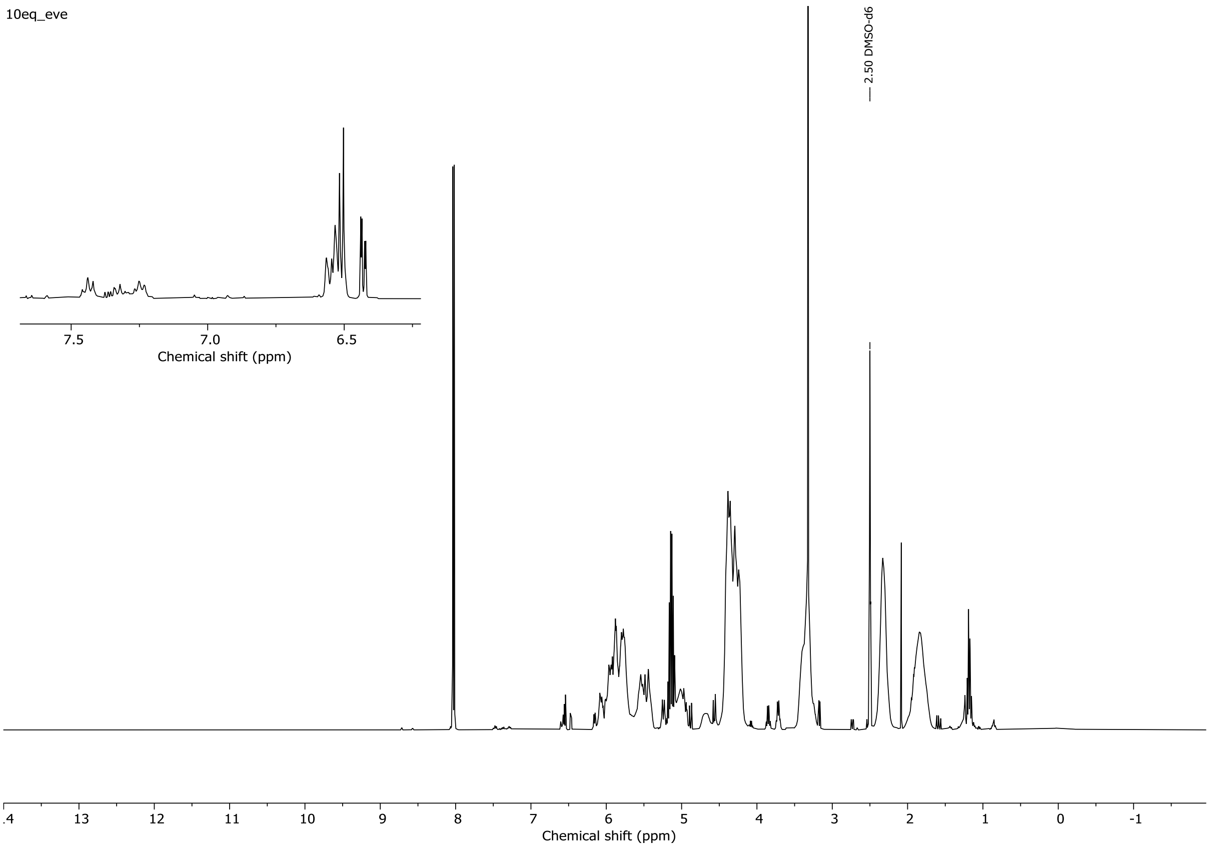

Figure S84: ^{1}H NMR spectrum of **pM1** made by 500 eq monomer in the presence of 10 eq EVE.

<Chromatogram>

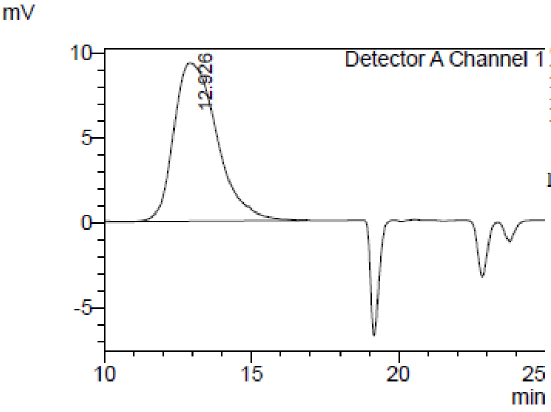

GPC Calibration Curve

Curve Fit Type : 3rd Order
R^2=0.9999813
Dispersion=0.002613652
Function : f(x)=-0.001304630*X^3+0.0640078
-1.331108*X+14.34273 (X=x-T.LIMI

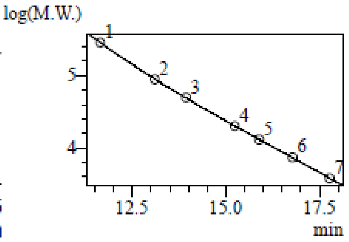

#	Iolecular Weigh
1	288300
2	89050
3	49350
4	20020
5	13030
6	7350
7	3790

GPC Graph

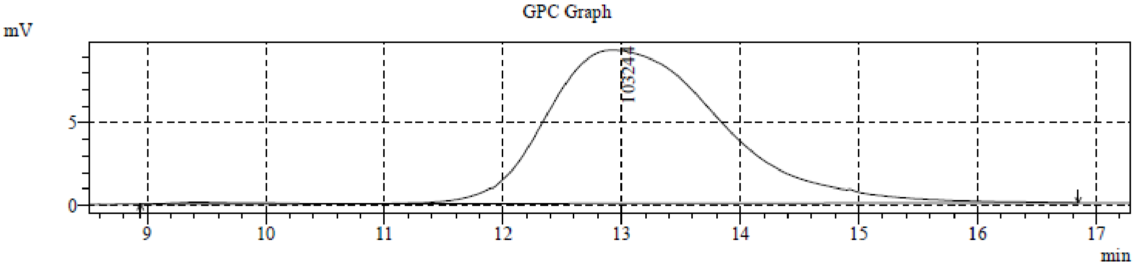

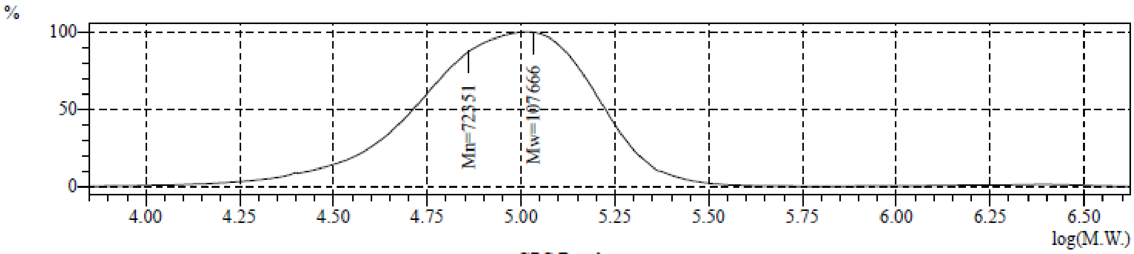

GPC Results

Peak#:1 (Detector A Channel 1)
[Peak Information]

Title	Time(min)	Iolecular Weigl	Height
Start	8.942	4220298	71
Top	12.926	103244	9308
End	16.850	7004	166

Area : 958713

[Average Molecular Weight]

Number Average Molecular Weight(Mn)	72351
Weight Average Molecular Weight(Mw)	107666
Mw/Mn	1.48811

Figure S85: GPC Chromatogram of **pM1** made by 500 eq monomer in the presence of 1 eq EVE.

<Chromatogram>

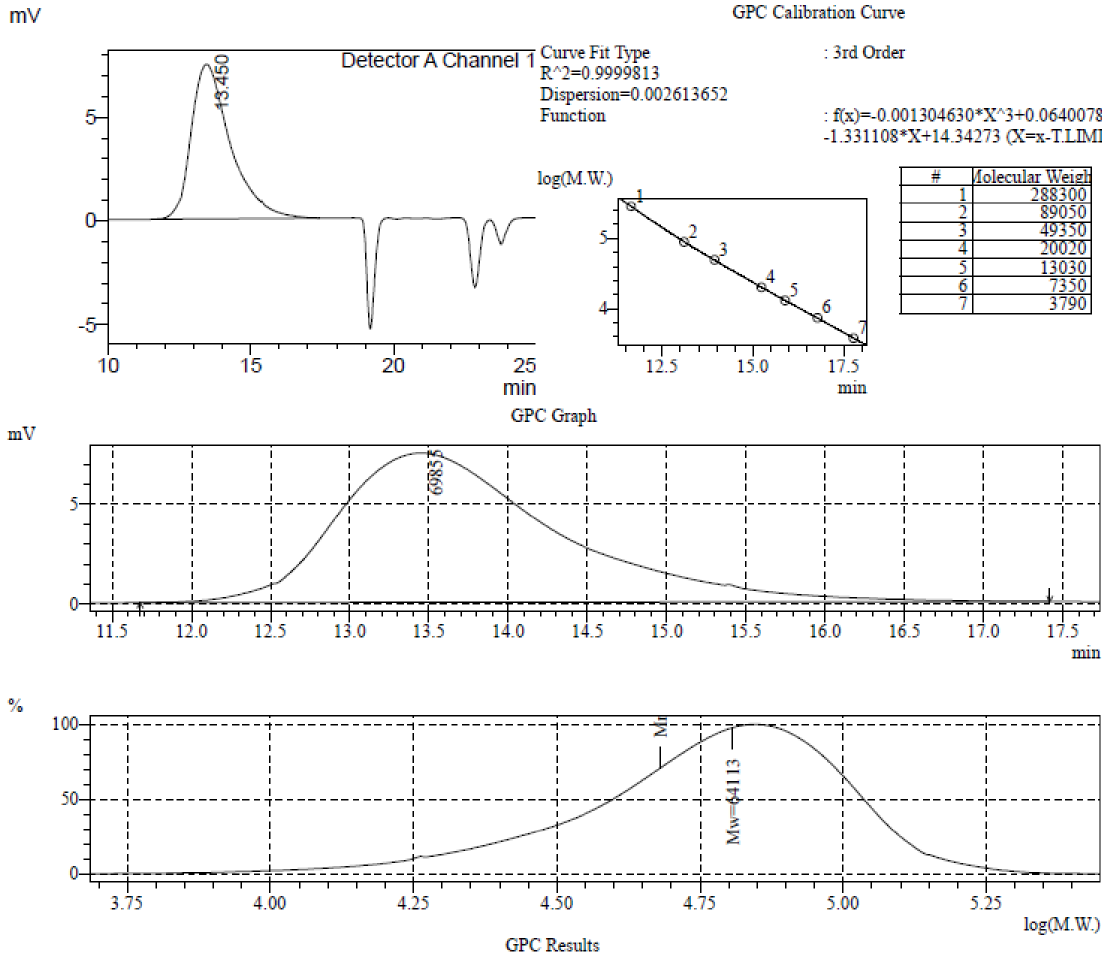

GPC Results

Peak#:1 (Detector A Channel 1)

[Peak Information]

Title	Time(min)	Molecular Weight	Height
Start	11.675	282175	80
Top	13.450	69855	7460
End	17.417	4817	134

Area : 715507

[Average Molecular Weight]

Number Average Molecular Weight(Mn)	47974
Weight Average Molecular Weight(Mw)	64113
Mw/Mn	1.33640

Figure S86: GPC Chromatogram of **pM1** made by 500 eq monomer in the presence of 2 eq EVE.

<Chromatogram>

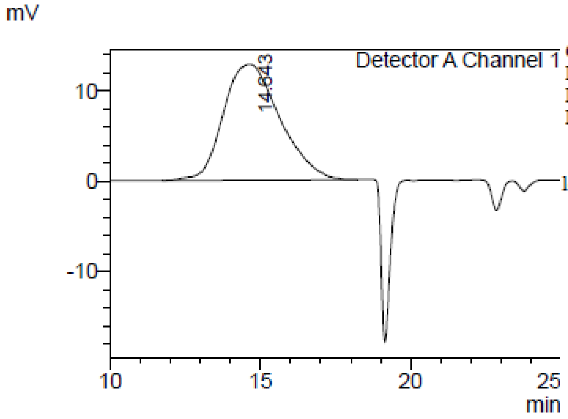

GPC Calibration Curve

Curve Fit Type : 3rd Order
R^2=0.9999813
Dispersion=0.002613652
Function : f(x)=-0.001304630*X^3+0.0640078
-1.331108*X+14.34273 (X=x-T.LIMI

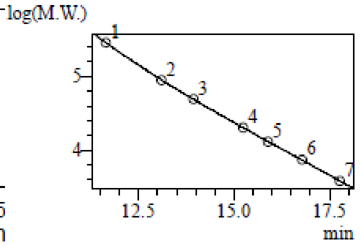

#	Molecular Weigh
1	288300
2	89050
3	49350
4	20020
5	13030
6	7350
7	3790

GPC Graph

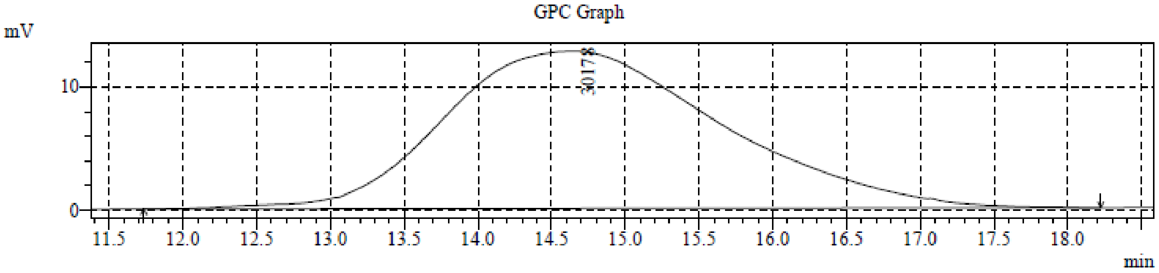

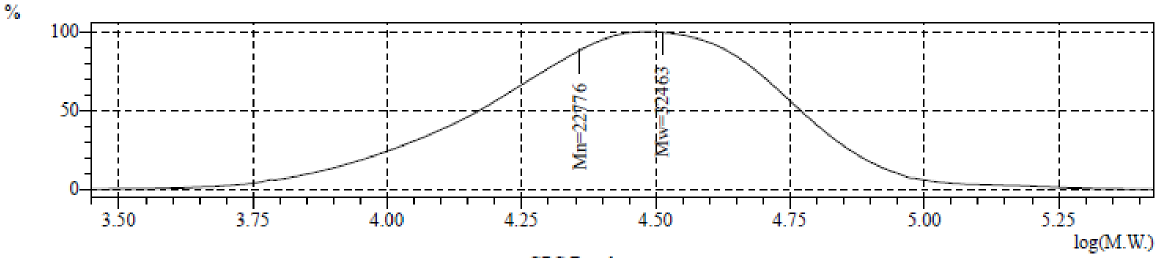

GPC Results

Peak#:1 (Detector A Channel 1)
[Peak Information]

Title	Time(min)	Molecular Weigl	Height
Start	11.733	268532	82
Top	14.643	30178	12796
End	18.225	2793	193

Area : 1671088

[Average Molecular Weight]

Number Average Molecular Weight(Mn)	22776
Weight Average Molecular Weight(Mw)	32463
Mw/Mn	1.42535

Figure S87: GPC Chromatogram of **pM1** made by 500 eq monomer in the presence of 5 eq EVE.

<Chromatogram>

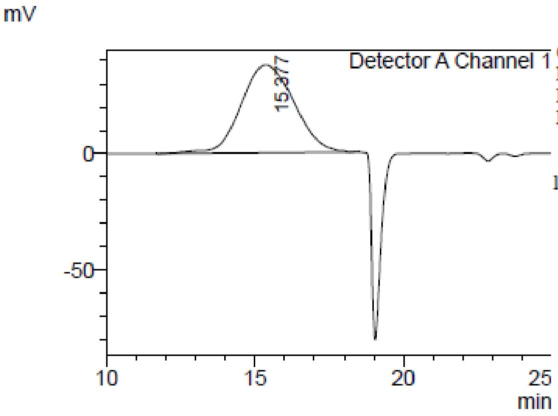

GPC Calibration Curve

Curve Fit Type : 3rd Order
R^2=0.9999813
Dispersion=0.002613652
Function : f(x)=-0.001304630*X^3+0.0640078
-1.331108*X+14.34273 (X=x-T.LIMI

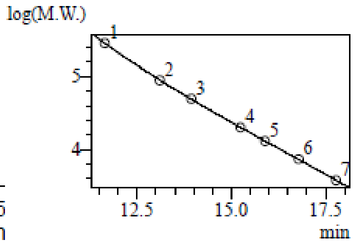

#	Molecular Weigh
1	288300
2	89050
3	49350
4	20020
5	13030
6	7350
7	3790

GPC Graph

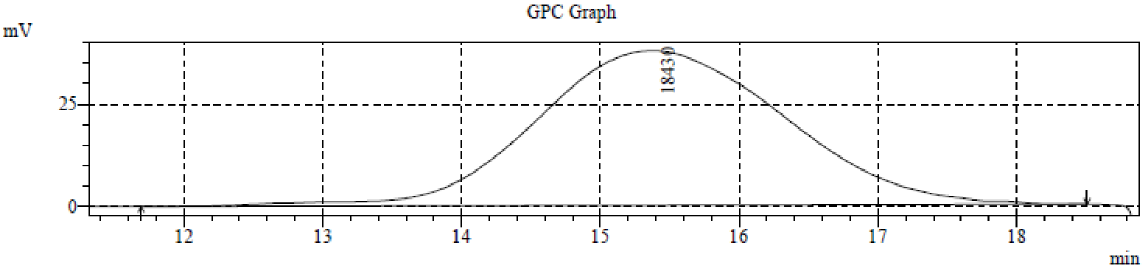

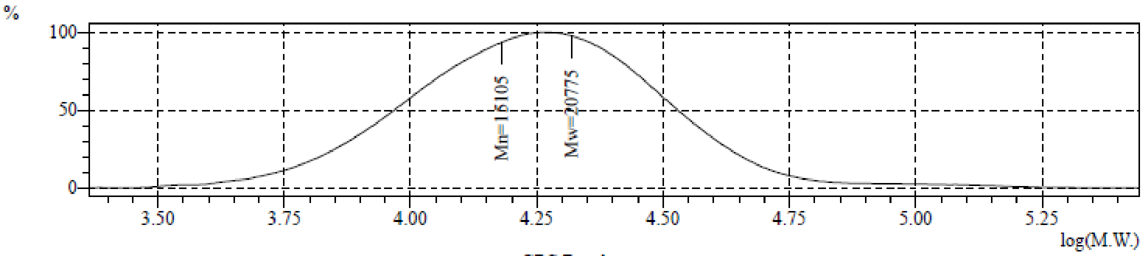

GPC Results

Peak#:1 (Detector A Channel 1)

[Peak Information]

Title	Time(min)	Molecular Weigl	Height
Start	11.692	278200	85
Top	15.377	18430	37656
End	18.508	2296	746

Area : 4656349

[Average Molecular Weight]

Number Average Molecular Weight(Mn)	15105
Weight Average Molecular Weight(Mw)	20775
Mw/Mn	1.37536

Figure S88: GPC Chromatogram of **pM1** made by 500 eq monomer in the presence of 10 eq EVE.

Chain extension pM1

GIII (9.6 mg) was dissolved in 13 mL DCM. Four separate solutions of **M1** (82.2 mg, 50 eq), each dissolved in 2 mL DCM, were prepared. Another four vials containing ethyl vinyl ether (2 mL each) were prepared. The first monomer solution was added to the catalyst and let stirring for 1 min before taking an aliquot of 1-2 mL that was then added to the quenching agent. It is important to note that a suspension needle was used here, since the polymer precipitates during the reaction. The reaction mixtures were brought outside the glovebox and the volatiles removed under reduced pressure. The crude materials were dissolved in 1,1,1,3,3,3-hexafluoroisopropanol (1 mL) and passed dropwise through a syringe filter into rigorously stirred MeOH (20 mL). The precipitated material was filtered off and dried in a vacuum oven set to 50 °C. The resulting polymers were isolated as off-white powders.

By taking aliquots, the expected Mn will be lower than the theoretical one, since the concentration of active polymer chains will be reduced in the process.

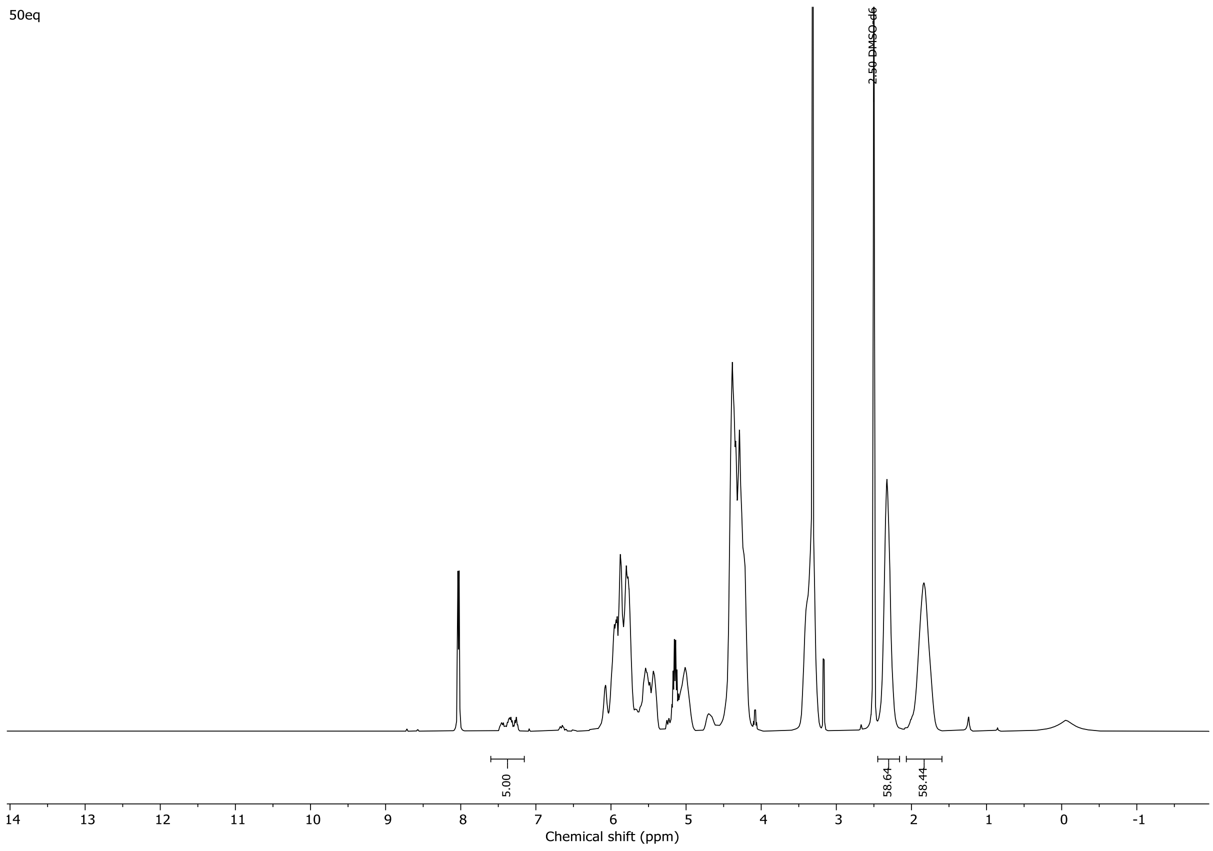

Figure S89: ^{1}H NMR spectrum of chain extension of **pM1** (first aliquot).

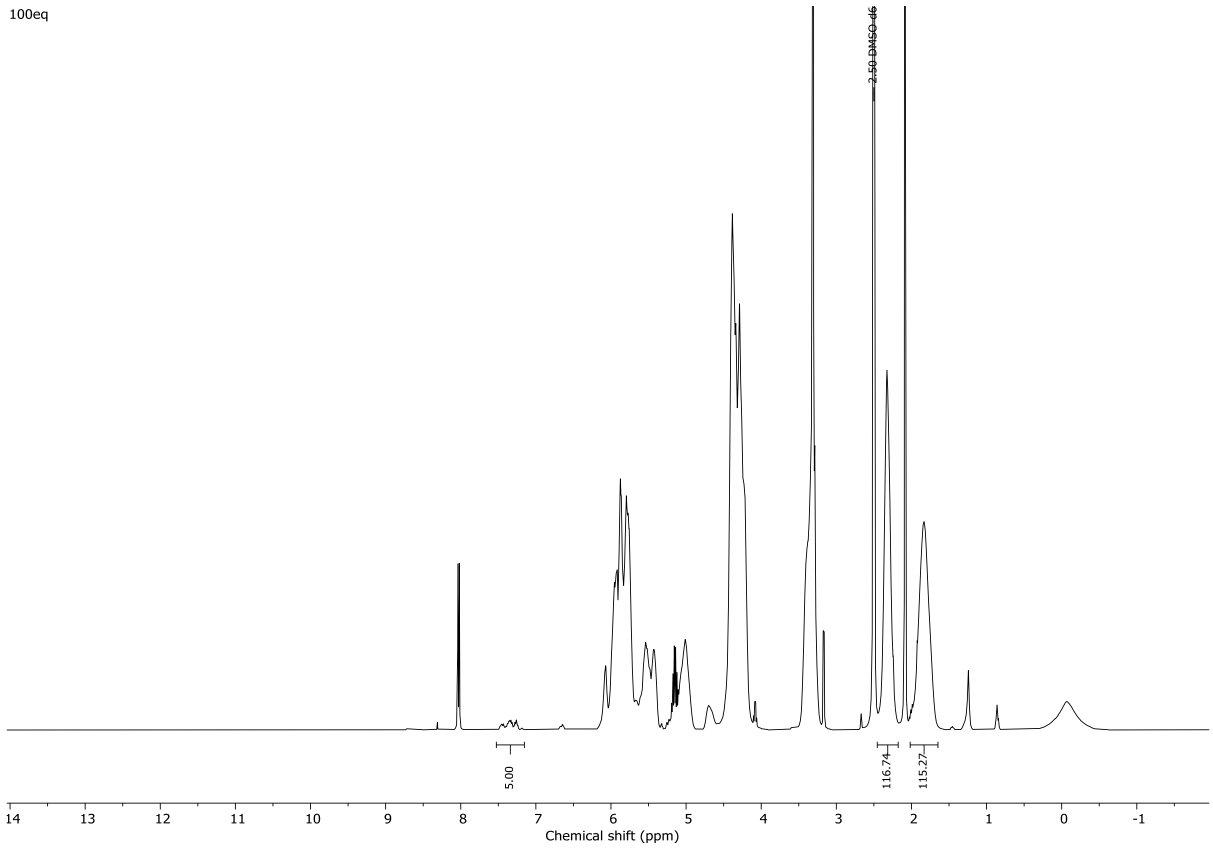

Figure S90: ^{1}H NMR spectrum of chain extension of **pM1** (second aliquot, 100 eq. added in total).

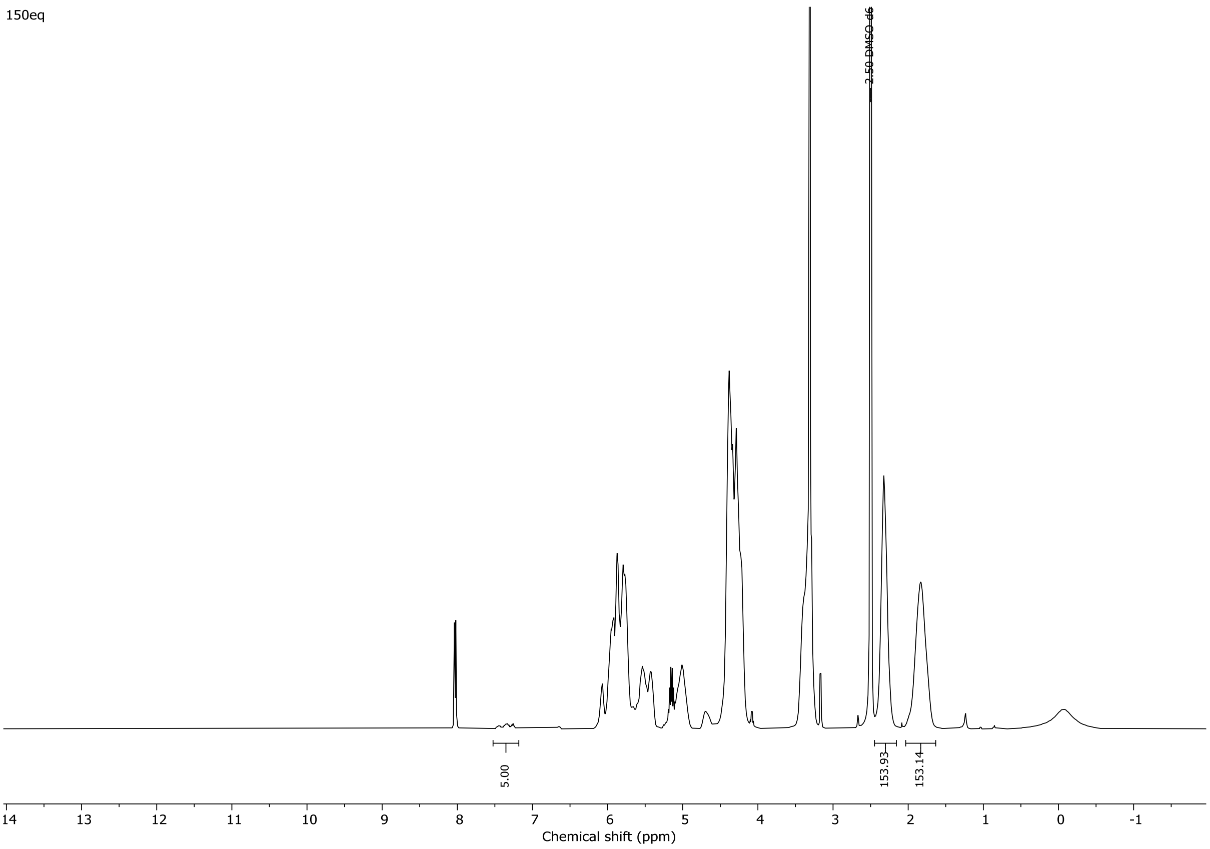

Figure S91: ^{1}H NMR spectrum of chain extension of **pM1** (second aliquot, 150 eq. added in total)

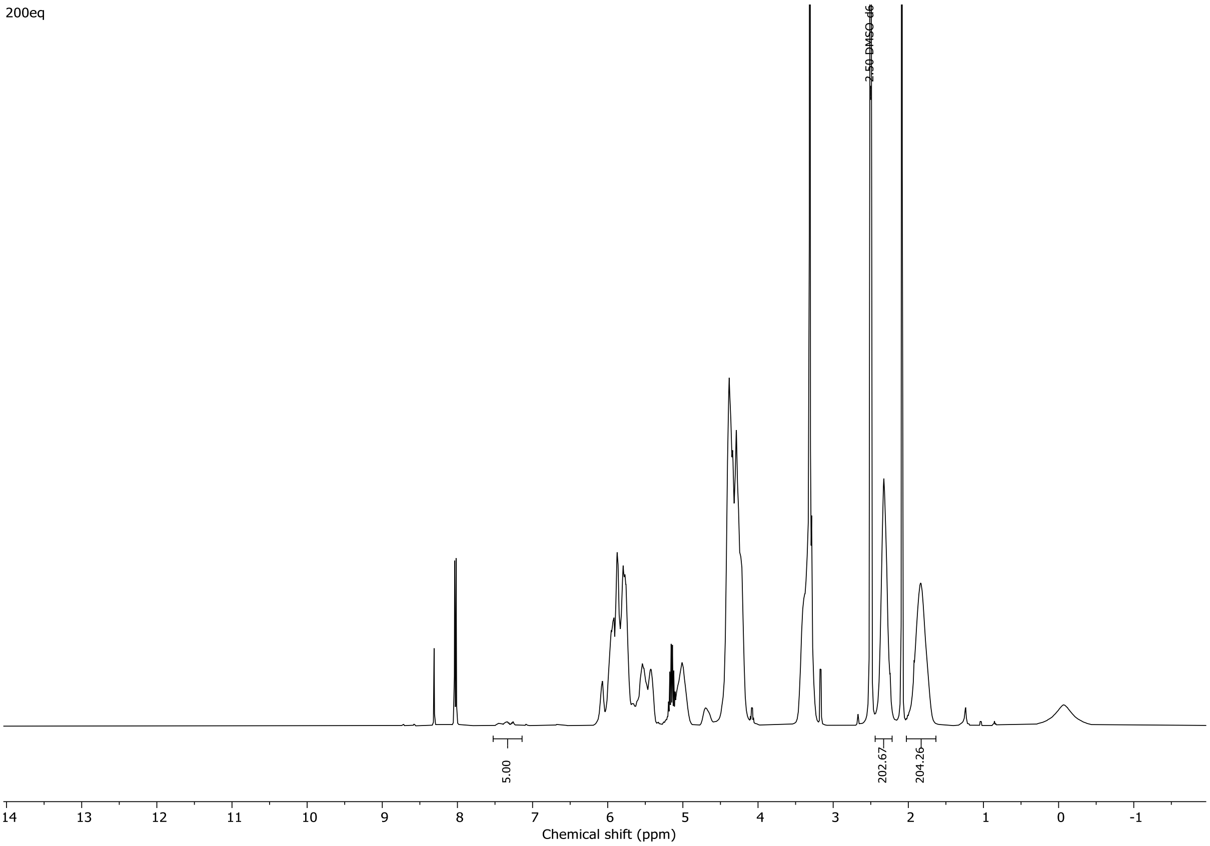

Figure 92: ^{1}H NMR spectrum of chain extension of **pM1** (second aliquot, 200 eq. added in total)

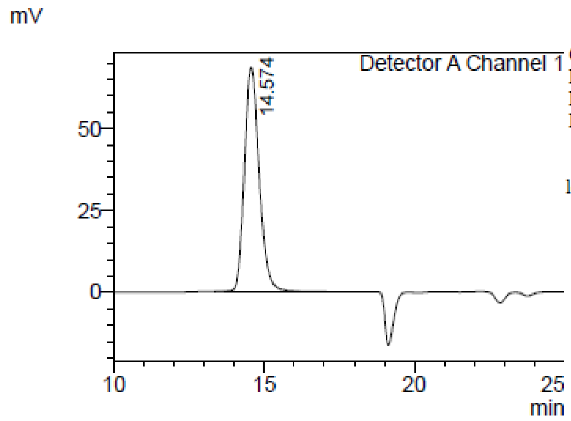

GPC Calibration Curve

Curve Fit Type : 3rd Order
R^2=0.9999813
Dispersion=0.002613652
Function : f(x)=-0.001304630*X^3+0.0640078
-1.331108*X+14.34273 (X=x-T.LIMI

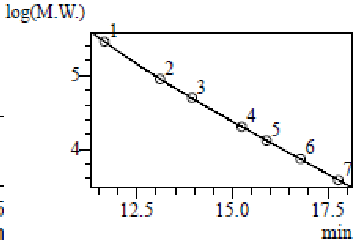

#	Iolecular Weigh
1	288300
2	89050
3	49350
4	20020
5	13030
6	7350
7	3790

GPC Graph

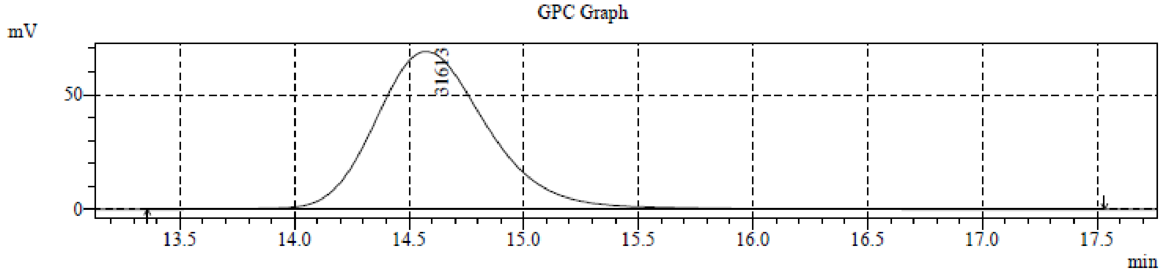

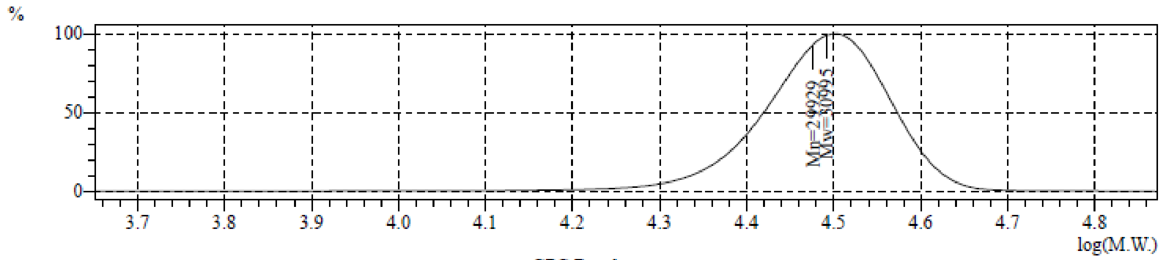

GPC Results

Peak#:1 (Detector A Channel 1)
[Peak Information]

Title	Time(min)	Iolecular Weigl	Height
Start	13.358	74703	196
Top	14.574	31613	68495
End	17.533	4457	147

Area : 2345694

[Average Molecular Weight]

Number Average Molecular Weight(Mn)	29929
Weight Average Molecular Weight(Mw)	30995
Mw/Mn	1.03561

Figure S93: GPC chromatogram of **pM1** consisting of 50 eq. **M1** (first aliquot).

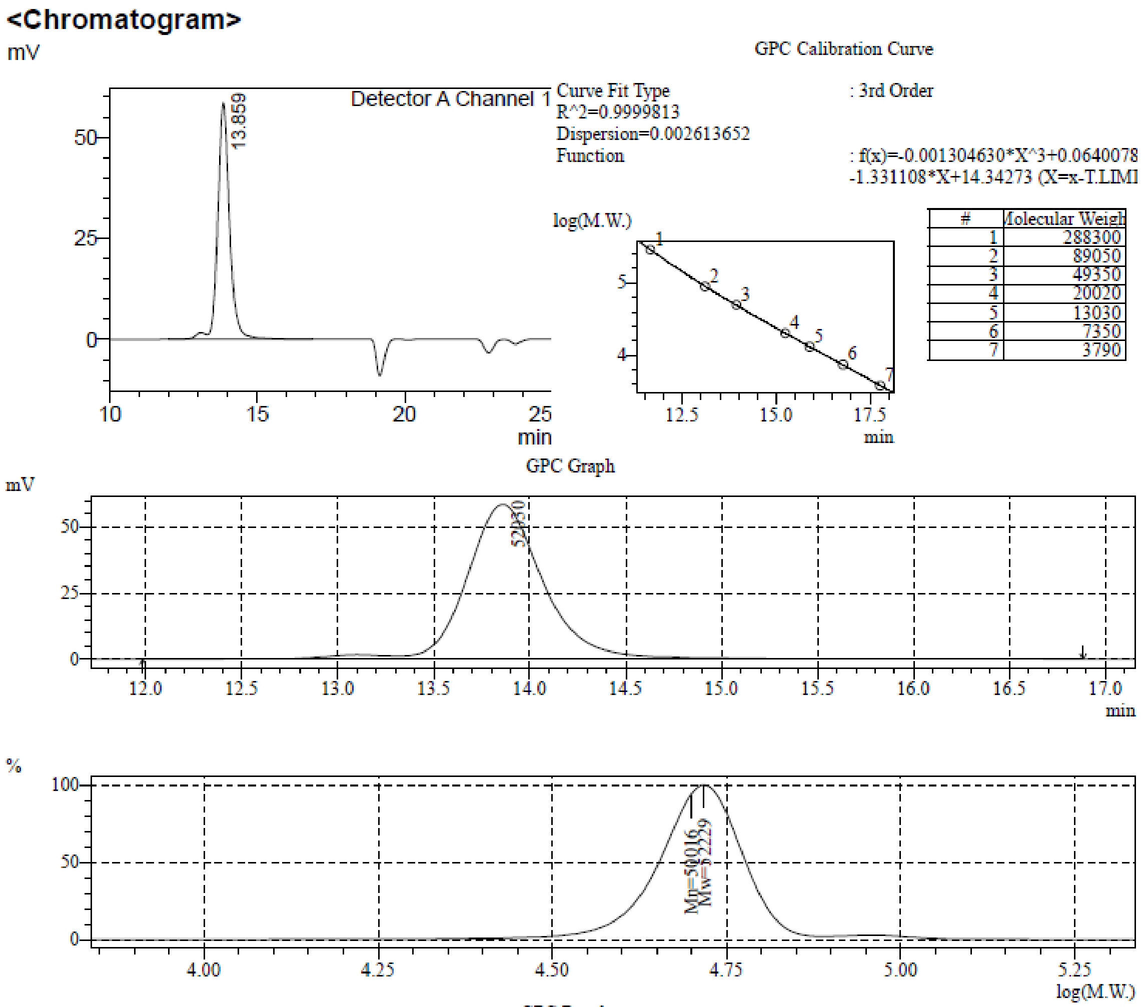

GPC Results

Peak#:1 (Detector A Channel 1)
[Peak Information]

Title	Time(min)	Molecular Weigl	Height
Start	11.983	217838	68
Top	13.859	52030	58383
End	16.883	6852	128

Area : 1647302

[Average Molecular Weight]

Number Average Molecular Weight(Mn)	50016
Weight Average Molecular Weight(Mw)	52229
Mw/Mn	1.04424

Figure S94: GPC chromatogram of **pM1** after first addition of additional monomer (100 eq. theoretically).

<Chromatogram>

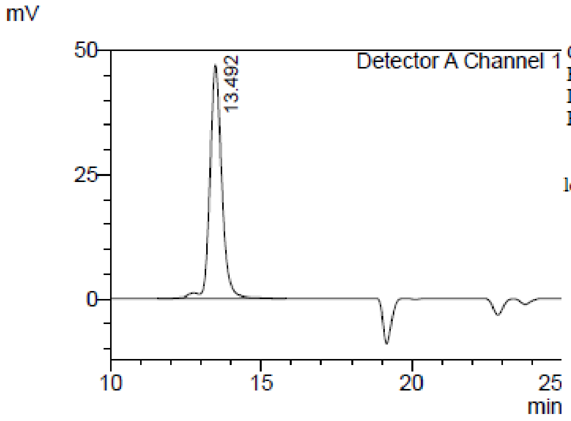

GPC Calibration Curve

Curve Fit Type : 3rd Order
R^2=0.9999813
Dispersion=0.002613652
Function : f(x)=-0.001304630*X^3+0.0640078
-1.331108*X+14.34273 (X=x-T.LIMI

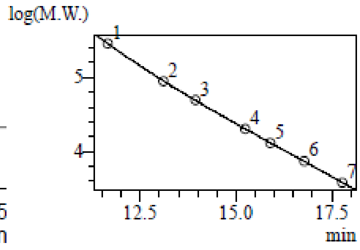

#	Molecular Weigh
1	288300
2	89050
3	49350
4	20020
5	13030
6	7350
7	3790

GPC Graph

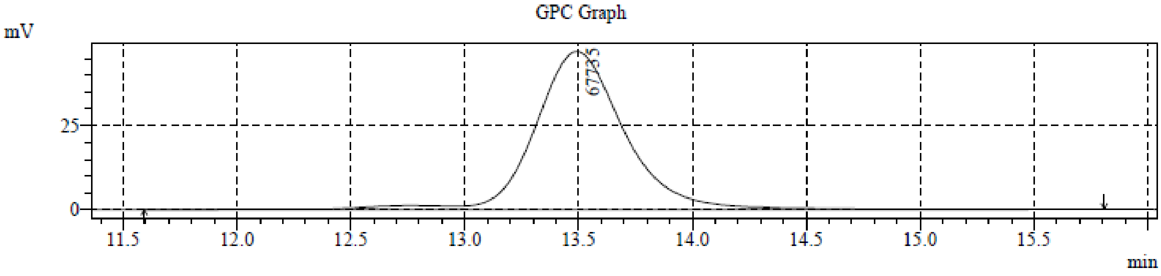

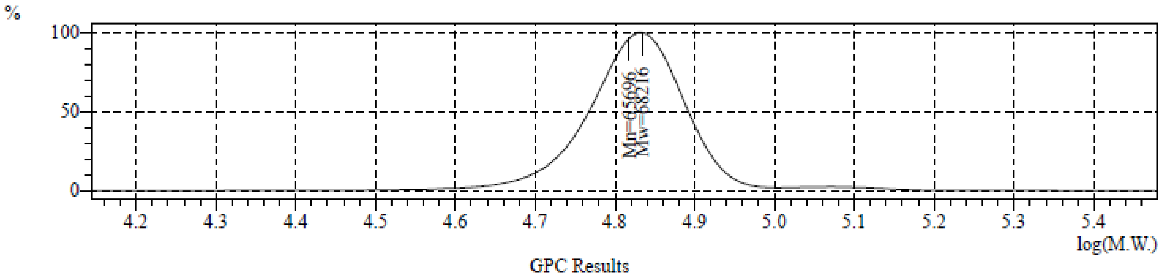

GPC Results

Peak#:1 (Detector A Channel 1)

[Peak Information]

Title	Time(min)	Molecular Weig	Height
Start	11.592	303026	73
Top	13.492	67735	46932
End	15.808	13864	137

Area : 1284099

[Average Molecular Weight]

Number Average Molecular Weight(Mn)	65696
Weight Average Molecular Weight(Mw)	68216
Mw/Mn	1.03836

Figure S95: GPC chromatogram of **pM1** after first addition of additional monomer (150 eq. theoretically).

<Chromatogram>

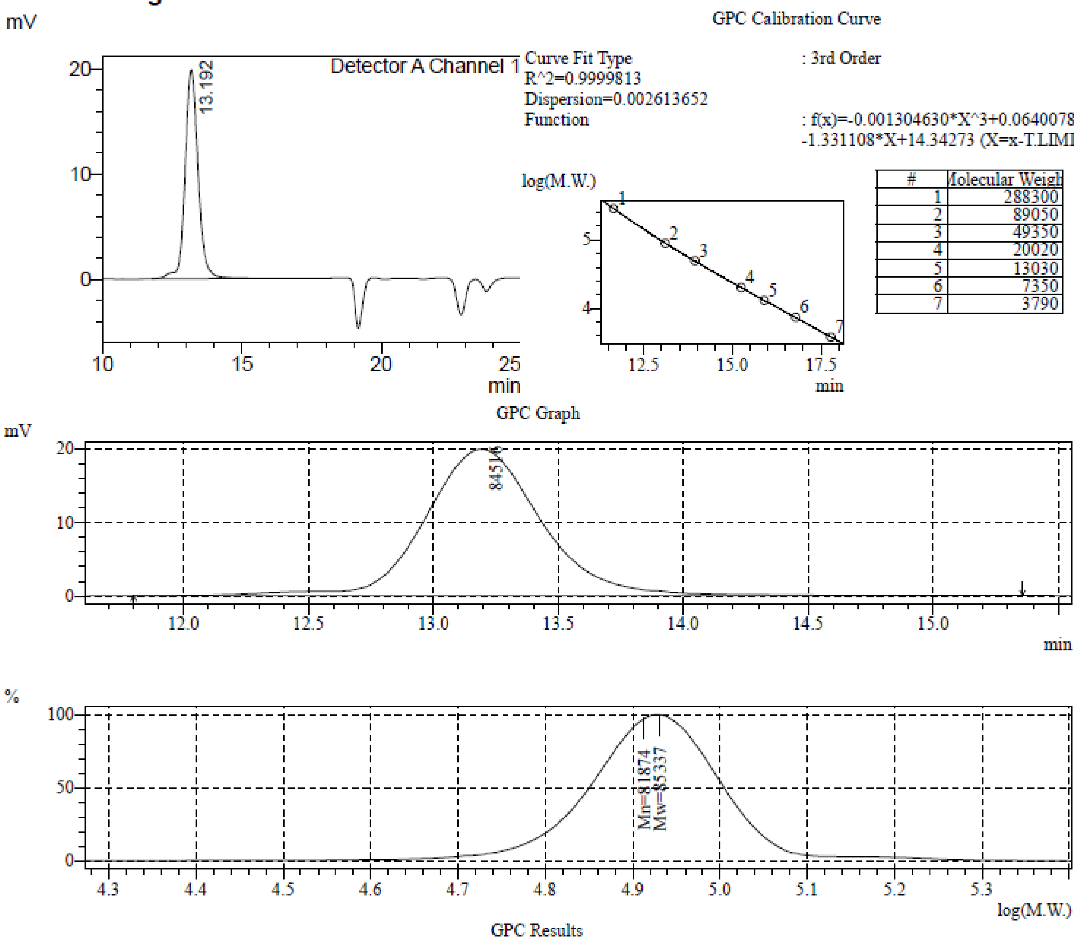

GPC Results

Peak#:1 (Detector A Channel 1)
[Peak Information]

Title	Time(min)	Molecular Weig	Height
Start	11.800	253834	74
Top	13.192	84516	19837
End	15.358	18660	103

Area : 642679

[Average Molecular Weight]

Number Average Molecular Weight(Mn)	81874
Weight Average Molecular Weight(Mw)	85337
Mw/Mn	1.04229

Figure S96: GPC chromatogram of **pM1** after first addition of additional monomer (200 eq. theoretically).

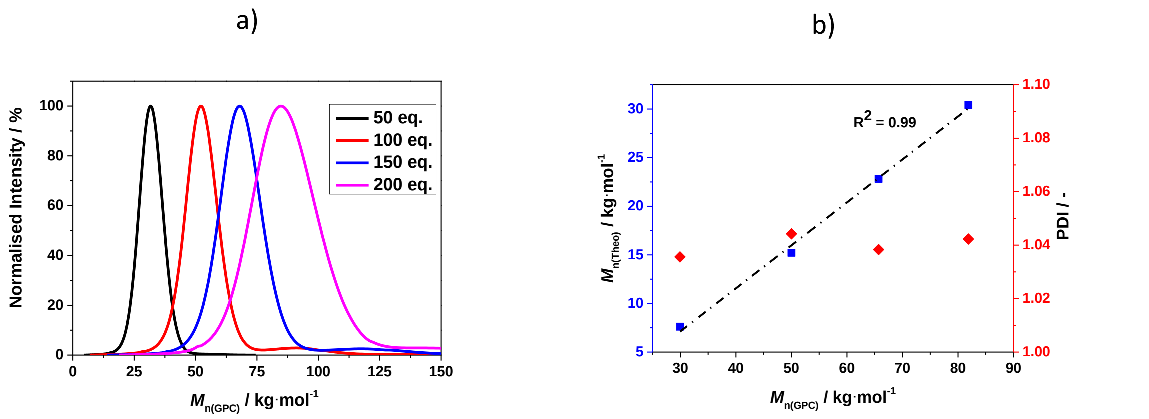

Figure S97: a) GPC traces of chain extension experiment; b) plot of $M_{n(Theo)}$ ($M_{(M1)}$ x monomer loading) against $M_{n(GPC)}$ to highlight the linearity of chain growth while maintaining narrow polydispersity.

Assignment linkages

Determining the regioregularity of the synthesized polymers was done via a combination of APT-$^{13}C-^{1}H$-HMBC and *J*-resolved NMR spectra. Since the ^{1}H NMR spectra of the polymers showed a significant broadening of the signals, *J*-resolved (JRES) NMR was chosen as a technique to identify the underlying multiplicity and thus assign the associated isomerism of the linkages. Spin-spin coupling (f1 dimension) information and chemical shift (f2 dimension) are separated in this experiment to allow for a more intuitive interpretation of the coupling information, which proved to be useful in the investigated systems, since they show significant signal broadening in ^{1}H NMR. Determination of multiplicity and coupling constants can be done by identifying vertically aligned resonances in the 2D plot and measuring the distance between them with an arbitrary cursor tool.

Depicted above the spectra are schematics of the correlations between carbon and proton nuclei that allowed a rational determination of connectivity. A Head-To-Tail (H-T) linkage should show relations between the alkene protons (H_{alk}) and the appended tertiary (C_t) and quartenary (C_q) carbon, a Tail-To-Tail (T-T) linkage would be determined through $H_{alk}-C_q$ only and Head-To-Head (H-T) through $H_{alk}-C_t$. The percentage of each linkage is determined through integration of the HSQC spectra (Figure S79).

Assignment linkages pM1

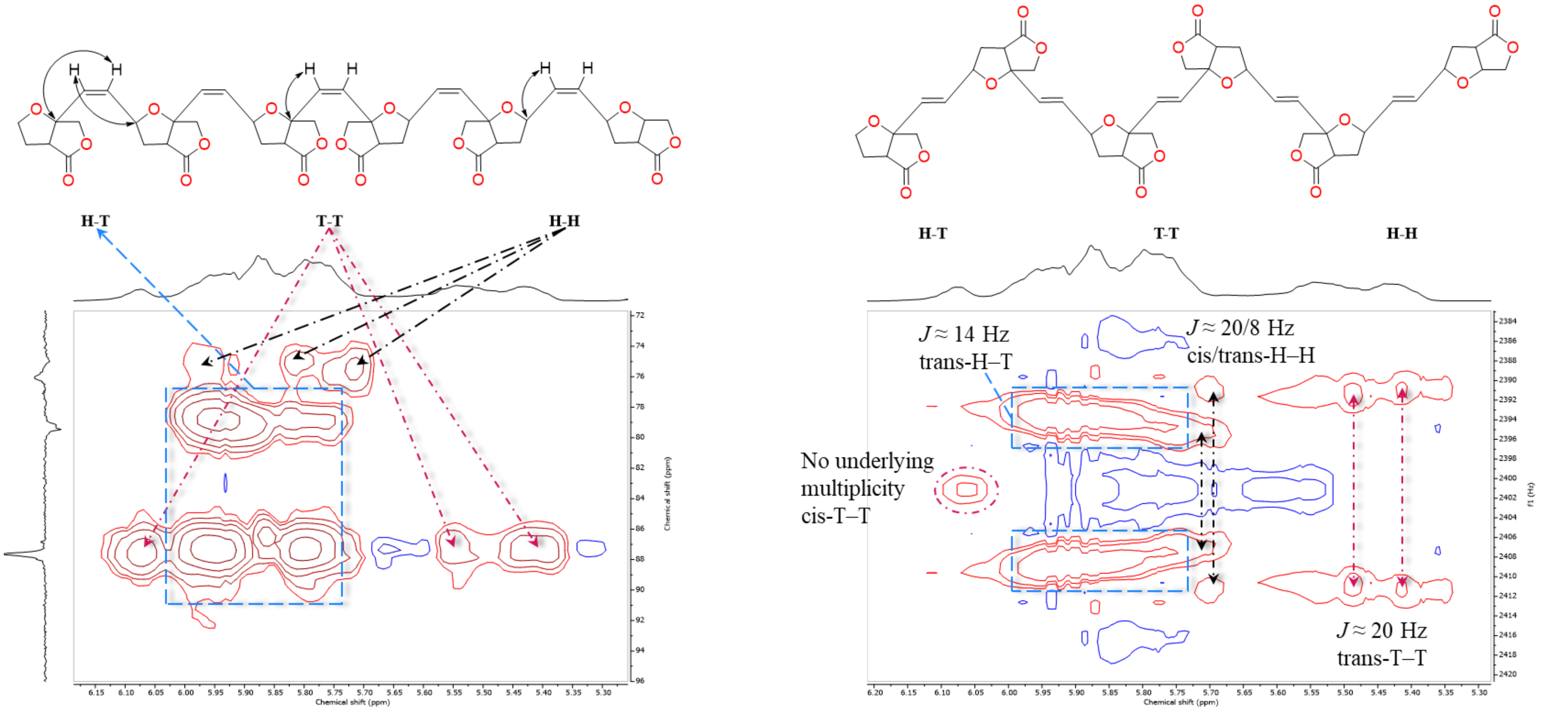

Figure S98: Color-coded rationale behind assignment of linkages in **pM1** through a combination of HMBC (left) and J-resolved (right) NMR spectra (H = Head, T = Tail, blue = Head-to-Tail, fuchsia = Tail-to-Tail, black = Head-to-Head). HMBC $^{2}J_{13C-1H}$ and $^{3}J_{13C-1H}$ correlations are depicted with double-headed arrows, a schematic representation of both cis- and trans-linkages are above the spectra.

Assignment linkages pM2

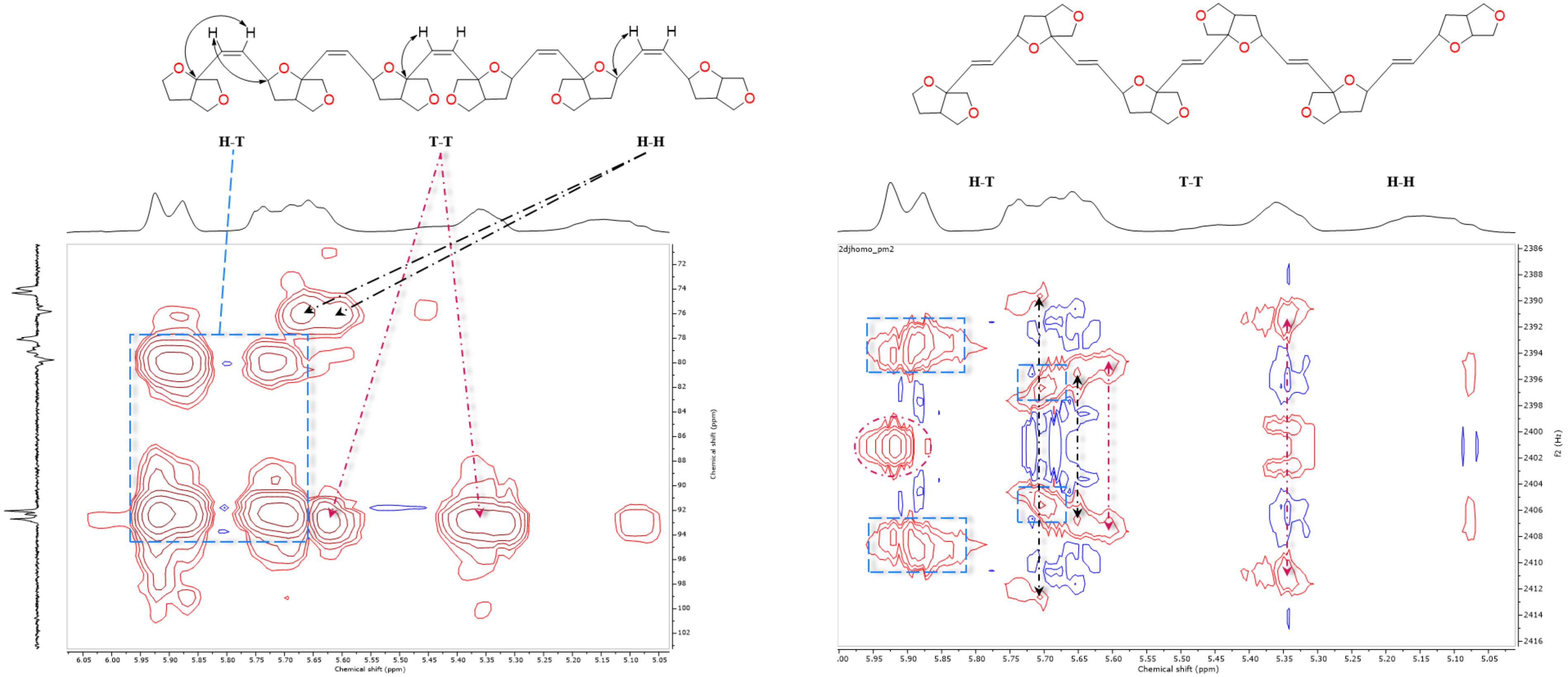

Figure S99: Color-coded rationale behind assignment of linkages in **pM2** through a combination of HMBC (left) and J-resolved (right) NMR spectra (H = Head, T = Tail, blue = Head-to-Tail, fuchsia = Tail-to-Tail, black = Head-to-Head). HMBC $^{2}J_{13C-1H}$ and $^{3}J_{13C-1H}$ correlations are depicted with double-headed arrows, a schematic representation of both cis- and trans-linkages are above the spectra.

In the HMBC of **M2** a cluster of 4 crosspeak-resonances can be seen at (5.92/92.3) ppm, (5.89/92.3) ppm, (5.73/80.1) ppm, (5.70/80.1) ppm respectively. These ^{1}H NMR resonances show coupling to both C_q and C_t which can be assigned to H–T linkages. The associated coupling constants as determined by JRES are in agreement with conventional H–T_{trans} and H–T_{cis} isomerism. The two main resonance couples at (5.67/93.3, trans) and (5.62/92.8, cis) ppm can be assigned to H-H linkages through their coupling to C_t only, which would indicate a H–H connection, while the residual peak pairs at (5.61/76.2, cis) and (5.33/76.2, trans) ppm show coupling to C_q only, making them assignable to a T–T link. JRES NMR of those peak pairs shows high values for the trans-isomers of both linkages with 20 Hz while the cis isomer is implied by a coupling of 8 Hz.

Assignment linkages pM3

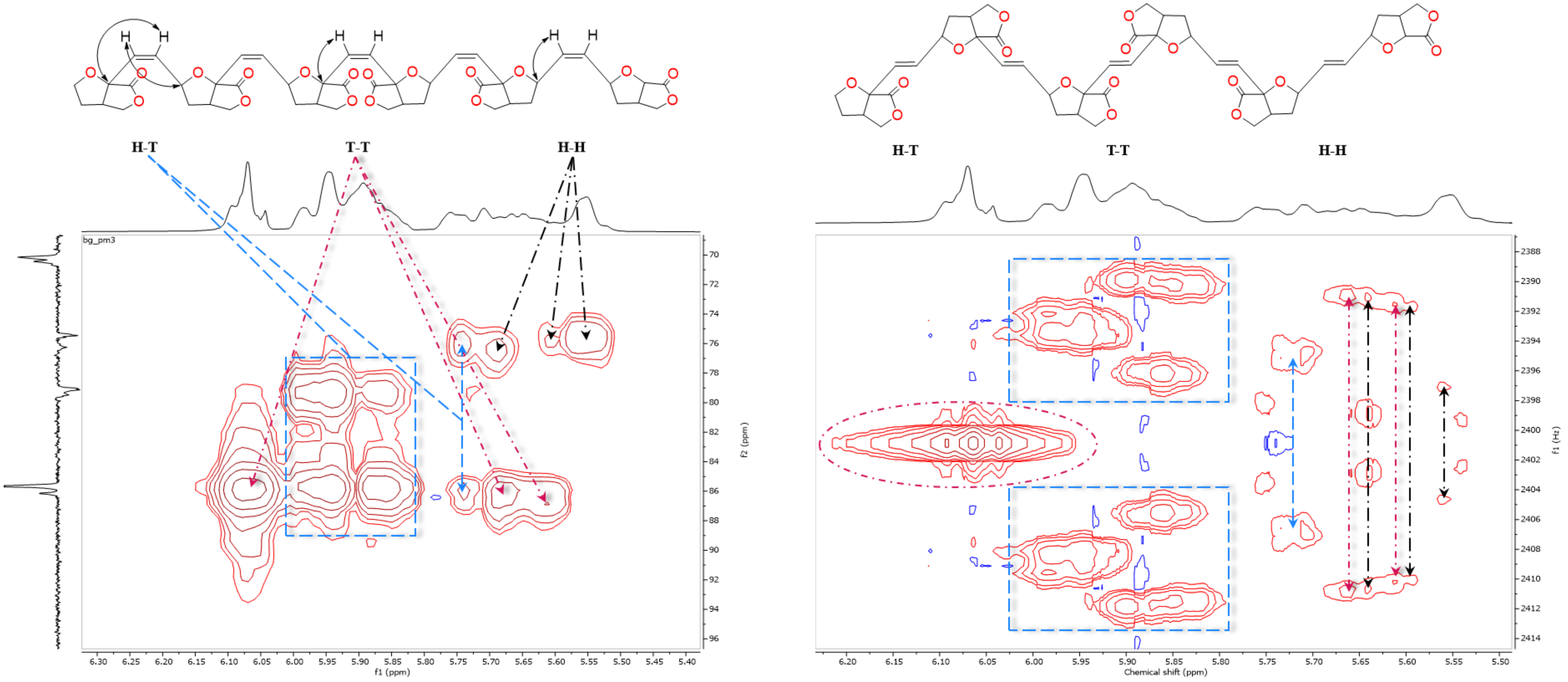

Figure S100: Color-coded rationale behind assignment of linkages in **pM3** through a combination of HMBC (left) and J-resolved (right) NMR spectra (H = Head, T = Tail, blue = Head-to-Tail, fuchsia = Tail-to-Tail, black = Head-to-Head). HMBC $^{2}J_{13C-1H}$ and $^{3}J_{13C-1H}$ correlations are depicted with double-headed arrows, a schematic representation of both cis- and trans-linkages are above the spectra.

HMBC analysis of **pM3** shows one proton at 6.10 ppm that only correlates to a quaternary carbon at 86 ppm, and another proton at 5.55 ppm that only correlates to a tertiary carbon at 76 ppm. According to our previous discussion, they are assigned as T–T and H–H linkages, respectively. JRES NMR spectra shows that H-H linkages identified at 5.55 ppm in the ^{1}H NMR spectrum consist of the cis geometrical isomer (J = 8 Hz). The normalized integration of the corresponding interlinked peak areas in the HSQC spectrum led us to the conclusion that there are 20% T–T and thus 20% H–H linkages, the latter consisting of at least 10% H–H$_{cis}$ connectivity.

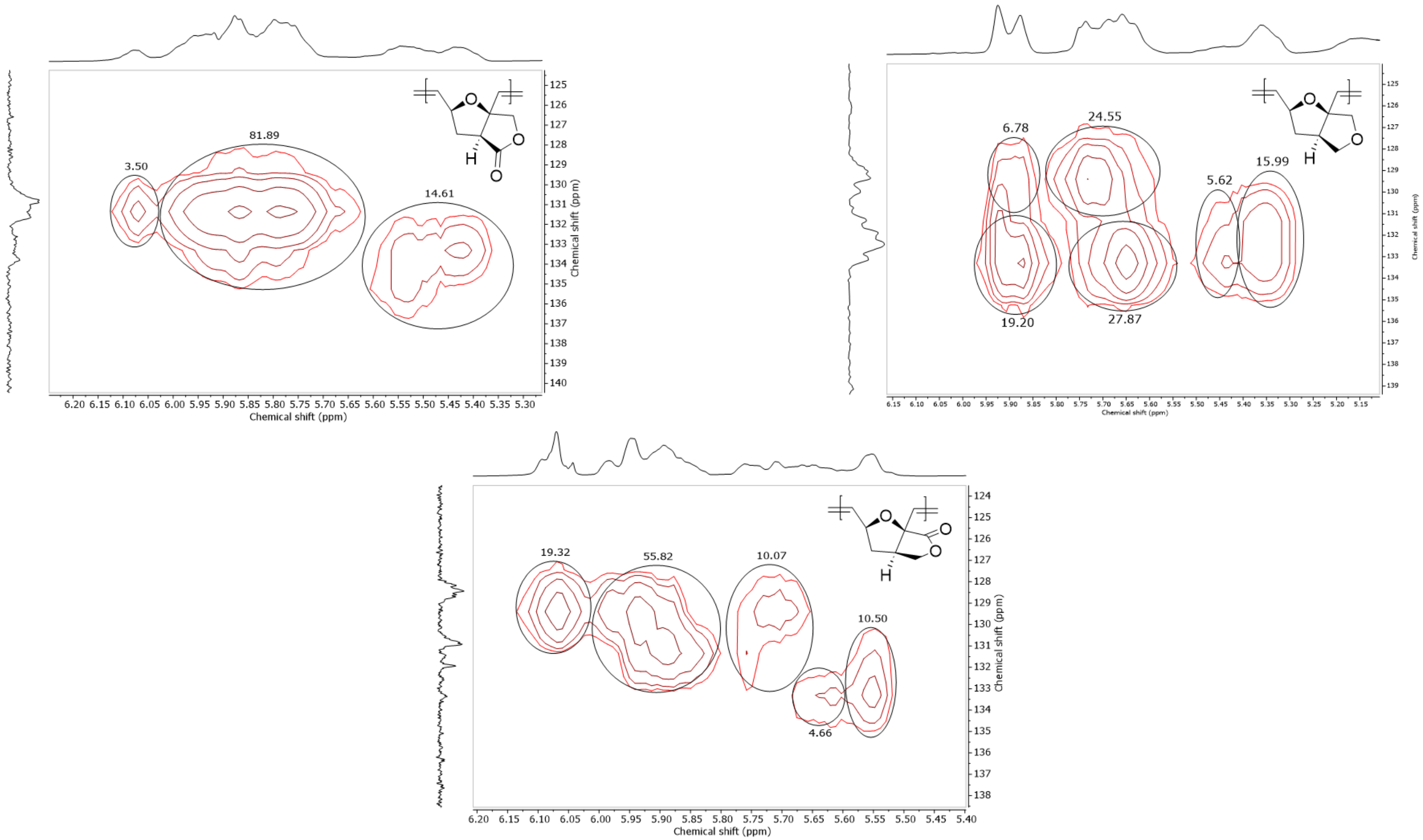

Figure S101: Alkene regions of HSQC NMR spectra. The integrated areas are set to a total of 100 protons to obtain the percentages of different linkages for each polymer.

Table S1: Percentages of linkages determined by HSQC NMR data.

Polymer	%H – T	%H – H	%T – T
pM1	64	18	18
pM2	66	16	16
pM3	60	20	20

Kinetic studies

In a glovebox monomer (**M1** and **M3** 164.3 mg, **M2** 152.2 mg, final concentration in reaction 0.068 M) was dissolved in 8 mL DCM. **GIII** (9.6 mg) was dissolved in 8 mL DCM and partitioned between seven screw cap vials (1 mL of catalyst solution each). 1 mL of monomer stock solution was successively added to each vial and the reaction quenched with ethyl vinyl ether (2 mL) after definite time increments. The vials were brought outside the box and the solvent and excess quenching agent removed via rotary distillation. To the residues 1 mL of DMSO-D6 was added for successive NMR analysis (1H, relaxation delay = 5 s).

The reaction constants k_{obs} were obtained by plotting the natural logarithm of the integral area of selected resonances (~6.6 and ~6.1 ppm; corresponding to the formation of ring-opened product after quenching as discussed in main text) against the time (see figure below).

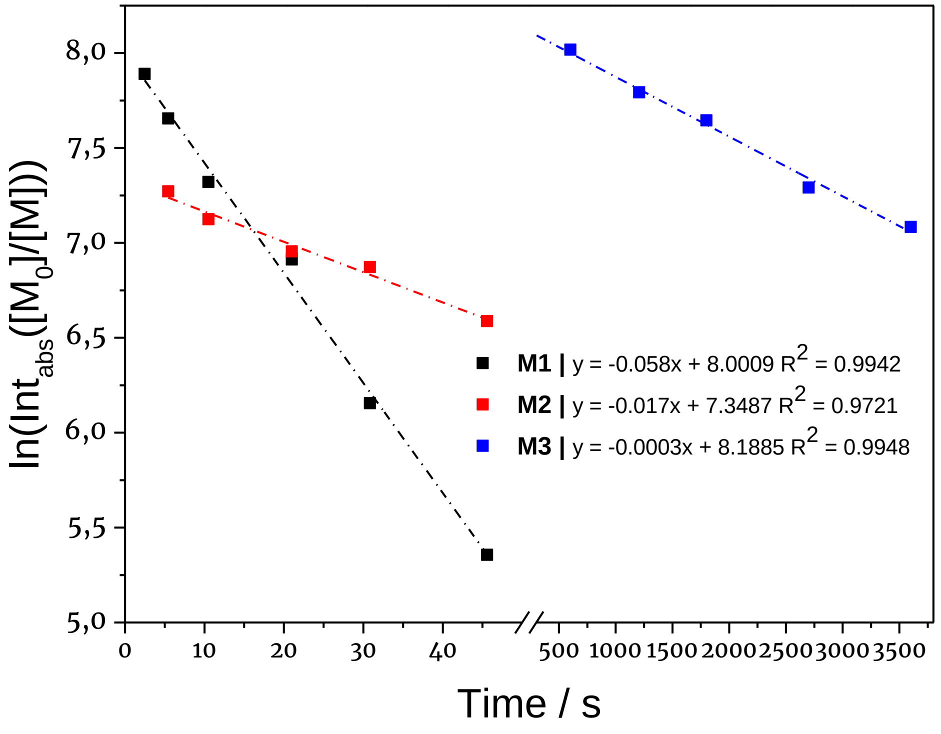

Figure S102: Plot of natural logarithm of absolute integral area of ring-opened products against time.

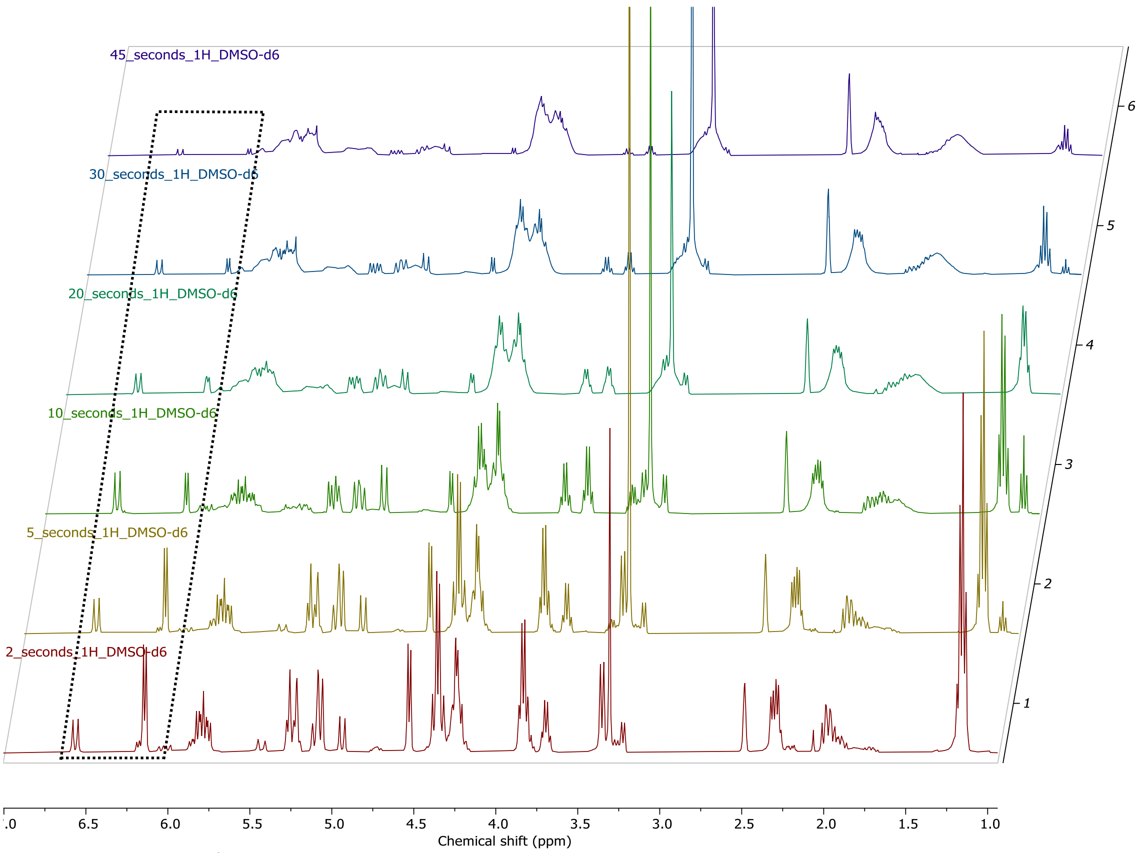

Figure S103: Stack of ^{1}H NMR spectra (DMSO-D6) of the polymerization of **M1** with GIII quenched at various time.

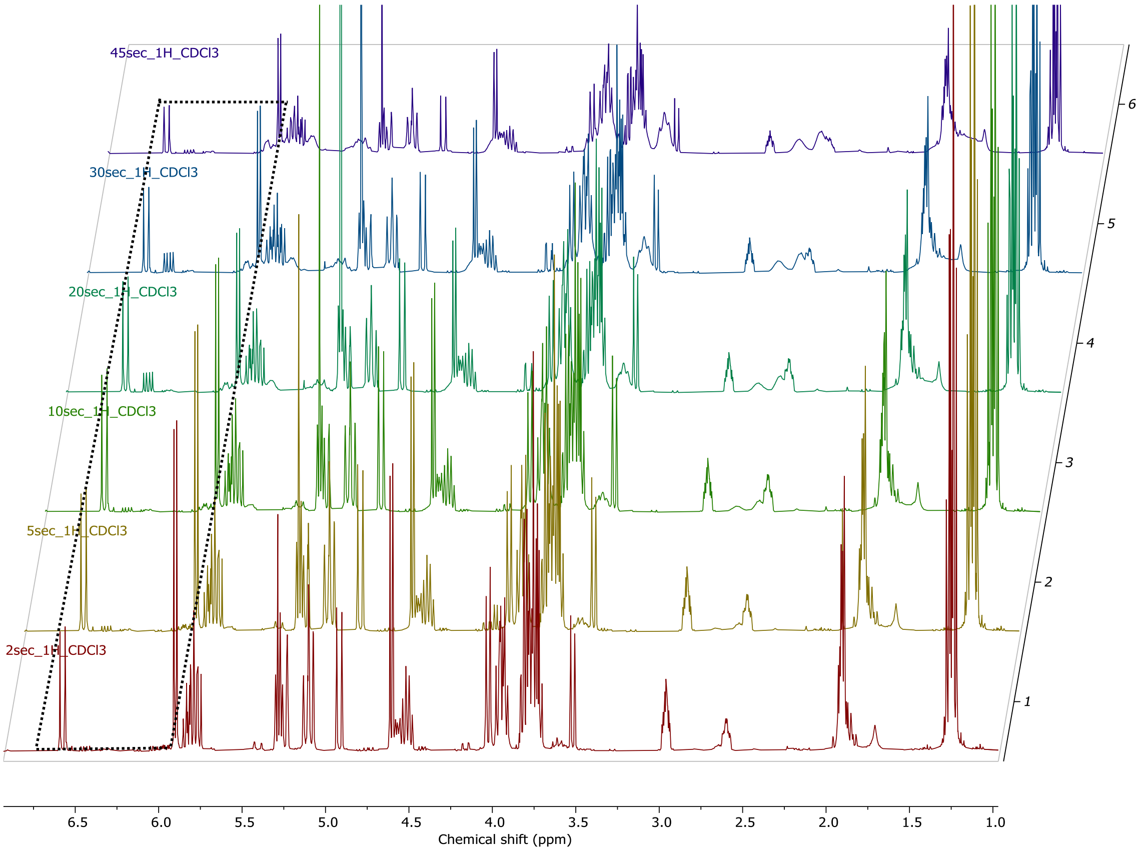

Figure S104: Stack of ^{1}H NMR spectra (DMSO-D6) of the polymerization of **M2** with GIII quenched at various time.

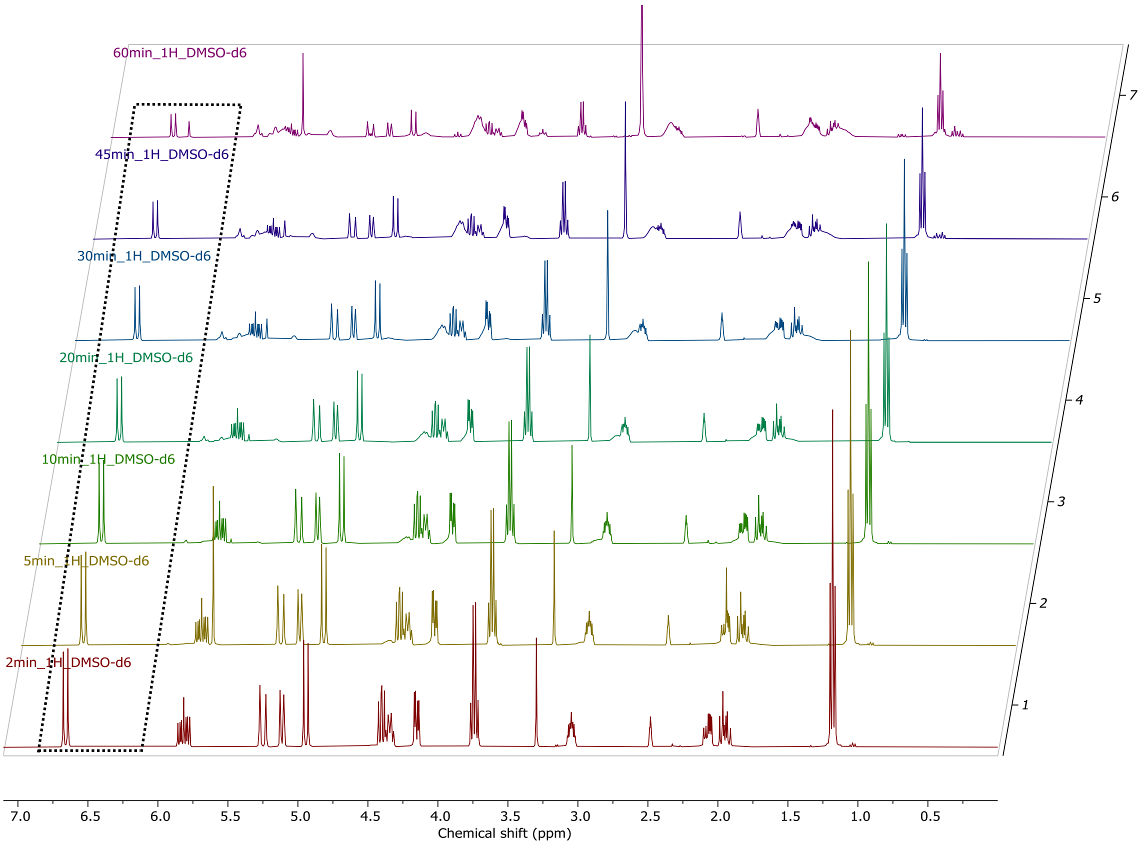

Figure S105: Stack of ^{1}H NMR spectra (DMSO-D6) of the polymerization of **M3** with GIII quenched at various time.

In a glovebox, an NMR tube was preloaded with monomer **M1**, **M2** or **M3** (15, 16, 15 mg) dissolved in 0.3 mL DCM-d2 and capped with a rubber septum. A solution of **GIII** (9.6 mg) in 0.3 mL DCM-D2 was prepared and drawn into a 1 mL syringe. The NMR tube and syringe were brought outside of the glovebox and stored in a Styrofoam box filled with dry ice (Note: it is important to not cool down the septum of the NMR tube, since thermal contraction can lead to contamination with oxygen and spilling of the sample). The solution of the catalyst was added through the septum and the NMR tube was turned upside down once to ensure mixing of both solutions. The reaction mixture was quickly inserted into the NMR machine, which probe head was precooled to 0 °C(*). 500 spectra were collected in total for each sample with a sampling density of 1 s.

The reaction constant was determined through integral analysis of the dissipating benzylidene resonance of unreacted GIII at 19.1 ppm (exponential apodization of 1 Hz prior to integration).

(*)It is recommended to use a fully reacted sample for shimming at 0 °C prior to the kinetic measurements.

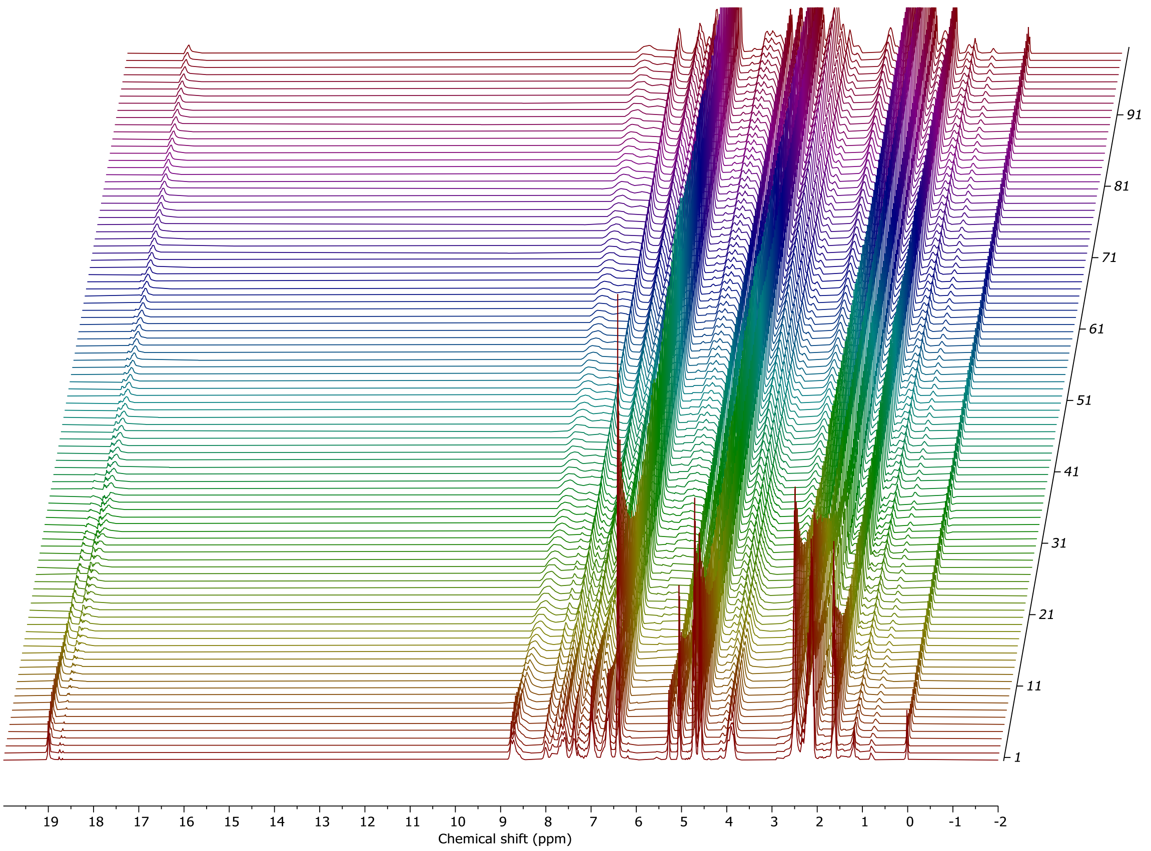

Figure S106: Stack of ^{1}H NMR spectra of the reaction of GIII with 10 equiv. of **M1** at 0 °C over time

.

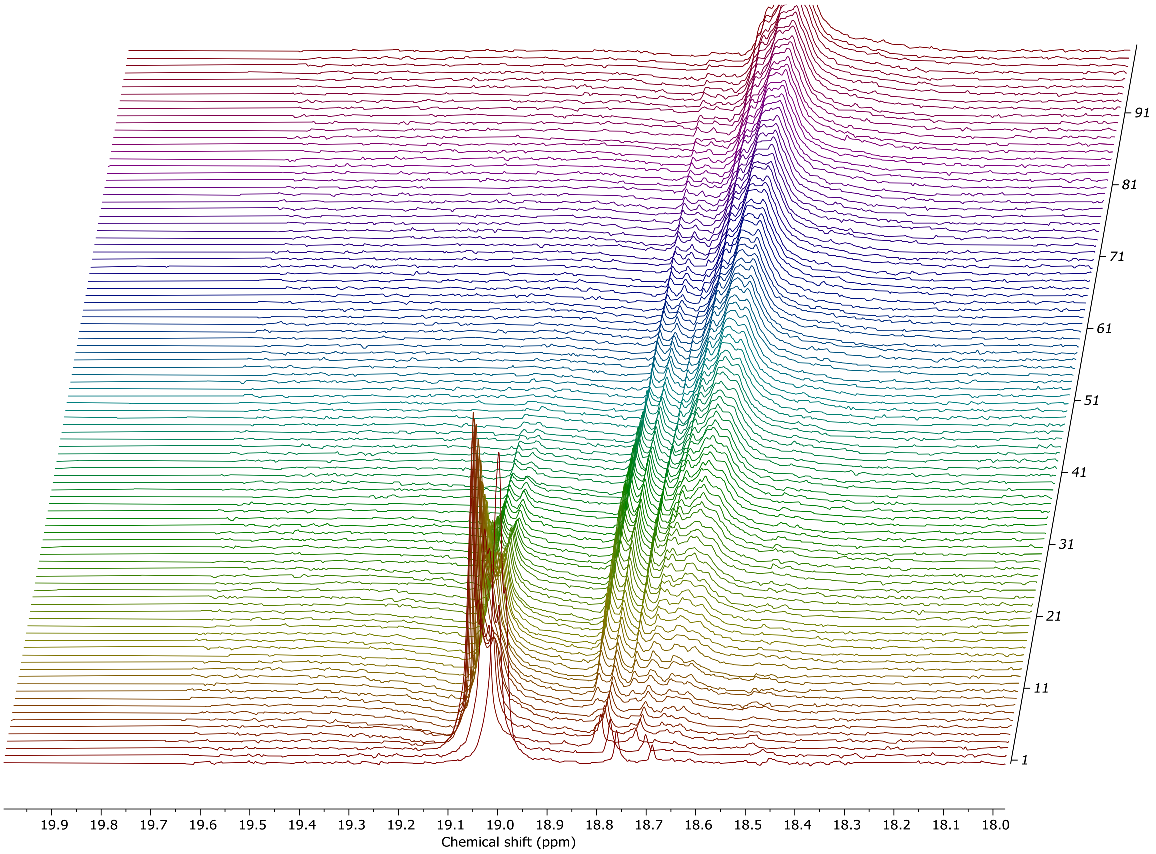

Figure S107: Stack of ^{1}H NMR spectra of the reaction of GIII with 10 equiv. of **M1** at 0 °C over time zoomed in the benzylidene region.

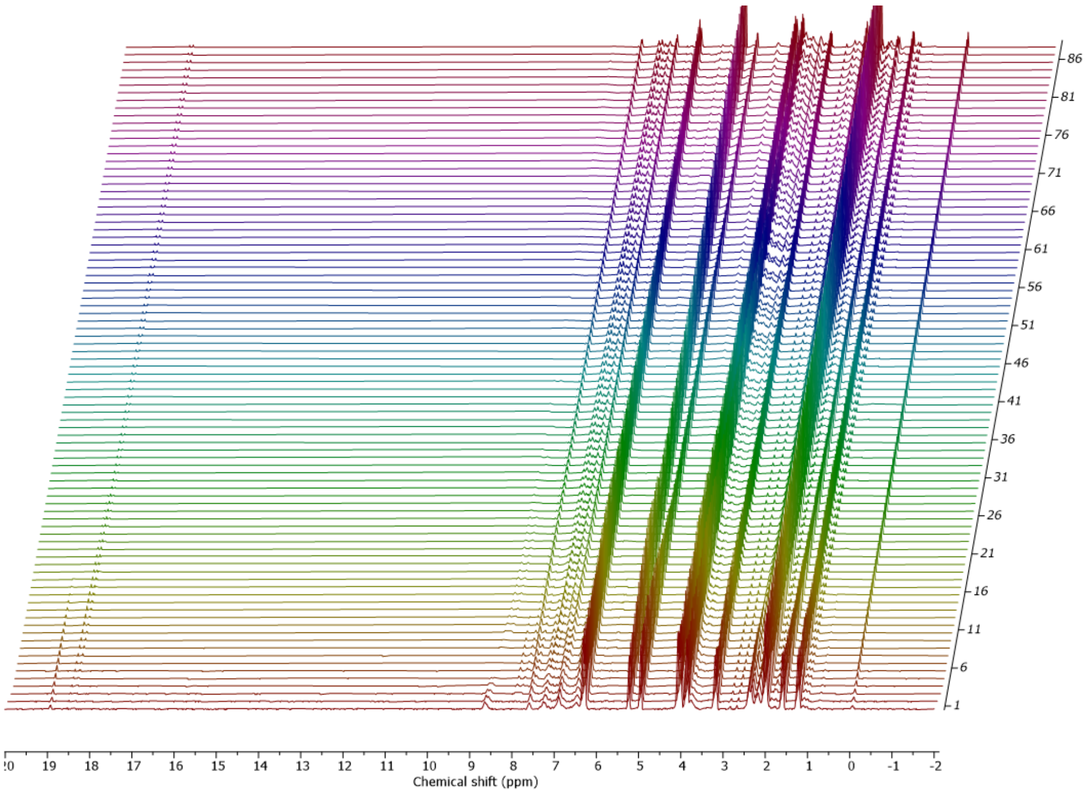

Figure S108: Stack of ^{1}H NMR spectra of the reaction of GIII with 10 equiv. of **M2** at 0 °C over time.

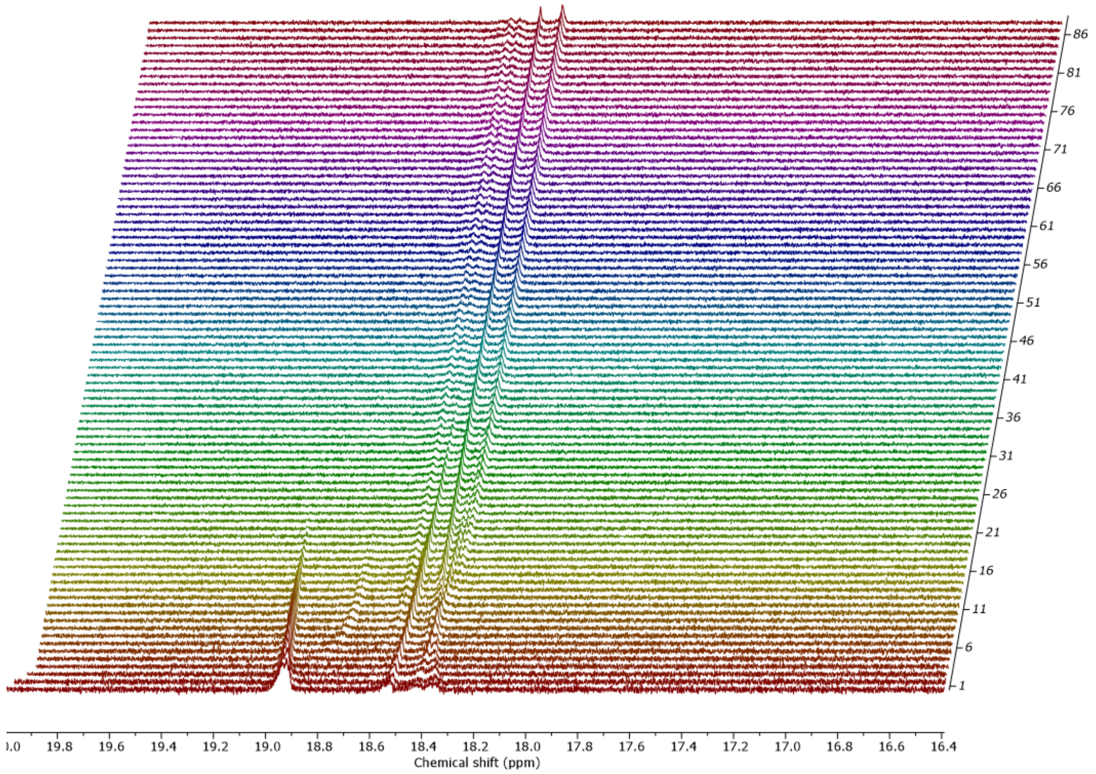

Figure S109: Stack of ^{1}H NMR spectra of the reaction of GIII with 10 equiv. of **M2** at 0 °C over time zoomed in the benzylidene region.

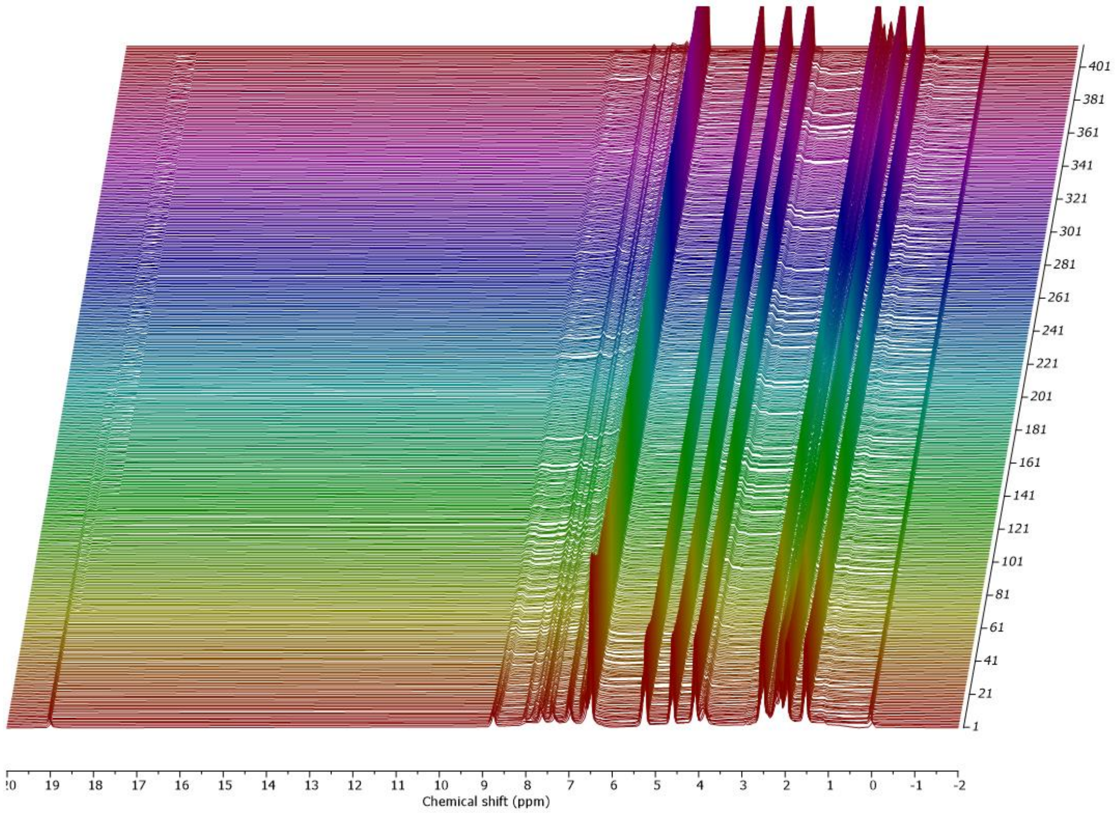

Figure S110: Stack of ^{1}H NMR spectra of the reaction of GIII with 10 equiv. of **M3** at 0 °C over time.

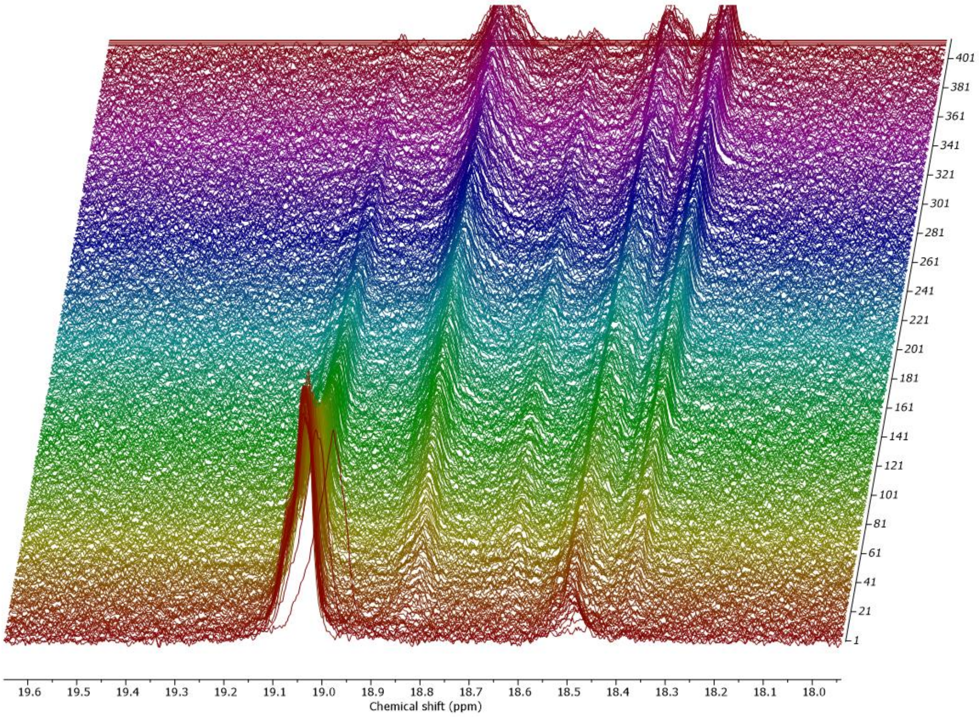

Figure S111: Stack of ^{1}H NMR spectra of the reaction of GIII with 10 equiv. of **M3** at 0 °C over time zoomed in the benzylidene region

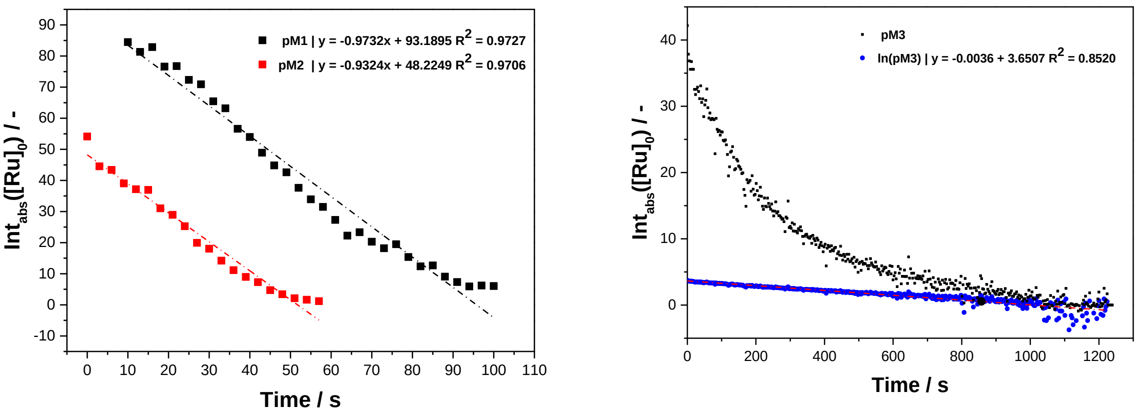

Figure S112: Plot of integral areas of corresponding benzylidene regions against time.

.

Chelation studies[4]

A solution of **M1** or **M3** (16.4 mg, 10 eq.) in CD_2Cl_2 (0.5 mL) was added to J Young NMR tube containing **pyr-GIII** (9.6 mg, 10 mol%) in CD_2Cl_2 (0.5 mL). The NMR tube was then inserted into a precooled machine and ^{1}H NMR spectra were collected in 10 °C intervals between -60 and 20 °C (8 scans each, 5 s relaxation delay, frequency tuning and gradient shimming done at every interval point). The observed equivalents of coordinated (7.9 ppm) and uncoordinated (8.6 ppm) pyridine were integrated.

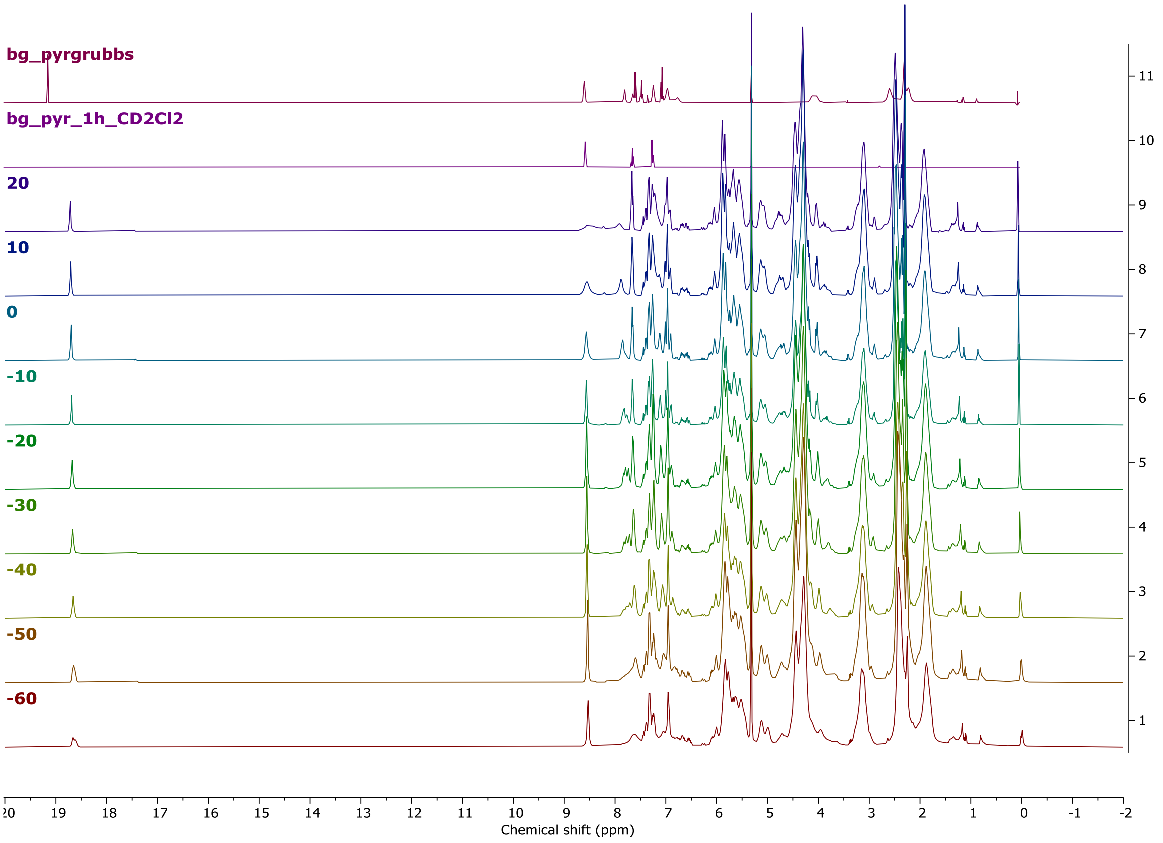

Figure 113: Stacked ^{1}H VT NMR spectra of **pM1**.

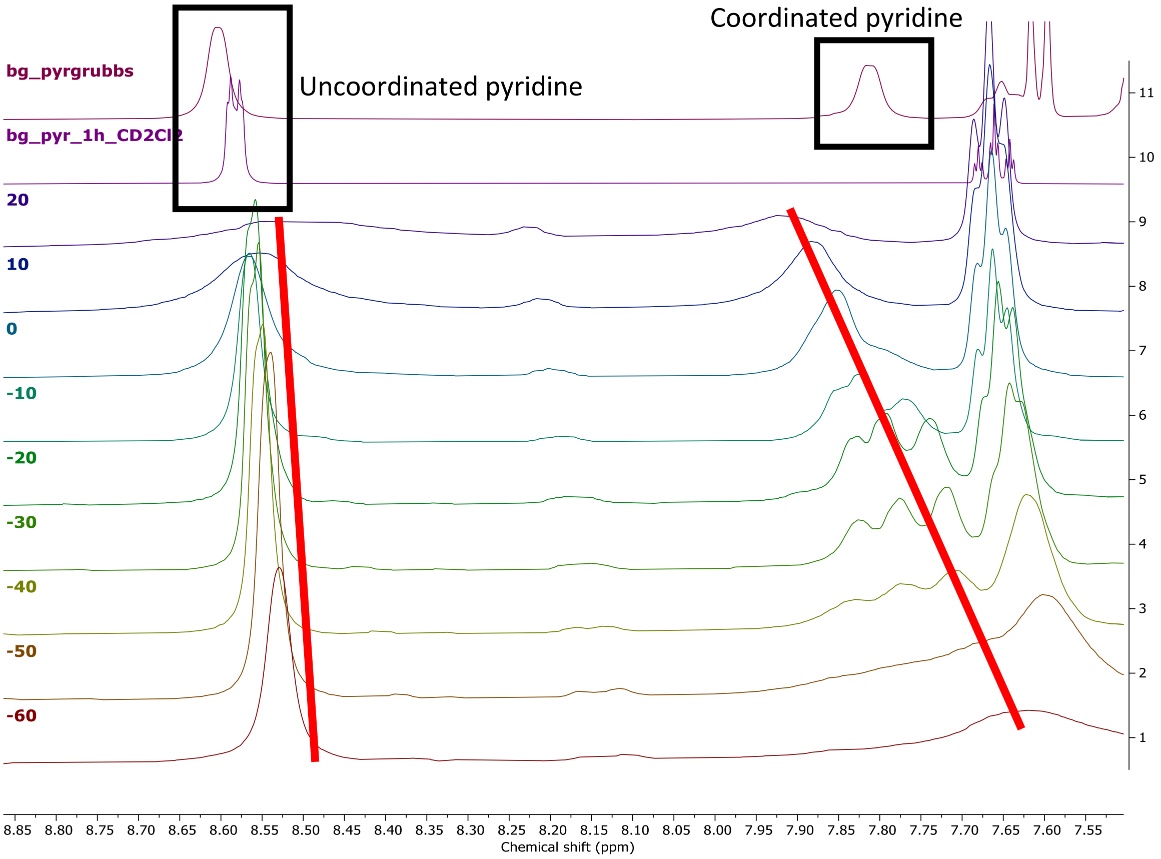

Figure S114: Stacked ^{1}H VT NMR spectra of **pM1** highlighting the temperature dependent shift of pyridine.

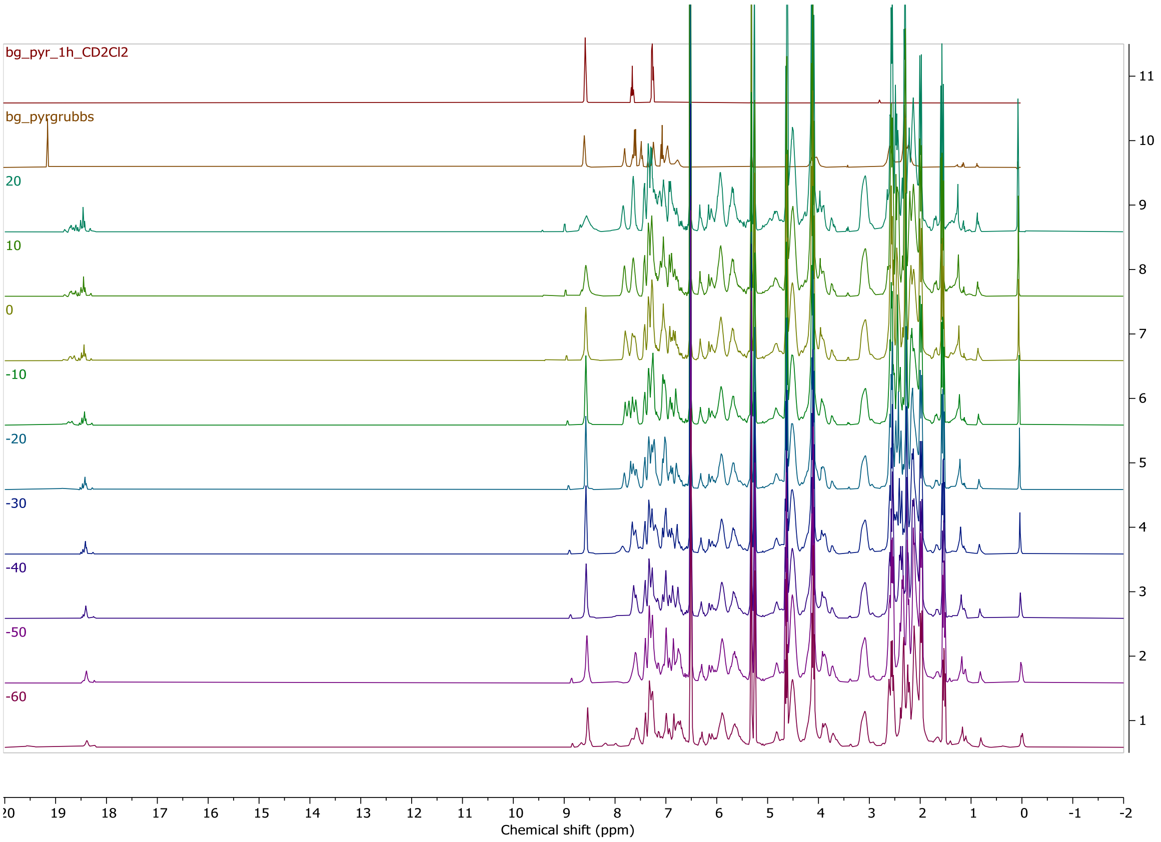

Figure S115: Stacked ^{1}H VT NMR spectra of **pM3**.

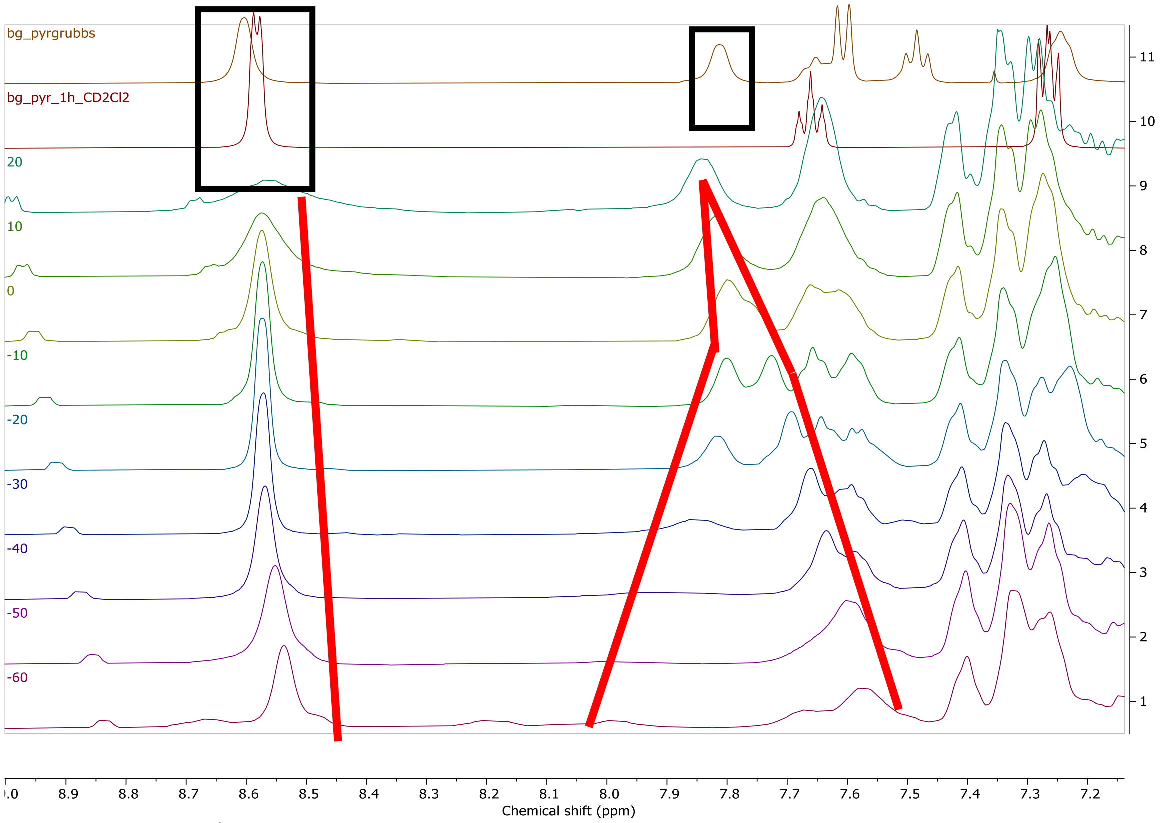

Figure S116: Stacked ^{1}H VT NMR spectra of **pM3** highlighting the temperature dependent shift of pyridine.

Eyring-Polanyi and Arrhenius analysis M1

In a glovebox monomer **M1** (164.3 mg, final concentration in reaction 0.068 M) was dissolved in 8 mL DCM. **GIII** (9.6 mg) was dissolved in 8 mL DCM and partitioned between seven screw cap vials (1 mL of catalyst solution each). All screwcap vials were placed in a 3x3 aluminium block and allowed to cool down in a glovebox integrated coldwell via an external kryostat. 1 mL of monomer stock solution was successively added to each vial and the reaction quenched with ethyl vinyl ether (2 mL) after definite time increments. The vials were brought outside the box and the solvent and excess quenching agent removed via rotary distillation. To the residues 1 mL of DMSO-d6 was added for successive NMR analysis (1H, relaxation delay = 5 s).

k_{obs} at four different temperatures were determined by plotting the natural logarithm of the integral area of selected resonances (~6.6 and ~6.1 ppm; corresponding to the formation of ring-opened product after quenching as discussed in main text) against the time.

A summary of the determined values is given in Table S2. The equations used for determining $\Delta H^{\ddagger}$, $\Delta S^{\ddagger}$ and E_a are included in the corresponding plot.

Table S2: Temperatures and determined kobs for Eyring and Arrhenius analysis for pM1.

**T / K**	***k_{obs}* / s^{-1}**	**1/T**	**ln(k/T)**	**ln(k)**
298	0.0343	0.0033557	-9.06970341	-3.37260992
284	0.0097	0.00352113	-10.2846036	-4.63562939
278	0.0027	0.00359712	-11.5421246	-5.91450351
267	0.0011	0.00374532	-12.3996938	-6.8124451

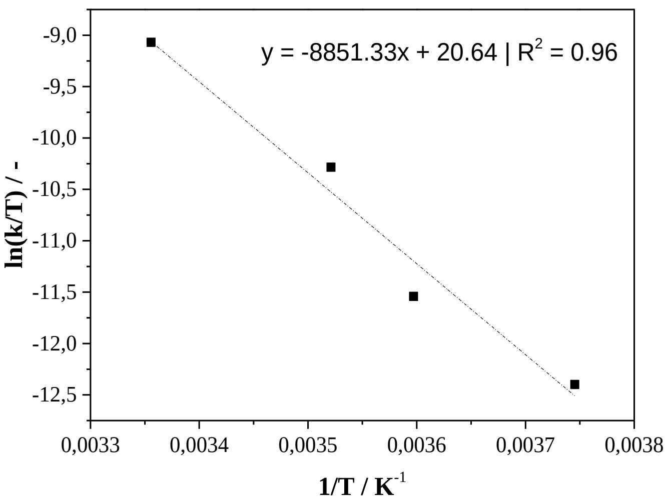

$$\boldsymbol{ln\frac{k}{T} = \frac{-\Delta H^{\neq}}{R}\frac{1}{T} + \frac{\kappa k_B}{h} + \frac{\Delta S^{\neq}}{R}}$$

$$\boldsymbol{\frac{-\Delta H^{\neq}}{R} = -8851.3\ K^{-1}}$$

$$\underline{\boldsymbol{\Delta H^{\neq} = 73.6\ kJ \cdot mol^{-1}}}$$

$$\boldsymbol{ln\left(\frac{k_B}{h}\right) + \frac{\Delta S^{\neq}}{R} = 20.641}$$

$$\underline{\boldsymbol{\Delta S^{\neq} = -25.9\ J \cdot mol^{-1} \cdot K^{-1}}}$$

Figure S117: Eyring-Polanyi plot of **M1** and according determination of enthalpy and entropy of activation.

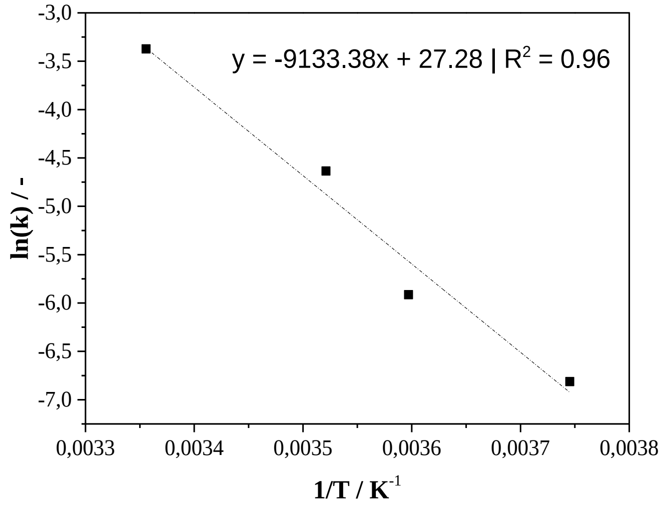

$$\boldsymbol{ln(k) = ln(A) - \frac{E_a}{R}\left(\frac{1}{T}\right)}$$

$$\boldsymbol{k = e^{27.28} - e^{-\frac{9133.38}{T}}}$$

$$\boldsymbol{\frac{E_a}{R} = -9133.4\ K}$$

$$\underline{\boldsymbol{E_a = 75.94\ kJ \cdot mol^{-1}}}$$

Figure S118: Arrhenius plot of **M1** and determination of activation energy.

Computational details for mechanistic considerations

Density functional theory (DFT) calculations were performed using the B3LYP[5–8] functional with all electron 6-311+G(d) basis set[9,10] on all atoms as implemented in Gaussian 16 C.01 program.[11] Grimme's D3 correction scheme with Becke-Johnson damping was used in all calculations. Nature of the stationary points was confirmed by the vibrational analysis carried out at the same level of theory. All structures corresponding to local minima showed no imaginary frequencies. Reaction Gibbs free energies (ΔG_{298K}) were computed using the results of the normal-mode analysis within the ideal gas approximation at a pressure of 1 atm and temperature of 298.15 K.

Conformational analysis

Monomers **Oxa**, **M1**, **M2** and **M3** are rigid structures. In **Open** free rotation is possible around two C-C bonds; conformational analysis has not been performed on this structure with the assumption that the differences in conformer stability would not be greater that the accuracy of the computational method. The ethylene adducts are more flexible structures, several conformers are possible

Assignment of IR vibrations was done through frequency analysis of qM1, qM2 and qM3 on B3LYP-D3/6-31G(d,p) level of theory. Gaussian 16 was used as the software package.

Table S4: Coordinates of optimized geometries of **qM1**, **qM2** and **qM3**.

**qM1**
C 3.77566 2.47451 0.28568
C 2.51900 2.21668 0.64192
C 1.48758 1.49859 -0.18350
O 0.67546 0.72749 0.72439
C 0.08972 -0.37572 0.00084
C 1.24158 -0.79123 -0.92852
C 1.99619 0.50336 -1.26291
C 0.00656 -1.54953 0.98664
O 1.31712 -2.14222 1.01721
C 2.06435 -1.72763 -0.04205
O 3.20429 -2.07486 -0.20721
C -1.18369 0.02603 -0.68434
C -2.40870 -0.28955 -0.25596
O -3.52578 0.09627 -0.92473
C -4.76291 -0.04093 -0.21283
C -4.95179 1.06553 0.81654
H 4.44100 3.03046 0.93864
H 4.19229 2.14948 -0.66333
H 2.13994 2.57013 1.59919
H 0.84228 2.25243 -0.66521
H 0.91378 -1.34748 -1.81187
H 1.76283 0.85712 -2.26973
H 3.07266 0.32807 -1.20344
H -0.71018 -2.30754 0.65665
H -0.24050 -1.21343 1.99540
H -1.09293 0.66017 -1.56257
H -2.58160 -0.89620 0.63472
H -4.81218 -1.03331 0.25968
H -5.53895 0.00057 -0.98151
H -4.17117 1.02461 1.58269
H -5.92340 0.96542 1.31103
H -4.90397 2.04529 0.33341
**qM2**
C 2.45681 0.35315 0.98880
C 2.40603 -1.00636 0.28263
O 1.46679 -0.81757 -0.78928
C 0.43796 0.07824 -0.34849
C 0.99075 0.80740 0.93107
C 0.29798 1.25888 -1.33266
O -0.10155 2.36032 -0.52382
C 0.72262 2.30357 0.64602
C -0.84155 -0.68491 -0.11126
C -2.05241 -0.12173 -0.10453
C 3.68324 -1.53762 -0.30045
C 4.91101 -1.15845 0.04921
O -3.17873 -0.83778 0.17590
C -4.41328 -0.15953 -0.07004
C -5.53982 -1.07998 0.36143
H 3.09614 1.03023 0.41113
H 2.85372 0.28904 2.00568
H 1.99905 -1.76169 0.98171
H 0.44510 0.47210 1.81561
H -0.45979 1.10571 -2.10310
H 1.27597 1.43378 -1.80836
H 0.18668 2.81394 1.45033
H 1.66878 2.83874 0.46589
H -0.73769 -1.74428 0.10636
H -2.19057 0.94322 -0.29305
H 3.54042 -2.31349 -1.05062
H 5.08817 -0.39083 0.79753
H 5.79099 -1.60984 -0.39853
H -4.49796 0.08909 -1.13832
H -4.44146 0.78456 0.49513
H -5.50624 -2.01485 -0.20483
H -6.50896 -0.60172 0.18991
H -5.45058 -1.31924 1.42479
**qM3**
C -4.42863 -1.69979 0.49520
C -3.16405 -1.95360 0.16418
C -2.27640 -1.05665 -0.65104
O -0.95445 -1.08211 -0.07364
C -0.30964 0.18842 -0.26741
C -1.30530 1.09052 -1.03111
C -2.65503 0.42541 -0.72548
C -0.19695 0.91885 1.09293
O -0.70481 2.17978 0.98425
C -1.09175 2.45656 -0.37915
C 1.02365 0.02117 -0.93104
C 2.19631 0.08474 -0.28874
O 3.37265 -0.06774 -0.94345
C 4.53454 -0.20708 -0.11145

C 4.63631 -1.60289 0.48850	H 2.25889 0.27469 0.78158
H -5.01592 -2.41114 1.06733	H 4.52197 0.56132 0.67471
H -4.93005 -0.77942 0.20820	H 5.38121 0.00141 -0.77085
H -2.69341 -2.88761 0.46483	H 3.78564 -1.80813 1.14558
H -2.20816 -1.46567 -1.67545	H 5.55490 -1.69856 1.07654
H -1.10331 1.11492 -2.10532	H 4.64721 -2.35673 -0.30368
H -3.41995 0.63685 -1.47683	O 0.25273 0.49364 2.12478
H -3.02587 0.75611 0.25119	H -0.28358 3.01504 -0.86433
H 1.01353 -0.21268 -1.99253	H -1.99055 3.07702 -0.34883

Table S5. Optimized structures and energies of monomers

Monomer	Optimized geometry[a]	H (a.u.)	G (a.u.)
**Oxa**	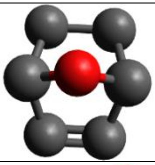	-308.585614	-308.620025
**M1**	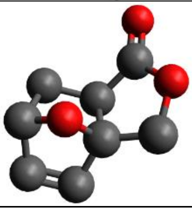	-535.304964	-535.345981
**M3**	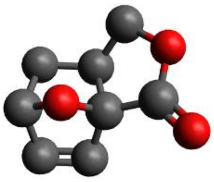	-535.297244	-535.33832
**M2**	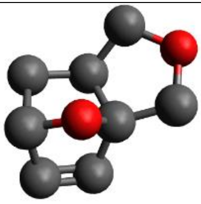	-461.221347	-461.261021
**Open**	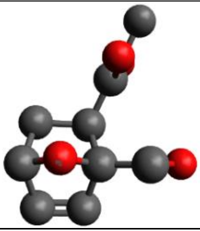	-651.021257	-651.073607

[a] H atoms omitted for clarity.

Table S6. Optimized structures and energies of ethylene adducts

Monomer	Optimized geometry[a]	H (a.u.)	G (a.u.)
**Ethylene**	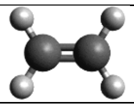	-78.557491	-78.583002
**Oxa**	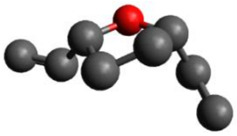	-387.172843	-387.218329
**M1**	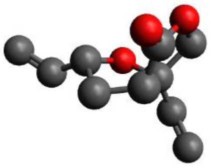	-613.89747	-613.949822
**M3**	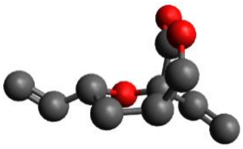	-613.890374	-613.941791
**M2**	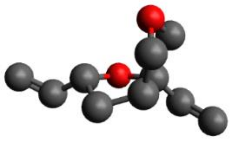	-539.812712	-539.862688
**Open**	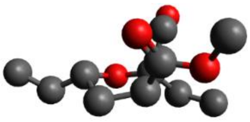	-729.605802	-729.667698

[a] H atoms omitted for clarity.

Table S7. Coordinates of optimized structures

**Ethylene**			
C	-0.00000	0.66432	0.00000
H	0.92272	1.23668	-0.00000
H	-0.92268	1.23673	0.00000
C	-0.00000	-0.66432	0.00000
H	-0.92272	-1.23668	-0.00000
H	0.92268	-1.23673	0.00000
Monomers			
**Oxanorbornene**			
C	-1.29001	-0.66675	-0.46772
C	-0.08088	-1.07057	0.36581
C	-0.08054	1.07057	0.36586
C	-1.28971	0.66714	-0.46783
C	1.18375	-0.77814	-0.50295
H	2.07412	-1.17420	-0.01182
H	1.11675	-1.21455	-1.50074
C	1.18406	0.77783	-0.50282
H	2.07454	1.17347	-0.01155
H	1.11730	1.21443	-1.50054
H	-0.10675	-2.03328	0.87068
H	-0.10617	2.03326	0.87078
O	-0.01026	-0.00004	1.32450
H	-1.92415	-1.34216	-1.02739
H	-1.92355	1.34276	-1.02758
**M1**			
C	1.61790	-0.82226	0.66389
C	1.69669	0.98712	-0.73515
C	0.36500	0.75705	-0.04888
O	0.76992	0.19919	1.21616
H	3.46907	-0.27478	-0.63709
C	2.47307	-0.01163	-0.30651
H	1.89970	1.72284	-1.50198
H	2.12813	-1.36647	1.45439
C	-0.76682	1.73738	0.11838
O	-1.95865	0.92360	0.21869
C	-0.30904	-0.48986	-0.69302
C	0.57057	-1.64216	-0.16549
H	-0.86694	2.40094	-0.74421
H	-0.68539	2.32522	1.03140
H	1.02077	-2.23504	-0.96161
H	0.00513	-2.30667	0.48828
H	-0.34759	-0.43759	-1.78390
C	-1.73041	-0.36855	-0.16793
O	-2.57685	-1.21165	-0.10197
**M3**			
C	-1.86734	0.08005	0.66703
C	-0.87183	-1.41161	-0.75208
C	0.06180	-0.43630	-0.07708
O	-0.56754	-0.23674	1.20491
H	-3.04549	-1.46205	-0.61420
C	-2.08401	-1.07451	-0.30478
H	-0.59196	-2.13285	-1.50691
H	-2.58935	0.20832	1.46963
O	2.11683	0.64843	0.03023
C	-0.12523	0.98023	-0.70087
C	-1.52287	1.36529	-0.16344
H	-2.24540	1.56638	-0.95479
H	-1.48321	2.23420	0.49637
H	-0.07460	0.95120	-1.79032
C	1.10556	1.68433	-0.12359
C	1.56299	-0.59289	0.06231
H	0.91213	2.10967	0.86204
H	1.53562	2.44390	-0.77437

O	2.20419	-1.59748	0.16080
**M2**			
C	-1.65775	0.48956	0.60383
C	-1.09709	-1.29877	-0.69736
C	0.06590	-0.63890	0.01518
O	-0.55648	-0.18004	1.23569
H	-3.18331	-0.66735	-0.71812
C	-2.17101	-0.58802	-0.34395
H	-1.01932	-2.08779	-1.43394
H	-2.34938	0.88075	1.34644
C	1.43440	-1.23730	0.25439
O	2.38998	-0.20791	-0.04795
C	0.34054	0.73790	-0.69400
C	-0.88759	1.55894	-0.24970
H	1.64007	-2.08834	-0.39883
H	1.53005	-1.55485	1.29730
H	-1.47730	1.94193	-1.08353
H	-0.60547	2.39961	0.38783
H	0.41217	0.61782	-1.77630
C	1.71325	1.05390	-0.10564
H	1.61634	1.48104	0.90017
H	2.32420	1.71697	-0.71943
**Open**			
C	1.96045	-1.36248	0.43862
C	2.17215	0.46169	-0.90045
C	1.03203	0.54240	0.11320
O	1.55643	-0.22057	1.20785
C	2.75190	-0.72026	-0.69246
H	2.46597	-2.08921	1.07014
C	0.58759	1.90800	0.57506
C	-0.06378	-0.43136	-0.47781
C	0.58810	-1.81292	-0.14795
H	-0.07806	1.79586	1.43352
H	0.67771	-2.45446	-1.02494
H	0.02308	-2.34198	0.62074
H	-0.16994	-0.28192	-1.54959
C	-1.42904	-0.29538	0.14851
O	-1.66848	-0.04762	1.30562
H	1.46750	2.48943	0.87355
O	-0.08226	2.51635	-0.53205
H	-0.45097	3.36147	-0.25416
C	-3.74509	-0.45048	-0.28656
H	-4.37465	-0.66897	-1.14585
H	-3.90856	-1.18172	0.50576
H	-3.95687	0.54705	0.09965
O	-2.39523	-0.52379	-0.77024
H	2.35419	1.19021	-1.67890
H	3.54105	-1.19598	-1.26025
Ethylene adducts			
**Oxanorbornene+ethylene**			
C	1.10180	0.44422	0.20178
C	0.24258	1.06483	-0.91457
C	-1.04538	1.44137	-0.17769
C	-1.18732	0.28790	0.81868
O	0.16662	-0.03966	1.19320
C	-1.86578	-0.95885	0.30460
C	1.97884	-0.67659	-0.26038
H	1.71462	1.21663	0.68214
H	0.03513	0.32195	-1.68908
H	0.74301	1.91165	-1.38665
H	-1.90545	1.55903	-0.83718
H	-1.64350	-1.84170	0.90071
H	1.45167	-1.53719	-0.66873
C	-2.67109	-1.06690	-0.74806
H	-2.92970	-0.22327	-1.37993
C	3.30731	-0.66112	-0.20653
H	3.84865	0.18324	0.21170

H	3.90557	-1.48748	-0.57517
H	-3.11407	-2.01923	-1.01989
H	-1.68813	0.60537	1.73985
H	-0.91664	2.37916	0.36967
**M1+ethylene**			
C	-1.49986	-0.19696	0.07923
C	-0.77036	-0.32898	-1.26451
C	0.69465	-0.27556	-0.84025
C	0.70541	0.68211	0.35654
O	-0.63108	0.62281	0.89651
C	1.18573	-1.59933	-0.25612
O	1.69856	-1.39745	0.98405
C	1.68089	0.00131	1.33359
C	1.03383	2.12941	0.11136
C	-2.84536	0.44633	-0.00958
H	-1.58816	-1.18195	0.55375
H	-1.00193	0.51961	-1.91295
H	-1.02429	-1.25059	-1.78811
H	1.36407	0.01331	-1.65139
H	1.34498	0.08844	2.36533
H	2.69496	0.39286	1.23187
H	0.62925	2.79619	0.86908
H	-2.84674	1.46964	-0.38051
C	1.74242	2.62533	-0.89771
H	2.16557	2.00537	-1.68184
C	-3.98319	-0.15946	0.31459
H	-3.99726	-1.17559	0.69853
H	-4.94323	0.33368	0.20781
H	1.93156	3.69005	-0.97811
O	1.13330	-2.68115	-0.76759
**M3+ethylene**			
C	1.58475	-0.02193	0.05939
C	0.99047	-0.23060	1.46897
C	-0.53299	-0.05467	1.28025
C	-0.64612	0.45091	-0.17406
O	0.65049	0.86424	-0.57916
C	-1.37022	-1.33718	1.28449
O	-1.43953	-1.80083	-0.08213
C	-1.06070	-0.83412	-0.95416
C	-1.62346	1.54109	-0.49415
C	2.94233	0.59739	0.04393
H	1.60207	-0.97409	-0.48814
H	1.37418	0.53597	2.14549
H	1.26419	-1.20329	1.88176
H	-0.92740	0.66358	1.99729
H	-0.93615	-2.14391	1.87470
H	-2.39253	-1.15875	1.62549
H	-1.49727	1.95465	-1.49065
H	2.99946	1.60001	0.46346
C	-2.60524	1.96343	0.29539
H	-2.77604	1.56247	1.28923
C	4.03171	-0.00867	-0.41655
H	3.98973	-1.00298	-0.85186
H	5.00627	0.46575	-0.38011
H	-3.28967	2.73812	-0.03134
O	-1.04596	-0.99235	-2.13825
**M2+ethylene**			
C	-1.46769	0.03701	0.00507
C	-0.81098	0.21440	1.37953
C	0.66800	0.42045	1.03776
C	0.83880	-0.36267	-0.30735
O	-0.49109	-0.70657	-0.74033
C	1.04899	1.86554	0.65972
O	0.94333	1.95118	-0.75954
C	1.43683	0.70944	-1.24275
C	1.63503	-1.63694	-0.27352
C	-2.75041	-0.72893	0.02739
H	-1.61982	1.01410	-0.46909
H	-0.93981	-0.70066	1.96500
H	-1.24463	1.04142	1.94519
H	1.31880	0.03177	1.82006
H	0.38999	2.62253	1.08716
H	2.08129	2.08526	0.96828
H	1.11458	0.58156	-2.27513
H	2.53459	0.68722	-1.18970
H	1.22899	-2.42565	-0.90129
H	-2.67075	-1.75644	0.37798
C	2.75801	-1.83888	0.40948
H	3.19850	-1.07666	1.04615
C	-3.92856	-0.22385	-0.32493
H	-4.02407	0.79451	-0.69130
H	-4.84175	-0.80591	-0.26221
H	3.28803	-2.78335	0.35552
**Open+ethylene**			
C	-1.96326	-0.45059	-0.00004
C	-1.04257	-1.16937	-0.99552
C	0.30298	-0.44023	-0.84467
C	-0.08448	0.91678	-0.11656
O	-1.51374	0.90715	-0.05731
C	1.32039	-1.25937	-0.08003
C	0.50082	1.00478	1.31297
C	0.30657	2.14775	-0.90147
C	-3.41412	-0.50138	-0.34983
H	-1.80636	-0.85468	1.00813
H	-1.42572	-1.03317	-2.00934
H	-0.97114	-2.23654	-0.78965
H	0.73795	-0.19562	-1.81151
H	1.58804	0.86546	1.25962
H	-0.49784	2.86654	-1.03003
H	-3.68391	-0.00403	-1.27966
C	1.52329	2.39382	-1.37992
H	2.34091	1.68767	-1.26683
C	-4.33619	-1.12209	0.37868
H	-4.08662	-1.61297	1.31505
H	-5.37484	-1.16398	0.06900
H	1.74098	3.31037	-1.91723
O	1.07103	-2.10829	0.74065
H	0.08721	0.21159	1.93543
O	0.18685	2.21783	1.96801
H	0.57931	2.94718	1.47066
O	2.58069	-0.91082	-0.42338
C	3.63320	-1.58692	0.28680
H	3.57194	-2.66264	0.12327
H	4.56013	-1.18904	-0.11857
H	3.56146	-1.38213	1.35548

Table S8. Total dipole moments as extracted from the geometry optimizations

Monomer	Total Dipole moment / D
M1	5.3405
M2	2.8390
M3	6.0719

References

(1) Love, J. A.; Morgan, J. P.; Trnka, T. M.; Grubbs, R. H. A Practical and Highly Active Ruthenium-Based Catalyst That Effects the Cross Metathesis of Acrylonitrile. *Angew. Chem. Int. Ed.* **2002**, *41* (21), 4035–4037. https://doi.org/10.1002/1521-3773(20021104)41:21<4035::AID-ANIE4035>3.0.CO;2-I.

(2) Lancefield, C. S.; Fölker, B.; Cioc, R. C.; Stanciakova, K.; Bulo, R. E.; Lutz, M.; Crockatt, M.; Bruijnincx, P. C. A. Dynamic Trapping as a Selective Route to Renewable Phthalide from Biomass-Derived Furfuryl Alcohol. *Angew. Chem. Int. Ed.* **2020**, *59* (52), 23480–23484. https://doi.org/10.1002/anie.202009001.

(3) Cioc, R. C.; Harsevoort, E.; Lutz, M.; Bruijnincx, P. C. A. Efficient Synthesis of Fully Renewable, Furfural-Derived Building Blocks via Formal Diels–Alder Cycloaddition of Atypical Addends. *Green Chem.* **2023**, *25* (23), 9689–9694. https://doi.org/10.1039/D3GC02357E.

(4) Wolf, W. J.; Lin, T.-P.; Grubbs, R. H. Examining the Effects of Monomer and Catalyst Structure on the Mechanism of Ruthenium-Catalyzed Ring-Opening Metathesis Polymerization. *J. Am. Chem. Soc.* **2019**, *141* (44), 17796–17808. https://doi.org/10.1021/jacs.9b08835.

(5) Vosko, S. H.; Wilk, L.; Nusair, M. Accurate Spin-Dependent Electron Liquid Correlation Energies for Local Spin Density Calculations: A Critical Analysis. *Can. J. Phys.* **1980**, *58* (8), 1200–1211. https://doi.org/10.1139/p80-159.

(6) Lee, C.; Yang, W.; Parr, R. G. Development of the Colle-Salvetti Correlation-Energy Formula into a Functional of the Electron Density. *Phys. Rev. B* **1988**, *37* (2), 785–789. https://doi.org/10.1103/PhysRevB.37.785.

(7) Becke, A. D. Density-functional Thermochemistry. III. The Role of Exact Exchange. *J. Chem. Phys.* **1993**, *98* (7), 5648–5652. https://doi.org/10.1063/1.464913.

(8) Stephens, P. J.; Devlin, F. J.; Chabalowski, C. F.; Frisch, M. J. Ab Initio Calculation of Vibrational Absorption and Circular Dichroism Spectra Using Density Functional Force Fields. *J. Phys. Chem.* **1994**, *98* (45), 11623–11627. https://doi.org/10.1021/j100096a001.

(9) McLean, A. D.; Chandler, G. S. Contracted Gaussian Basis Sets for Molecular Calculations. I. Second Row Atoms, Z=11–18. *J. Chem. Phys.* **1980**, *72* (10), 5639–5648. https://doi.org/10.1063/1.438980.

(10) Krishnan, R.; Binkley, J. S.; Seeger, R.; Pople, J. A. Self-consistent Molecular Orbital Methods. XX. A Basis Set for Correlated Wave Functions. *J. Chem. Phys.* **1980**, *72* (1), 650–654. https://doi.org/10.1063/1.438955.

(11) Frisch, M. J.; Trucks, G. W.; Schlegel, H. B.; Scuseria, G. E.; Robb, M. A.; Cheeseman, J. R.; Scalmani, G.; Barone, V.; Petersson, G. A.; Nakatsuji, H.; Li, X.; Caricato, M.; Marenich, A. V.; Bloino, J.; Janesko, B. G.; Gomperts, R.; Mennucci, B.; Hratchian, H. P.; Ortiz, J. V.; Izmaylov, A. F.; Sonnenberg, J. L.; Williams; Ding, F.; Lipparini, F.; Egidi, F.; Goings, J.; Peng, B.; Petrone, A.; Henderson, T.; Ranasinghe, D.; Zakrzewski, V. G.; Gao, J.; Rega, N.; Zheng, G.; Liang, W.; Hada, M.; Ehara, M.; Toyota, K.; Fukuda, R.; Hasegawa, J.; Ishida, M.; Nakajima, T.; Honda, Y.; Kitao, O.; Nakai, H.; Vreven, T.; Throssell, K.; Montgomery Jr., J. A.; Peralta, J. E.; Ogliaro, F.; Bearpark, M. J.; Heyd, J. J.; Brothers, E. N.; Kudin, K. N.; Staroverov, V. N.; Keith, T. A.; Kobayashi, R.; Normand, J.; Raghavachari, K.; Rendell, A. P.; Burant, J. C.; Iyengar, S. S.; Tomasi, J.; Cossi, M.; Millam, J. M.; Klene, M.; Adamo, C.; Cammi, R.; Ochterski, J. W.; Martin, R. L.; Morokuma, K.; Farkas, O.; Foresman, J. B.; Fox, D. J. Gaussian 16 Rev. C.01, 2016.
